# Synthesis and *in Vitro* Evaluation of Novel 5‐Nitroindole Derivatives as *c‐Myc* G‐Quadruplex Binders with Anticancer Activity

**DOI:** 10.1002/cmdc.202000835

**Published:** 2021-03-22

**Authors:** Vijaykumar D. Nimbarte, Julia Wirmer‐Bartoschek, Santosh L. Gande, Islam Alshamleh, Marcel Seibert, Hamid Reza Nasiri, Frank Schnütgen, Hubert Serve, Harald Schwalbe

**Affiliations:** ^1^ Institute for Organic Chemistry and Chemical Biology Center for Biomolecular Magnetic Resonance (BMRZ) Goethe University Frankfurt Max-von-Laue-Straße 7 60438 Frankfurt am Main Germany; ^2^ German Cancer Research Center and German Cancer Consortium Im Neuenheimer Feld 280 69120 Heidelberg Germany; ^3^ Department of Medicine 2 Hematology/Oncology University Hospital Frankfurt Goethe University Theodor-Stern-Kai 7 60596 Frankfurt am Main Germany; ^4^ Frankfurt Cancer Institute (FCI) Theodor-Stern-Kai 7 60596 Frankfurt am Main Germany

**Keywords:** *c-Myc*, G-quadruplex binders, nitroindoles, oncogene promoters, reactive oxygen species, structure-activity relationships

## Abstract

Lead‐optimization strategies for compounds targeting *c‐Myc* G‐quadruplex (G4) DNA are being pursued to develop anticancer drugs. Here, we investigate the structure‐activity‐ relationship (SAR) of a newly synthesized series of molecules based on the pyrrolidine‐substituted 5‐nitro indole scaffold to target G4 DNA. Our synthesized series allows modulation of flexible elements with a structurally preserved scaffold. Biological and biophysical analyses illustrate that substituted 5‐nitroindole scaffolds bind to the *c‐Myc* promoter G‐quadruplex. These compounds downregulate c‐Myc expression and induce cell‐cycle arrest in the sub‐G1/G1 phase in cancer cells. They further increase the concentration of intracellular reactive oxygen species. NMR spectra show that three of the newly synthesized compounds interact with the terminal G‐quartets (5′‐ and 3′‐ends) in a 2 : 1 stoichiometry.

## Graphical abstract

**Targeting DNA‐G‐quadruplexe**s: Fragment extension of indole fragments resulted in the synthesis of a series of pyrrolidine‐substituted 5‐nitroindole derivatives that bind to the c‐Myc G‐quadruplex DNA in a 2 : 1 stoichiometry. We could show that the compounds downregulate c‐Myc expression and induce cell‐cycle arrest in cancer cells.

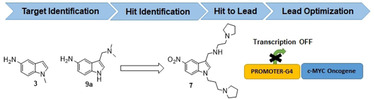

## Introduction

**Figure 1:**
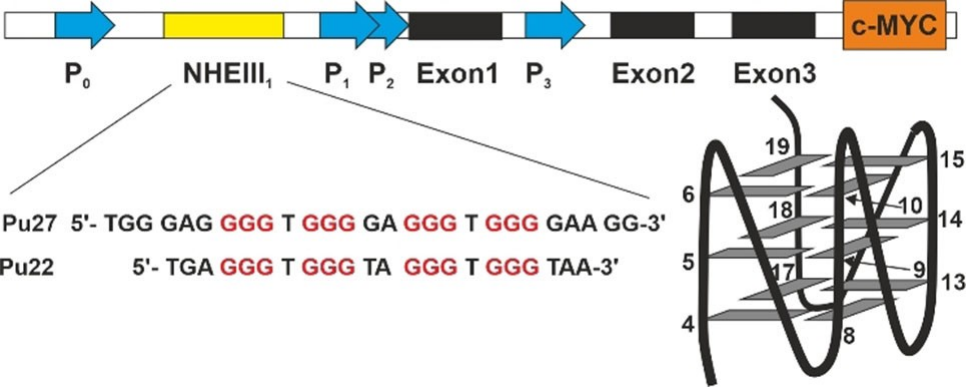
DNA sequence of the human *Myc* gene promoter. The G4‐forming region NHE III_1_ sequence is shown, a schematic structure of the P22 sequence is shown on the right.

DNA G‐quadruplexes (G4) are noncanonical DNA structures, found within guanine‐rich sequences.^[1]^ The basic structural unit is the G‐tetrad, which is stabilized by the association of four guanines into a cyclic Hoogsteen hydrogen‐bonding arrangement.[^2^, ^3^] Till date, more than 716 000 G4‐forming sequences have been identified in the human genomic DNA by G4‐sequencing techniques.^[4]^ G4 are implied in the regulation of human genes:[^4^, ^5^, ^6^] their formation is significantly associated with tumour suppressors and somatic copy number alterations related to cancer development.^[7]^ They are found in the promoter regions of oncogenes including *c‐Myc*, *c‐KIT*, *BCL‐2*, and in telomeres.[^8^, ^9^] G4 structures are interesting targets in cancer drug discovery. G4 stabilization in oncogene promoters by small molecules leads to down‐regulation of the expression of their respective genes.[^10^, ^11^, ^12^, ^13^] Up to 80 % of all solid tumours (including gastrointestinal, ovarian and breast tumours) overexpress c‐Myc.^[14]^ It has been proposed that the G4 present in NHE III_1_ in the c‐*Myc* gene is crucial for transcriptional silencing.[^15^, ^16^] This element is comprised of 27 nucleotides containing five G‐tracts of which G‐tracts 2, 3, 4 and 5 form a parallel G4 as the major conformation. In biophysical studies therefore a shortened version (Pu22) is used to facilitate investigations (Figure 1).^[17]^ It was shown that G4‐stabilizing ligands binding to Pu22 can downregulate c‐*Myc* transcription.^[15]^ Further, the c‐Myc protein‐dependent proliferation can be inhibited, leading to inhibition of cancer cell growth.^[18]^

In addition, redox homeostasis is essential for the maintenance of diverse cellular processes. Cancer cells have higher levels of reactive oxygen species (ROS) than normal cells as result of hypermetabolism. Recently, anticancer therapies that induce oxidative stress by increasing ROS and/or inhibiting antioxidant processes have received significant attention. The acceleration of accumulative ROS disrupts redox homeostasis and causes severe damage in cancer cells.^[19]^

A number of different ligands have been developed to target G4 DNAs including indolylmethyleneindanone, indenopyrimidine and bisbenzimidazole carboxamide derivatives of naphthyridine and phenanthroline,[^20^, ^21^] indoles,^[22]^ 7‐azaindoles,^[23]^ 1*H*‐indazol‐3‐yl,^[24]^ benzothiazole,^[25]^ imidazo[1,5‐*a*]pyridine,^[26]^ 2,6‐diaminopyrimidin‐4‐ol,^[27]^ 1*H*‐pyrazolo[4,3‐*d*]pyrimidin‐7‐amine,^[28]^ morpholino,^[28]^ bis‐indoles,[^29^, ^30^] 2‐hydroxynaphthalene‐1,4‐dione,^[31]^1,4‐dihydroxyanthracene‐9,10‐dione,^[32]^ benzofuran and piperonal, which were derived from several alkaloids.^[33]^

Herein, we describe pyrrolidine‐substituted 5‐nitroindole compounds as a new class of G4 ligands that bind to the *c‐Myc* promoter G4 sequence. These new compounds induce c‐Myc downregulation both, at transcription and translation level along with the generation of reactive oxygen species inducing antiproliferative effects in the cancerous cells. This novel pharmacophore was identified and improved by iterative fluorescence screening of series of substituted heterocycles. Our findings were validated using fluorescence intensity titrations, microscale thermophoresis (MST)‐based analyses, and subsequent evaluation in cellular assays and structural studies by NMR spectroscopy.

### Lead identification strategy: High‐throughput screening

**Scheme 1:**
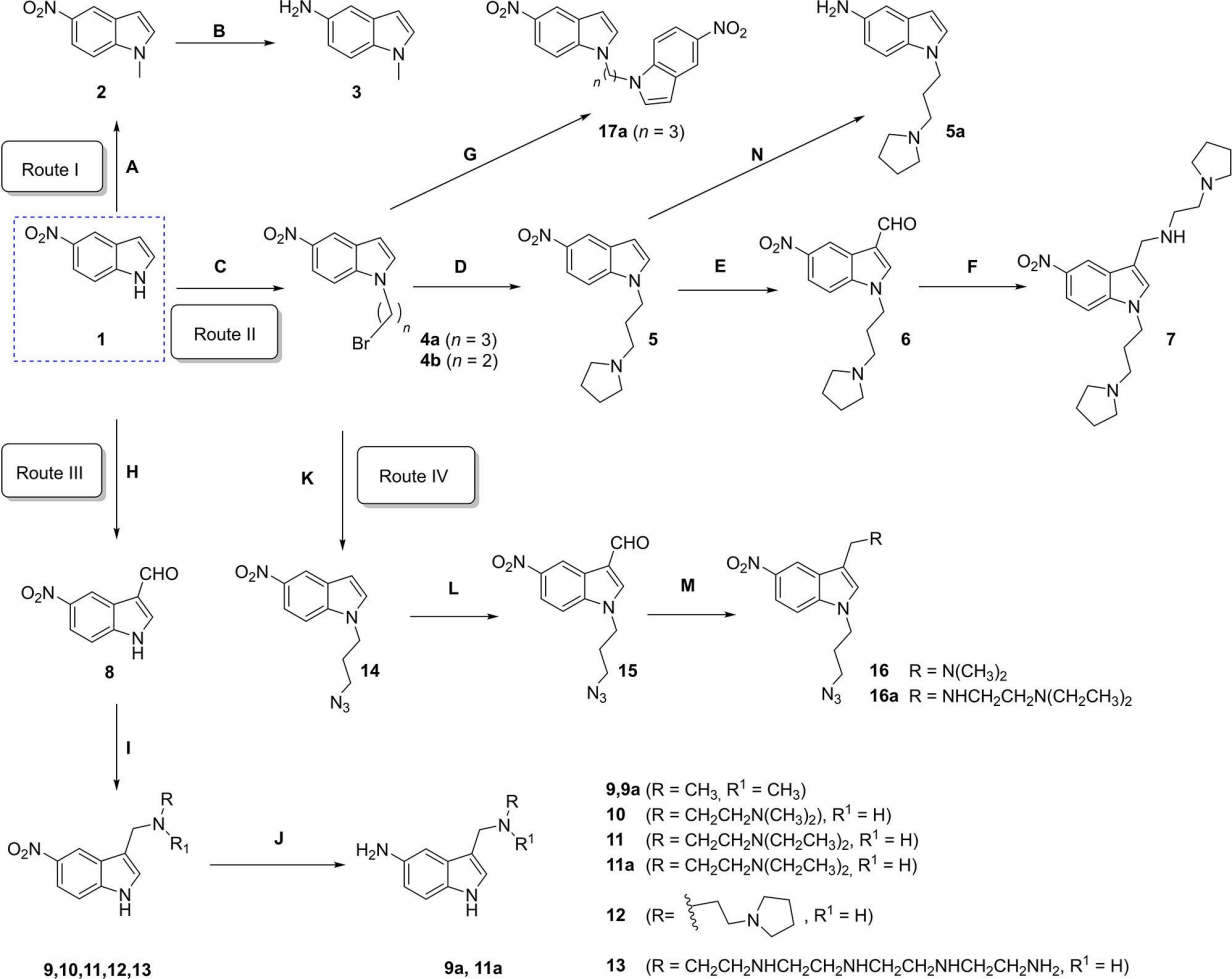
A) NaH, DMF, RT, 1 h, CH_3_I, RT, 8 h, 95 %; B) Pd/C, H_2_, MeOH, RT, 3 h, 52 %; C) K_2_CO_3_, DMF, 1,3‐dibromopropane and 1,2‐dibromoethane, RT, 4 h, 65 %; D) dry ACN, pyrrolidine, reflux, 4 h, 32 %; E) POCl_3_, DMF, 0 °C‐RT, 1 h, 46 %; F) NaBH_4_, MeOH, substituted amine, RT, 3 h, 72 %; G) K_2_CO_3_, DMF, RT, 3 h, 62 %; H) POCl_3_, DMF, 0 °C‐RT, 1 h, 30 %; I) NaBH_4_, MeOH, substituted amine, RT, 3 h, 56 %, J) Pd/C, H_2_, MeOH, RT, 3 h, 52 %; K) NaN_3_, DMF, 80 °C, reflux, 4 h, 48 %; L) POCl_3_, DMF, 0 °C‐RT, 1 h, 35 %; M) NaBH_4_, MeOH, corresponding amine, RT, 3 h, 45 %; N) Pd/C, H_2_, MeOH, RT, 3 h, 23 %.

Our investigations started from the parent compound 1‐methyl‐1*H*‐indol‐5‐amine (**3** in Scheme 1)^[34]^ and an initial library comprised of 129 structurally and chemically diverse commercially available fragments containing at least one aromatic moiety (Table S1 in the Supporting Information fragment no. A1–A129). Compounds in this fragment family followed principal criteria for fragment libraries (Figure S14, Tables S5–S7).[^35^, ^36^, ^37^]

The initial screening against the *c‐Myc* Pu22 DNA was conducted by a fluorescence intercalator displacement (FID) assay^[38]^ that utilizes thiazole orange (TO) as fluorescent dye. TO is a well‐validated probe for screening G4‐binding fragment molecules.^[39]^ It is highly fluorescent when bound. Its fluorescence is quenched after displacement (*λ*_ex_=501 nm, *λ*_em_=539 nm). Using this assay, we identified fragment **9** **a** (see Scheme 1<xschr1) and a general trend towards the fused 5‐ and 6‐membered heterocycles as suitable scaffolds (Figures S2 and S3).

### Synthetic strategy for lead optimization

**Scheme 2:**
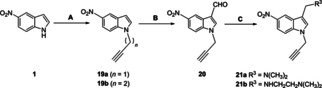
A) K_2_CO_3_, DMF, 3‐bromo‐1‐propyne/4‐bromobut‐1‐yne, RT, 4 h, 86–88 %; B) POCl_3_, DMF, 0 °C‐RT, 1 h, 58 %; C) NaBH_4_, MeOH, substituted amine, RT, 3 h, 38 %

**Scheme 3:**
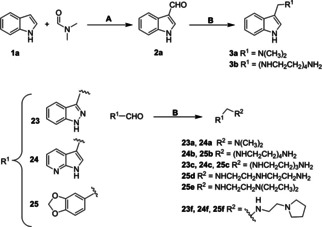
A) POCl_3_, DMF, −10 °C to RT, reflux, 100 °C, 1 h, 66 %; B) NaBH_4_, MeOH, substituted amine, RT, 3 h, 46 %

To further validate the findings of the screening of these commercially available fragments and to gain deeper insight into the structure‐activity‐relationship, several other indoles, 5‐nitroindoles, 5‐aminoindoles, 7‐azaindole and indazole derivatives were synthesized and tested (a total of 52 new compounds). We developed feasible synthetic strategies to generate drug‐like fragments derived from 5‐nitroindole (Schemes 1 and 2), from indole (Schemes 3 and S5) and from methylmethanamine derivatives (Scheme S4)

In route 1 of synthetic Scheme 1, scaffold 1‐methyl‐1H‐indol‐5‐amine (**3**) was obtained in 60 % yield by Pd/C‐catalysed hydrogenation of **2**, which is a nucleophilic substitution product of nitro‐indole **1**.^[40]^ In route II, intermediates **4** **a** and **b** were generated *via* a nucleophilic substitution reaction of **1** with 1,3‐dibromopropane and 1,2‐dibromoethane, respectively, which generates the 5‐nitroindole dimer **17** **a** as a by‐product of intermediate **4** **a**.^[41]^ In the next step, **4** **a** was protected with pyrrolidine to obtain **5**.The intermediate **5** was then treated with the Vilsmeier reagent to generate carbaldehyde **6** and to facilitate the one pot *in‐situ* generation of **7**. In route III, intermediate **8** was obtained in 60 % yield *via* Vilsmeier‐Haack reaction starting from 5‐nitro‐1H‐indole (**1**). This reaction was a key step to generate conjugates **9**–**13** in 56 % yields in a one pot *in‐situ* reaction of aldehydes with substituted amines in presence of NaBH_4_ as a reducing agent. Pd/C‐catalysed hydrogenation^[40]^ of the nitro group in **9** to the amine in **9** **a** and for the conversion of **5** to **5** **a** was applied. During the development of these synthetic strategies, we found that the substituted 5‐aminoindole derivatives are potentially unstable against air oxidation.

In addition to this, the indole derivatives **16** and **16** **a** were synthesized via route IV in a three‐step synthesis starting from intermediate **4** **a**. In a first step,**4** **a** was allowed to react with NaN_3_ in DMF to form the azide substituted derivative **14** in a nucleophilic substitution reaction in 60 % yield. Precursor **14** was then treated with the Vilsmeier reagent to obtain carbaldehyde **15** in 35 % yield. Conjugates **16** and **16** **a** were obtained from carbaldehyde **15** by one pot *in‐situ* reaction of the corresponding substituted amine with aldehyde group in presence of NaBH_4_ in methanol media with 45 % yield. In Scheme 2, 2‐propyne‐substituted nitro‐indoles **21** **a** and **21** **b** were prepared *via* substitution reaction of propargyl/butargyl bromide with 5‐nitroindole (**1**) to obtain intermediates**19a** and **19** **b**. Compound **20** could be synthesized from **19** **a** in a Vilsmeier–Haack reaction.

In addition to above in Scheme 3 several other aldehydes (**2** **a**, **23**, **24** and **25**) were used to prepare conjugates **3** **a**, **b**, **23** **a**, **b**, **e**, **24** **a**–**c**, **e** and **25** **b**, **d**, **e** by one pot *in‐situ* reaction of substituted amine with aldehyde in presence of NaBH_4_ in methanol media with 56 % yield. In addition, 7‐azaindole derivatives and 1‐(3‐(pyrrolidin‐1‐yl) propyl)‐1H‐indole were synthesized as described in Scheme 4 and Scheme 5 discussed in the Supporting Information.

### Evaluation of expanded hit series

**Figure 2:**
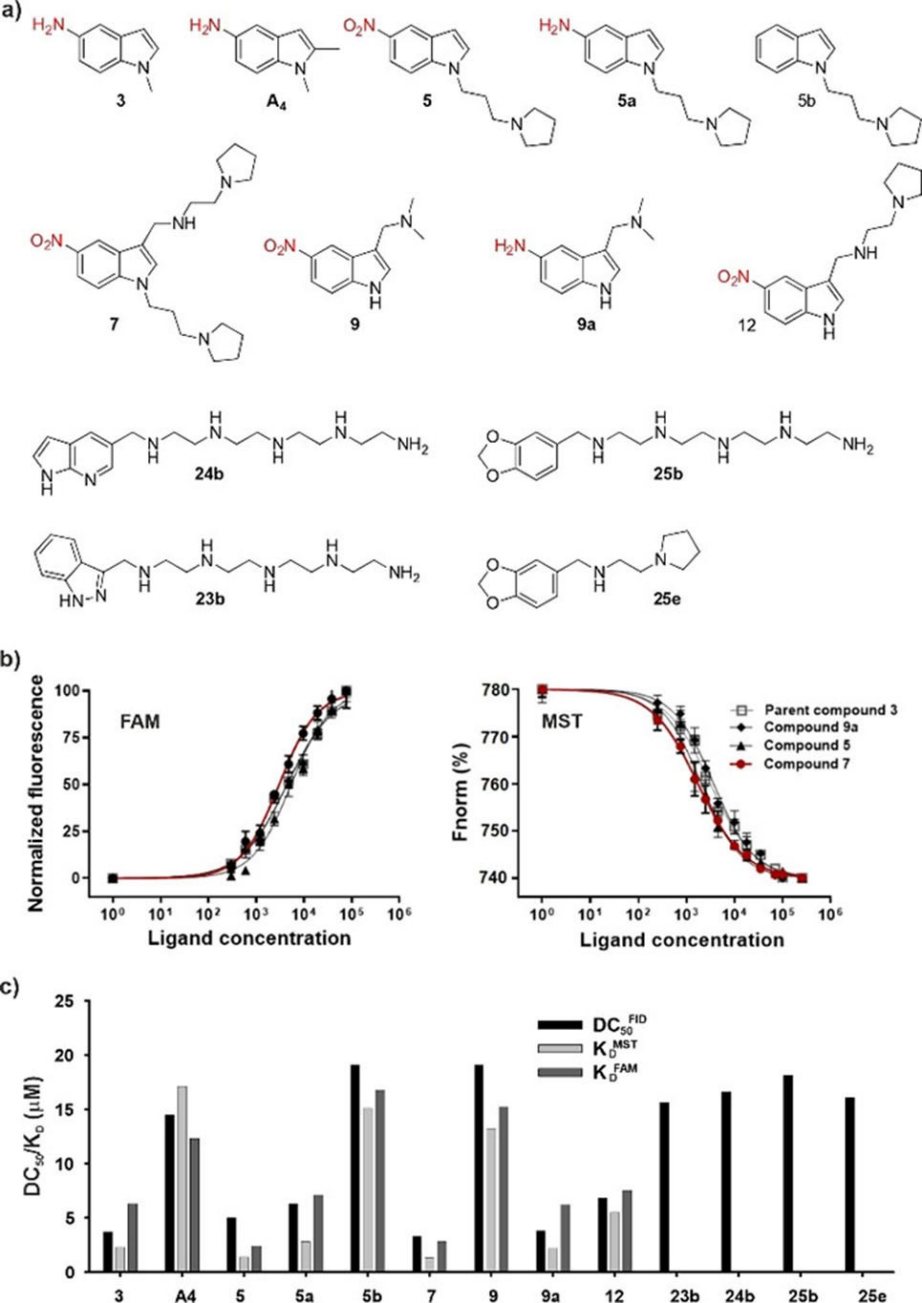
a) Chemical structure of best hits from initial FID experiments. b) Dose‐response curves representing DC_50_ and *K*_D_ results from FID titrations, MST measurements and FAM experiments for compound **7** of best hits from initial FID experiments. c) DC_50_ and *K*_D_ results from FID titrations, MST measurements and FAM experiments. Errors are within 5 %.

The 52 expanded and in‐house synthesized compounds were subjected to additional rounds of FID screening (Figure S4). The affinity of the 12 best fragments **9** **a**, **9**, **12**, **7**, **5, 5** **a**, **b**, **24** **b**, **25** **b**, **25**, **23** **b** and **A4** (Figures 2 and S5) along with parent fragment **3** ^[34]^ were further investigated in the TO assay.^[42]^ Indole containing compounds in general bind better than compounds containing another heterocycle such as **24** **b**, **25** **b**, **25** **e** and **23** **b**. Within the indoles, 5‐nitro indoles **12**, **7** and **5** as well as 5‐aminoindoles **3**, **9** **a** and **5** **a** bind with DC_50_ values lower than 10 μM. Substitution on the fifth position of the indole seems to be important for the activity: the 5‐nitro‐indole compound **9** binds more weakly than the corresponding 5‐aminoindole compound **9** **a** (aminogramine), while the unsubstituted indole compound **5** **b** binds more weakly than the corresponding nitro or amine substituted compounds **5** and **5** **a**, respectively. Further, substitution of position 2 reduces affinity as can be observed by the comparison of compound **3** with compound **A4**.

Microscale thermophoresis (MST)^[43]^ and fluorescence binding titrations were used to further evaluate the binding affinities of the indole compounds (**3**, **5**, **7**, **9** **a**, **9**,**12**, **A4**, **5** **a** and **5** **b**) to *c‐Myc* G4 (Figure 2b and c). Consistent with the FID measurements, comparison of compounds **5** (KMSTD =1.42 μM), **5** **a** (KMSTD =2.87 μM) and **5** **b** (KMSTD =15.1 μM) differing only at the fifth position suggests that nitro and amino functionalities at the fifth position of the indole heterocycle are beneficial for the binding. Most promising affinities with KMSTD of 1.32 and 1.42 μM; KFAMD of 2.4 and 2.83 μM were obtained for compounds **5** and **7**, respectively, both of which contain a methylene bridged pyrrolidine substitution at the first position of the indole skeleton, while compound **12** bearing the same side chain only at the third position of the indole has an approximately threefold higher *K*_D_ in both methods. Furthermore, based on MST assays, we observed that the 5‐nitroindole compounds (**5** and **7**) with methylene bridged pyrrolidine side‐chains are distinctly selective for *c‐Myc* G4 over the duplex DNA with *K*_d duplex_/*K*_d *c‐MYC*_ ranging from 24.12–29.23 (Figure S7 and Table S2). While in the case of the 5‐aminoindole compound (**5** **b**) the selectivity towards the *c‐Myc* G4 compared to the duplex DNA (*K*_d duplex_/*K*_d *c‐Myc*_) is reduced to the 6.55 (Figure S7).

Taken all three biophysical screens, we found that the amine and nitro functional group on the fifth position of central indole core plays a crucial role in improvement of G4 binding. In addition to these, the introduction of methylene bridged pyrrolidine at the third position along with the protection of N‐indole functional group plays crucial role in further improvement of binding affinities to *c‐Myc* G4.

### NMR characterization of binding

**Figure 3:**
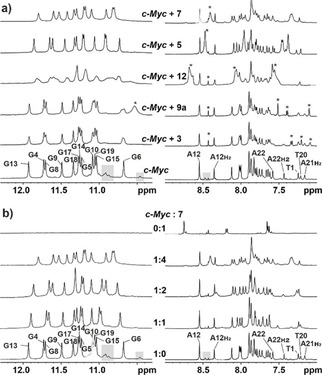
c‐Myc‐ligand interactions observed by ^1^H NMR spectra (imino and aromatic region). a) Comparison of interactions of c‐Myc with different fragments at a [DNA]/[ligand] ratio of 1 : 4. Signals clearly belonging to the respective ligand are indicated by asterisks. b) Interactions of fragment **7** with c‐Myc at different DNA/ligands ratios. a+b) assignments (17) of imino signals and clearly separated aromatic protons of loop and tail residues are indicated; grey boxes indicate signals of minor conformations; experimental conditions: 0.1 mM c‐Myc in 25 mM KPi, 70 mM KCl, pH 7, 0.025 mM DSS and 10 % [D_6_]DMSO.

The interactions of **3**, **5**, **7**, **9** **a** and **12** with *c‐Myc* were characterized by NMR spectroscopy. Figure 3a shows NMR spectra of the DNA‐ligand complexes at a ratio of 1 : 4 ([DNA]/[ligand]). In the spectrum of the DNA alone, signals (indicated by grey boxes) of minor conformations of the DNA are well visible. These signals disappear upon addition of ligand, indicating the stabilization of the major conformation upon binding. For ligands **3** and **9** **a**, only minor chemical shift perturbations and weak line broadening are observed corresponding to weak binding in fast exchange regime.

This differs drastically for ligand **12** where severe line broadening in combination with chemical shift changes are nicely visible, for example, for G13 of the DNA and indicate stronger interactions in the intermediate exchange regime. Strong chemical shift perturbations are observed for **5** and even more so for **7** coupled to only slight line broadening, allowing a detailed examination of the DNA‐ligand interactions as shown for **7** in Figure 3b.

Already at a [DNA]/[ligand] ratio of 1 : 1, signals of minor conformations have disappeared and shifting of a significant number of signals rather than appearance of new signals indicate binding in fast exchange. Most prominent chemical shift perturbations with values (positive and negative) around 0.06 ppm at ratio 1 : 1 are observed for imino‐signals of G19, G15 and G6, located at the 3’‐tetrad of G4 (see also Figure 5a). Perturbations between 0.034 and 0.045 ppm are observed for the imino‐signals of G4, G8 and G13, while all other perturbations are in magnitude smaller than 0.022 ppm. G4, G8 and G13 belong to the 5′‐tetrad. These findings indicate that the ligand binds to both, the 3′ and the 5′ tetrad with slight preference for the 3′‐tetrad. This trend is also seen at a concentration ratio of 1 : 2 where CSPs for the respective 6 imino‐signals are between 0.68 and 1.2 ppm in magnitude while they are less than 0.037 ppm for the other iminos. Binding and structural rearrangement of residues in loop and capping structures can be followed by analysing the aromatic NMR resonances. Signals of A12, A22, T1, T20 and A21 are reasonably well resolved to allow the assessment of conformational changes upon binding. As A12 does not shift upon binding the loop does not change its environment upon binding. This is different for T1, T20, A21 and A22. These residues located in the capping structures (see Figure 5a) are perturbed significantly upon binding indicating rearrangement of the capping structures to accommodate the ligands. This rearrangement is also seen in the literature for *c‐Myc*,^[44]^ as well as for other G4s.^[45]^

In agreement with the above NMR investigations the binding stoichiometry of 2 ligands per DNA was determined by additional fluorescence measurements for fragment **7** (Figure S8).

### In vitro characterization of DNA‐ligand interaction

**Figure 4:**
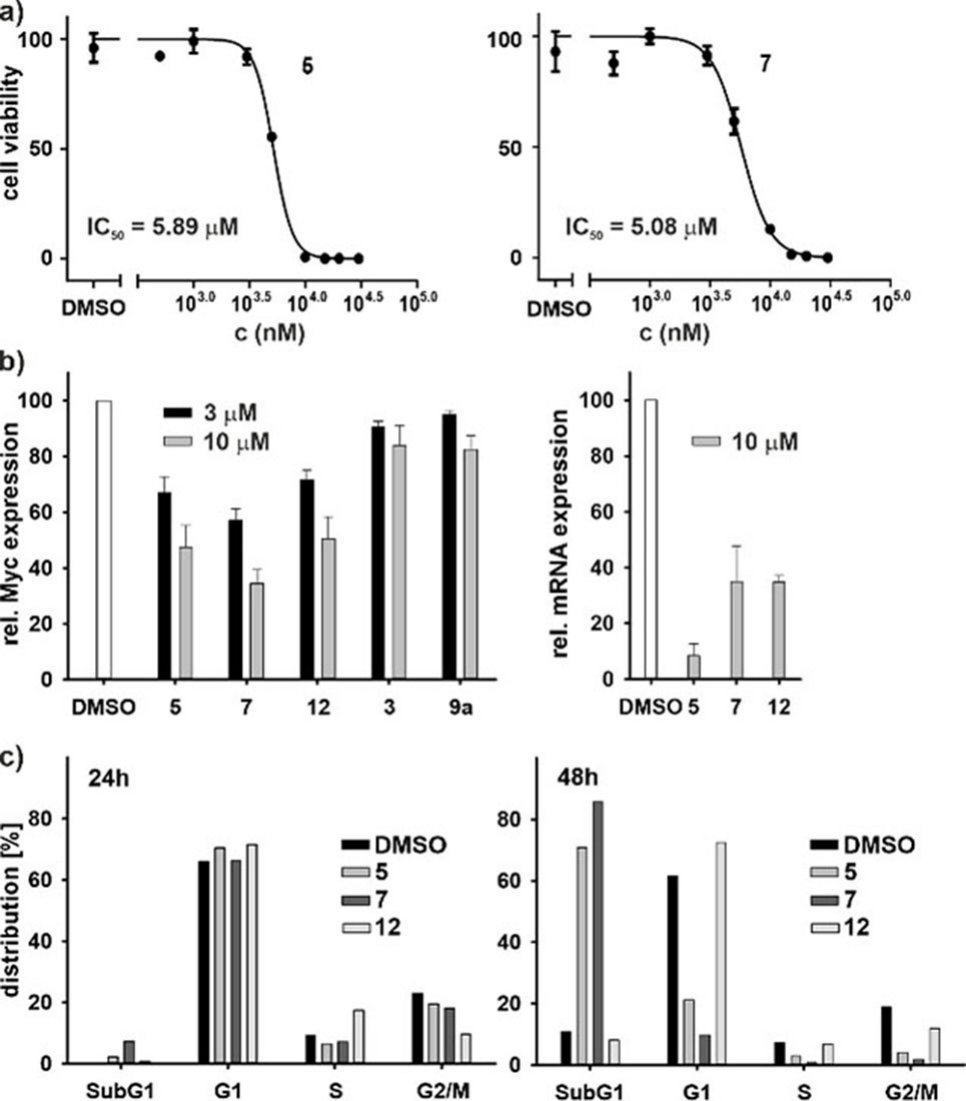
Cell cytotoxicity assays for tested ligands in HeLa cells: a) Dose‐response and IC_50_values derived for tested ligands in HeLa cells after 72 h of treatment. b) Western blot analysis (see also Figure S6) and qRT‐PCR show transcriptional downregulation of the *c‐Myc* gene, by tested ligands at defined concentrations. c) Hoechst staining‐mediated cell cycle analysis of HeLa cells treated with fragments **5**, **7** and **12** at 6 μM concentrations for 24 and 48 h (see also Figure S7 and Tables S2 and S3). Error bars represent the standard error of the mean calculated from three replicates.

The above biophysical and structural results show that the optimized ligands have a high binding affinity for the *c‐Myc* promoter G‐quadruplexes. To investigate the effect of these indole fragments on cancer cell proliferation, all identified hits from high throughput FID screening (**3**, **9** **a**, **9**, **12**, **7**, **5**, **5** **a**, **5** **b**, **24** **b**, **25** **b**, **25** **e**, **23** **b** and **A4**) were analysed *in vitro* by cell viability assays using HeLa cells^[46]^ (Figure 4a). Substituted 5‐nitroindoles derivatives have been found to show broad‐spectrum anticancer activities against different cancer cell lines.^[47]^ The anti‐proliferation effects of substituted 5‐nitroindole derivatives against human cancer HeLa cells were further investigated by Alamar blue assay, as shown in Figure 4a. Among all the ligands, **5** and **7** inhibited cell proliferation most efficiently with an IC_50_ value of 5.08±0.91 μM and 5.89±0.73 μM respectively (Table 1 and Figure 4a). To determine the potential role of ROS in the anticancer activity of 5‐nitroindole compounds, we also detect the effects of N‐acetyl cysteine (NAC) after treatment of HeLa cells with compound **7** (Figure S12). The viability of HeLa cells was 44.6±2.5 % after 5 μM of compound **7** treatment, which was reversed to be 77.8±1.9 % by the 1‐h pretreatment with 2.5 mM NAC.

**Table 1:** Characteristic properties of 5‐nitroindole compounds obtained by different biophysical and cellular assays.

	**3**	**9** **a**	**12**	**7**	**5**	**5** **a**	**5** **b**	**A4**	**9**	**25** **b**	**24** **b**	**25** **e**	**23** **b**
DC_50_^FID^ [μM]	3.71±0.32	3.83±0.11	6.81±0.9	3.30±0.86	5.01±0.15	6.31±0.43	19.12±.92	14.53±0.61	19.11±0.88	18.11±1.69	16.63±1.93	16.11±1.33	15.63±1.08
*K*_D_^MST^ [μM]	2.32±0.41	2.21±0.89	5.52±0.61	1.30±0.21	1.49±0.12	2.86±0.33	15.11±0.30	17.18±0.54	13.22±0.80	N.D.	N.D.	N.D.	N.D.
*K*_D_^FAM^ [μM]	6.30±0.63	6.29±0.71	7.53±0.93	2.81±0.37	2.43±0.29	7.12±0.45	16.87±1.58	12.32±0.94	15.21±1.93	N. D	N. D	N.D.	N.D.
IC_50_ [μM]	>45	>45	>45	5.08±0.91	5.89±0.7	>50	>50	>50	>50	>50	>50	>50	>50
NMR Shifts	+	+	+++	++++	+++	N.D.	N.D.	N.D.	N.D.	N.D.	N.D.	N.D.	N.D.
Rel. mRNA *Myc*	N.D.	N.D.	35 %	35 %	8 %	N.D.	N.D.	N.D.	N.D.	N.D.	N.D.	N.D.	N.D.
rel. protein Myc	84 %	83 %	51 %	35 %	48 %	N.D.	N.D.	N.D.	N.D.	N.D.	N.D.	N.D.	N.D.
sub‐G1	N.D.	N.D.	8 %	71 %	86 %	N.D.	N.D.	N.D.	N.D.	N.D.	N.D.	N.D.	N.D.

DCFID50 : values based on displacement of TO; *K*_D_ MST: *K*_D_ values from MST measurements; *K*_D_ FAM: values obtained from fluorescence titrations of FAM‐labelled *cMyc*; NMR: qualitative interpretation of NMR results in the fast to intermediate exchange regime: +weak chemical shift deviation, +++chemical shift deviation or strong line broadening, ++++strong chemical shift deviations; IC_50_ values based on cell viability assays; rel. mRNA *Myc*: mRNA level from qRT‐PCR after treatment with 10 μM fragment; rel. protein Myc: relative protein expression from western‐blot analysis after treatment with 10 μM fragment; Sub‐G1: cells being in the Sub‐G1 phase after 48 h of treatment with compound‐ for comparison: 10.8 % of control cells are in Sub‐G1 at this point; N.D.=not determined.

The above findings suggest that substituted 5‐nitro indole compounds (**5** and **7**) could improve the antiproliferative activity due to generation of reactive oxygen species along with *c‐Myc* stabilization in cancerous cells. However, we cannot rule out that these antiproliferative activities against the HeLa cells could be due to several other multiple targets. Interestingly, most promising compounds **5** and **7** did not significantly inhibit the growth of normal kidney epithelial cells (NKE) even at 5, 10 and 30 μM, respectively (Figure S13). We further analysed the functional effects of the substituted 5‐nitroindole compounds **5**, **7**, **12**, **3** and **9** **a** on the transcription and translation of the *c‐Myc* gene. Immuno blot results showed that the compounds **5** and **12** reduced the expression of c‐Myc protein levels in HeLa cells relative to untreated cells, by approximately 30 and 50 % when treated with 3 and 10 μM, respectively, and similarly for compound **7**, by 43 % at 3 μM and 65 % at10 μM. The expression of control house‐keeping gene, GAPDH was not affected by the tested ligands (Figure S10). Furthermore, at the transcriptional level by qRT‐PCR, compound **5** showed a more prominent decrease in the expression of *c‐Myc* mRNA at 10 μM in comparison to compound **7** and **12** (Figure 4b). Interestingly, the effect of the compounds on c‐MYC expression is more apparent on the mRNA level than on the protein level. We suspect that the effect appears earlier on the mRNA level than on the protein level.

To investigate the effect of ligands (**5**, **7** and **12**) on the cell cycle in HeLa cells, we performed Hoechst‐staining‐mediated cell cycle analysis (Figure 4c and Tables S3 and S4). Upon exposure to fragments **5** and **7** at 6 μM concentration for 24 h, cell cycle arrest was observed with fewer replicating cells undergoing DNA replication (S/G2/M phase), and the effect is not clear for compound **12**. After 48 h of treatment, HeLa cells showed prominent cell cycle arrest and cell death (sub‐G1) with significant increase in the sub G1 population to 70.9 and 85.7 %, respectively when treated with fragments **5** and **7**, and these effects were not observed for cells treated with compound **12**.

## Discussion and Conclusion

**Figure 5:**
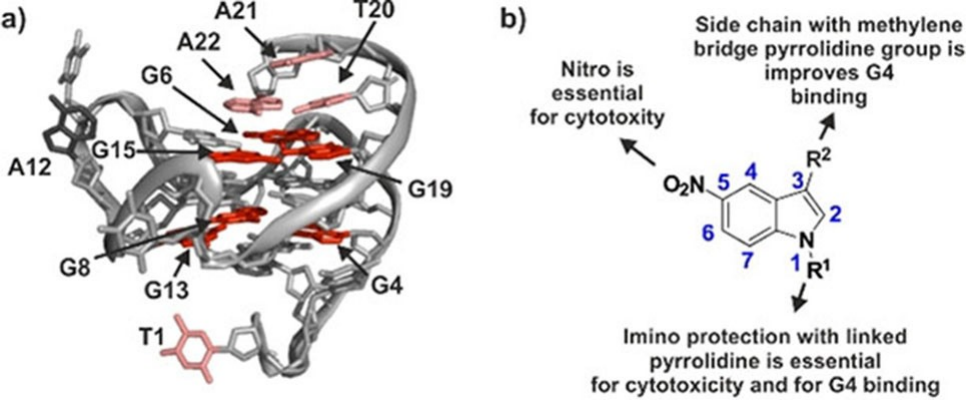
a) Binding of compound **7** by NMR mapped onto the structure of free *c‐Myc* (PDB ID: 1XAV). Shifting imino resonances of guanines in the tetrads are coloured in red, assigned loop and tail resonances are indicated in black (not shifting) and light red (shifting). b) SAR found in this investigation.

In our investigations, we set out to identify new binders to target the *c‐Myc* promoter G‐quadruplex starting from a previously identified compounds *via* high throughput FID screening of commercially obtained compounds. We identified the 5‐nitro indole core as a lead structure and created a library of new scaffolds based on fragment expansion to improve the total polar surface area of the novel compounds exploiting the chemical nature of methylene bridged pyrrolidine‐substituted 5‐nitroindole conjugates, which may aid in the electrostatic binding and contributes to hydrogen binding interactions of the fragments to G4 base pairs. Several synthetic strategies were reported to optimize and generate best hits starting from indoles, 5‐nitroindoles and methylmethanamine derivatives. After screening all compounds applying an FID assay, several biophysical investigations were performed to evaluate the structure‐activity relationships of the 13 best fragments (Figure 5b). We found that either an amino or a nitro‐group at the fifth position of the central indole core is critical for binding, while protection of the N‐indole at the first position plays a significant role in improved G4 binding. In addition to above inclusion of a methylene bridged pyrrolidine side chain at the third position of the indole core is crucial for G4 binding and with *K*_D_/DC_50_ values in the same magnitudes, large differences in the binding are seen by investigations using NMR‐spectroscopy.

In biological investigations, significant differences were observed in between the compounds selected from the high throughput screening. Conjugates **5** and **7** with the inclusion of the imino linked pyrrolidine side chain at the third position and N‐indole protections with methylene bridged pyrrolidines have better IC_50_ values and cell arrest potential. In comparisons, substituted 5‐amino indoles along with the unprotected N‐indole 5‐nitro compound **12** with imino linked pyrrolidine side chain at the third position is found to be ineffective against the cancerous cells. In addition, compound **5** and **7** exhibited improved *c‐Myc* binding compared to that of the previously reported parent compound (**3**). Furthermore, *in vitro* biological evaluations we found that *c‐Myc* mRNA and protein level is significantly downregulated by the treatment of 5‐nitro indole compounds (**5**, **7** and **12**). *In vitro* cellular studies with FACS analysis revealed that upon treatment with compounds **5** and **7** at 6 μM prominent sub‐G1/G1 phase arrest was observed in the cell cycle. One explanation for the relative supremacy of the 5‐nitro indole compounds compared to the 5‐amino indole compounds in the cell assays could be the smaller long‐term stability of the substituted amino‐indoles. Based on our investigations, we could conclude that these antiproliferative effects against cancerous cells might be due to the c‐Myc downregulation at both transcription and translation level along with the generation of reactive oxygen species by nitro group of the most active compounds.

## Experimental Section

**Chemistry**: All solvents and reagents were purified by standard techniques or used as supplied from commercial sources (Sigma‐Aldrich, unless stated otherwise). All reactions were generally carried out under inert atmosphere unless otherwise noted. TLC was performed on Kieselgel 60 F254 plates, and spots were visualized under UV light. Products were purified by flash chromatography on silica gel (100–200 mesh). ^1^H NMR spectra were recorded on 600 MHz instruments at 298 K. ^13^C NMR spectra were recorded at 151 MHz with proton decoupling. Chemical shifts are reported in parts per million (ppm) and are referred to the residual solvent peak. The following notations are used: singlet (s); doublet (d); triplet (t); quartet (q); multiplet (m); broad (br). Coupling constants are quoted in Hertz and are denoted as *J*. Mass spectra were recorded on a Micromass Q‐Tof (ESI) spectrometer. Purity of the key target compounds were detected by HPLC system [Waters e2695 (alliance), Column: GRACE‐C18 (250 mm×4.6 mm×5 mm), Mobile phase: 1 % TFA buffer and ACN and methanol, Flow rate: 1 mL/min] consisting of low‐pressure gradient pump plus auto sampler and Photo Diode Array (PDA) detector. The output signal was monitored and processed using Empower 2 software.

**Declaration of purity**: All final compounds were equal to or more than 95 % pure by HPLC (for key target compounds) and ^1^H NMR (600 MHz and 400 MHz) for the synthesized compounds.

**Generalized procedure for the reduction of nitro to amine (3, 5** **a, 9** **a and 11** **a)**: a solution of a nitro conjugates (2, 5, 9 and 11) in 10 mL of ethanol was dropped into a suspension of 10 % Pd/C (12.5 mg) saturated with H_2_ in ethanol (20 mL). The mixture was stirred at room temperature for 3 h, according to TLC analysis (MeOH/DCM 1 : 3). The catalyst was recovered and the filtrate was evaporated under reduced pressure to dryness to give the corresponding amino‐indoles.

**Generalized procedure for intermediates 6, 8, 15, 2** **a and 20 with Vilsmeier‐Haack reaction (A)**: POCl_3_ (1.2 mL, 9.25 mmol, 1.5 equiv.) as reaction solvent was added in dropwise manner to the dried DMF (10 mL) under inert condition at 0 °C. This mixture was allowed to stir at room temperature for 1 hour followed with the addition of reactant (1 equiv.) **1**, **5**, **14**, **19** **a**, and **1** **a**. The reaction mixture was then allowed to react for 1 hour by stirring at room temperature. The reaction mixture was then quenched with equal amount of ice water and 50 % NaOH was added until a pH of 9.

**Generalized procedure for the condensation of substituted carboxaldehyde and corresponding amines (B) to generate (7, 9, 10, 11, 12, 13, 16, 16** **a, 21** **a, 21** **b, 3** **a, 3** **b, 23** **a, b, e, 24** **a**–**c, e and 25** **b, d, e)**: Substituted carboxaldehydes **6**, **8**, **15**, **2** **a**, **20**, **23**, **24** and **25** (1 equiv.) and substituted amines (3 equiv.) were dissolved in 50 mL of dry EtOH: CH_3_CN (1 : 1). The resulting mixture stirred for 2 h at room temperature and then the solvent was concentrated under reduced pressure. The resultant residue was dissolved in 20 mL EtOH and then NaBH_4_ (10 equiv.) was added to it in portion wise. The reaction mixture is allowed to stir for 24 h at room temperature, and then the excess of NaBH_4_ was filtered off and the solvent was evaporated to dryness. The resultant solid was treated with deionized water and extracted with CH_2_Cl_2_ (3×50 mL). The organic phase was evaporated under reduced pressure.

**Generalized procedure for making HCl salt (C)**: The solid was dissolved in EtOH/dioxane (1 : 3) and precipitated with aqueous HCl 37 % to obtain its hydrochloride salt. The precipitate was filtered and recrystallized with MeOH to give the desired product.

**Generalized procedure for nucleophilic substitution of alkynes (19** **a and 19** **b)**: 5‐Nitro‐1*H*‐indole (2.01 g, 12.3 mmol) has added to a solution of anhydrous KOH (0.692 g, 12.3 mmol) in DMF (100 mL) at room temperature for 30 min. followed with the addition of 3‐bromoprop‐1‐yne and 4‐bromobut‐1‐yne (4 equiv.) and reaction mixture was allowed to stir overnight.

**1‐Methyl‐5‐amino‐1*H*‐indole (3)**: A solution of a 1‐methyl‐5‐nitro‐1*H*‐indole (2) (5.87 mmol) is treated according to protocol A. Yield 96 %; brown solid. *R*_f_=0.44 (eluent MeOH/DCM 1 : 3) 0.44; Mp 87 °C; ^1^H NMR (600 MHz, [D_6_]DMSO): *δ*=7.09 (dd, *J*=9.2, 5.8 Hz, 2H), 6.68 (s, 1H), 6.54 (d, *J*=4.5 Hz, 1H), 6.10 (d, *J*=2.9 Hz, 1H), 4.45 (bs, 2H), 3.66 (s, 3H) ppm; ^13^C NMR (151 MHz, [D_6_]DMSO): *δ*=211.30, 141.55, 130.56, 129.22, 111.82, 109.77, 103.59, 98.77, 32.65 ppm; HRMS: *m/z* calcd for C_9_H_10_N_2_ [*M*+H]^+^ 146.19, found 146.19.

**5‐Nitro‐1‐(3‐bromopropyl)‐1*H*‐Indole (4** **a)**: 5‐Nitro‐1*H*‐indole (2.01 g, 12.3 mmol) was added to a solution of anhydrous KOH (0.692 g, 12.3 mmol) in DMF (100 mL) at room temperature for 30 min, followed by the addition of 1,3‐dibromopropane (3.77 mL, 37.0 mmol). The reaction mixture was then allowed to stir overnight. Solvent was removed under reduced pressure, and the crude product was purified on silica as a stationary phase; Yield 46 % as yellow crystals; *R*_f_=0.65 (eluent EA/*n*‐Hex 1 : 4); Mp 102 °C. ^1^H NMR (600 MHz, CDCl_3_): *δ*=8.58 (d, *J*=2.2 Hz, 1H), 8.04 (d, *J*=9.1 Hz, 1H), 7.71 (d, *J*=9.1 Hz, 1H), 7.67 (d, *J*=3.2 Hz, 1H), 6.78 (d, *J*=3.1 Hz, 1H), 4.40 (t, *J*=6.5 Hz, 2H), 3.28–3.35 (m, 2H), 2.33–2.44 (m, 2H) ppm; ^13^C NMR (151 MHz, CDCl_3_): *δ*=141.79, 138.84, 131.22, 127.92, 118.42, 117.52, 109.33, 104.54, 44.53, 32.70, 30.05 ppm; ESI‐MS: *m/z* 282.0 [*M*+H]; HRMS: *m/z* calcd for C_11_H_11_BrN_2_O_2_ [*M*+H]^+^ 282.0, found 282.0.

**1‐(2‐Bromoethyl)‐5‐nitro‐1*H*‐indole (4** **b)**: 5‐Nitro‐1*H*‐indole (2.01 g, 12.3 mmol) was added to a solution of anhydrous KOH (0.692 g, 12.3 mmol) in DMF (100 mL) at room temperature for 30 min, followed by the addition of 1, 2‐dibromoethane (3.77 mL, 37.0 mmol). The reaction mixture was then allowed to stir overnight. Solvent was removed under reduced pressure, and the crude product was purified on silica as a stationary phase; Yield 46 % as yellow crystals; *R*_f_=0.65 (eluent EA/*n*‐Hex 1 : 4); Mp 112–114 °C. ^1^H NMR (600 MHz, CDCl_3_): *δ*=8.60 (d, *J*=1.9 Hz, 1H), 8.18–8.10 (m, 1H), 7.37 (d, *J*=9.1 Hz, 1H), 7.31 (d, *J*=3.2 Hz, 1H), 6.72 (d, *J*=3.2 Hz, 1H), 4.61–4.57 (t, *J*=6.5 Hz, 2H), 3.68 (t, *J*=6.9 Hz, 2H) ppm; ^13^C NMR (151 MHz, CDCl_3_): *δ*=142.07, 138.72, 131.15, 128.19, 118.50, 117.70, 107.70, 109.06, 104.85, 48.15, 29.73 ppm; ESI‐MS: *m/z* 269.09 [*M*+H]; HRMS: *m/z* calcd for C_10_H_9_BrN_2_O_2_ [*M*+H]^+^ 269.09, found 269.09.

**1,3‐Bis(5‐nitro‐1*H*‐indol‐1‐yl)propane (17** **a)**: Yield 46 % as yellow crystals; *R*_f_=0.85 (eluent EA/n‐Hex 1 : 4); Mp 112–114 °C. ^1^H NMR (600 MHz, CDCl_3_): *δ*=8.50 (t, *J*=5.0 Hz, 2H), 8.09 (dd, *J*=9.1, 2.0 Hz, 2H), 7.51 (d, *J*=3.4 Hz, 2H), 7.43–7.39 (m, 2H), 7.20–7.12 (m, 2H), 6.73 (d, *J*=3.4 Hz, 2H), 5.27 (dd, *J*=15.7, 7.1 Hz, 2H), 4.91 (d, *J*=8.9 Hz, 2H) ppm. ^13^C NMR (151 MHz, CDCl_3_): *δ*=30.05, 32.70, 44.53, 104.54, 109.33, 117.52, 118.42, 127.92, 131.22, 138.84, 141.79 ppm; ESI‐MS: *m/z* 364.35 [*M*+H]^+^; HRMS: *m/z* calcd for C_19_H_16_N_4_O_4_ [*M*+H]^+^ 364.35, found 364.35.

**5‐Nitro‐1‐(3‐(pyrrolidin‐1‐yl) propyl)‐1*H*‐indole (5)**: Purified 5‐nitro‐1‐(3‐bromopropyl)‐1*H*‐indole (**4** **a**; 0.24 g, 0.88 mmol) was dissolved in dry ACN (6 mL/mmol) and to this pyrrolidine (3–10 equiv.) was added, and the mixture was refluxed for 3–4 h. Solvent was concentrated under reduced pressure. The crude product was purified by column chromatography (eluent MeOH/DCM 1 : 3); using silica as a stationary phase; Yield 36 % as yellow viscous solid; *R*_f_ (eluent MeOH/ DCM 1 : 4) 0.35; Mp 112–114 °C. ^1^H NMR (600 MHz, [D_6_]DMSO): *δ*=8.58 (d, *J*=2.2 Hz, 1H), 8.05 (dd, *J*=9.1, 2.3 Hz, 1H), 7.74 (d, *J*=9.1 Hz, 1H), 7.68 (d, *J*=3.1 Hz, 1H), 6.79 (d, *J*=3.2 Hz, 1H), 4.36 (t, *J*=7.0 Hz, 2H), 3.03 (s, 4H), 2.92 (s, 2H), 2.08–2.14 (br, 2H), 1.85 (bs, 4H) ppm; ^13^C NMR (151 MHz, [D_6_]DMSO): *δ*=140.79, 138.63, 132.38, 127.37, 117.58, 116.40, 110.34, 103.91, 53.31, 51.60, 43.43, 26.93, 22.71 ppm; ESI‐MS: *m/z* 273.33 [*M*+H]^+^; HRMS: *m/z* calcd for C_15_H_19_N_3_O_2_ [*M*+H]^+^ 273.15, found 273.15.

**5‐Nitro‐1‐(3‐(pyrrolidin‐1‐yl) propyl)‐1*H*‐indole‐3‐carbaldehyde** (**6**): The crude product is obtained from 5‐nitro‐1‐(3‐(pyrrolidin‐1‐yl) propyl)‐1H‐indole (5) by Vilsmeier‐Haack general protocol. Further purified on silica as a stationary phase with 1 : 4 MeOH/DCM. Yield 56 % as yellow solid; *R*_f_=0.25 (eluent MeOH/DCM 1 : 4); Mp 132–134 °C. ^1^H NMR (600 MHz, [D_6_]DMSO): *δ*=9.99 (s, 1H), 8.93 (s, 1H), 8.60 (s, 1H), 8.18 (d, *J*=9.0 Hz, 1H), 7.88 (d, *J*=9.1 Hz, 1H), 4.41 (t, *J*=6.7 Hz, 2H), 3.40 (s, 4H), 2.37–2.40 (br, 2H), 1.98–2.00 (br, 2H), 1.67 (br, 4H) ppm; ^13^C NMR (151 MHz, [D_6_]DMSO): *δ*=185.08, 143.87, 143.40, 140.06, 123.87, 119.06–118.89, 118.35, 117.11, 111.96, 53.28, 51.95, 44.88, 28.32, 23.08 ppm; HRMS: *m/z* calcd for C_16_H_19_N_3_O_3_ [*M*+H]^+^ 301.14, found 301.14.

***N*** **‐((5‐Nitro‐1‐(3‐(pyrrolidin‐1‐yl)propyl)‐1*H*‐indol‐3‐yl)methyl)‐2‐(pyrrolidin‐1‐yl)ethanamine (7)**: In a round bottom flask, 8‐carboxaldehyde‐5‐nitro‐1‐[3‐(1‐pyrrolidinyl)propyl]‐1*H*‐Indole (6) (6 g, 0.0199 mol) and 2‐(pyrrolidin‐1‐yl)ethanamine (4.54 g, 0.039 mol) were reacted according to protocol B. The product was purified on silica as a stationary phase with 1 : 4 MeOH/DCM. Yield 46 % as yellow viscous solid; *R*_f_ (eluent MeOH/DCM 1 : 4) 0.25; ^1^H NMR (600 MHz, [D_6_]DMSO): *δ*=8.63 (d, *J*=2.3 Hz, 1H), 8.03 (dt, *J*=9.1, 2.1 Hz, 1H), 7.67–7.63 (m, 1H), 7.53 (s, 1H), 4.31–4.23 (m, 2H), 3.92 (bs, 1H), 2.66 (dd, *J*=13.0, 6.4 Hz, 2H), 2.35 (dd, *J*=18.3, 2.7 Hz, 9H), 2.31–2.23 (m, 3H), 1.96–1.87 (m, 2H), 1.72–1.65 (m, 6H), 1.66–1.56 (m, 4H) ppm; ^13^C NMR (151 MHz, [D_6_]DMSO): *δ*=185.08, 143.87, 143.40, 140.06, 123.87, 119.06–118.89, 118.35, 117.11, 111.96, 53.28, 51.95, 44.88, 28.32, 23.08 ppm. HRMS: *m/z* calcd for C_22_H_33_N_5_O_2_ [*M*+Na]^+^ 422.25.

**5‐Nitroindole‐3‐carboxaldehyde (8)**: The crude product was obtained from 5‐nitroindole (**1**) by the Vilsmeier‐Haack general protocol. The product was purified on silica as a stationary phase; with 1 : 4 MeOH/DCM. Yield 85 % as yellow amorphous solid; *R*_f_=0.55 (eluent MeOH/DCM 1 : 4) 0.55; M.p: 109–111 °C; ^1^H NMR(600 MHz, CDCl_3_): *δ*=9.64 (s, 1H), 8.69 (d, *J*=7.8 Hz, 1H), 7.73 (m, 2H), 7.18 (d, *J*=6 Hz, 1H), 2.50–2.62 (br, 1H) ppm; ^13^C NMR (151 MHz, CDCl_3_): *δ*=183.97, 138.90, 118.13, 117.38, 111.91 ppm; HRMS (ESI): *m/z* calcd for C_9_H_6_N_2_O_3_: 190.04, found: 191.09.

***N***,***N*** **‐Dimethyl‐1‐(5‐nitro‐1*H*‐indol‐3‐yl)methanamine (9)**: 5‐Nitroindole‐3‐carboxaldehyde (8) (1 equiv.) and 3 equivalents of dimethylamine (40 % aqueous, 60 mL) allowed to react according to the protocol B. The crude product is obtained as yellow crystalline solid and further purified on silica as a stationary phase with DCM/MeOH (9 : 1) over silica gel column. Yield 60 % as yellow solid; *R*_f_=0.45 (eluent MeOH/DCM 1 : 4) 0.45; Mp 114–116 °C; ^1^H NMR (600 MHz, [D_6_]DMSO): *δ*=11.71 (s, 1H), 8.62 (d, *J*=5.9 Hz, 1H), 8.00 (dt, *J*=11.6, 4.2 Hz, 2H), 7.56–7.51 (m, 1H), 3.68 (s, 2H), 2.22 (s, 6H) ppm. ^13^C NMR (151 MHz, DMSO‐*d*_6_): *δ*=140.09, 139.47, 132.50, 128.31, 126.81, 116.34, 111.59, 69.59, 54.07, 44.65 ppm; HRMS (ESI): *m/z* calcd for C_11_H_13_N_3_O_2_: 219.10, found: 219.10.

***N***,***N*** **‐Dimethyl‐1‐(5‐amino‐1*H*‐indol‐3‐yl)methanamine (9** **a)**: Compound 9 was treated with protocol A. The crude product was purified with DCM/MeOH (9 : 1) over silica gel column. (**10** **a**) Yield 96 %; Brown solid. *R*_f_=0.41(eluent MeOH/DCM 1 : 3); Mp 87–89 °C; ^1^H NMR (600 MHz, [D_6_]DMSO): *δ*=10.38 (s, 1H), 7.04–6.99 (m, 2H), 6.76 (s, 1H), 6.47 (dd, *J*=8.6, 4.9 Hz, 1H), 3.31–3.32 (b, 2H) 4.41 (s, 2H), 2.12 (s, 6H) ppm;^13^C NMR (151 MHz, [D_6_]DMSO): *δ*=211.06, 140.62, 129.95, 128.50, 124.07, 111.63, 111.20, 110.40, 102.17, 54.50, 44.83 ppm; HRMS (ESI): *m/z* calcd for C_11_H_15_N_3_: 189.13 found: 189.13.

***N*** **1,*N*1‐Dimethyl‐N2‐((5‐nitro‐1*H*‐indol‐3‐yl)methyl)ethane‐1,2‐diamine (10)**: 5‐Nitroindole‐3‐carboxaldehyde (**8**; 6 g, 62.5 mmol) and *N*1,*N*1‐dimethylethane‐1,2‐diamine (40 % aqueous, 60 mL) were allowed to react according to the protocol B. Yield 36 %, yellow solid; *R*_f_=0.23 (Eluent MeOH/DCM 1 : 3); Mp 162–164 °C; ^1^H NMR:(600 MHz, CDCl_3_): *δ*=11.68 (s, 1H), 8.53 (dd, *J*=8.6, 3.6 Hz, 1H), 8.02–7.92 (m, 1H), 7.57–7.45 (m, 2H), 3.77 (s, 2H), 3.46 (bs, 1H), 2.54 (t, *J*=4.6 Hz, 2H), 2.46 (t, *J*=6.3 Hz, 2H), 2.06 (s, 6H) ppm; ^13^C NMR: (151 MHz, CDCl_3_): *δ*=140.27, 139.42, 128.91, 126.30, 116.41, 116.42, 116.10, 112.11, 51.00, 46.25, 45.35, 43.05, 11.35 ppm; HRMS (ESI): *m/z* calcd for C_13_H_18_N_4_O_2_: 262.14, found: [*M*+H]^+^=263.14.

***N*** **1,*N*1‐Diethyl‐N2‐((5‐nitro‐1*H*‐indol‐3‐yl)methyl)ethane‐1,2‐diamine (11)**: 5‐Nitroindole‐3‐carboxaldehyde (**8**) (6 g, 62.5 mmol) and *N*1,*N*1‐diethylethane‐1,2‐diamine (40 % aqueous, 60 mL) were allowed to react according to the protocol B. Yield 36 %, yellow solid; *R*_f_=0.23 (eluent MeOH/DCM 1 : 3) 0.23; Mp 152–154 °C; ^1^H NMR(600 MHz, [D_6_]DMSO): *δ*=11.84 (s, 1H), 8.67 (s, 1H), 7.97 (d, *J*=8.0 Hz, 1H), 7.70 (s, 1H), 7.62–7.47 (m, 1H), 4.09 (s, 2H), 3.78 (s, 1H), 2.73 (m, 2H), 2.57 (s, 2H), 2.41 (t, *J*=17.3 Hz, 4H), 1.01–0.82 (m, 6H) ppm. ^13^C NMR (151 MHz, [D_6_]DMSO): *δ*=140.12, 139.57, 127.96, 126.76, 116.94, 116.83, 116.38, 115.11, 111.58, 57.01, 50.58, 49.05, 44.99 ppm; HRMS (ESI): *m/z* calcd for C_15_H_22_N_4_O_2_: 290.17, found=290.17.

***N*** **‐((5‐Nitro‐1*H*‐indol‐3‐yl)methyl)‐2‐(pyrrolidin‐1‐yl)ethanamine (12)**: 5‐Nitroindole‐3‐carboxaldehyde (**8**; 6 g, 62.5 mmol) and 2‐(pyrrolidin‐1‐yl)ethanamine (40 % aqueous, 60 mL) were allowed to react according to the protocol B. Yield 26 %, yellow solid; *R*_f_=0.13 (eluent MeOH/DCM 1 : 3) 0.13; Mp 162–164 °C. ^1^H NMR (600 MHz, CDCl_3_): *δ*=11.70 (bs, 1H), 8.67 (t, *J*=9.1 Hz, 1H), 8.05–7.97 (m, 1H), 7.58–7.53 (m, 2H), 4.01 (s, 2H), 3.48 (bs, 1H), 2.73 (t, *J*=6.4 Hz, 2H), 2.58 (t, *J*=6.4 Hz, 2H), 2.49–2.40 (m, 4H), 1.71–1.62 (m, 4H) ppm; ^13^C NMR (151 MHz, CDCl_3_): *δ*=140.11, 139.61, 127.82, 126.18, 116.83, 116.19, 115.60, 111.86, 54.35, 53.43, 46.61, 43.35, 26.46, 22.85 ppm; HRMS (ESI): *m/z* calcd for C_15_H_20_N_4_O_2_: 288.16, found=288.16.

***N*** **1‐(2‐Aminoethyl)‐*N*2‐(2‐((2‐(((5‐nitro‐1*H*‐indol‐3‐yl)methyl)amino)ethyl)amino)ethyl)ethane‐1,2‐diamine (13)**: Yield 46 %, Yellow solid.^1^H NMR (600 MHz, D_2_O): *δ*=8.59 (s, 1H), 8.00 (d, *J*=9.2 Hz, 1H), 7.67 (d, *J*=3.8 Hz, 1H), 7.48 (d, *J*=9.1 Hz, 1H), 4.58–4.40 (m, 2H), 3.68 (s, 2H), 3.62–2.73 (m, 19H), 1.10 (t, *J*=7.1 Hz, 2H) ppm. ^13^C NMR (151 MHz, D_2_O): *δ*=141.10, 131.11, 125.63, 117.90, 115.39, 112.0, 65.0, 50.77, 43.0, 43.06, 42.34, 35.27, 16.84 ppm; HRMS (ESI): calcd for C_17_H_29_N_7_O_2_ 363.24; found 363.24.

**1‐(3‐(Pyrrolidin‐1‐yl)propyl)‐1*H*‐indol‐5‐amine (5** **a)**: 5‐nitro‐1‐(3‐(pyrrolidin‐1‐yl)propyl)‐1*H*‐indole (5) was treated as per the Generalized procedure for the reduction of nitro to amine; The crude product is brown in colour and highly unstable in nature so we could not purify it further; Yield 23 % as yellow viscous solid; *R*_f_=0.25 (eluent MeOH/DCM 1 : 4) 0.25; ^1^H NMR (600 MHz, [D_6_]DMSO): *δ*=7.11–7.09 (m, 2H), 6.69 (s, 1H), 6.54–6.56 (d, *J*=7.0 Hz, 1H), 6.09–6.11(d, *J*=7.3 Hz, 1H), 4.46 (br, 2H), 4.34–4.37 (t, *J*=6.5 Hz, 2H), 3.04–3.08 (br, 6H), 2.11–2.14 (m, 2H), 1.86–191 (m, 4H) ppm; ^13^C NMR (151 MHz, [D_6_]DMSO) *δ*140.79, 138.63, 132.38, 127.37, 117.58, 116.40, 110.34, 103.91, 53.31, 51.60, 43.43, 26.93, 141.73, 137.73, 125.53 125.42, 117.85, 115.52, 112.19, 54.16, 54.54, 45.86, 43.37, 26.61, 23.31 ppm; ESI‐MS: *m/z* 244.17 [*M*+H]^+^.

**1‐(3‐Azidopropyl)‐5‐nitro‐1*H*‐indole (14)**: To a stirring mixture of 4 (1 g, 3.225 mmol, 1 equiv.) in 15 mL Dry DMF about 3 equiv. of sodium azide (0.6386 g, 9.667 mmol) were added and allowed to reflux for 3–5 h. Followed by quenching with ice water and extraction with ethyl acetate. Organic extract was then concentrated under reduced pressure, and the crude product was purified on silica as a stationary phase with cyclohexane/ethyl acetate (9 : 1) over silica gel column. Yield 56 %, yellow solid; *R*_f_=(eluent EA/cyclo 1 : 3) 0.63; Mp 92–94 °C. ^1^H NMR (600 MHz, [D_6_]DMSO): *δ*=10.00 (s, 1H), 8.94 (d, *J*=2.1 Hz, 1H), 8.62 (s, 1H), 8.19 (dd, *J*=9.1, 2.3 Hz, 1H), 7.90 (d, *J*=9.1 Hz, 1H), 4.43 (t, *J*=7.0 Hz, 2H), 3.40 (t, *J*=6.6 Hz, 2H), 2.07–2.11 (br, 2H) ppm; ^13^C NMR (151 MHz, [D_6_]DMSO): *δ*=185.13, 146.04–144.33, 143.35, 139.92, 123.97, 118.75, 118.26, 117.17, 111.90, 47.89, 44.23, 28.53 ppm; HRMS (ESI): calcd for C_11_H_11_N_5_O_2_ 245.09; found [*M*+H]^+^ 246.09.

**1‐(3‐Azidopropyl)‐5‐nitro‐1*H*‐indole‐3‐carbaldehyde (15)**: The crude product was further purified with Cyclohexane: Ethyl acetate (9 : 1) over silica gel column. Yield 66 %, yellow solid; *R*_f_=0.31(eluent EA/cyclo 1 : 3); Mp 102–104 °C; ^1^H NMR (600 MHz, [D_6_]DMSO): *δ*=10.00 (s, 1H), 8.94 (d, *J*=2.1 Hz, 1H), 8.62 (s, 1H), 8.19 (dd, *J*=9.1, 2.3 Hz, 1H), 7.90 (d, *J*=9.1 Hz, 1H), 4.43 (t, *J*=7.0 Hz, 2H), 3.40 (t, *J*=6.6 Hz, 2H), 2.07–2.11 (br, 2H) ppm; ^13^C NMR (151 MHz, [D_6_]DMSO): *δ*=185.13, 146.04, 144.33, 143.35, 139.92, 123.97, 118.75, 118.26, 117.17, 111.90, 47.89, 44.23, 28.53 ppm; HRMS (ESI): calcd for C_12_H_11_N_5_O_3_ 273.09; found 273.09.

**1‐(1‐(3‐Azidopropyl)‐5‐nitro‐1*H*‐indol‐3‐yl)‐*N***,***N*** **‐dimethylmethanamine (16)**: Compound **8** (1 g, 3.663 mmol, 1 equiv.) and 3 equiv. of *N*1, *N*1‐dimethylethane‐1,2‐diamine (1.284 g, 11.070 mmol) were allowed to react according to generalized procedure B. Crude product was then further purified with DCM/MeOH (9 : 1) over silica gel column. Yield 26 %, yellow solid; *R*_f_ *=*0.43(eluent MeOH/DCM 1 : 3) 0.43; Mp 102–104 °C. ^1^H NMR (600 MHz, [D_6_]DMSO): *δ*=8.66 (d, *J*=1.8 Hz, 1H), 8.03 (dd, *J*=9.1, 2.1 Hz, 1H), 7.67 (d, *J*=9.1 Hz, 1H), 7.56 (s, 1H), 4.29 (t, *J*=6.9 Hz, 2H), 3.95 (s, 2H), 3.30–3.32 (bs, 4H), 2.64 (t, *J*=6.4 Hz, 2H), 2.44 (q, *J*=7.1 Hz, 2H), 1.99–2.03 (br, 2H) ppm; ^13^C NMR (151 MHz, [D_6_]DMSO): *δ*=140.35, 139.09, 126.55, 116.58, 110.24, 51.79, 47.98, 46.34, 43.59, 43.09, 28.90, 11.61 ppm. HRMS (ESI): calcd for C_14_H_18_N_6_O_2_ 302.15; found 302.15.

***N***,***N*** **‐Dimethyl‐*N*′‐[[5‐nitro‐1‐(3‐Azidopropyl)‐1*H*‐indole]methyl]‐1,2‐Ethanediamine (16** **a)**: Compound **8** (1 g, 3.663 mmol, 1 equiv.) and about 3 equiv. of *N*1,*N*1‐diethylethane‐1,2‐diamine ( 1.284 g, 11.07 mmol) were allowed to react according to generalized procedure B. Crude product was then further purified with DCM/MeOH (9 : 1) over silica gel column. Yield 46 %, yellow solid; *R*_f_=0.33(eluent MeOH/DCM 1 : 3); Mp 162–164 °C; ^1^H NMR (600 MHz, [D_6_]DMSO): *δ*=8.63 (d, *J*=2.3 Hz, 1H), 8.60 (d, *J*=2.3 Hz, 1H), 8.03 (dt, *J*=9.1, 2.1 Hz, 2H), 7.66 (dd, *J*=9.1, 4.7 Hz, 2H), 7.55 (d, *J*=5.6 Hz, 1H), 7.54 (d, *J*=9.6 Hz, 1H), 4.35–4.21 (m, 4H), 3.92 (s, 2H), 2.71–2.60 (m, 3H), 2.35 (dd, *J*=18.3, 2.7 Hz, 2H), 2.24 (dd, *J*=14.1, 7.7 Hz, 2H), 2.02–1.87 (m, 2H), 1.76–1.49 (m, 4H) ppm. ^13^C NMR (151 MHz, [D_6_]DMSO): *δ*=140.11, 139.22, 130.92, 130.39, 126.30, 116.70, 116.47, 116.12, 114.59, 110.20, 85.45, 55.25, 53.42, 53.03, 52.79, 51.84, 47.46, 43.62, 30.81, 28.75, 24.19, 23.06 ppm; HRMS (ESI): calcd for C_18_H_27_N_7_O_2_ 373.22; found 374.22.

**5‐Nitro‐1‐(prop‐2‐yn‐1‐yl)‐1*H*‐indole (19** **a)**: Yield 86 %, yellow solid; *R*_f_=0.46 (eluent EA/cyclo 1 : 3); M.p: 113–115 °C; ^1^H NMR (600 MHz, CDCl_3_): *δ*=8.57 (d, *J*=2.2 Hz, 1H), 8.17–8.11 (m, 1H), 7.43 (d, *J*=9.1 Hz, 1H), 7.37 (d, *J*=3.3 Hz, 1H), 6.71 (d, *J*=3.3 Hz, 1H), 4.93 (d, *J*=2.6 Hz, 2H), 2.48 (t, *J*=2.6 Hz, 1H) ppm; ^13^C NMR (151 MHz, CDCl_3_): *δ*=142.04, 138.04, 130.46, 128.18, 118.25, 117.63, 109.37, 104.64, 76.33, 74.60, 36.27 ppm; HRMS (ESI): calcd for C_11_H_8_N_2_O_2_ 200.06; found 201.06.

**1‐(But‐3‐yn‐1‐yl)‐5‐nitro‐1*H*‐indole (19** **b)**: Yield 88 %, yellow solid; *R*_f_=0.39(eluent EA/cyclo 1 : 3); M.p: 113–115 °C; ^1^H NMR (600 MHz, [D_6_]DMSO): *δ*=8.52 (t, *J*=2.7 Hz, 1H), 8.08–8.03 (m, 1H), 7.32 (d, *J*=9.1 Hz, 1H), 7.25 (t, *J*=2.7 Hz, 1H), 6.62 (d, *J*=3.0 Hz, 1H), 4.28 (q, *J*=6.9 Hz, 2H), 2.65 (tt, *J*=5.4, 2.7 Hz, 2H), 1.98 (dt, *J*=5.3, 2.7 Hz, 1H) ppm; ^13^C NMR (151 MHz, [D_6_]DMSO): *δ*=131.54, 128.56, 118.1, 118.09, 109.41, 105.17, 80.2, 72.14, 46.39, 21.12 ppm; HRMS (ESI): calcd for C_12_H_10_N_2_O_2_ 214.07; found 215.07.

**5‐Nitro‐1‐(prop‐2‐yn‐1‐yl)‐1*H*‐indole‐3‐carbaldehyde (20)**: The crude product was purified on silica as a stationary phase; with 1 : 4 EA/CH. The product was a yellow solid. ^1^H NMR (600 MHz, [D_6_]DMSO): *δ*=10.01 (s, 1H), 8.93 (t, *J*=6.6 Hz, 1H), 8.61 (s, 1H), 8.23 (dd, *J*=9.1, 2.4 Hz, 1H), 7.87 (d, *J*=9.1 Hz, 1H), 5.33 (d, *J*=2.5 Hz, 2H), 3.61 (t, *J*=2.5 Hz, 1H) ppm; ^13^C NMR (151 MHz, [D_6_]DMSO): *δ*=185.49, 143.43, 142.92, 139.37, 123.94, 118.99, 118.41, 117.24, 112.08, 77.32, 36.41 ppm.

***N***,***N*** **‐Dimethyl‐1‐(5‐nitro‐1‐(prop‐2‐yn‐1‐yl)‐1*H*‐indol‐3‐yl)methanamine (21** **a)**: 5‐nitro‐1‐(prop‐2‐yn‐1‐yl)‐1H‐indole‐3‐carbaldehyde and 3 equivalent of dimethylamine (40 % aqueous, 60 mL) were allowed to react according to the protocol B. ^1^H NMR (600 MHz, [D_6_]DMSO): *δ*=8.61–8.59 (d, *J*=7.1 Hz, 1H), 8.11 (dd, *J*=9.1, 2.2 Hz, 1H), 7.72 (s, 1H), 7.75 (d, *J*=9.1 Hz, 1H), 5.17 (d, *J*=2.4 Hz, 2H), 4.29 (s, 2H), 3.61 (bs, 1H), 2.17–2.18 (m, 6H) ppm; ^13^C NMR (151 MHz, [D_6_]DMSO): *δ*=141.08, 139.16, 131.14, 127.62, 117.15, 116.99, 116.92, 115.37, 110.96, 78.78, 76.28, 53.96, 44.96, 35.93 ppm; HRMS (ESI): calcd for C_14_H_15_N_3_O_2_ 257.12; found 258.12.

***N*** **1,*N*1‐Diethyl‐N2‐((5‐nitro‐1‐(prop‐2‐yn‐1‐yl)‐1*H*‐indol‐3‐yl)methyl)ethane‐1,2‐diamine (21** **b)**: 5‐nitro‐1‐(prop‐2‐yn‐1‐yl)‐1H‐indole‐3‐carbaldehyde and *N*1,*N*1‐diethylethane‐1,2‐diamine (40 % aqueous, 60 mL) were allowed to react according to the protocol B. ^1^H NMR (400 MHz, [D_6_]DMSO): *δ*=8.83 (d, *J*=7.1 Hz, 1H), 8.13 (dd, *J*=9.1, 2.2 Hz, 1H), 7.82 (s, 1H), 7.75 (d, *J*=9.1 Hz, 1H), 5.25 (d, *J*=6.4 Hz, 2H), 4.29 (s, 2H), 3.61 (bs, 2H), 3.01–2.93 (m, 2H), 2.84–2.74 (m, 2H), 2.67 (q, *J*=7.1 Hz, 4H), 1.05–0.96 (m, 6H) ppm; ^13^C NMR (151 MHz, [D_6_]DMSO): *δ*=140.81, 138.29, 132.66, 126.62, 117.03, 116.61, 110.87, 110.69, 78.7, 76.22, 48.79, 46.14, 43.39, 41.20, 35.69, 10.11 ppm. HRMS (ESI): calcd for C_18_H_24_N_4_O_2_328.19; found 329.19.

**1*H*‐Indole‐3‐carbaldehyde (2** **a)**: The crude product was obtained from 1*H*‐indole (5) by Vilsmeier‐Haack general protocol A. Crude product was purified with DCM/MeOH (9 : 1) over silica gel column. Yield 66 %, White crystalline solid; *R*_f_ (eluent MeOH/DCM 1 : 3) 0.33; M.p: 123–125 °C; ^1^H NMR (600 MHz, CDCl_3_): *δ*=9.60 (s, 1H), 7.83 (d, *J*=6 Hz, 2H), 7.53 (d, *J*=6 Hz, 1H), 7.10 (d, *J*=6 Hz, 1H), 6.89–6.84 (m, 2H) ppm; ^13^C NMR (151 MHz, CDCl_3_): *δ*=184.11, 136.63, 122.92, 121.57, 120.63, 111.50 ppm. (ESI): *m/z* calcd for C_9_H_7_NO:145.10, found: 146.02.

**1‐(1*H*‐Indol‐3‐yl)‐*N***, ***N*** **‐dimethylmethanamine (3** **a)**: Yield 46 %, yellow solid; *R*_f_=0.43(eluent MeOH/DCM 1 : 3) 0.43; M.p: 143–145 °C. ^1^H NMR (600 MHz, [D_6_]DMSO): *δ*=10.92 (s, 1H), 7.61 (d, *J*=7.9 Hz, 1H), 7.36 (d, *J*=8.1 Hz, 1H), 7.21 (t, *J*=5.1 Hz, 1H), 7.08 (t, *J*=7.5 Hz, 1H), 6.98 (t, *J*=7.4 Hz, 1H), 3.55 (s, 2H), 2.17 (s, 6H) ppm; ^13^C NMR (151 MHz, [D_6_]DMSO): *δ*=136.32, 127.52, 124.38, 120.85, 118.98, 118.29, 111.50, 111.24, 54.36, 44.82 ppm. HRMS: *m/z* calcd for C_11_H_14_N_2_ [*M*+H]^+^ 174.12, found 174.12.

***N*** **1‐((1*H*‐Indol‐3‐yl)methyl)‐*N*2‐(2‐((2‐((2‐aminoethyl)amino)ethyl)amino)ethyl)ethane‐1,2‐diamine (3** **b)**: Yield 46 %, white solid; ^1^H NMR (600 MHz, D_2_O): *δ*=10.62 (s, 1H), 7.78 (d, *J*=7.88 Hz, 1H), 7.61 (d, *J*=2.48 Hz, 1H), 7.58 (s, 1H), 7.33 (t, *J*=7.34 Hz, 1H), 7.26 (t, *J*=7.38 Hz, 1H), 4.57 (s, 2H), 3.39–3.48 (m, 22H) ppm; ^13^C NMR (151 MHz, D_2_O): *δ*=42.20, 42.93, 43.77, 43.92, 44.24, 103.58, 112.32, 118.01, 120.29, 122.62, 126.13, 128.20, 136.19 ppm; HRMS (ESI): calcd for C_17_H_30_N_6_ 318.26; found [*M*+H]^+^ 319.26.

**1‐(1*H*‐Indazol‐3‐yl)‐*N***,***N*** **‐dimethylmethanamine (23** **a)**: Yield 36 %, white solid; *R*_f_=0.51(eluent MeOH/DCM 1 : 3) 0.51; M.p: 143–145 °C; ^1^H NMR (600 MHz, [D_6_]DMSO): *δ*=12.93 (d, *J*=12.7 Hz, 1H), 8.00 (s, 1H), 7.59 (s, 1H), 7.47 (d, *J*=8.6 Hz, 1H), 7.29 (d, *J*=8.6 Hz, 1H), 3.44 (s, 2H), 2.12 (s, 6H) ppm; ^13^C NMR (151 MHz, [D_6_]DMSO): *δ*=211.08, 133.18, 130.77, 127.53, 122.53, 119.87, 109.83, 63.28, 44.66 ppm; HRMS (ESI): calcd for C_10_H_13_N_3_ 175.11; found [*M*+H]^+^ 176.11.

***N*** **1‐((1*H*‐Indazol‐3‐yl)methyl)‐*N*2‐(2‐((2‐((2‐aminoethyl)amino)ethyl)amino)ethyl)ethane‐1,2‐diamine (23** **b)**: Yield 66 %, white solid; ^1^H NMR (600 MHz, D_2_O): *δ*=8.14 (d, *J*=6.0 Hz, 1H), 7.92 (s, 1H), 7.65 (t, *J*=7.6 Hz, 1H), 7.46 (d, *J*=7.8 Hz, 1H), 4.39 (s, 2H), 3.56–2.68 (m, 23H) ppm; ^13^C NMR (151 MHz, D_2_O): *δ*=140.22, 134.64, 128.54, 123.62, 122.79, 51.97, 43.52, 42.90, 42.30, 42.24 ppm. HRMS (ESI): calcd for C_16_H_29_N_7_ 319.25; found [*M*+H]^+^ 320.25.

***N*** **‐((1*H*‐Indazol‐3‐yl)methyl)‐2‐(pyrrolidin‐1‐yl)ethanamine (23** **e)**: Yield 56 %, white solid; *R*_f_=0.27(eluent MeOH/DCM 1 : 3); M.p: 173–175 °C. ^1^H NMR (600 MHz, [D_6_]DMSO): *δ*=7.92 (s, 1H), 7.56 (s, 1H), 7.39 (d, *J*=8.6 Hz, 1H), 7.25 (d, *J*=8.6 Hz, 1H), 3.70 (d, *J*=5.7 Hz, 2H), 2.50 (s, 2H), 2.48–2.38 (m, 2H), 2.37–2.22 (m, 2H), 1.90 (d, *J*=5.8 Hz, 4H), 1.65–1.48 (m, 4H) ppm; ^13^C NMR (151 MHz, [D_6_]DMSO): *δ*=132.60, 126.55, 118.69, 109.46, 55.33, 55.39, 53.70, 53.25, 53.16, 47.53, 23.07 ppm. HRMS (ESI): calcd for C_14_H_20_N_4_ 244.17; found [*M*+H]^+^ 245.17.

***N***,***N*** **‐Dimethyl‐1‐(1*H*‐pyrrolo[2,3‐b]pyridin‐3‐yl)methanamine (24** **a)**: Yield 56 %, white solid; *R*_f_=0.13 (eluent MeOH/DCM 1 : 3); M.p: 143–145 °C. ^1^H NMR (600 MHz, [D_6_]DMSO): *δ*=11.39 (bs, 1H), 8.22–8.17 (m, 1H), 8.00 (dd, *J*=6.3, 5.2 Hz, 1H), 7.34 (s, 1H), 7.06–7.02 (m, 1H), 3.55 (s, 2H), 2.16 (d, *J*=4.3 Hz, 6H) ppm;^13^C NMR (151 MHz, [D_6_]DMSO): *δ*=185.17, 148.80, 145.17, 142.64, 127.37, 124.83, 119.78, 118.43, 115.43, 114.72, 110.20, 54.14, 44.33; HRMS (ESI): calcd for C_10_H_13_N_3_ 175.11; found [*M*+H]^+^ 176.11.

***N*** **1‐((1*H*‐Pyrrolo[2,3‐b]pyridin‐3‐yl)methyl)‐*N*2‐(2‐((2‐((2‐aminoethyl)amino)ethyl)amino)ethyl)ethane‐1,2‐diamine (24** **b)**: Yield 36 %, white solid; ^1^H NMR (600 MHz, D_2_O): *δ*=8.74 (dd, *J*=8.0, 7.8 Hz, 1H), 8.41 (m, 1H), 8.02–7.78 (m, 1H), 7.58 (t, *J*=6.7 Hz, 1H), 4.56 (d, *J*=9.3 Hz, 2H), 3.76–2.72 (m, 23H) ppm; ^13^C NMR (151 MHz, D_2_O): *δ*=138.22, 137.01, 133.41, 131.41, 131.98, 124.50, 116.58, 105.39, 52.57, 51.88, 50.33, 48.99, 44.65, 43.65, 43.46; HRMS (ESI): calcd for C_16_H_29_N_7_ 319.25; found [*M*+H]^+^ 320.26.

***N*** **1‐((1*H*‐Pyrrolo[2,3‐b]pyridin‐3‐yl)methyl)‐*N*2‐(2‐((2‐aminoethyl)amino)ethyl)ethane‐1,2‐diamine (24** **c)**: Yield 36 %, white solid; ^1^H NMR (600 MHz, D_2_O): *δ*=8.76 (d, *J*=12.4 Hz, 1H), 8.40 (d, *J*=15.1 Hz, 1H), 7.90 (s, 1H), 7.61 (d, *J*=6.3 Hz, 1H), 4.61 (dd, *J*=12.5 Hz, 13.2 Hz, 6H), 3.63–2.47 (m, 14H) ppm; HRMS (ESI): calcd for C_14_H_24_N_6_ 276.21; found [*M*+H]^+^ 277.21.

***N*** **‐((1*H*‐Pyrrolo[2,3‐b]pyridin‐3‐yl)methyl)‐2‐(pyrrolidin‐1‐yl)ethanamine (24** **e)**: Yield 46 %, white solid; ^1^H NMR (600 MHz, [D_6_]DMSO): *δ*=11.44 (bs, 1H), 8.19 (dd, *J*=6.6, 3.8 Hz, 1H), 8.00 (dd, *J*=8.5, 7.6 Hz, 1H), 7.92–7.85 (m, 1H), 7.41–7.31 (m, 1H), 7.08–6.92 (m, 1H), 3.85 (s, 2H), 3.72 (s, 2H), 2.67–2.61 (m, 2H), 2.46–2.33 (m, 2H), 2.31–2.23 (m, 2H), 1.70–1.53 (m, 4H) ppm; ^13^C NMR (151 MHz, [D_6_]DMSO): *δ*=148.80, 142.41, 127.45, 126.94, 124.88, 123.80, 119.57, 119.17, 114.83, 112.83, 112.87, 111.47, 85.47, 55.30, 53.42, 49.62, 22.71 ppm; HRMS (ESI): calcd for C_14_H_20_N_4_ 244.17; found [*M*+H]^+^ 245.17.

***N*** **1‐(2‐Aminoethyl)‐*N*2‐(2‐((2‐((benzo[d][1,3]dioxol‐5‐ylmethyl)amino)ethyl)amino)ethyl)ethane‐1,2‐diamine (25** **b)**: Yield 46 %, white solid; ^1^H NMR (600 MHz, D_2_O): *δ*=6.91 (dd, *J*=12.5, 6.4 Hz, 4H), 5.96 (s, 2H), 4.25–4.05 (m, 2H), 3.59–2.73 (m, 21H) ppm; ^13^C NMR (151 MHz, D_2_O): *δ*=147.80, 138.45, 133.80, 131.13, 124.69, 116.42, 105.97, 51.84, 44.65, 43.51, 42.46, 41.57, 40.07, 35.20, 18.06 ppm; HRMS (ESI): calcd for C_16_H_29_N_5_O_2_ 323.23; found [*M*+H]^+^ 324.23.

***N*** **1‐(Benzo[d][1,3]dioxol‐5‐ylmethyl)‐*N*2,** ***N*** **2‐dimethylethane‐1,2‐diamine (25** **d)**: Yield 56 %, white solid; *R*_f_ (eluent MeOH/DCM 1 : 3) 0.13; M.p: 103–105 °C. ^1^H NMR (600 MHz, [D_6_]DMSO): *δ*=6.91 (s, 1H), 6.79 (dd, *J*=12.5, 7.9 Hz, 2H), 5.97 (s, 1H), 3.63 (s, 2H), 2.54–2.56 (m, 2H), 2.33 (t, *J*=6.4 Hz, 4H), 2.11 (s, 6H) ppm; ^13^C NMR (151 MHz, [D_6_]DMSO): *δ*=147.23, 145.69, 134.46, 120.98, 108.32, 107.66, 100.51, 58.51, 52.48, 45.85, 45.08 ppm; HRMS (ESI): calcd for C_12_H_18_N_2_O_2_ 222.14; found 222.14.

***N*** **‐(Benzo[d][1,3]dioxol‐5‐ylmethyl)‐2‐(pyrrolidin‐1‐yl)ethanamine (25** **e)**: Yield 46 %, white solid; ^1^H NMR (600 MHz, D_2_O): *δ*=7.05–6.92 (m, 4H), 6.04 (s, 2H), 4.26 (s, 2H), 3.57 (dt, *J*=14.6, 6.6 Hz, 8H), 2.12 (s, 4H) ppm; ^13^C NMR (151 MHz, D_2_O): *δ*=148.02, 147.67, 124.35, 123.55, 117.89, 114.96, 108.80, 109.14, 101.74, 54.83, 51.53, 49.47, 41.94, 22.39; HRMS (ESI): calcd for C_14_H_20_N_2_O_2_ 248.15; found [*M*+H]^+^ 249.15.

***N*** **1‐((5‐Amino‐1*H*‐indol‐3‐yl)methyl)‐*N*2,** ***N*** **2‐diethylethane‐1, 2‐diamine (11** **a)**: Brown (crude); ^1^H NMR (600 MHz, [D_6_]DMSO): *δ*=10.32 (s, 1H), 7.03 (d, *J*=3.8 Hz, 1H), 7.00 (d, *J*=2.3 Hz, 2H), 6.71 (d, *J*=1.9 Hz, 1H), 6.46 (dd, *J*=8.5, 2.0 Hz, 2H), 3.71 (s, 2H), 3.17 (d, *J*=4.9 Hz, 2H), 2.64–2.55 (m, 3H), 2.48–2.39 (m, 4H), 0.95–0.88 (m, 6H); **11** **a** is unstable in nature and hence degrades very quickly, so it is not possible to do further analysis.

Please refer to the Supporting Information for the general procedure and synthetic Scheme 4.

**DNAs**: The *c‐Myc* DNA (TGAGGGTGGGTAGGGTGGGTAA) was purchased by Eurofins, Germany as HPSF (High Purity Salt Free) purified oligos and further purified by HPLC, and stock solutions (100 μM) were made by resuspending the DNA in molecular biology grade water and quantified by A260 at 95 °C, using *ϵ* 260 values as provided by the manufacturers, before the DNAs were aliquoted and stored at −20 °C. All samples were freshly prepared prior to each experiment, the *Myc* DNA was dissolved in the respective buffer heated at 90 °C for 5 min, and slowly cooled down to room temperature. All other reagents were purchased from Sigma‐Aldrich unless otherwise stated.

**Fragment screening with thiazole displacement assays**: The FID assay was performed using the procedure described earlier.^[39]^ Dissociation constant (*K*_D_) for TO binding to *c‐Myc*22‐merhas been determined and reported in (see the Supporting Information and Figure S1) using the following conditions 0.25 μM DNA, 0.5 μM Thiazole Orange, 10 % DMSO, 20 mM Na caco, 140 mM KCl, pH 7 (25 μL/well). All fragment molecules were 95 % pure and obtained from commercial sources or were synthesized in house. For assay optimization sufficient negative and positive controls were used, DMSO only (10 % *v*/*v*) wells, which contained no small molecule, were used as a negative control, while positive control wells consisted target DNA and the intercalator TO.

For screening, 1.25 μL of each fragment from its original 100 mM DMSO stock plate was transferred to a 384 well assay plate (low volume, flat bottom black NBS treated, Corning 3820) with each 384 well plate containing 182 fragments with negative and positive controls. 23.75 μL of the annealed c‐*Myc* oligo containing 0.25 μM DNA, 0.5 μM Thiazole Orange, 20 mM Na cacodylate, 140 mM KCl, pH 7 were added to the ligands and the plate was incubated for 30 min at room temperature. The fluorescent measurements were taken at 25 °C using an excitation filter of 510 nm and an emission filter of 540 nm using an Infinite 200 Pro Micro Plate Reader (Tecan i‐control). Experiments were performed in triplicate and were repeated three times. The ligands were ranked according to their TO displacement effect and those fragments showing≥95 % displacement were subjected to a dose response, under the original screening conditions Titration Scheme of *c‐Myc*: *c*(TO)=0.5 μM; *c*(c‐*Myc*)=0,5 μM; *c*(DMSO)=10 %; c (ligand)=256, 128, 64, 32, 16, 8, 4, 2, 1, 0.5, 0.25, 0.125, and 0.0625 μM; RT; 10 % DMSO, 20 mM Na cacodylate, 140 mM KCl, pH 7.The 50 % displacement value (DC_50_), were calculated for ligands as described in the supplementary.

**Microscale thermophoresis (MST) assay**: The fluorescently labelled oligonucleotides (FAM−Pu22, 5′‐TGAGGGTGGGTAGGGTGGGTAA‐3′ and FAM‐duplex, 5′‐CGCGCGCGTTTTCGCGCGCG‐3′, was diluted from stock to the required concentration (10 μM) in 10 mM Tris‐HCl buffer, pH 7.4, in the presence of 150 mM KCl. Then the mix was annealed by heating at 95 °C for 5 min, gradually cooled to room temperature, and incubated at 4 °C overnight. The concentration of fragments was varied from 0 to 20 μM for G‐Quadruplex sequences and 0 to 200 μM for tested the *K*_D_ of duplex DNA. A 12‐point dilution series was prepared for each DNA. After incubation, the samples were loaded into MST standard‐treated glass capillaries, and MST analysis was performed using a Monolith NT.115 instrument (Nano Temper).

**Fluorescence intensityy titration FAM‐labelled** ***Myc*** **(pu22)**: 5′‐TGAGGGTGGGTAGGGTGGGTAA‐3′ was heated at 95 °C for 3 min, allowed to cool to RT, and diluted to 50 nM in 10 mM Tris ⋅ HCl buffer, pH 7.4, in the presence of 150 mM KCl. Compound was added as a solution both in buffer containing 2–3 % DMSO, and the sample was allowed to equilibrate for 10 min. Fluorescence intensity spectra were recorded at RT using an Infinite 200 Pro Micro Plate Reader (Tecan i‐control). Fluorescence intensity was recorded at an excitation wavelength of 645 nm, with the resulting emission spectrum recorded from 650 to 800 nm, and the fluorescence intensity at the emission maximum was used in all calculations. The concentration of fragments was varied from 0 to 20 μM for G‐Quadruplex DNA.

**NMR**: All NMR measurements were recorded at 600 MHz at 298 K. Samples contained 0.1 mM *c‐Myc* in 25 mM KP_i_, 70 mM KCl, pH 7, 0.025 mM DSS and 10 % [D_6_]DMSO. Due to poor solubility of the fragments in the absence of DNA, a single sample for each DNA: fragment ratio was prepared to keep the DMSO concentration constant. For concentration the titration, the DNA was provided as a 100 μM solution in 25 mM Tris ⋅ HCl buffer (pH 7.4) with 100 mM KCl in 10 % [D_6_]DMSO/90 % H_2_O. Small amounts of the ligand stock solution in 100 % [D_6_]DMSO were added directly into the NMR tube (10 % [D_6_]DMSO at the end of the titration). 2,2‐Dimethyl‐2‐silapentane‐5‐sulfonate (DSS) was used as internal reference. Watergate W5 pulse sequence with gradients (37) was used for water suppression.

**Cells and culture conditions**: HeLa cells (human cervical cancer cells) were grown in Dulbecco's modified Eagle's medium (DMEM, Gibco® Life Technologies) supplemented with 10 % foetal bovine serum (PAN Biotech), and 1 % penicillin/streptomycin (Invitrogen). NKE cells were cultured in RPMI 1640 (GIBCO) with 10 % FBS. HeLa cells were maintained in 25 or 75 cm^2^ flasks in a humidified atmosphere containing 5 % CO_2_, at 37 °C. For cell passaging, biotase (Invitrogen) was used for detaching the cells and the standard Neubauer chamber for counting the cells and seeding accordingly.

**Cell proliferation assay**: HeLa cells were used for performing the cell viability assays. A day before the assay cells were seeded in a 96 well plate (Nunclon 96 Flat Bottom Transparent), with a density of 1000 cells per well in 100 μL. Ligand dilutions were prepared in DMSO, vortexed and stored in −20 °C dilution range varied between 0, 3, 10, 30, 100, 300, 1000, 3000, 9000, 30 000, 100 000 nM depending upon the potency of the respective fragments. The working concentrations were set up in culture medium and added to the cells, to a final DMSO concentration of 0.1 %. Cells treated with the fragments were placed back in the 5 % CO_2_ incubator. After 3 days of fragment treatment cell viability assay was performed using Alamar Blue reagent (Thermo Scientific) following the instructions of the manufacturer. The fluorescence signal of the Alamar Blue was measured with infinite 200 Pro Micro Plate Reader (Tecan i‐control) with the excitation wavelength 540 nm and emission wavelength 590 nm. Ligand treatments were performed in triplicates and were repeated three times. The dose‐dependent cytotoxicity on cells was evaluated in comparison to the DMSO control. To scavenge the ROS produced by substituted 5‐nitroindoles, HeLa cells were pretreated with 2.5 mM NAC for 1 h and then treated with 5‐nitroindoles for 72 h. After compound treatment, Alamar Blue reagent (10 μL, 5 mg/mL) was then added into each well for 4 h incubation. Absorbance were then taken to estimate the cell viability.

The percentage of viable cells was calculated by the following equation:

$$\%\;viable\;cells=\frac{Absorbance\;of\;treated\;cells}{Absorbance\;of\;untreated\;cells}\;\times 100$$

The final IC_50_ values were calculated by using the GraphPad Prism 6.0 software. In the GraphPad Prism® equation, log‐transformed concentration values and the effect data were fitted to a four‐parameter logistic equation. The original, % control, or % inhibition data are represented by *Y* along with their minimal (min) and maximal (max) values. The inhibitor concentration is represented by *X*, IC_50_ is the concentration at 50 % maximal value, and Hill Slope is the slope factor.

$$Y=\min+\frac{\left(\max-\;min\right)}{1+10^{((X-\log IC50)\times Hill\;slope}}\;\times 100$$

**c‐Myc expression and immuno blotting**: for monitoring the expression levels of c‐Myc after ligand treatment, Western blot analysis was performed. Cells treated for 24 h with different fragments in varying concentrations were harvested with biotase, and suspended in PBS buffer containing EDTA free protease inhibitor. Followed by cell lysis with ultrasonicator and quantification of the cell lysate with Roti‐Quant. Equal amounts of the cell lysates were loaded onto the pre‐casted NuPAGE 4–12 % Bis‐Tris Gels and with the MES SDS running buffer (50 mM MES, 50 mM Tris base, 0.1 % SDS, 1 mM EDTA, pH 7.3) gel electrophoresis was performed. Proteins on the gel were transferred onto the PVDF membrane (activated by brief incubation in methanol) for immunoblotting. After the protein transfer, the membrane was blocked for an hour in 5 % non‐fat milk in TBS buffer. The blot was then probed either with c‐Myc rabbit monoclonal antibody (1 : 2 000, #5605, Cell Signaling) in 1X TBS with 5 % BSA, in 1X TBS with 5 % BSA or GAPDH monoclonal antibody in 1X PBS with 5 % non‐fat dried milk and incubated overnight at 4 °C, on a gel rocking platform shaker. To get rid off the unbound primary antibody, the membrane was washed three times with PBST (PBS with 0.05 % Tween 20) for 10 min/wash. Further, the membrane was probed either with horsera dish peroxidase (HRP)‐conjugated affinipure goat anti‐rabbit IgG (H+L) sera in PBS (1 : 5 000, Dianova) or with HRP‐conjugated goat anti‐mouse secondary antibody in PBS (1 : 5 000, Dianova) respectively, for 1 h. Followed by three washing steps with PBS Tween (0.05 %, 10 min/wash). Subsequently, the membrane was developed with PierceTM ECL chemiluminescent HRP substrate, and the chemiluminescence signals were detected using digital Lumi‐imager (Roche). Relative intensities of the ECL signals were then determined using Image J software.

**Reverse transcription polymerase chain reaction (RT‐PCR)**. Total RNA was isolated after 24 h ligand treatment using TRIzol reagent (Invitrogen, Life Technologies) according to the manufacturer's instructions. RNA was quantified, and 1 μg of RNA was used for cDNA preparation using Verso cDNA synthesis kit. The relative transcript expression level for genes was measured by quantitative real‐time PCR using SYBR Green‐based method. Δ*C*_t_ values were calculated by the difference in threshold cycles (*C*_t_) between test and control samples. 18 s rRNA gene was used as an internal control for normalizing the cDNA concentration of each sample. Primers used for monitoring the gene expressions are as follows:

$$c\text{-}Myc\;\mathrm{forward}:\;5{}^{'}\text{-}\mathrm{CTGCGACGAGGAGGAGGACT}\text{-}3{}^{'}$$

$$c\text{-}Myc\;\mathrm{reverse}:\;5{}^{'}\text{-}\mathrm{GGCAGCAGCTCGAATTTCTT}\text{-}3{}^{'}$$

$$18s\;\mathrm{rRNA}\;\mathrm{forward}:\;5{}^{'}\text{-}\mathrm{GATTCCGTGGGTGGTGGTGC}\text{-}3{}^{'}$$

$$18s\;\mathrm{rRNA}\;\mathrm{reverse}:\;5{}^{'}\text{-}\mathrm{AAGAAGTTGGGGGACGCCGA}\text{-}3{}^{'}$$

For amplification of *c‐Myc* genes, the samples were subjected to pre‐incubation at 95 °C for 10 min and then 40 cycles of 95 °C (15 s) and 60 °C (60 s).

**Cell cycle analysis**: Exponentially growing HeLa cells were seeded in 6‐well plates at a density of 0.1×10^6^ cells/mL and allowed to grow in DMEM complete media (Thermofisher) for 24 h. Cells were then treated with fragments **5**, **7** and **12** (6 μM) in fresh DMEM media for 24 h and 48 h in 2 different plates and 1.2 μL DMSO in 2 mL DMEM serves as control. Supernatant of seeded HeLa cells culture were discarded and wells were refilled with fresh media containing compounds/ DMSO controls. Treated cells were kept in the incubator (37 °C, 5 % CO_2_) for 24 and 48 h in 2 plates.

**Hoechst staining protocol**: The DMEM was freshly supplemented with Hoechst (1 : 10 000 of 90 mM; final conc. 9 μM) to prepare a staining solution 24 and 48 h after treatment induction, supernatant of treated cells were collected in a 15 mL tube (many cells died and were not adherent any more), centrifuged and pellets were resuspended in 500 μL fresh medium containing Hoechst (staining solution) 500 μL staining solution was added to adherent cells in wells (e. g., control cells). Final staining volume was 1 mL (500 μL in plate+500 μL resuspended pellets) for all conditions. Hoechst staining (1 mL, 9 μM) was performed at 37 °C, 5 % CO_2_ for 30 min. After staining cells were collected (cell in supernatant, adherent cells by trypsin treatment), washed with PBS and transferred in a FACS tube. The unstained cells serve as FACS control (negative signal).

**Statistics**: All results are representative of three independent experiments and the data presented are expressed as mean ±SD. Statistical analysis was performed using Student's t‐test except that the Young's modulus is performed using Kruskal‐Waillis test, and *p* <0.05 was regarded as statistically significant.

## Conflict of interest

The authors declare no conflict of interest.

## Supporting information

As a service to our authors and readers, this journal provides supporting information supplied by the authors. Such materials are peer reviewed and may be re‐organized for online delivery, but are not copy‐edited or typeset. Technical support issues arising from supporting information (other than missing files) should be addressed to the authors.

Supplementary
