# Supplementary material for: Synthesis and in Vitro Evaluation of Novel 5‐Nitroindole Derivatives as c‐Myc G‐Quadruplex Binders with Anticancer Activity
Source: ChemMedChem. 2021 Mar 22;16(10):1667–79. doi: 10.1002/cmdc.202000835 (PMC8252724; doi:10.1002/cmdc.202000835)

ChemMedChem

Supporting Information

Synthesis and *in Vitro* Evaluation of Novel 5-Nitroindole Derivatives as *c-Myc* G-Quadruplex Binders with Anticancer Activity

Vijaykumar D. Nimbarte, Julia Wirmer-Bartoschek, Santosh L. Gande, Islam Alshamleh, Marcel Seibert, Hamid Reza Nasiri, Frank Schnütgen, Hubert Serve, and Harald Schwalbe*

Supporting Information

Table of Content

List of compounds

Table S1. List of screened commercial and synthesized fragments with origin

Thiazole Displacement assays

Figure S1. Dissociation constant (K_d) values of TO for *c-MYC* Sequence

Figure S2 and S3. Thiazole Displacement assays of initial library of 129 fragments Series **I**, Series **II**

Figure S4. Thiazole Displacement assays of 52 synthesized screened fragments Series III

Figure S5. Dose response curves based on FID titration Ligands: c-Myc

Microscale Thermophoresis (MST) Assay

Figure S6. Dose response curves based on MST titration Ligands: c-Myc

Figure S7. Dose response curves based on MST titration Ligands: Duplex DNA sequence

Table S2: Kd values based on MST assays (Ligand: Duplex DNA)

Fluorescence binding titration

Figure S8. Binding Stoichiometry of Fragment: DNA (2:1) with Job Plot analysis

Figure S9. Dose response curves based on FAM titration Ligands: c-Myc

In vitro investigations

Figure S10: Western plot analysis of protein expression

Figure S11: Cell cycle analysis

Figure S12: ROS scavenger-NAC reversed compound 7 inhibited cell proliferation and compound 7 induced intracellular ROS production in Hela cancer cells.

Figure S13: Cell viability with Normal Kidney (NKE) cells for compound 5 and 7

Table S3: Effect of Fragments 5, 7 and 12 on cell cycle after 24 h

Table S4: Effect of Fragments 5, 7 and 12 on cell cycle after 48 h

Drug likeliness of compounds

Figure S14. SWISS ADME Radar of 9a and novel substituted 5-nitro indole derivatives 12, 7 & 5

Table S5. Physiochemical Properties of Fragments (SWISS-ADME-Filters)

Table S6. Pharmacokinetics, Drug-Likeliness and Medicinal Chemistry (SWISS-ADME-Filters)

Table S7. Lipophilicity and water Solubility Properties of Fragments (SWISS-ADME-Filters)

Supporting information for Synthesis

General Procedure and Synthetic Scheme 4 and Scheme 5

HPLC methods used to assess the purity of compounds

HPLC Chromatograms for 3, 9a, 5, 7, 12 and 5a

NMR supplementary information

Supplementary data and HRMS/ESI Spectra

List of compounds

Table S1. List of screened commercial and synthesized fragments with origin (commercial or synthesized) and SMILES screened with Fluorescent Intercalator Displacement (FID) Assay

HIT	STRUCTURE	SMILE NOTATION	ORIGIN (SOURCE)
A1	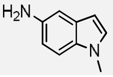	CN1C=CC2=C1C=CC(N)=C2	**Synthesized (3)**
A2	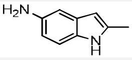	CC1=CC2=C(N1)C=CC(N)=C2	Commercial
A3	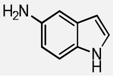	NC1=CC2=C(NC=C2)C=C1	**Synthesized (31)**
A4	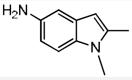	CN1C(C)=CC2=C1C=CC(N)=C2	Commercial
A5	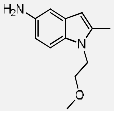	COCCN1C(C)=CC2=C1C=CC(N)=C2	Commercial
A6	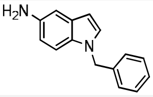	NC1=CC2=C(C=C1)N(CC1=CC=CC=C1)C=C2	Commercial
A7	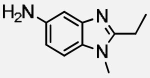	CCC1=NC2=C(C=CC(N)=C2)N1C	Commercial
A8	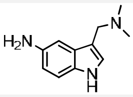	CN(C)CC1=CNC2=C1C=C(N)C=C2	**Synthesized (9a)**
A9	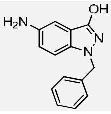	NC1=CC2=C(C=C1)N(CC1=CC=CC=C1)N=C2O	Commercial
A10	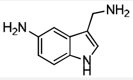	NCC1=CNC2=C1C=C(N)C=C2	Commercial
A11	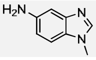	CN1C=NC2=C1C=CC(N)=C2	Commercial
A12	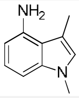	CN1C=C(C)C2=C1C=CC=C2N	Commercial
A13	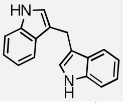	C(C1=CNC2C=CC=CC12)C1=CNC2=C1C=CC=C2	Commercial
A14	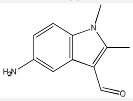	CN1C(C)=C(CCN)C2=C1C=CC(N)=C2	Commercial
A15	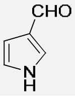	O=CC1=NNC2=CC=CC=C12	Commercial
A16	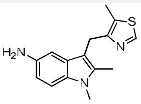	CN1C(C)=C(CC2=C(C)SC=N2)C2=C1C=CC(N)=C2	Commercial

**A17**	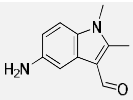	[H]C(=O)C1=C(C)N(C)C2=C1C=C(N)C=C2	Commercial
**A18**	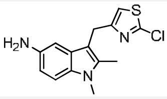	CN1C(C)=C(CC2=CSC(Cl)=N2)C2=C1C=CC(N)=C2	Commercial
**A19**	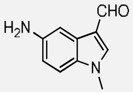	[H]C(=O)C1=CN(C)C2=C1C=C(C)C=C2	Commercial
**A20**	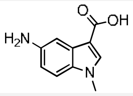	CN1C=C(C(O)=O)C2=C1C=CC(N)=C2	Commercial
**A21**	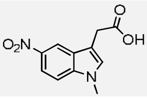	CN1C=C(CC(O)=O)C2=C1C=CC(=C2)N(=O)=O	Commercial
**A22**	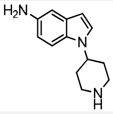	NC1=CC2=C(C=C1)N(C=C2)C1CCNCC1	Commercial
**A23**	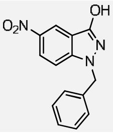	NCC1=NN(CC2=CC=CC=C2)C2=C1C=C(C=C2)N(=O)=O	Commercial
**A24**	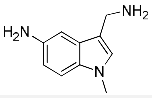	CN1C=C(CN)C2=C1C=CC(N)=C2	Commercial
**A25**	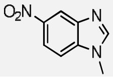	CN1C=NC2=C1C=CC(=C2)N(=O)=O	Commercial
**A26**	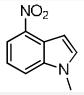	CN1C=CC2=C1C=CC(=C2)N(=O)=O	Commercial
**A27**	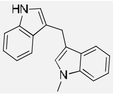	CN1C=C(CC2=CNC3=C2C=CC=C3)C2C=CC=CC12	Commercial
**A28**	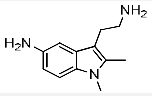	CN1C(C)=C(CCN)C2=C1C=CC(N)=C2	Commercial
**A29**	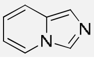	C1=NC=C2C=CC=CN12	Commercial
**A30**	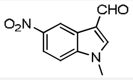	[H]C(=O)C1=CN(C)C2=C1C=C(N)C=C2	Commercial
**A31**	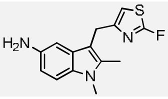	CN1C(C)=C(CC2=CSC(F)=N2)C2=C1C=CC=C2	Commercial
**A32**	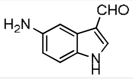	[H]C(=O)C1=CNC2=C1C=C(N)C=C2	Commercial
**A33**	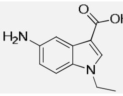	CCN1C=C(C(O)=O)C2=C1C=CC(N)=C2	Commercial

**A34**	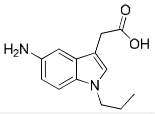	CCCN1C=C(CC(O)=O)C2=C1C=CC=C2	Commercial
**A35**	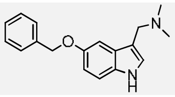	CN(C)CC1=CN(C)C2=C1C=C(OCC1=CC=CC=C1)C=C2	Commercial
**A36**	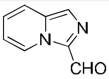	O=CC1=NC=C2C=CC=CN12	Commercial
**A37**	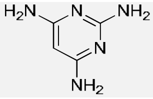	NC1=CC(N)=NC(N)=N1	Commercial
**A38**	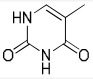	CC1=CNC(=O)NC1=O	Commercial
**A39**	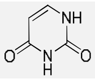	O=C1NC=CC(=O)N1	Commercial
**A40**	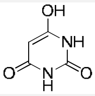	OC1=CC(=O)NC(=O)N1	Commercial
**A41**	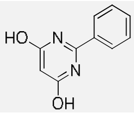	OC1=CC(O)=NC(=N1)C1=CC=CC=C1	Commercial
**A42**	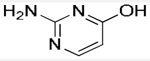	NC1=NC(O)=CC=N1	Commercial
**A43**	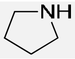	C1CCNC1	Commercial
**A44**		NC1=NC=NC2=C1NN=C2	Commercial
**A45**	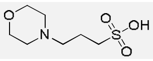	O.OS(=O)(=O)CCCN1CCOCC1	Commercial
**A46**	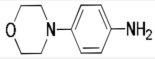	NC1=CC=C(C=C1)N1CCOCC1	Commercial
**A47**	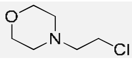	ClCCN1CCOCC1	Commercial
**A48**	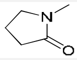	CN1CCCC1=O	Commercial
**A49**	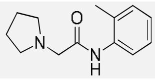	CC1=CC=CC=C1NC(=O)CN1CCCC1	Commercial
**A50**	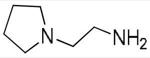	NCCN1CCCC1	Commercial
**A51**	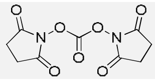	O=C(ON1C(=O)CCC1=O)ON1C(=O)CCC1=O	Commercial
**A52**	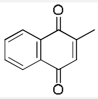	CC1=CC(=O)C2=C(C=CC=C2)C1=O	Commercial
**A53**	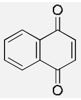	O=C1C=CC(=O)C2=C1C=CC=C2	Commercial
**A54**	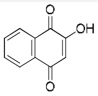	OC1=CC(=O)C2=C(C=CC=C2)C1=O	Commercial

**A55**	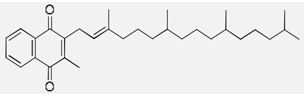	CC(C)CCCC(C)CCCC(C)CCC\C(C)=C\CC1=C(C)C(=O)C2=C(C=CC=C2)C1=O	Commercial
**A56**	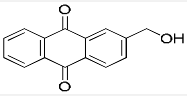	OCC1=CC=C2C(=O)C3=C(C=CC=C3)C(=O)C2=C1	Commercial
**A57**	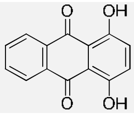	OC1=C2C(=O)C3=C(C=CC=C3)C(=O)C2=C(O)C=C1	Commercial
**A58**	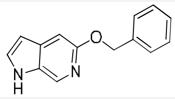	C(OC1=NC=C2NC=CC2=C1)C1=CC=CC=C1	Commercial
**A59**	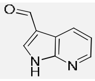	O=CC1=CNC2=C1C=CC=N2	Commercial
**A60**	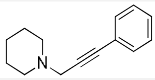	C(C#CC1=CC=CC=C1)N1CCCCC1	Commercial
**A61**	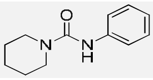	O=C(NC1=CC=CC=C1)N1CCCCC1	Commercial
**A62**	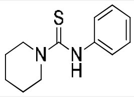	S=C(NC1=CC=CC=C1)N1CCCCC1	Commercial
**A63**	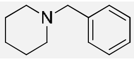	C(N1CCCCC1)C1=CC=CC=C1	Commercial
**A64**	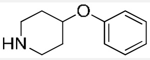	C1CC(CCN1)OC1=CC=CC=C1	Commercial
**A65**	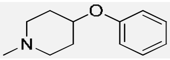	CN1CCC(CC1)OC1=CC=CC=C1	Commercial
**A66**	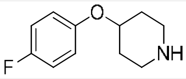	FC1=CC=C(OC2CCNCC2)C=C1	Commercial
**A67**	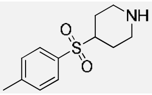	CC1=CC=C(C=C1)S(=O)(=O)C1CCNCC1	Commercial
**A68**	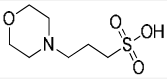	OS(=O)(=O)CCCN1CCOCC1	Commercial
**A69**	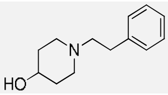	OC1CCN(CCC2=CC=CC=C2)CC1	Commercial
**A70**	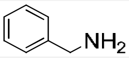	NCC1=CC=CC=C1	Commercial
**A71**	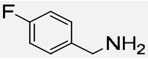	NCC1=CC=C(F)C=C1	Commercial
**A72**	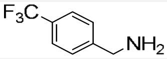	NCC1=CC=C(C=C1)C(F)(F)F	Commercial
**A73**	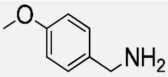	COC1=CC=C(CN)C=C1	Commercial
**A74**	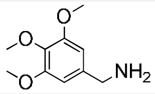	COC1=CC(CN)=CC(OC)=C1OC	Commercial
**A75**	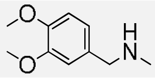	CNCC1=CC=C(OC)C(OC)=C1	Commercial

**A76**	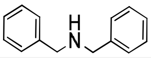	C(NCC1=CC=CC=C1)C1=CC=CC=C1	Commercial
**A77**	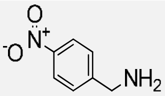	NCC1=CC=C(C=C1)[N+]([O-])=O	Commercial
**A78**	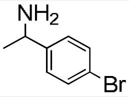	CC(N)C1=CC=C(Br)C=C1	Commercial
**A79**	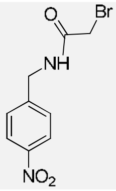	[O-][N+](=O)C1=CC=C(CNC(=O)CBr)C=C1	Commercial
**A80**	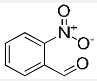	[O-][N+](=O)C1=CC=CC=C1C=O	Commercial
**A81**	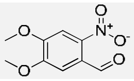	COC1=C(OC)C=C(C(C=O)=C1)[N+]([O-])=O	Commercial
**A82**	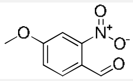	COC1=CC=C(C=O)C(=C1)[N+]([O-])=O	Commercial
**A83**	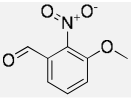	COC1=C(C(C=O)=CC=C1)[N+]([O-])=O	Commercial
**A84**	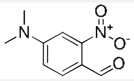	CN(C)C1=CC=C(C=O)C(=C1)[N+]([O-])=O	Commercial
**A85**	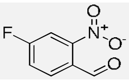	[O-][N+](=O)C1=CC(F)=CC=C1C=O	Commercial
**A86**	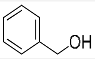	OCC1=CC=CC=C1	Commercial
**A87**	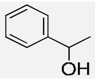	CC(O)C1=CC=CC=C1	Commercial
**A88**	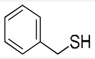	SCC1=CC=CC=C1	Commercial
**A89**	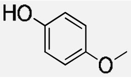	COC1=CC=C(O)C=C1	Commercial
**A90**	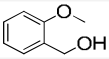	COC1=CC=CC=C1CO	Commercial
**A91**	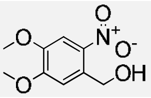	COC1=C(OC)C=C(C(CO)=C1)[N+]([O-])=O	Commercial
**A92**	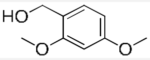	COC1=CC=C(CO)C(OC)=C1	Commercial
**A93**	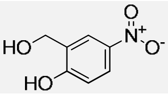	OCC1=CC(=CC=C1O)[N+]([O-])=O	Commercial
**A94**	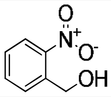	OCC1=CC=CC=C1[N+]([O-])=O	Commercial
**A95**	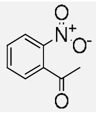	CC(=O)C1=CC=CC=C1[N+]([O-])=O	Commercial

**A96**	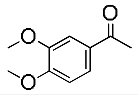	COC1=CC=C(C=C1OC)C(C)=O	Commercial
**A97**	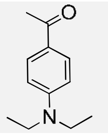	CCN(CC)C1=CC=C(C=C1)C(C)=O	Commercial
**A98**	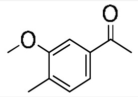	COC1=CC(=CC=C1C)C(C)=O	Commercial
**A99**	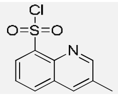	CC1=CC2=CC=CC(=C2N=C1)S(Cl)(=O)=O	Commercial
**A100**	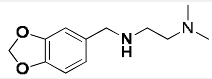	CN(C)CCNCC1=CC=C2OCOC2=C1	Commercial
**A101**	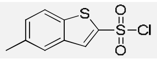	CC1=CC=C2SC(=CC2=C1)S(Cl)(=O)=O	Commercial
**A102**	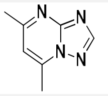	CC1=NC2=NC=NN2C(C)=C1	Commercial
**A103**	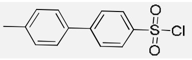	CC1=CC=C(C=C1)C1=CC=C(C=C1)S(Cl)(=O)=O	Commercial
**A104**	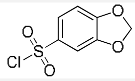	ClS(=O)(=O)C1=CC=C2OCOC2=C1	Commercial
**A105**	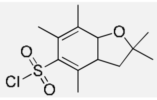	CC1=C(C(C)=C(C)C2OC(C)(C)CC12)S(Cl)(=O)=O	Commercial
**A106**	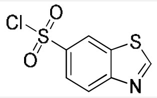	CC1=C(C(C)=C(C)C2OC(C)(C)CC12)S(Cl)(=O)=O	Commercial
**A107**	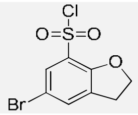	ClS(=O)(=O)C1=C2OCCC2=CC(Br)=C1	Commercial
**A108**	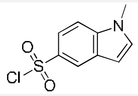	CN1C(C)=CC2=C1C=CC=C2S(Cl)(=O)=O	Commercial
**A109**	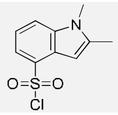	CN1C(C)=CC2=C1C=CC=C2S(Cl)(=O)=O	Commercial
**A110**	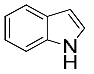	N1C=CC2=CC=CC=C12	Commercial
**A111**	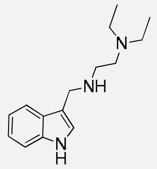	CCN(CC)CCNCC1=CNC2=CC=CC=C12	Commercial
**A112**	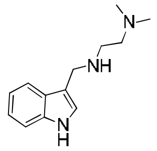	CN(C)CCNCC1=CNC2=CC=CC=C12	Commercial

**A113**	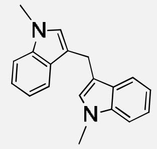	CN1C=C(CC2=CC3C=CC=CC3N2C)C2=C1C=CC=C2	Commercial
**A114**	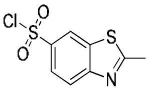	CC1=NC2=CC=C(C=C2S1)S(Cl)(=O)=O	Commercial
**A115**	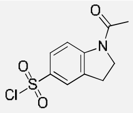	CC(=O)N1CCC2=C1C=CC(=C2)S(Cl)(=O)=O	Commercial
**A116**	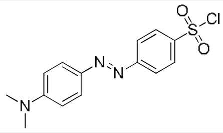	CN(C)C1=CC=C(C=C1)\N=N\C1=CC=C(C=C1)S(Cl)(=O)=O	Commercial
**A117**	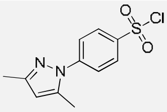	CC1=NN(C(C)=C1)C1=CC=C(C=C1)S(Cl)(=O)=O	Commercial
**A118**	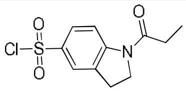	CCC(=O)N1CCC2=C1C=CC(=C2)S(Cl)(=O)=O	Commercial
**A119**	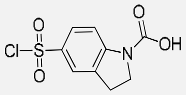	OC(=O)N1CCC2=C1C=CC(=C2)S(Cl)(=O)=O	Commercial
**A120**	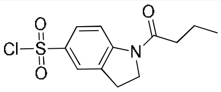	CCCC(=O)N1CCC2=C1C=CC(=C2)S(Cl)(=O)=O	Commercial
**A121**	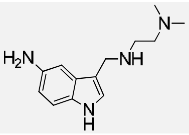	CN(C)CCNCC1=CNC2=CC=C(N)C=C12	Commercial
**A122**	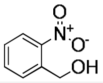	OCC1=CC=CC=C1[N+]([O-])=O	Commercial
**A123**	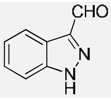	O=CC1=CNC2=NC=CC=C12	Commercial
**A124**	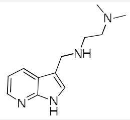	CN(C)CCNCC1=CNC2=NC=CC=C12	Commercial
**A125**	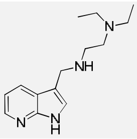	CCN(CC)CCNCC1=CNC2=NC=CC=C12	Commercial
**A126**	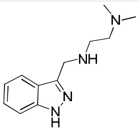	CN(C)CCNCC1=NNC2=CC=CC=C12	Commercial

**A127**	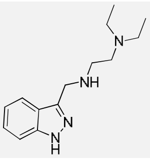	CCN(CC)CCNCC1=NNC2=CC=CC=C12	Commercial
**A128**	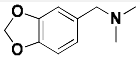	CN(C)CC1=CC2=C(OCO2)C=C1	Commercial
**A129**	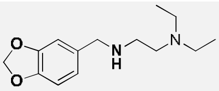	CCN(CC)CCNCC1=CC2=C(OCO2)C=C1	Commercial
**(24c)**	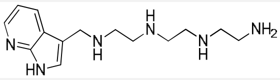	NCCNCCNCCNCC1=CNC2=C1C=CC=N2	**Synthesized (24c)**
**(2A)**	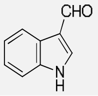	[H]C(=O)C1=CNC2=C1C=CC=N2	**Synthesized (2a)**
**(19b)**	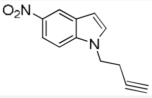	C#CCCN1C=CC2=C1C=CC=C2	**Synthesized (19b)**
**(8)**	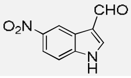	CN1C=CC2=C1C=CC(N)=C2	**Synthesized (8)**
**(9)**	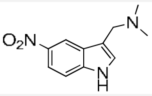	CN(C)CC1=CNC2=C1C=C(C=C2)N(=O)=O	**Synthesized (9)**
**(10)**	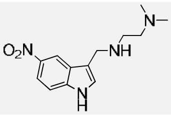	CN(C)CNCC1=CNC2=C1C=C(C=C2)N(=O)=O	**Synthesized (10)**
**(12)**	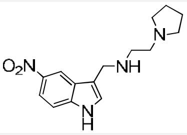	O=N(=O)C1=CC2=C(NC=C2CNCCN2CCCC2)C=C1	**Synthesized (12)**
**(11)**	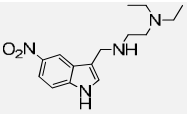	CCN(CC)CCNCC1=CNC2=C1C=C(C=C2)N(=O)=O	**Synthesized (11)**
**(7)**	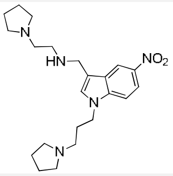	O=N(=O)C1=CC2=C(C=C1)N(CCN1CCCC1)C=C2CNCCN1CCCC1	**Synthesized (7)**
**(17a)**	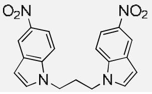	O=N(=O)C1=CC2C=CN(CCCN3C=CC4=C3C=CC(=C4)N(=O)=O)C2C=C1	**Synthesized (17a)**
**(5)**	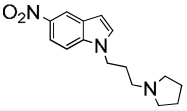	O=N(=O)C1=CC2=C(C=C1)N(CCCN1CCCC1)C=C2	**Synthesized (5)**
**(19a)**	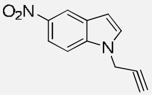	O=N(=O)C1=CC2=C(C=C1)N(CC#C)C=C2	**Synthesized (19a)**

**(20)**	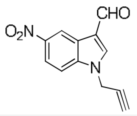	[H]C(=O)C1=CN(CC#C)C2=C1C=C(C=C2)N(=O)=O	**Synthesized (20)**
**(21a)**	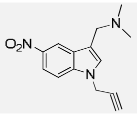	CN(C)CC1=CN(CC#C)C2=C1C=C(C=C2)N(=O)=O	**Synthesized (21a)**
**(4b)**	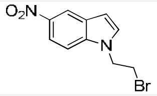	BrCCN1C=CC2=C1C=CC(=C2)N(=O)=O	**Synthesized (4b)**
**(4a)**	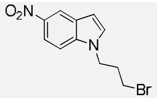	BrCCCN1C=CC2=C1C=CC(=C2)N(=O)=O	**Synthesized (4a)**
**(14)**	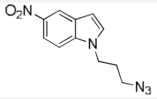	N=[N]=NCCCN1C=CC2=C1C=CC(=C2)N(=O)=O	**Synthesized (14)**
**(21b)**	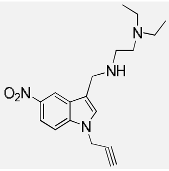	CCN(CC)CCNCC1=CN(CC#C)C2=C1C=C(C=C2)N(=O)=O	**Synthesized (21b)**
**(16)**	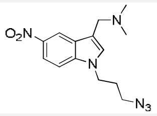	CN(C)CC1=CN(CCCN=[N]#N)C2=C1C=C(C=C2)N(=O)=O	**Synthesized (16)**
**(6)**	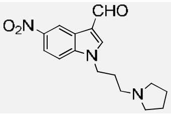	[H]C(=O)C1=CN(CCCN2CCCC2)C2=C1C=C(C=C2)N(=O)=O	**Synthesized (6)**
**(15)**	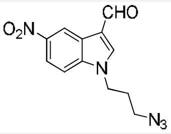	[H]C(=O)C1=CN(CCCN=[N]#N)C2=C1C=C(C=C2)N(=O)=O	**Synthesized (15)**
**(16A)**	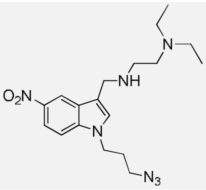	CCN(CC)CCNCC1=CN(CCCN=[N+]=N)C2=C1C=C(C=C2)N(=O)=O	**Synthesized (16a)**
**(23a)**	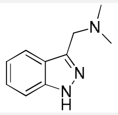	CN(C)CC1=NNC2=C1C=CC=C2	**Synthesized (23a)**
**(26b)**	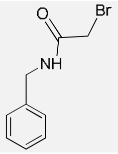	BrCC(=O)NCC1=CC=CC=C1	**Synthesized (26b)**
**(27b)**	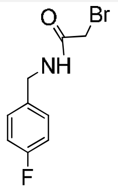	FC1=CC=C(CNC(=O)CBr)C=C1	**Synthesized (27b)**

**(30b)**	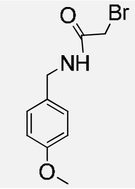	COC1=CC=C(CNC(=O)CBr)C=C1	**Synthesized (30b)**
**(29b)**	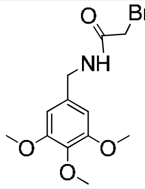	COC1=CC(CNC(=O)CBr)=CC(OC)=C1OC	**Synthesized (29b)**
**(28b)**	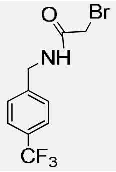	FC(F)(F)C1=CC=C(CNC(=O)CBr)C=C1	**Synthesized (28b)**
**(26d)**	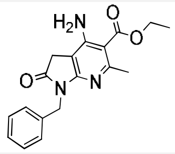	CCOC(=O)C1=C(N)C2=C(N=C1C)N(CC1=CC=CC=C1)C(=O)C2	**Synthesized (26d)**
**(27d)**	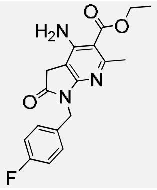	CCOC(=O)C1=C(N)C2=C(N=C1C)N(CC1=CC=C(F)C=C1)C(=O)C2	**Synthesized (27d)**
**(28d)**	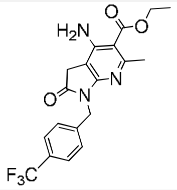	CCOC(=O)C1=C(N)C2=C(N=C1C)N(CC1=CC=C(C=C1)C(F)(F)F)C(=O)C2	**Synthesized (28d)**
**(29d)**	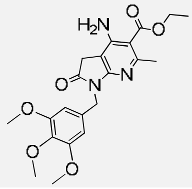	CCOC(=O)C1=C(N)C2=C(N=C1C)N(CC1=CC(OC)=C(OC)C(OC)=C1)C(=O)C2	**Synthesized (29d)**
**(30d)**	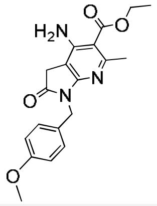	CCOC(=O)C1=C(N)C2=C(N=C1C)N(CC1=CC=C(OC)C=C1)C(=O)C2	**Synthesized (30d)**
**(26c)**	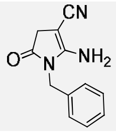	NC1=C(CC(=O)N1CC1=CC=CC=C1)C#N	**Synthesized (26c)**

**(27c)**	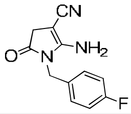	NC1=C(CC(=O)N1CC1=CC=C(F)C=C1)C#N	**Synthesized (27c)**
**(28c)**	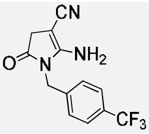	NC1=C(CC(=O)N1CC1=CC=C(C=C1)C(F)(F)F)C#N	**Synthesized (28c)**
**(30c)**	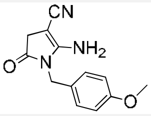	COC1=CC=C(CN2C(=O)CC(C#N)=C2N)C=C1	**Synthesized (30c)**
**(29c)**	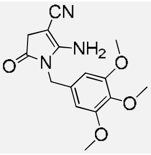	COC1=CC(CN2C(=O)CC(C#N)=C2N)=CC(OC)=C1OC	**Synthesized (29c)**
**(3a)**	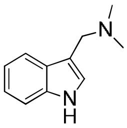	CN(C)CC1=CNC2=CC=CC=C12	**Synthesized (3a)**
**(11A)**	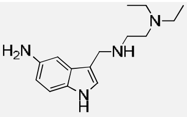	CCN(CC)CCNCC1=CNC2=CC=C(N)C=C12	**Synthesized (11a)**
**(3b)**	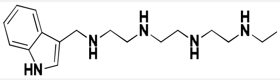	NCCNCCNCCNCCNCC1=CNC2=CC=CC=C12	**Synthesized (3b)**
**(13)**	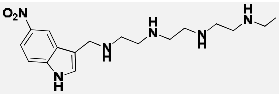	NCCNCCNCCNCC1=CNC2=CC=CC=C12	**Synthesized (13)**
**(24b)**	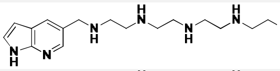	NCCNCCNCCNCCNCC1=CC2=C(NC=C2)N=C1	**Synthesized (24b)**
**(25B)**	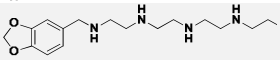	NCCNCCNCCNCCNCC1=CC2=C(OCO2)C=C1	**Synthesized (25b)**
**(23B)**	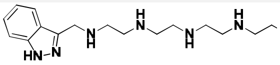	NCCNCCNCCNCCNCC1=NNC2=CC=CC=C12	**Synthesized (23b)**
**(24A)**	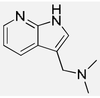	CN(C)CC1=CNC2=NC=CC=C12	**Synthesized (24a)**
**(24E)**	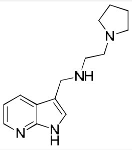	C(CN1CCCC1)NCC1=CNC2=NC=CC=C12	**Synthesized (24e)**
**(23E)**	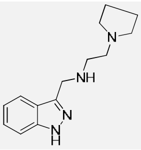	C(CN1CCCC1)NCC1=NNC2=CC=CC=C12	**Synthesized (23e)**
**(25E)**	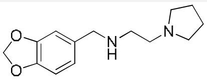	C(CN1CCCC1)NCC1=CC=C2OCOC2=C1	**Synthesized (25e)**

**(25D)**	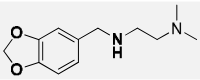	CN(C)CCNCC1=CC2=C(OCO2)C=C1	**Synthesized (25d)**
**(5A)**	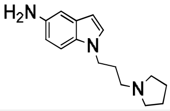	NC1=CC2=C(C=C1)N(CCCN1CCCC1)C=C2	**Synthesized (5a)**
**(5B)**	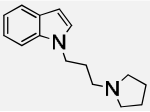	C(CN1CCCC1)CN1C=CC2=C1C=CC=C2	**Synthesized (5b)**

Thiazole Displacement assays

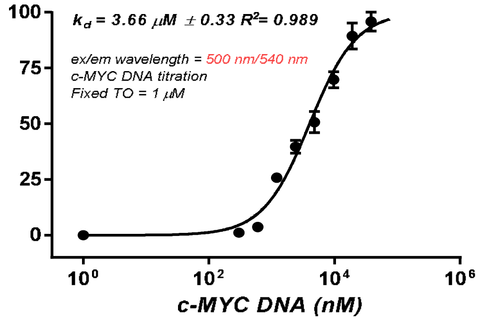

Figure S1. Dissociation constant (K_d) values of TO for *c-MYC* **A)** Titration of fixed Thiazole Orange: c(TO) = 1 μM; c(cMYC)= 0 μM, 0.27 μM, 0.55 μM, 1.09 μM, 2.18 μM, 4.36 μM, 8.72 μM, 17.44 μM, 34.88 μM, 69.76 μM; c(DMSO) = 10%; 20 mM Na-cacodylate, 140 mM KCl, pH = 7, r.t ; The error bars represent the standard error of the mean (SEM) calculated from three replicates.

- **Initial screening of 129 fragments which in total includes 126 commercially available and 3 In-house synthesized fragments (A1, A3 and A8 please see compound 3, 9a and 31 in scheme 1)**

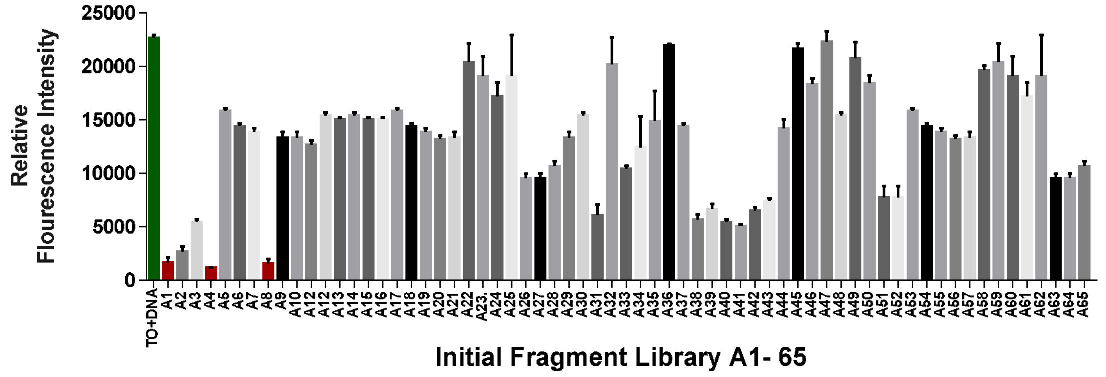

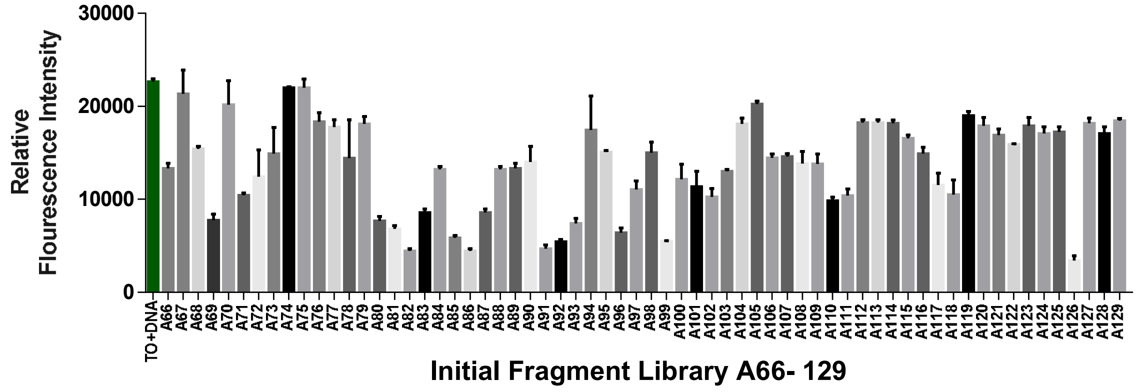

Figure S2 and S3. Thiazole Displacement assays of initial screening of fragments Series I, Series II; **Experimental condition:** 0.25 μM DNA, 0.5 μM Thiazole Orange, 10 % DMSO, 20 mM Na caco, 140 mM KCl, pH 7 (25 μL/well)

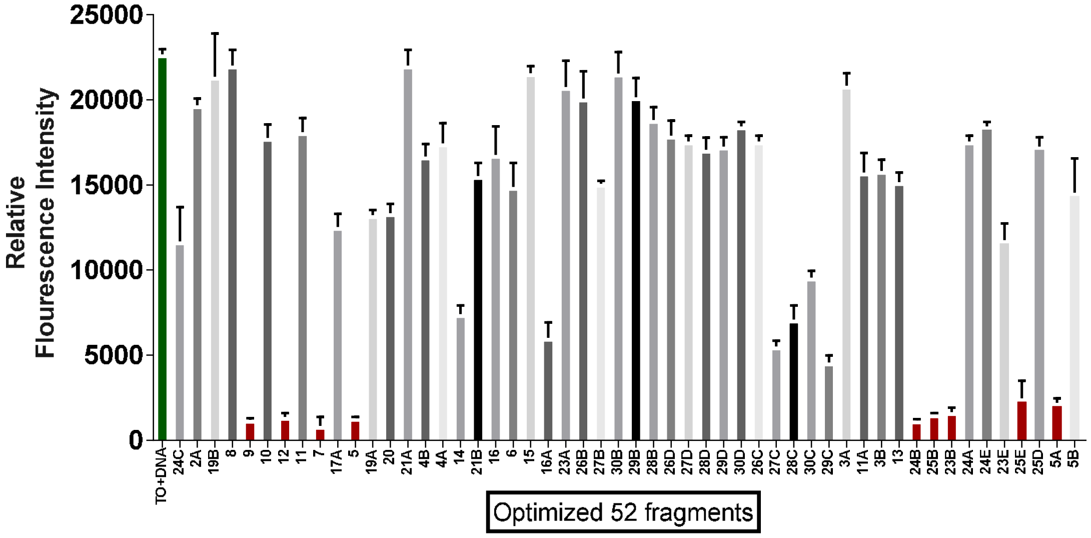

Figure S4. Thiazole Displacement assays of screened fragments Series I, Series II; **Experimental condition:** 0.25 μM DNA, 0.5 μM Thiazole Orange, 10 % DMSO, 20 mM Na caco, 140 mM KCl, pH 7 (25 μL/well)

Data analysis: The final DC_{50} values were determine by fitting log-transformed concentration values and the effect data were to a four-parameter logistic equation in GraphPad Prism 6.0.. The original, % control, or % inhibition data are represented by Y along with their minimal (min) and maximal (max) values. The inhibitor concentration is represented by X, DC_{50} is the concentration at 50% maximal value, and Hill Slope is the slope factor:

Equation 2…

$$\mathbf{Y = min + \frac{(max - min)}{1 + 10^{((X - \log DC_{50}) \times Hill\ slope}} \times 100}$$

Definition of DC_{50}: Concentration of inhibitor that displaces 50 % of bound thiazole-orange from respective DNA sequences

TO-Displacement % FID = 100 + [100 + F/F_0]

$F = F_{(Ligand + DNA + TO)} - F_{(Buffer + DNA)} - F_{(Ligand + DNA)}$

$F_0 = F_{(DNA+TO)} - F_{(Buffer + TO)}$

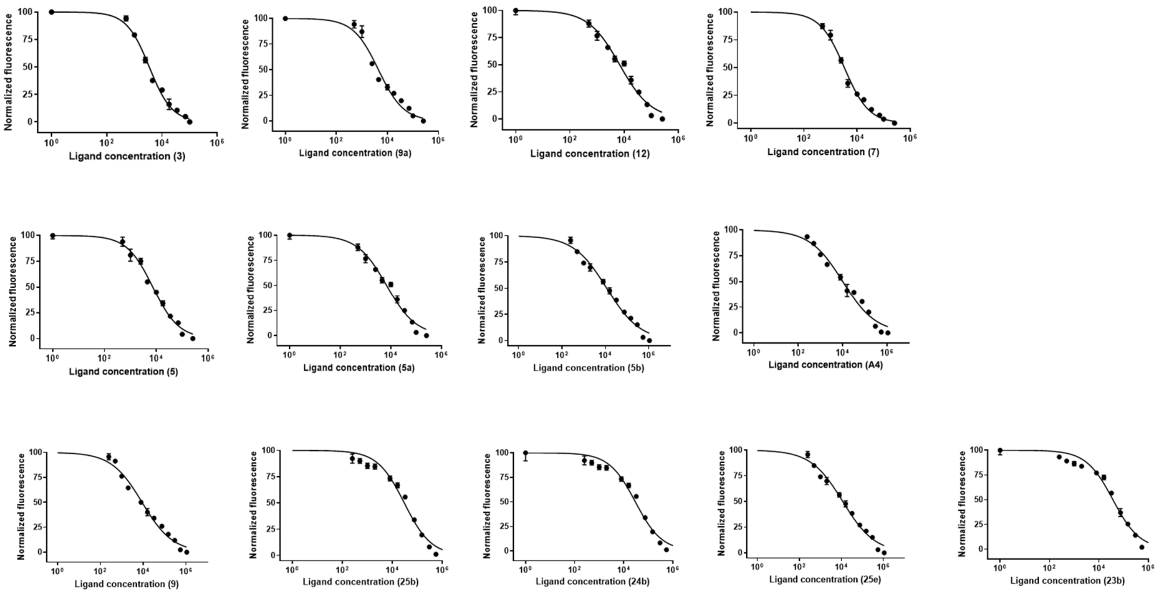

Figure S5. Dose response curves based on FID titration Ligands: c-Myc DC_{50} values for the most active ligands for G-quadruplex and Duplex DNA sequences; Titration scheme of c-MYC: c(TO) = 0,5 µM; Buffer conditions10 mM Tris-HCl buffer, pH 7.4, in the presence of 150 mM KCl

Microscale Thermophoresis (MST) Assay

Analayis of MST Data: **:** The dissociation constants (K_D) were determined with MO. Binding Affinity Software (Nano Temper Technologies GmbH, München, Germany) using the K_D binding model below; built-in analysis in the MO.

$$\mathbf{f(c)} = \mathbf{unbound} + \frac{\mathbf{(Bound - unbound) \times c(target) + K_D - \sqrt{(c + c(target) + K_D)^2)} - 4\mathit{c} \times \mathit{c(tar}} \text{[illegible]}}{\mathbf{2c(target)}}$$

The equation is based on the Langmuir binding isotherm. The optimal time region to assess binding was determined manually by selecting a region with a high signal-to-noise ratio (>5). Where, f(c) is the fraction bound at a given ligand concentration c. Unbound is either the normalized fluorescence signal (F_{norm}; MST mode) or raw fluorescence counts (initial fluorescence mode) of the target alone. Bound is either the normalized fluorescence signal or raw fluorescence counts (initial fluorescence mode) of the *c-Myc* DNA - ligand complex. K_D is the dissociation constant or binding affinity, and c (target) is the final concentration of the target in the assay.

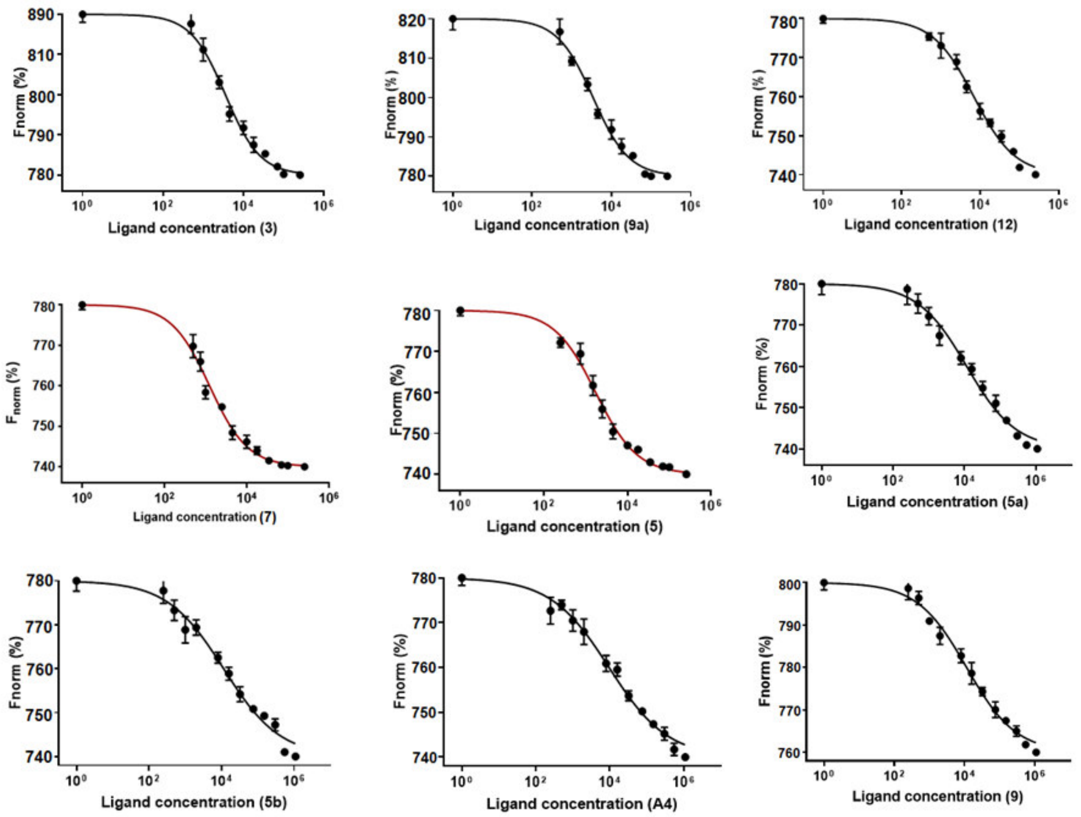

Figure S6. Dose response curves based on MST titration Ligands: c-Myc; Buffer conditions10 mM Tris-HCl buffer, pH 7.4, in the presence of 150 mM KCl

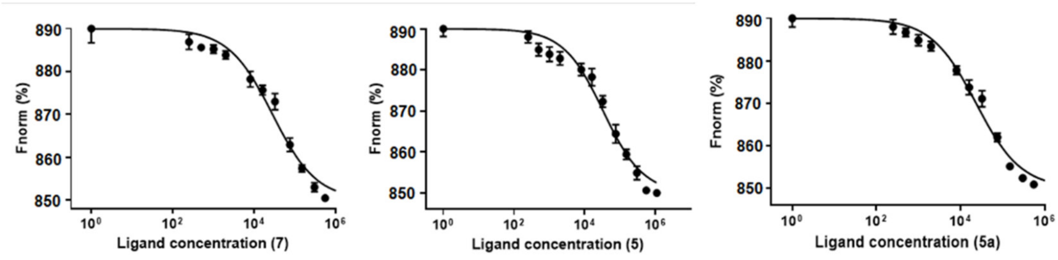

Figure S7. Dose response curves based on MST titration Ligands: Duplex DNA; Buffer conditions10 mM Tris-HCl buffer, pH 7.4, in the presence of 150 mM KCl

Table S2. DNA oligonucleotides used in Biophysical Studies and Comparison of Binding Data Obtained for indole derivatives from MST Assays

FAM-DNA Sequence (5′---3′)	K_d (μM) *		
	**7**	**5**	**5a**
dsDNA 24bp CGCGAATTCGCGCGCGAATTCGCG (24-mer)	32.3±1.69	38.1±2.39	18.0±1.33

Conditions: Oligonucleotide sequences were diluted from stock to the required concentration (10 μM) in 10 mM Tris-HCl buffer, pH 7.4, in the presence of 150 mM KCl; concentration of fragments was varied from 0 to 100 μM for tested the K_d of duplex DNA; experiments were done in triplicates

Fluorescence binding titration

Binding stoichiometries were measured by continuous variation binding analysis by the method according to Job. All solutions were freshly prepared in other appropriate oligonucleotide was heated at 95 °C for three minutes, allowed to cool to RT, and diluted to 50 nM in 25 mM Tris buffer (pH 6.4, containing 50 mM KCl). Fluorescence spectra were recorded on an Infinite 200 Pro Micro Plate Reader (Tecan i-control) at 25 °C. Fragment 7 and MYC (pu22): (c-MYC: TGA GGG TGG GTA TGG GTA A (22-mer) were varied for a fixed and constant summed concentration of 1 μM. The mole fractions of fragment 7 ranging from 0, 0.1, 0.2, 0.3, 0.4, 0.5, 0.6, 0.7, 0.8, 0.9, 1.0 μM) in 10 mM Tris-HCl, 100 mM KCl buffer (pH 7.4) were added to the MYC (pu22) (mole fraction: 1.0, 0.9, 0.8, 0.7, 0.6, 0.5, 0.4, 0.3, 0.2, 0.1, 0 μM), respectively. Fluorescence intensity was recorded at an excitation wavelength of 400 nm, with the resulting emission wavelengths from 315-600 nm. Experiments were performed in triplicate and were repeated three times. Each reading is a mean of three scans. The normalization of the fluorescence intensity values were done by following formula,

$$FI_{Normalized} = \frac{FI_x - FI_{initial}}{FI_{max} - FI_{initial}}$$

Where,

$FI_{Normalized}$ is the normalized fluorescence intensity,

FI_x is the fluorescence intensity after each mole fraction of Fragment 7 added**,**

$FI_{initial}$ is the fluorescence intensity of free DNA,

FI_{max} is the maximum fluorescence intensity observed during the experiment for a certain mole fraction of fragment 7 added (in this case 0.69).

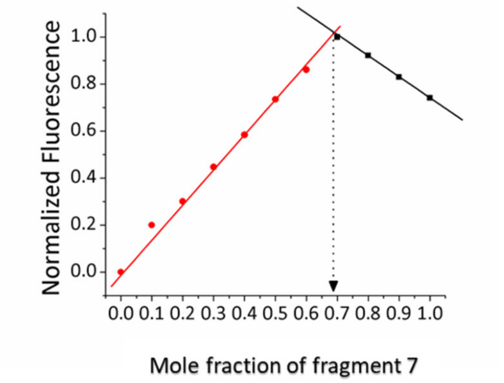

Figure S8. Binding Stoichiometry of Fragment: DNA (2:1) with Job Plot analysis

Data analysis of FAM Measurements: The K_D dissociation constant calculated by a binding isotherm equation using nonlinear regression. Equation 4 as follows:

Equation 4…

$$\mathbf{Y} = \frac{\mathbf{Y_0 + a\,[L]}}{\{\mathbf{K_D + [L]}\}}$$

Here, Y is the fluorescence intensity measured at ligand concentration [L], Y_o is the fluorescence intensity when [L] = 0, and Y_o + a is the fluorescence intensity at saturation.

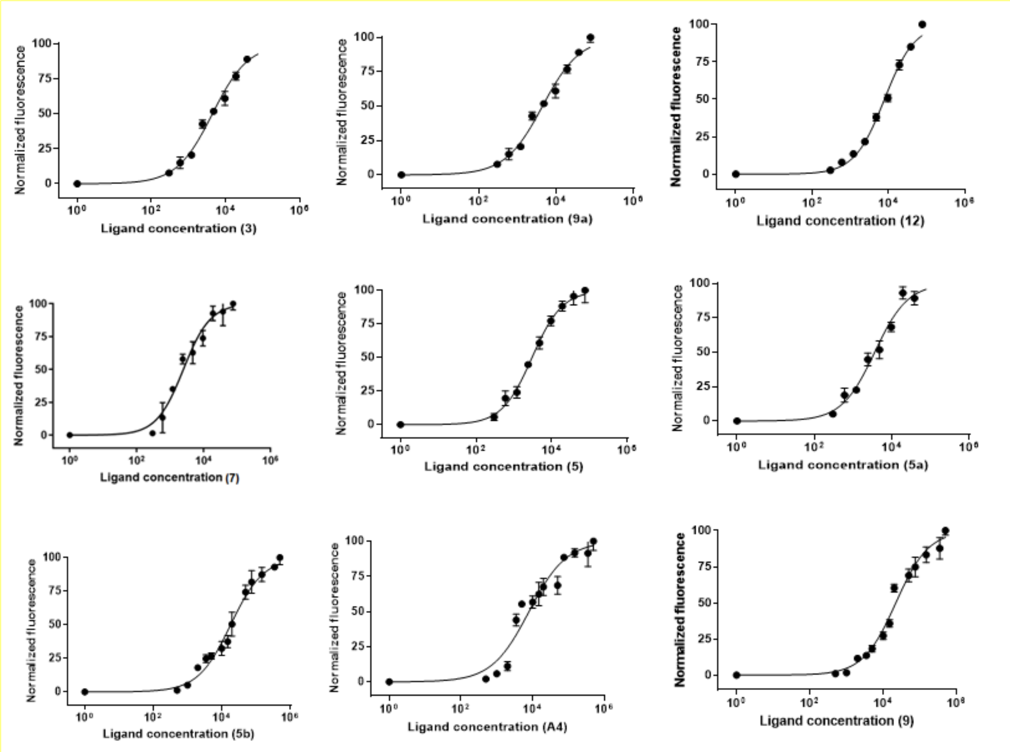

Figure S9. Dose response curves based on FAM titration Ligands: c-Myc; Buffer conditions10 mM Tris-HCl buffer, pH 7.4, in the presence of 150 mM KCl

In vitro investigations

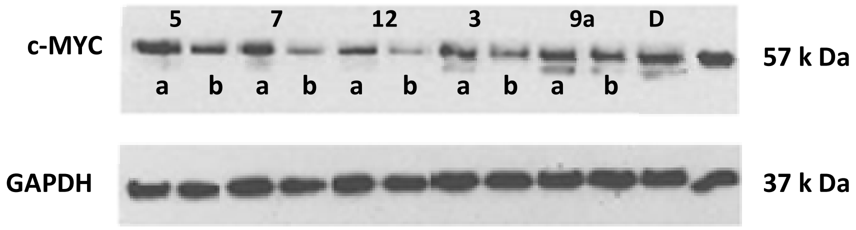

Figure S10: Western plot analysis of protein expression upon treatment with the respective ligand at 3µM (a) and 10µM.

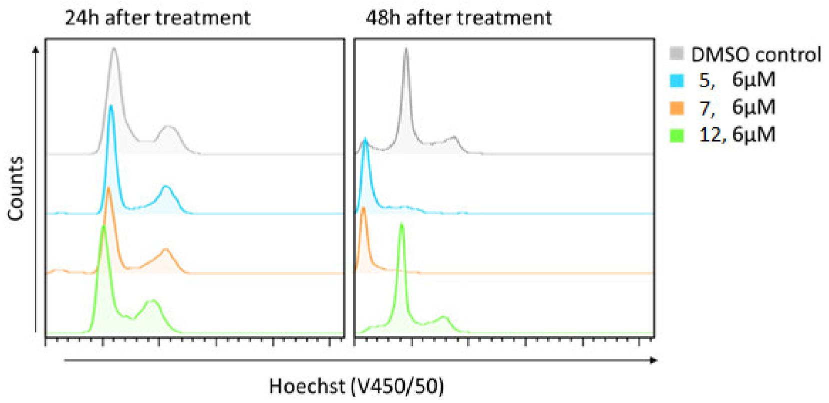

Figure S11: Cell cycle, depicted as Hoechst staining, all cells were gated

Table S3: Effect of Fragments 5, 7 and 12 on cell cycle after 24 h

Cell cycle analysis after 24 h				
	**Sub-G1**	**G1**	**G2/M**	**S-phase**
DMSO-Control	0.331 %	66.0 %	23 %	9.25 %
Fragment 5	2.27 % ↑	70.4 % ↑	19.5 % ↓	6.53 % ↓
Fragment 7	7.34 % ↑	66.2 % ↑	18.1 % ↓	7.12 % ↓
Fragment 12	0.739 % ↑	71.6 % ↑	9.57 % ↓	17.4 % ↑

Table S4: Effect of Fragments 5, 7 and 12 on cell cycle after 48 h

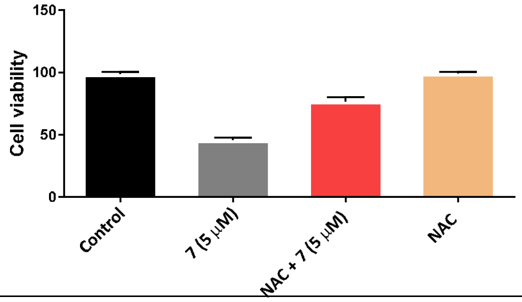

Cell cycle analysis after 48 h				
	Sub-G1	G1	G2/M	S-phase
DMSO-Control	10.8 %	61.7 %	18.8 %	7.23 %
5	70.9 % ↑	21.1 % ↓	4.05 % ↓	2.83 % ↓
7	85.7 % ↑	9.82 % ↓	1.79 % ↓	0.893 % ↓
12	8.05 % ↓	72.5 % ↑	11.9 % ↓	6.78 % ↓

Figure S12: ROS scavenger-NAC reversed compound 7 inhibited cell proliferation and compound 7 induced intracellular ROS production in Hela cancer cells.

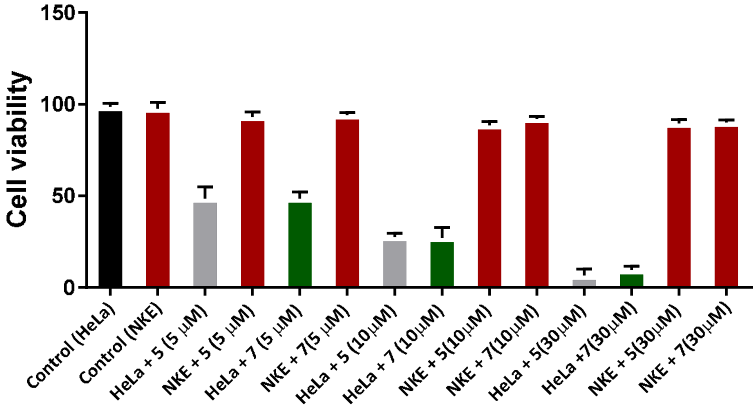

Figure S13: Cell viability with Normal Kidney (NKE) cells for compound 5 and 7

Drug Likeness properties of fragments:

The physico-chemical properties of all compounds were assessed by using the online Swiss-ADME web tool[1] (Figure S2, Table S3, S4 & S5). Based on Lipinski's rule of five,[2] used as a requirement for drug-like properties to predict oral bioavailability (O-B-A), the molecular property of a compound is described in order to estimate its pharmacokinetic parameters in the human body, including its absorption, distribution, metabolism and excretion.

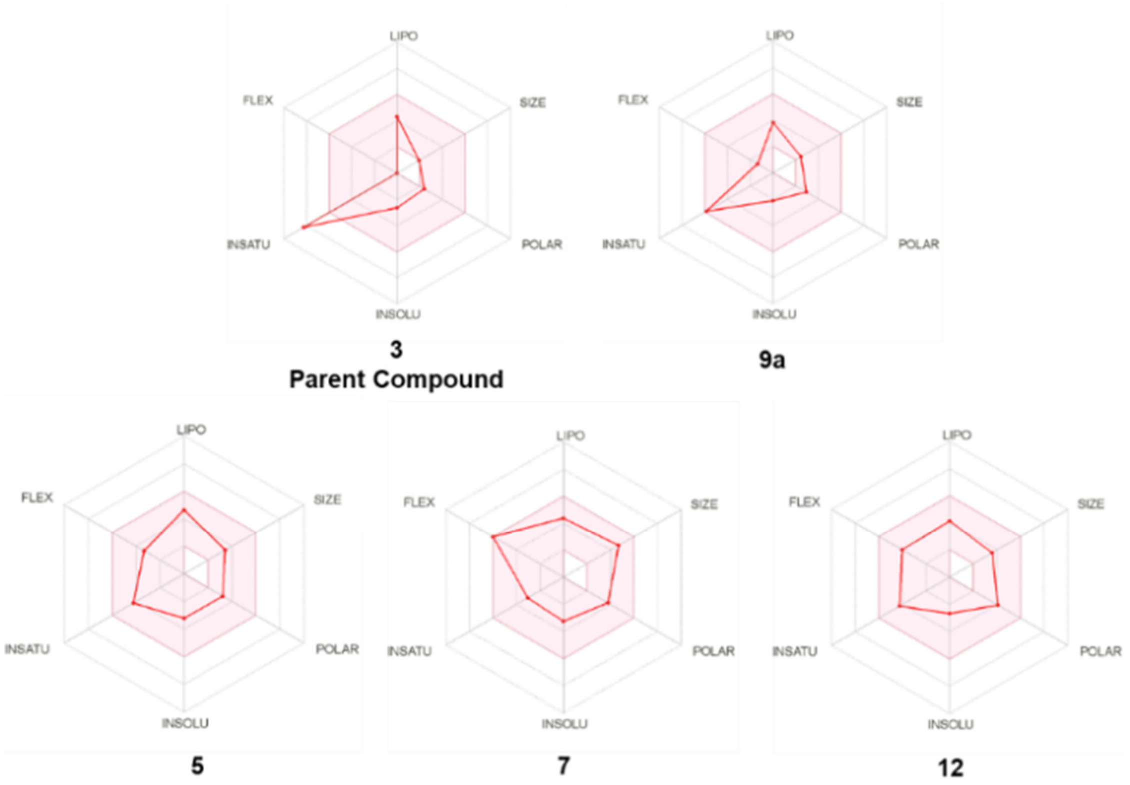

Figure S14. SWISS ADME Radar of 9a and novel substituted 5-nitro indole derivatives 12, 7 & 5. Light red regions indicate preferred regions using the following borders: LIPO (Lipophilicity): -0.7 < XLOGP3 < +5.0; SIZE: 150 g/mol < MW < 500 g/mol; POLAR (Polarity): 20 $Å^2$ < TPSA < 130 $Å^2$; INSOL (Insolubility): 0 < LOGS (ESOL) < 6; INSATU (IN saturation): 0.25 < Fraction CSP^3 < 1; FLEX (Flexibility): 0 < Number of rotable Bonds < 9.

For most 'drug-like' molecules all together six physicochemical properties are taken into account for prediction of oral bioavailability radar, these are good permeability across the cell membrane (LIPO), solubility in aqueous media (INSOL), the saturation character (INSATU), the molecular flexibility as function of number of hydrogen bond acceptors (FLEX), a molecular weight below 500 (SIZE) a polar surface area characterizing drug absorption (POLAR) and less than 5 hydrogen bond donors. [3] Molecules violating more than one of these rules decrease the activity and selectivity of a likely drug candidate and therefore make it unlikely to be orally active in humans. Figure 2 shows results of the best lead compounds (**5**, **7**, **9a** and **12**) and the parent compound **3**.[4] While the ADME radar and the drug-likeliness parameters are not optimal for **3**, the values for the developed compounds fit into the allowed region of the ADME radar. The miLog *P* value of all fragments was found to be < 5 (3.88), suggesting that these molecules have a good permeability across the cell membrane. The molecular weights of all selected fragments are less than 500 g/mol, on average 234.34 g/mol. The number of hydrogen bond acceptors (O and N atoms) (NOHNH=1) and number of hydrogen bond donors (NH and OH; *n*ON=2) of the best fragments were found to be less than 5, respectively, and were in good agreement with the Lipinski's rule of five. The above-determined molecular properties of all the fragments justify their use as a possible screening candidate for high throughput fluorescence intercalator displacement (FID) assays.[5]

Table S5: Physiochemical Properties of Fragments (SWISS-ADME-Filters)

	Formula	MW	#Heavy atoms	#Aromatic heavy atoms	Fraction Csp3	#Rotatable bonds	#H-bond acceptors	#H-bond donors	MR	TPSA
A1	$C_9H_{10}N_2$	146.19	11	9	0.11	0	0	1	47.6	30.95
A2	$C_9H_{10}N_2$	146.02	11	9	0.11	0	0	2	47.67	41.81
A3	$C_8H_8N_2$	132.16	10	9	0	0	0	2	42.7	41.81
A4	$C_{10}H_{12}N_2$	160.22	12	9	0.2	0	0	1	52.57	30.95
A5	$C_{12}H_{16}N_{2O}$	204.27	15	9	0.33	3	1	1	63.27	40.18
A6	$C_{15}H_{14}N_2$	222.29	17	15	0.07	2	0	1	72.09	30.95
A7	$C_{10}H_{13}N_3$	175.23	13	9	0.3	1	1	1	55.17	43.84
A8	$C_8H_8N_2$	132.16	10	9	0	0	0	2	42.7	41.81
A9	$C_{14}H_{13}N_3O$	239.27	18	15	0.07	2	2	2	71.91	64.07
A10	$C_9H_{11}N_3$	161.2	12	9	0.11	1	1	3	50.38	67.83
A11	$C_8H_9N_3$	147.18	11	9	0.12	0	1	1	45.4	43.84
A12	$C_9H_{10}N_2$	146.19	11	9	0.11	0	0	1	47.6	30.95
A13	$C_{17}H_{16}N_2$	248.32	19	9	0.18	2	0	2	82.79	27.82
A14	$C_{12}H_{17}N_3$	203.28	15	9	0.33	2	1	2	65.05	56.97
A15	$C_8H_6N_2O$	146.15	11	9	0	1	2	1	41.48	45.75
A16	$C_{15}H_{17}N_3S$	271.38	19	14	0.27	2	1	1	82.66	72.08
A17	$C_{11}H_{12}N_2O$	188.23	14	9	0.18	1	1	1	57.96	48.02
A18	$C_{14}H_{14}C_lN_3S$	291.8	19	14	0.21	2	1	1	82.71	72.08
A19	$C_{11}H_{11}NO$	173.21	13	9	0.18	1	1	0	53.55	22
A20	$C_{10}H_{10}N_2O_2$	190.2	14	9	0.1	1	2	2	54.56	68.25
A21	$C_{11}H_{12}N_2O_2$	204.23	15	9	0.18	2	2	2	59.15	68.25
A22	$C_{13}H_{17}N_3$	215.29	16	9	0.38	1	1	2	71.44	42.98
A23	$C_{14}H_{11}N_3O_3$	239.27	18	15	0.07	2	2	2	71.91	64.07
A24	$C_{10}H_{13}N_3$	175.11	12	9	0.11	1	1	3	50.38	67.83
A25	$C_8H_7N_3O_2$	147.18	11	9	0.12	0	1	1	45.4	43.84
A26	$C_8H_9N_3$	147.18	11	9	0.12	0	1	1	45.4	43.84
A27	$C_{17}H_{16}N_2$	248.32	19	9	0.18	2	0	2	82.79	27.82
A28	$C_{12}H_{17}N_3$	203.28	15	9	0.33	2	1	2	65.05	56.97
A29	$C_7H_6N_2$	118.14	9	9	0	0	1	0	35.19	17.3
A30	$C_{10}H_{10}N_2O$	174.2	13	9	0.1	1	1	1	52.99	48.02
A31	$C_{14}H_{14}ClN_3S$	291.8	19	14	0.21	2	1	1	82.71	72.08
A32	$C_9H_8N_2O$	160.17	12	9	0	1	1	2	48.09	58.88
A33	$C_{10}H_{10}N_2O_2$	190.2	14	9	0.1	1	2	2	54.56	68.25
A34	$C_{11}H_{12}N_2O_2$	204.23	15	9	0.18	2	2	2	59.15	68.25
A35	$C_{19}H_{22}N_2O$	294.39	22	15	0.26	5	2	0	91.65	17.4
A36	$C_8H_6N_2O$	146.15	11	9	0	1	2	0	40.58	34.37
A37	$C_{18}H_{16}N_4O_4$	352.34	26	9	0.22	5	4	0	104.55	99.81
A38	$C_4H_7N_5$	125.13	9	6	0	0	2	3	35.25	103.84
A39	$C_5H_6N_2O_2$	126.11	9	6	0.2	0	2	2	32.65	65.72
A40	$C_4H_4N_2O_2$	112.09	8	6	0	0	2	2	27.68	65.72
A41	$C_4H_4N_2O_3$	128.09	9	6	0	0	3	3	29.71	85.95
A42	$C_{10}H_8N_2O_2$	188.18	14	12	0	1	4	2	51.51	66.24
A43	$C_4H_5N_3O$	111.1	8	6	0	0	3	2	28.46	72.03
A44	C_4H_9N	71.12	5	0	1	0	1	1	25.94	12.03
A45	$C_7H_{17}NO_5S$	227.28	14	0	1	4	6	2	55.13	84.45
A46	$C_{10}H_{14}N_2O$	178.23	13	6	0.4	1	1	1	56.57	38.49
A47	$C_6H_{12}ClNO$	149.62	9	0	1	2	2	0	41.53	12.47
A48	C_5H_9NO	99.13	7	0	0.8	0	1	0	31.05	20.31
A49	$C_{13}H_{18}N_2O$	218.29	16	6	0.46	4	2	1	69.65	32.34
A50	$C_6H_{14}N_2$	114.19	8	0	1	2	2	1	38.36	29.26
A51	$C_9H_8N_2O_7$	256.17	18	0	0.44	4	7	0	58.33	110.29
A52	$C_{11}H_8O_2$	172.18	13	6	0.09	0	2	0	49.05	34.14
A53	$C_{10}H_6O_2$	158.15	12	6	0	0	2	0	44.24	34.14
A54	$C_{10}H_6O_3$	174.15	13	6	0	0	3	1	45.81	54.37
A55	$C_{31}H_{46}O_2$	450.7	33	6	0.61	14	2	0	144.72	34.14
A56	$C_{15}H_{10}O_3$	238.24	18	12	0.07	1	3	1	65.88	54.37
A57	$C_{14}H_8O_4$	240.21	18	12	0	0	4	2	63.8	74.6
A58	$C_{14}H_{12}N_2O$	224.26	17	15	0.07	3	2	1	67.07	37.91
A59	$C_8H_6N_2O$	146.15	11	9	0	1	2	1	41.48	45.75
A60	$C_{14}H_{17}N$	199.29	15	6	0.43	1	1	0	67.92	3.24
A61	$C_{12}H_{16}N_2O$	204.27	15	6	0.42	3	1	1	65.07	32.34
A62	$C_{12}H_{16}N_2S$	220.33	15	6	0.42	3	0	1	72.27	47.36
A63	$C_{12}H_{17}N$	175.27	13	6	0.5	2	1	0	60.14	3.24
A64	$C_{11}H_{15}NO$	177.24	13	6	0.45	2	2	1	56.76	21.26
A65	$C_{12}H_{17}NO$	191.27	14	6	0.5	2	2	0	61.67	12.47
A66	$C_{11}H_{14}FNO$	195.23	14	6	0.45	2	3	1	56.72	21.26
A67	$C_{12}H_{17}NO_2S$	239.33	16	6	0.5	2	3	1	68.33	54.55
A68	$C_7H_{15}NO_4S$	209.26	13	0	1	4	5	1	52.08	75.22
A69	$C_{13}H_{19}NO$	205.3	15	6	0.54	3	2	1	66.11	23.47
A70	C_7H_9N	107.15	8	6	0.14	1	1	1	34.12	26.02
A71	C_7H_8FN	125.14	9	6	0.14	1	2	1	34.07	26.02
A72	$C_8H_8F_3N$	175.15	12	6	0.25	2	4	1	39.12	26.02
A73	$C_8H_{11}NO$	137.18	10	6	0.25	2	2	1	40.61	35.25
A74	$C_{10}H_{15}NO_3$	197.23	14	6	0.4	4	4	1	53.59	53.71
A75	$C_{10}H_{15}NO_2$	181.23	13	6	0.4	4	3	1	52	30.49
A76	$C_{14}H_{15}N$	197.28	15	12	0.14	4	1	1	63.5	12.03
A77	$C_7H_8N_2O_2$	152.15	11	6	0.14	2	3	1	42.94	71.84

A78	$C_8H_{10}BrN$	200.08	10	6	0.25	1	1	1	46.62	26.02
A79	$C_9H_9BrN_2O_3$	273.08	15	6	0.22	5	3	1	60.72	74.92
A80	$C_7H_5NO_3$	151.12	11	6	0	2	3	0	40.65	62.89
A81	$C_9H_9NO_5$	211.17	15	6	0.22	4	5	0	53.64	81.35
A82	$C_8H_7NO_4$	181.15	13	6	0.12	3	4	0	47.14	72.12
A83	$C_8H_7NO_4$	181.15	13	6	0.12	3	4	0	47.14	72.12
A84	$C_9H_{10}N_2O_3$	194.19	14	6	0.22	3	3	0	54.86	66.13
A85	$C_7H_4FNO_3$	169.11	12	6	0	2	4	0	40.61	62.89
A86	C_7H_8O	108.14	8	6	0.14	1	1	1	32.57	20.23
A87	$C_8H_{10}O$	122.16	9	6	0.25	1	1	1	37.38	20.23
A88	C_7H_8S	124.2	8	6	0.14	1	0	0	39.34	38.8
A89	$C_7H_8O_2$	124.14	9	6	0.14	1	2	1	34.96	29.46
A90	$C_8H_{10}O_2$	138.16	10	6	0.25	2	2	1	39.06	29.46
A91	$C_9H_{11}NO_5$	213.19	15	6	0.33	4	5	1	54.38	84.51
A92	$C_9H_{12}O_3$	168.19	12	6	0.33	3	3	1	45.55	38.69
A93	$C_7H_7NO_4$	169.13	12	6	0.14	2	4	2	43.41	86.28
A94	$C_7H_7NO_3$	153.14	11	6	0.14	2	3	1	41.39	66.05
A95	$C_8H_7NO_3$	165.15	12	6	0.12	2	3	0	45.46	62.89
A96	$C_{10}H_{12}O_3$	180.2	13	6	0.3	3	3	0	49.62	35.53
A97	$C_{12}H_{17}NO$	191.27	14	6	0.42	4	1	0	60.46	20.31
A98	$C_{10}H_{12}O_2$	164.2	12	6	0.3	2	2	0	48.09	26.3
A99	$C_{10}H_8ClNO_2S$	241.69	15	10	0.1	1	3	0	59.79	55.41
A100	$C_9H_7ClO_2S_2$	246.73	14	9	0.11	1	2	0	59.87	70.76
A101	$C_7H_8N_4$	148.17	11	9	0.29	0	3	0	40.71	43.08
A102	$C_{13}H_{11}ClO_2S$	266.74	17	12	0.08	2	2	0	69.93	42.52
A103	$C_7H_5ClO_4S$	220.63	13	6	0.14	1	4	0	45.59	60.98
A104	$C_{13}H_{19}ClO_3S$	290.81	18	0	0.69	1	3	0	74.31	51.75
A105	$C_{13}H_{19}ClO_3S$	290.81	18	0	0.69	1	3	0	74.31	51.75
A106	$C_8H_6BrClO_3S$	297.55	14	6	0.25	1	3	0	56.57	51.75
A107	$C_{10}H_{10}ClNO_2S$	243.71	15	9	0.2	1	2	0	61.25	47.45
A108	C_8H_7N	117.15	9	9	0	0	0	1	38.3	15.79
A109	$C_{15}H_{23}N_3$	245.36	18	9	0.47	7	2	2	77.8	31.06
A110	$C_{11}H_{14}N_2$	174.24	13	9	0.27	2	1	1	55.77	19.03
A111	$C_{13}H_{19}N_3$	217.31	16	9	0.38	5	2	2	68.19	31.06
A112	$C_{13}H_{19}N_3$	217.6	14	9	0.12	1	3	0	57.67	83.65
A113	$C_{19}H_{18}N_2$	259.71	16	6	0.3	2	3	0	64.53	62.83
A114	$C_{14}H_{14}ClN_3O_2S$	323.8	21	12	0.14	4	4	0	84.35	70.48
A115	$C_{11}H_{11}ClN_2O_2S$	270.74	17	11	0.18	2	3	0	66.58	60.34
A116	$C_{11}H_{12}ClNO_3S$	273.74	17	6	0.36	3	3	0	69.33	62.83
A117	$C_9H_8ClNO_4S$	261.68	16	6	0.22	2	4	1	61.68	83.06
A118	$C_{12}H_{14}ClNO_3S$	287.76	18	6	0.42	4	3	0	74.14	62.83
A119	$C_{15}H_{24}N_4$	260.38	19	9	0.47	7	2	3	82.21	57.08
A120	$C_{17}H_{30}N_6$	318.46	23	9	0.53	13	5	6	95.63	89.93
A121	$C_{15}H_{25}N_5$	275.39	20	9	0.47	10	4	5	83.22	77.9
A122	$C_{16}H_{29}N_5O_2$	323.43	23	6	0.62	13	7	5	89.84	92.6
A123	$C_{14}H_{20}N_4$	244.34	18	9	0.5	5	3	2	77.4	43.95
A124	$C_{14}H_{22}N_4$	246.35	18	9	0.5	7	3	2	75.6	43.95
A125	$C_{14}H_{20}N_4$	244.34	18	9	0.5	5	3	2	77.4	43.95
A126	$C_{10}H_{13}NO_2$	179.22	13	6	0.4	2	3	0	49.98	21.7
A127	$C_{12}H_{18}N_2O_2$	222.28	16	6	0.5	5	4	1	62.4	33.73
A128	$C_{10}H_{13}NO_2$	179.09	18	9	0.47	7	2	2	77.8	31.06
A129	$C_{14}H_{22}N_2O_2$	250.17	16	9	0.38	5	2	2	68.19	31.06
24C	$C_{18}H_{20}N_2O$	280.36	21	15	0.22	5	2	1	86.75	28.26
2A	$C_8H_6N_2O$	146.15	7	5	0	1	1	1	26.18	32.86
19B	$C_{15}H_{17}N_3S$	271.38	19	14	0.27	2	1	1	82.66	72.08
8	$C_9H_{10}N_2$	146.19	11	9	0.11	0	0	1	47.6	30.95
9	$C_{11}H_{13}N_3O_2$	219.24	16	9	0.27	3	3	1	64.6	64.85
10	$C_{12}H_{16}N_4O_2$	248.28	18	9	0.33	5	4	2	72.21	76.88
12	$C_{15}H_{20}N_4O_2$	288.34	21	9	0.47	6	4	2	88.43	76.88
11	$C_{15}H_{22}N_4O_2$	290.36	21	9	0.47	8	4	2	86.63	76.88
7	$C_{21}H_{31}N_5O_2$	385.5	28	9	0.62	9	5	1	122.06	69.26
17A	$C_{19}H_{18}N_4O_4$	366.37	27	9	0.26	6	4	0	109.36	99.81
5	$C_{15}H_{19}N_3O_2$	273.33	20	9	0.47	5	3	0	85.56	53.99
19A	$C_{11}H_8N_2O_2$	200.19	15	9	0.09	2	2	0	59.8	50.75
20	$C_{12}H_8N_2O_3$	228.2	17	9	0.08	3	3	0	65.19	67.82
21A	$C_{14}H_{15}N_3O_2$	257.29	19	9	0.29	4	3	0	77.27	53.99
4B	$C_{10}H_9BrN_2O_2$	269.09	15	9	0.2	3	2	0	64.7	50.75
4A	$C_{11}H_{11}BrN_2O_2$	283.12	16	9	0.27	4	2	0	69.51	50.75
14	$C_{11}H_{12}N_5O_2$	246.25	18	9	0.27	5	5	1	68.53	86.96
21B	$C_{18}H_{24}N_4O_2$	328.41	24	9	0.44	9	4	1	99.3	66.02
16	$C_{14}H_{18}N_6O_2$	302.33	22	9	0.43	7	6	0	83.93	103.74
6	$C_{16}H_{19}N_3O_3$	301.34	22	9	0.44	6	4	0	90.95	71.06
15	$C_{12}H_{11}N_5O_3$	273.25	20	9	0.25	6	6	0	71.84	117.57
16A	$C_4H_6N_4O$	126.12	9	6	0	0	3	3	32.86	98.05

23A	$C_{10}H_{13}N_3$	175.23	13	9	0.3	2	2	1	53.57	31.92
26B	$C_9H_{10}BrNO$	228.09	12	6	0.22	4	1	1	51.89	29.1
27B	C_9H_9BrFNO	246.08	13	6	0.22	4	2	1	51.85	29.1
30B	$C_{10}H_{12}BrNO_2$	258.11	14	6	0.3	5	2	1	58.39	38.33
29B	$C_{12}H_{16}BrNO_4$	318.16	18	6	0.42	7	4	1	71.37	56.79
28B	$C_{10}H_9BrF_3NO$	296.08	16	6	0.3	5	4	1	56.9	29.1
26D	$C_{18}H_{19}N_3O_3$	325.36	24	12	0.28	5	4	1	94.37	85.52
27D	$C_{18}H_{18}FN_3O_3$	343.35	25	12	0.28	5	5	1	94.33	85.52
28D	$C_{19}H_{18}F_3N_3O_3$	393.36	28	12	0.32	6	7	1	99.38	85.52
29D	$C_{21}H_{25}N_3O_6$	415.44	30	12	0.38	8	7	1	113.85	113.21
30D	$C_{19}H_{21}N_3O_4$	355.39	26	12	0.32	6	5	1	100.87	94.75
26C	$C_{12}H_{11}N_3O$	213.24	16	6	0.17	2	2	1	62.32	70.12
27C	$C_{12}H_{10}FN_3O$	231.23	17	6	0.17	2	3	1	62.28	70.12
28C	$C_{13}H_{10}F_3N_3O$	281.23	20	6	0.23	3	5	1	67.32	70.12
30C	$C_{13}H_{13}N_3O_2$	243.26	18	6	0.23	3	3	1	68.81	79.35
29C	$C_{15}H_{17}N_3O_4$	303.31	22	6	0.33	5	5	1	81.8	97.81
3A	$C_{17}H_{14}N_2$	246.31	19	18	0.06	2	0	2	79.61	31.58
11A	$C_{16}H_{29}N_7$	319.45	23	9	0.56	13	6	6	93.43	102.82
3B	$C_{16}H_{29}N_7$	319.45	23	9	0.56	13	6	6	93.43	102.82
13	$C_8H_6N_2O$	146.15	11	9	0	1	2	1	41.48	45.75
24B	$C_{10}H_{13}N_3$	175.23	13	9	0.3	2	2	1	53.57	31.92
25B	$C_{12}H_{18}N_4$	218.3	16	9	0.42	5	3	2	65.99	43.95
23B	$C_{14}H_{22}N_4$	246.35	18	9	0.5	7	3	2	75.6	43.95
24A	$C_{12}H_{18}N_4$	218.3	16	9	0.42	5	3	2	65.99	43.95
24E	$C_{14}H_{24}N_4O_2$	280.37	20	6	0.57	10	6	4	77.43	80.57
23E	$C_{14}H_{22}N_2O_2$	250.34	18	6	0.57	7	4	1	72.01	33.73
25E	$C_{14}H_{20}N_2O_2$	248.32	18	6	0.57	5	4	1	73.81	33.73
25D	$C_{12}H_{18}N_2O_2$	222.14	13	9	0.27	2	1	1	55.77	19.03
5a	$C_{15}H_{21}N_3$	243.35	18	9	0.47	4	1	1	81.14	34.19
5b	$C_{15}H_{20}N_2$	228.14	17	9	0.47	4	1	0	76.74	8.17
13	$C_8H_6N_2O$	146.15	11	9	0	1	2	1	41.48	45.75
24B	$C_{10}H_{13}N_3$	175.23	13	9	0.3	2	2	1	53.57	31.92
25B	$C_{12}H_{18}N_4$	218.3	16	9	0.42	5	3	2	65.99	43.95
23B	$C_{14}H_{22}N_4$	246.35	18	9	0.5	7	3	2	75.6	43.95
24A	$C_{12}H_{18}N_4$	218.3	16	9	0.42	5	3	2	65.99	43.95
24E	$C_{14}H_{24}N_4O_2$	280.37	20	6	0.57	10	6	4	77.43	80.57
23E	$C_{14}H_{22}N_2O_2$	250.34	18	6	0.57	7	4	1	72.01	33.73
25E	$C_{14}H_{20}N_2O_2$	248.32	18	6	0.57	5	4	1	73.81	33.73
25D	$C_{12}H_{18}N_2O_2$	222.14	13	9	0.27	2	1	1	55.77	19.03
5A	$C_{15}H_{21}N_3$	243.35	18	9	0.47	4	1	1	81.14	34.19
5B	$C_{15}H_{20}N_2$	228.14	17	9	0.47	4	1	0	76.74	8.17

Table S6: Pharmacokinetics, Drug-Likeliness and Medicinal Chemistry (SWISS-ADME-Filters)

	GI absorption	BBB permeant	Pgp substrate	CYP1A2 inhibitor	CYP2C19 inhibitor	CYP2C9 inhibitor	CYP2D6 inhibitor	CYP3A4 inhibitor	log Kp (cm/s)	Lipinski #violations	Lead-likeness #violations	Synthetic Accessibility
A1	High	Yes	No	Yes	No	No	No	No	-5.74	0	1	1.2
A2	High	Yes	No	Yes	No	No	No	No	-5.94	0	1	1.18
A3	High	Yes	No	Yes	No	No	No	No	-6.61	0	1	1.12
A4	High	Yes	No	Yes	No	No	No	No	-5.76	0	1	1.33
A5	High	Yes	No	Yes	No	No	No	No	-6.47	0	1	1.78
A6	High	Yes	Yes	Yes	No	No	No	No	-9.64	0	1	1.52

A7	High	Yes	No	Yes	No	No	No	No	-6.24	0	1	1.84
A8	High	Yes	No	Yes	No	No	No	No	-6.61	0	1	1.12
A9	High	Yes	Yes	Yes	Yes	No	No	No	-6.01	0	1	2.16
A10	High	No	No	Yes	No	No	No	No	-7.12	0	1	1.4
A11	High	Yes	No	Yes	No	No	No	No	-6.66	0	1	1.43
A12	High	Yes	No	Yes	No	No	No	No	-6.25	0	1	1.36
A13	High	Yes	Yes	Yes	Yes	Yes	Yes	No	-5.39	0	1	4.02
A14	High	Yes	Yes	No	No	No	No	No	-6.8	0	1	1.96
A15	High	Yes	No	Yes	No	No	No	No	-6.23	0	1	1.22
A16	High	Yes	Yes	Yes	Yes	Yes	No	Yes	-5.75	0	0	2.68
A17	High	Yes	No	Yes	No	No	No	No	-6.61	0	1	1.57
A18	High	Yes	Yes	Yes	Yes	Yes	No	Yes	-5.47	0	1	2.79
A19	High	Yes	No	Yes	No	No	No	No	-6.06	0	1	1.35
A20	High	Yes	No	No	No	No	No	No	-6.52	0	1	1.47
A21	High	Yes	No	No	No	No	No	No	-6.99	0	1	1.73
A22	High	Yes	Yes	Yes	No	No	No	No	-6.65	0	1	1.7
A23	High	Yes	Yes	Yes	Yes	No	No	No	-6.01	0	1	2.16
A24	High	No	No	Yes	No	No	No	No	-7.12	0	1	1.4
A25	High	Yes	No	Yes	No	No	No	No	-6.66	0	1	1.43
A26	High	Yes	No	Yes	No	No	No	No	-6.66	0	1	1.43
A27	High	Yes	Yes	Yes	Yes	Yes	Yes	No	-5.39	0	1	4.02
A28	High	Yes	Yes	No	No	No	No	No	-6.8	0	1	1.96
A29	High	Yes	No	No	No	No	No	No	-5.71	0	1	1.26
A30	High	Yes	No	Yes	No	No	No	No	-6.81	0	1	1.32
A31	High	Yes	Yes	Yes	Yes	Yes	No	Yes	-5.47	0	1	2.79
A32	High	Yes	No	Yes	No	No	No	No	-6.69	0	1	1.16
A33	High	Yes	No	No	No	No	No	No	-6.52	0	1	1.47
A34	High	Yes	No	No	No	No	No	No	-6.99	0	1	1.73
A35	High	Yes	No	Yes	Yes	No	Yes	Yes	-5.75	0	0	2.34
A36	High	Yes	No	Yes	No	No	No	No	-6.03	0	1	1.37
A37	High	No	No	No	Yes	Yes	No	Yes	-6.28	0	1	4.61
A38	High	No	No	No	No	No	No	No	-7.66	0	1	1.89
A39	High	No	No	No	No	No	No	No	-7.51	0	1	1.48
A40	High	No	No	No	No	No	No	No	-7.74	0	1	1.35
A41	High	No	No	No	No	No	No	No	-8	0	1	1.63
A42	High	Yes	No	Yes	No	No	No	No	-5.89	0	1	1.8
A43	High	No	No	No	No	No	No	No	-7.1	0	1	1.61
A44	Low	No	No	No	No	No	No	No	-6.41	0	1	1
A45	High	No	No	No	No	No	No	No	-10.26	0	1	2.62
A46	High	Yes	Yes	No	No	No	No	No	-6.9	0	1	1.43
A47	Low	No	No	No	No	No	No	No	-6.87	0	1	1.88
A48	Low	No	No	No	No	No	No	No	-7.29	0	1	1
A49	High	Yes	No	No	No	No	Yes	No	-6.28	0	1	1.57
A50	High	No	No	No	No	No	No	No	-6.94	0	1	1
A51	High	No	No	No	No	No	No	No	-8.75	0	0	2.4
A52	High	Yes	No	Yes	No	No	No	No	-5.79	0	1	2.37
A53	High	Yes	No	Yes	No	No	No	No	-6.05	0	1	2.3
A54	High	Yes	No	Yes	No	No	No	No	-6.38	0	1	2.42
A55	Low	No	Yes	Yes	No	No	No	No	-1.3	1	3	5.35
A56	High	Yes	No	Yes	No	No	No	No	-5.84	0	1	2.34
A57	High	Yes	No	Yes	No	No	No	Yes	-5.17	0	2	2.32
A58	High	Yes	Yes	Yes	Yes	No	Yes	Yes	-5.69	0	1	2.19
A59	High	Yes	No	Yes	No	No	No	No	-6.64	0	1	1.18
A60	High	Yes	No	No	No	No	Yes	No	-5.39	0	1	2.36
A61	High	Yes	No	No	No	No	No	No	-6.16	0	1	1.64
A62	High	Yes	No	Yes	Yes	No	Yes	No	-5.83	0	1	1.99
A63	Low	Yes	No	No	No	No	Yes	No	-5.52	0	1	1
A64	High	Yes	No	No	No	No	No	No	-5.95	0	1	1.27
A65	High	Yes	No	No	No	No	Yes	No	-5.71	0	1	1.37
A66	High	Yes	No	No	No	No	Yes	No	-5.99	0	1	1.51
A67	High	Yes	No	No	No	No	No	No	-6.59	0	1	2.21

A68	High	No	No	No	No	No	No	No	-9.81	0	1	2.52
A69	High	Yes	No	No	No	No	Yes	No	-6.07	0	1	1.82
A70	High	Yes	No	Yes	No	No	No	No	-6.18	0	1	1
A71	High	Yes	No	Yes	No	No	No	No	-6.21	0	1	1
A72	High	Yes	No	No	No	No	No	No	-5.96	0	1	1
A73	High	Yes	No	Yes	No	No	No	No	-6.54	0	1	1
A74	High	Yes	No	No	No	No	No	No	-7.28	0	1	1.57
A75	High	Yes	No	Yes	No	No	No	No	-6.79	0	1	1.33
A76	High	Yes	No	Yes	Yes	No	Yes	No	-5.61	0	1	1
A77	High	No	No	No	No	No	No	No	-6.48	0	1	1.37
A78	High	Yes	No	Yes	No	No	No	No	-6.19	0	1	1.56
A79	High	No	No	Yes	Yes	No	No	No	-6.79	0	0	1.73
A80	High	Yes	No	Yes	No	No	No	No	-5.99	0	1	1.44
A81	High	No	No	No	No	No	No	No	-6.76	0	1	2.14
A82	High	No	No	No	No	No	No	No	-6.38	0	1	1.88
A83	High	No	No	No	No	No	No	No	-6.69	0	1	1.8
A84	High	Yes	No	Yes	No	No	No	No	-6.48	0	1	2.11
A85	High	Yes	No	No	No	No	No	No	-6.47	0	1	1.66
A86	High	Yes	No	Yes	No	No	No	No	-6.18	0	1	1
A87	High	Yes	No	Yes	No	No	No	No	-6.04	0	1	1
A88	High	Yes	No	Yes	No	No	No	No	-5.32	0	1	1
A89	High	Yes	No	No	No	No	No	No	-5.94	0	1	1
A90	High	Yes	No	Yes	No	No	No	No	-6.34	0	1	1
A91	High	No	No	No	No	No	No	No	-7.03	0	1	2.28
A92	High	Yes	No	No	No	No	No	No	-6.25	0	1	1.35
A93	High	No	No	No	No	No	No	No	-6.72	0	1	1.63
A94	High	No	No	No	No	No	No	No	-6.35	0	1	1.58
A95	High	Yes	No	Yes	No	No	No	No	-6.4	0	1	1.63
A96	High	Yes	No	Yes	No	No	No	No	-6.38	0	1	1.5
A97	High	Yes	No	Yes	No	No	No	No	-5.46	0	1	1.12
A98	High	Yes	No	Yes	No	No	No	No	-5.55	0	1	1.28
A99	High	Yes	No	Yes	Yes	No	No	No	-6.18	0	1	1.78
A100	High	Yes	No	Yes	Yes	Yes	No	No	-5.21	0	2	2.43
A101	High	Yes	No	Yes	No	No	No	No	-6.56	0	1	2
A102	High	Yes	No	Yes	Yes	Yes	No	No	-5.04	0	1	2.02
A103	High	Yes	No	Yes	No	No	No	No	-6.5	0	1	2.51
A104	High	Yes	No	No	Yes	No	No	No	-6.92	0	0	4.75
A105	High	Yes	No	No	Yes	No	No	No	-6.92	0	0	4.75
A106	High	Yes	No	Yes	Yes	No	No	No	-6.24	0	0	2.41
A107	High	Yes	No	Yes	Yes	No	No	No	-6.07	0	1	1.93
A108	High	Yes	No	Yes	No	No	No	No	-5.56	0	1	1
A109	High	Yes	Yes	Yes	No	No	Yes	No	-6.31	0	1	2
A110	High	Yes	No	Yes	No	No	No	No	-6.1	0	1	1.44
A111	High	Yes	Yes	Yes	No	No	Yes	No	-6.67	0	1	1.79
A112	High	No	No	Yes	Yes	Yes	No	No	-5.82	0	1	2.32
A113	High	Yes	No	Yes	Yes	No	No	No	-6.93	0	0	1.97
A114	High	No	No	Yes	Yes	Yes	No	No	-5.01	0	1	2.81
A115	High	Yes	No	Yes	Yes	Yes	No	No	-6.03	0	0	2.04
A116	High	Yes	No	Yes	Yes	No	No	No	-6.68	0	0	2.09
A117	High	No	No	Yes	No	No	No	No	-6.89	0	0	1.99
A118	High	Yes	No	Yes	Yes	No	No	No	-6.51	0	0	2.17
A119	High	Yes	Yes	No	No	No	No	No	-7.17	0	1	1.97
A120	High	Yes	Yes	No	No	No	Yes	No	-6.82	0	0	2.18
A121	High	No	Yes	No	No	No	Yes	No	-8.89	1	1	2.48
A122	High	No	Yes	No	No	No	No	No	-9.35	1	1	2.56

A123	High	Yes	Yes	Yes	No	No	Yes	No	-6.77	0	1	2.14
A124	High	Yes	Yes	No	No	No	No	No	-6.92	0	1	1.98
A125	High	Yes	Yes	No	No	No	Yes	No	-6.57	0	1	2.19
A126	High	No	Yes	No	No	No	No	No	-8.37	0	1	2.62
A127	High	Yes	No	Yes	No	No	No	No	-6.14	0	1	1.96
A128	High	Yes	No	Yes	No	No	No	No	-6.18	0	1	1
A129	High	Yes	No	Yes	No	No	No	No	-5.32	0	1	1
24C	High	Yes	Yes	Yes	Yes	No	Yes	Yes	-5.63	0	0	2.2
2A	High	Yes	No	No	No	No	No	No	-6.76	0	1	1
19B	High	Yes	Yes	Yes	Yes	Yes	No	Yes	-5.75	0	0	2.68
8	High	Yes	No	Yes	No	No	No	No	-5.74	0	1	1.2
9	High	Yes	No	Yes	No	No	No	No	-6.42	0	1	1.85
10	High	No	No	No	No	No	No	No	-6.83	0	1	2.1
12	High	Yes	Yes	No	No	No	Yes	No	-6.8	0	0	2.22
11	High	Yes	No	Yes	No	No	Yes	No	-6.64	0	1	2.37
7	High	Yes	Yes	No	No	No	Yes	Yes	-7.1	0	2	3.07
17A	High	No	No	No	Yes	Yes	No	Yes	-6.11	0	1	4.7
5	High	Yes	No	Yes	Yes	No	Yes	No	-6.09	0	0	2.14
19A	High	Yes	No	Yes	No	No	No	No	-6.07	0	1	1.81
20	High	Yes	No	Yes	No	No	No	No	-6.62	0	1	1.77
21A	High	Yes	No	Yes	Yes	No	No	No	-6.53	0	0	2.2
4B	High	Yes	No	Yes	Yes	No	No	No	-6.15	0	0	1.89
4A	High	Yes	No	Yes	Yes	No	No	No	-5.98	0	0	2.22
14	High	No	No	Yes	No	Yes	No	No	-5.85	0	1	2.24
21B	High	Yes	No	No	Yes	No	Yes	No	-6.76	0	1	2.7
16	High	No	No	Yes	Yes	Yes	No	No	-6.11	0	0	2.56
6	High	Yes	No	No	Yes	No	No	No	-6.64	0	0	2.19
15	High	No	No	Yes	Yes	No	No	No	-6.2	0	0	2.14
16A	High	No	No	No	No	No	No	No	-7.43	0	1	2.1
23A	High	Yes	No	Yes	No	No	No	No	-6.55	0	1	1.63
26B	High	Yes	No	Yes	No	No	No	No	-6.28	0	1	1.25
27B	High	Yes	No	Yes	No	No	No	No	-6.43	0	1	1.44
30B	High	Yes	No	Yes	No	No	No	No	-6.6	0	0	1.41
29B	High	Yes	No	Yes	Yes	No	No	No	-7.01	0	0	2
28B	High	Yes	No	Yes	Yes	No	No	No	-6.18	0	0	1.61
26D	High	No	Yes	No	Yes	Yes	No	No	-6.72	0	0	2.92
27D	High	No	Yes	No	Yes	Yes	No	No	-6.76	0	0	2.94
28D	High	No	No	No	Yes	Yes	No	Yes	-6.51	0	1	3.07
29D	High	No	Yes	No	Yes	Yes	No	Yes	-7.33	0	2	3.39
30D	High	No	Yes	No	Yes	Yes	No	Yes	-6.93	0	1	3.04
26C	High	No	No	Yes	No	No	No	No	-7	0	1	2.56
27C	High	No	No	Yes	No	No	No	No	-7.04	0	1	2.58
28C	High	Yes	No	Yes	Yes	No	No	No	-6.79	0	0	2.72
30C	High	No	No	Yes	No	No	No	No	-7.21	0	1	2.58
29C	High	No	Yes	No	No	No	No	No	-7.62	0	0	2.94
11A	High	No	Yes	No	No	No	Yes	No	-8.32	0	1	2.19
3B	High	No	Yes	No	No	No	No	No	-8.93	0	1	2.92
13	High	No	Yes	No	No	No	No	No	-9.15	1	1	2.66
24B	High	Yes	No	Yes	No	No	No	No	-6.64	0	1	1.18
25B	High	Yes	No	Yes	No	No	No	No	-6.55	0	1	1.63
23B	High	Yes	Yes	Yes	No	No	No	No	-7.12	0	1	1.93
24A	High	Yes	Yes	Yes	No	No	Yes	No	-6.93	0	1	2.01

24E	High	Yes	Yes	No	No	No	Yes	No	-6.74	0	1	2.05
23E	High	Yes	Yes	No	No	No	No	No	-6.7	0	1	2.24
25E	High	Yes	Yes	No	No	No	Yes	No	-6.35	0	1	2.24
25D	High	Yes	No	Yes	No	No	No	No	-6.04	0	1	1
5A	High	Yes	No	Yes	No	No	No	No	-6.4	0	1	1.63
5B	High	Yes	No	Yes	No	No	No	No	-6.38	0	1	1.5
24A	High	Yes	Yes	Yes	No	No	Yes	No	-6.93	0	1	2.01
24E	High	Yes	Yes	No	No	No	Yes	No	-6.74	0	1	2.05
23E	High	Yes	Yes	No	No	No	No	No	-6.7	0	1	2.24
25E	High	Yes	Yes	No	No	No	Yes	No	-6.35	0	1	2.24
25d	High	Yes	No	Yes	No	No	No	No	-6.04	0	1	1
5A	High	Yes	No	Yes	No	No	No	No	-6.4	0	1	1.63
5B	High	Yes	No	Yes	No	No	No	No	-6.38	0	1	1.5

Table S7: Lipophilicity and Water Solubility Properties of Fragments (SWISS-ADME-Filters)

	iLogP	xLogP3	WLogP	MLOGP	ESOL Class	Ali LogS	Ali Solubility (mol/l)	Ali Class
A1	1.58	2.04	1.77	1.23	Soluble	-2.32	4.81E-03	Soluble
A2	1.37	1.77	2.07	1.23	Soluble	-2.27	5.42E-03	Soluble
A3	1.08	0.7	1.76	0.91	Very soluble	-1.16	6.99E-02	Very soluble
A4	1.79	2.14	2.08	1.53	Soluble	-2.42	3.79E-03	Soluble
A5	2.26	1.51	2.19	1.22	Soluble	-1.96	1.09E-02	Very soluble
A6	2.25	-2.79	3.28	2.66	Highly soluble	2.69	4.94E+02	Highly soluble
A7	1.61	1.59	1.73	1.33	Soluble	-2.12	7.55E-03	Soluble
A8	1.08	0.7	1.76	0.91	Very soluble	-1.16	6.99E-02	Very soluble
A9	1.89	2.46	2.38	2.42	Soluble	-3.45	3.55E-04	Soluble
A10	0.83	0.23	1.06	0.35	Very soluble	-1.21	6.11E-02	Very soluble
A11	1.17	0.76	1.16	0.7	Very soluble	-1.26	5.49E-02	Very soluble
A12	1.59	1.32	1.77	1.23	Soluble	-1.57	2.69E-02	Very soluble
A13	2.2	3.42	2.93	2.88	Soluble	-3.68	2.07E-04	Soluble
A14	1.83	1.04	1.58	1.22	Soluble	-1.83	1.49E-02	Very soluble
A15	0.5	1.35	1.38	0.49	Soluble	-1.91	1.22E-02	Very soluble
A16	2.4	3.11	3.43	1.81	Soluble	-4.29	5.10E-05	Moderately soluble
A17	1.49	1.18	1.89	0.86	Soluble	-1.78	1.64E-02	Very soluble
A18	2.58	3.67	3.78	2.08	Moderately soluble	-4.87	1.34E-05	Moderately soluble
A19	1.97	1.83	2.3	1.47	Soluble	-1.91	1.22E-02	Very soluble
A20	0.83	1.32	1.47	0.8	Soluble	-2.35	4.42E-03	Soluble
A21	1.2	0.78	1.4	0.82	Very soluble	-1.79	1.61E-02	Very soluble
A22	2.05	1.35	1.78	1.5	Soluble	-1.85	1.40E-02	Very soluble
A23	1.89	2.46	2.38	2.42	Soluble	-3.45	3.55E-04	Soluble
A24	0.83	0.23	1.06	0.35	Very soluble	-1.21	6.11E-02	Very soluble
A25	1.17	0.76	1.16	0.7	Very soluble	-1.26	5.49E-02	Very soluble
A26	1.17	0.76	1.16	0.7	Very soluble	-1.26	5.49E-02	Very soluble
A27	2.2	3.42	2.93	2.88	Soluble	-3.68	2.07E-04	Soluble
A28	1.83	1.04	1.58	1.22	Soluble	-1.83	1.49E-02	Very soluble
A29	1.63	1.85	1.33	0.72	Soluble	-1.83	1.46E-02	Very soluble
A30	1.36	0.78	1.58	0.57	Very soluble	-1.37	4.28E-02	Very soluble
A31	2.58	3.67	3.78	2.08	Moderately soluble	-4.87	1.34E-05	Moderately soluble
A32	0.83	0.83	1.57	0.27	Very soluble	-1.65	2.24E-02	Very soluble
A33	0.83	1.32	1.47	0.8	Soluble	-2.35	4.42E-03	Soluble
A34	1.2	0.78	1.4	0.82	Very soluble	-1.79	1.61E-02	Very soluble
A35	3.47	3.3	3.52	2.74	Soluble	-3.34	4.56E-04	Soluble
A36	1.03	1.64	1.15	0.09	Soluble	-1.97	1.06E-02	Very soluble
A37	2.29	3.05	3.76	1.52	Soluble	-4.81	1.54E-05	Moderately soluble
A38	0.41	-0.84	-0.75	-1.06	Very soluble	-0.86	1.38E-01	Very soluble
A39	0.71	-0.62	-0.63	-0.39	Very soluble	-0.29	5.15E-01	Very soluble
A40	0.52	-1.07	-0.94	-0.8	Very soluble	0.18	1.51E+00	Highly soluble
A41	0.19	-1.29	-1.23	-0.96	Very soluble	-0.02	9.60E-01	Very soluble
A42	1.92	2.2	1.55	1.2	Soluble	-3.23	5.95E-04	Soluble
A43	0.76	-0.17	-0.23	-1.31	Very soluble	-0.89	1.30E-01	Very soluble
A44	1.51	0.46	-0.01	0.35	Very soluble	-0.28	5.23E-01	Very soluble
A45	0.74	-3.62	0.23	-1.43	Highly soluble	2.43	2.70E+02	Highly soluble
A46	1.71	0.68	0.73	0.83	Very soluble	-1.07	8.61E-02	Very soluble
A47	2.11	0.48	0.18	0.57	Very soluble	-0.31	4.88E-01	Very soluble
A48	1.48	-0.54	-0.14	0.1	Very soluble	0.58	3.83E+00	Highly soluble

A49	2.58	1.9	1.46	1.72	Soluble	-2.2	6.28E-03	Soluble
A50	1.71	0.08	-0.34	0.21	Very soluble	-0.25	5.64E-01	Very soluble
A51	1.19	-1.25	-1.49	-0.58	Very soluble	-0.57	2.69E-01	Very soluble
A52	1.74	2.2	2.01	1.2	Soluble	-2.55	2.81E-03	Soluble
A53	1.44	1.71	1.62	0.91	Soluble	-2.04	9.07E-03	Soluble
A54	1.4	1.38	1.51	0.03	Soluble	-2.12	7.50E-03	Soluble
A55	5.96	10.91	9.16	5.64	Poorly soluble	-11.59	2.57E-12	Insoluble
A56	1.95	2.69	1.8	1.24	Soluble	-3.48	3.28E-04	Soluble
A57	2.03	3.65	1.87	0.67	Moderately soluble	-4.91	1.24E-05	Moderately soluble
A58	2.3	2.78	2.99	1.93	Soluble	-3.23	5.86E-04	Soluble
A59	0.97	0.77	1.38	0.35	Very soluble	-1.31	4.89E-02	Very soluble
A60	3.09	3	2.22	3.12	Soluble	-2.73	1.85E-03	Soluble
A61	2.38	1.95	2.13	2.26	Soluble	-2.25	5.57E-03	Soluble
A62	2.67	2.55	2.3	2.33	Soluble	-3.19	6.43E-04	Soluble
A63	2.59	2.6	2.14	2.67	Soluble	-2.32	4.82E-03	Soluble
A64	2.3	2.02	1.44	1.73	Soluble	-2.09	8.06E-03	Soluble
A65	2.66	2.48	1.78	2.01	Soluble	-2.39	4.11E-03	Soluble
A66	2.48	2.12	2	2.14	Soluble	-2.2	6.34E-03	Soluble
A67	2.08	1.65	2.22	1.77	Soluble	-2.41	3.90E-03	Soluble
A68	0.84	-3.15	0.3	-0.62	Highly soluble	2.14	1.37E+02	Highly soluble
A69	2.54	2.09	1.3	2.02	Soluble	-2.21	6.13E-03	Soluble
A70	1.43	1.09	0.99	1.54	Very soluble	-1.23	5.91E-02	Very soluble
A71	1.56	1.2	1.55	1.97	Very soluble	-1.34	4.54E-02	Very soluble
A72	1.7	1.98	3.16	2.61	Soluble	-2.15	7.04E-03	Soluble
A73	1.7	0.84	1	1.21	Very soluble	-1.16	6.87E-02	Very soluble
A74	2.14	0.32	1.02	0.64	Very soluble	-1.01	9.75E-02	Very soluble
A75	2.47	0.86	1.27	1.22	Very soluble	-1.08	8.24E-02	Very soluble
A76	2.74	2.67	2.67	3.34	Soluble	-2.57	2.66E-03	Soluble
A77	1.15	1.06	0.9	0.34	Very soluble	-2.16	6.92E-03	Soluble
A78	2.24	1.87	2.14	2.61	Soluble	-2.04	9.16E-03	Soluble
A79	1.46	1.65	1.45	1.75	Soluble	-2.84	1.46E-03	Soluble
A80	0.89	1.74	1.41	1.07	Soluble	-2.68	2.10E-03	Soluble
A81	1.22	1.16	1.42	-0.2	Very soluble	-2.46	3.44E-03	Soluble
A82	1.09	1.45	1.42	0.03	Soluble	-2.57	2.69E-03	Soluble
A83	1.05	1	1.42	0.03	Very soluble	-2.1	7.88E-03	Soluble
A84	1.31	1.41	1.47	0.35	Soluble	-2.4	3.95E-03	Soluble
A85	0.88	1.22	1.97	1.51	Very soluble	-2.14	7.28E-03	Soluble
A86	1.66	1.1	1.03	1.54	Very soluble	-1.12	7.63E-02	Very soluble
A87	1.69	1.42	1.42	1.87	Very soluble	-1.45	3.55E-02	Very soluble
A88	1.79	2.45	1.96	2.61	Soluble	-2.91	1.23E-03	Soluble
A89	1.56	1.58	1.4	1.15	Soluble	-1.81	1.55E-02	Very soluble
A90	1.92	1.13	1.04	1.21	Very soluble	-1.34	4.55E-02	Very soluble
A91	1.61	0.81	0.95	-0.11	Very soluble	-2.17	6.82E-03	Soluble
A92	2.24	1.51	1.04	0.92	Soluble	-1.93	1.17E-02	Very soluble
A93	0.22	0.86	0.64	-0.22	Very soluble	-2.26	5.55E-03	Soluble
A94	0.99	1.24	0.94	0.34	Very soluble	-2.22	5.96E-03	Soluble
A95	1.1	1.28	1.8	0.59	Very soluble	-2.2	6.31E-03	Soluble
A96	2.19	1.44	1.91	1.13	Soluble	-1.79	1.62E-02	Very soluble
A97	2.5	2.83	2.74	2.31	Soluble	-2.91	1.22E-03	Soluble
A98	2.18	2.47	2.21	1.74	Soluble	-2.67	2.15E-03	Soluble
A99	1.95	2.24	3.55	1.55	Soluble	-3.04	9.14E-04	Soluble
A100	2.18	3.66	4.22	2.18	Moderately soluble	-4.84	1.46E-05	Moderately soluble
A101	1.65	0.9	0.74	0.47	Very soluble	-1.39	4.08E-02	Very soluble
A102	2.52	4.06	4.67	3.34	Moderately soluble	-4.66	2.20E-05	Moderately soluble
A103	2.05	1.61	2.42	0.67	Soluble	-2.5	3.14E-03	Soluble
A104	3.12	1.62	4.44	2.12	Soluble	-2.32	4.80E-03	Soluble
A105	3.12	1.62	4.44	2.12	Soluble	-2.32	4.80E-03	Soluble
A106	2.18	2.64	3.39	1.88	Soluble	-3.38	4.19E-04	Soluble
A107	2.17	2.42	3.5	1.59	Soluble	-3.06	8.73E-04	Soluble
A108	1.43	2.05	2.17	1.57	Soluble	-2.01	9.77E-03	Soluble
A109	2.99	2.09	2.45	1.75	Soluble	-2.37	4.24E-03	Soluble
A110	1.88	1.78	2.08	1.55	Soluble	-1.8	1.59E-02	Very soluble
A111	2.53	1.35	1.67	1.23	Soluble	-1.6	2.49E-02	Very soluble
A112	2.02	2.8	3.61	1.27	Soluble	-4.21	6.12E-05	Moderately soluble
A113	1.99	1.34	2.22	1.23	Soluble	-2.26	5.48E-03	Soluble
A114	2.94	4.6	5.18	2.57	Moderately soluble	-5.8	1.57E-06	Moderately soluble
A115	2.33	2.71	3.5	1.8	Soluble	-3.63	2.34E-04	Soluble
A116	2.21	1.81	2.61	1.51	Soluble	-2.75	1.78E-03	Soluble
A117	1.49	1.42	2.35	0.92	Soluble	-2.77	1.70E-03	Soluble
A118	2.38	2.17	3	1.78	Soluble	-3.12	7.54E-04	Soluble
A119	2.14	0.77	1.26	0.64	Very soluble	-1.55	2.83E-02	Very soluble
A120	2.51	1.51	2.04	1.16	Soluble	-2.32	4.82E-03	Soluble
A121	3.11	-0.91	-0.17	-0.25	Very soluble	-0.5	3.20E-01	Very soluble
A122	2.61	-1.55	-0.77	-0.62	Very soluble	-0.1	7.91E-01	Very soluble
A123	2.81	1.45	1.84	1.31	Soluble	-1.98	1.05E-02	Very soluble
A124	2.11	1	1.06	0.92	Very soluble	-1.51	3.08E-02	Very soluble
A125	2.47	1.73	1.84	1.45	Soluble	-2.27	5.38E-03	Soluble
A126	3.01	-0.5	-0.51	-0.15	Very soluble	-0.72	1.89E-01	Very soluble
A127	2.55	1.77	1.32	1.23	Soluble	-1.84	1.43E-02	Very soluble

A128	2.33	2.71	3.5	1.8	Soluble	-3.63	2.34E-04	Soluble
A129	1.49	1.42	2.35	0.92	Soluble	-2.77	1.70E-03	Soluble
24C	3.02	3.35	3.5	2.51	Soluble	-3.62	2.39E-04	Soluble
2A	0.8	0.17	0.83	-0.56	Very soluble	-0.42	3.82E-01	Very soluble
19B	2.4	3.11	3.43	1.81	Soluble	-4.29	5.10E-05	Moderately soluble
8	1.58	2.04	1.77	1.23	Soluble	-2.32	4.81E-03	Soluble
9	1.79	1.71	2.51	0.39	Soluble	-2.69	2.05E-03	Soluble
10	1.69	1.38	2.06	0.26	Soluble	-2.6	2.53E-03	Soluble
12	2.2	1.77	2.25	1.46	Soluble	-3	9.94E-04	Soluble
11	2.68	2.02	2.88	0.65	Soluble	-3.26	5.47E-04	Soluble
7	3.61	2.18	2.44	2.07	Soluble	-3.27	5.40E-04	Soluble
17A	2.62	3.41	4.15	1.75	Moderately soluble	-5.19	6.52E-06	Moderately soluble
5	2.82	2.65	3.18	2.27	Soluble	-3.43	3.67E-04	Soluble
19A	1.96	2.04	2.79	1.95	Soluble	-2.73	1.85E-03	Soluble
20	1.7	1.51	2.6	0.51	Soluble	-2.54	2.87E-03	Soluble
21A	2.63	1.88	2.7	1.13	Soluble	-2.64	2.31E-03	Soluble
4B	1.91	2.52	3.47	1.36	Soluble	-3.23	5.86E-04	Soluble
4A	2.17	2.88	3.86	1.63	Soluble	-3.61	2.48E-04	Soluble
14	2.13	2.75	3.01	0.24	Soluble	-4.23	5.87E-05	Moderately soluble
21B	3.59	2.18	3.07	1.3	Soluble	-3.2	6.31E-04	Soluble
16	2.74	2.86	2.64	0.27	Soluble	-4.7	2.01E-05	Moderately soluble
6	2.55	2.11	2.99	0.82	Soluble	-3.23	5.85E-04	Soluble
15	1.94	2.49	2.54	-0.34	Soluble	-4.6	2.49E-05	Moderately soluble
16A	0.62	-0.51	-0.64	-1.06	Very soluble	-1.08	8.30E-02	Very soluble
23A	1.81	1.15	1.47	1.06	Soluble	-1.41	3.85E-02	Very soluble
26B	1.8	1.99	1.55	2.03	Soluble	-2.23	5.92E-03	Soluble
27B	1.91	1.93	2.11	2.44	Soluble	-2.17	6.84E-03	Soluble
30B	2.2	1.8	1.55	1.7	Soluble	-2.22	5.97E-03	Soluble
29B	2.54	1.74	1.57	1.1	Soluble	-2.55	2.82E-03	Soluble
28B	2.09	2.71	3.72	2.99	Soluble	-2.97	1.06E-03	Soluble
26D	2.55	2.2	1.71	1.9	Soluble	-3.63	2.34E-04	Soluble
27D	2.66	2.3	2.27	2.29	Soluble	-3.73	1.85E-04	Soluble
28D	3.08	3.09	3.88	2.74	Moderately soluble	-4.55	2.80E-05	Moderately soluble
29D	3.01	2.12	1.74	1.03	Soluble	-4.13	7.44E-05	Moderately soluble
30D	2.75	2.17	1.72	1.61	Soluble	-3.79	1.61E-04	Soluble
26C	1.64	0.84	0.58	0.57	Very soluble	-1.9	1.27E-02	Very soluble
27C	1.71	0.94	1.14	0.97	Very soluble	-2	1.00E-02	Very soluble
28C	1.9	1.72	2.75	1.5	Soluble	-2.81	1.55E-03	Soluble
30C	2	0.81	0.59	-0.13	Very soluble	-2.06	8.75E-03	Soluble
29C	2.22	0.75	0.61	-0.69	Soluble	-2.38	4.14E-03	Soluble
3A	1.99	4.1	4.24	3	Moderately soluble	-4.47	3.40E-05	Moderately soluble
11A	2.52	-0.48	0.24	0.07	Very soluble	-0.69	2.05E-01	Very soluble
3B	3.75	-0.92	-0.92	-0.45	Very soluble	-0.54	2.88E-01	Very soluble
13	2.31	-1.27	-0.77	-0.48	Very soluble	-0.39	4.05E-01	Very soluble
24B	0.97	0.77	1.38	0.35	Very soluble	-1.31	4.89E-02	Very soluble
23B	2.38	0.72	1.06	0.78	Very soluble	-1.22	6.01E-02	Very soluble
24A	2.32	1.21	1.22	1.31	Soluble	-1.73	1.86E-02	Very soluble
24E	2.32	1.48	1.22	1.45	Soluble	-2.01	9.77E-03	Soluble
23E	3.03	1.34	0.91	0.96	Soluble	-1.65	2.24E-02	Very soluble
25E	3.61	2.08	1.69	1.49	Soluble	-2.42	3.82E-03	Soluble
25D	2.21	1.81	2.61	1.51	Soluble	-2.75	1.78E-03	Soluble
5A	2.61	-1.55	-0.77	-0.62	Very soluble	-0.1	7.91E-01	Very soluble
5B	2.33	2.71	3.5	1.8	Soluble	-3.63	2.34E-04	Soluble

General Procedure and Synthetic Scheme 4

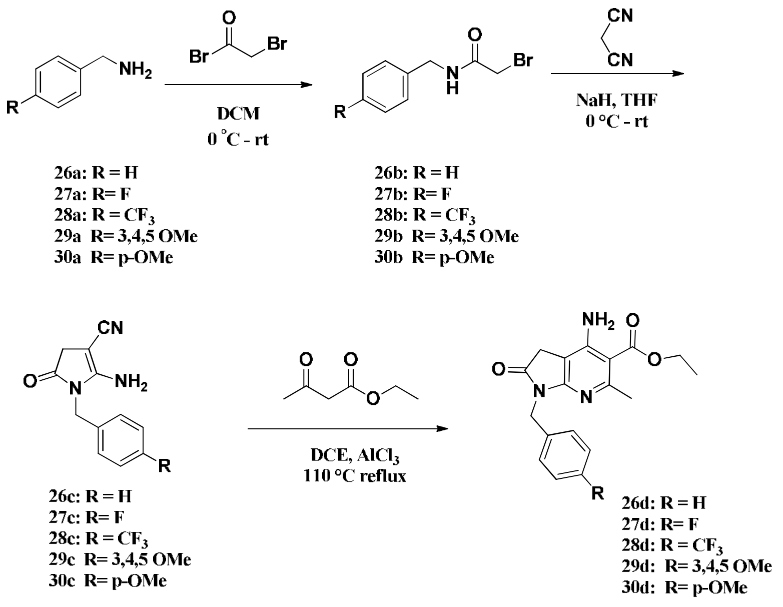

Scheme 4. A) DCM, 0 °C - r.t, 1 h, 95 %; B) NaH, THF, 0 °C - r.t, 52%; C) $AlCl_3$, DCE, 110 °C, reflux, 4 h, 48 %

Due to the great potential of pharmaceutical and biological applications, we aimed at exploring a concise and efficient approach (Scheme 4) to synthesize 7-azaindole derivatives. Here, we started from substituted N-benzyl-2-bromoacetamine **(26b, 27b, 28b, 29b and 30b)**, which could be obtained from 2-bromoacetyl bromide **2** with various substituted phenylmethanamine **(26a, 27a, 28a, 29a and 30a)**. Treatment of substituted N-alkyl-α-bromoacetamide with malononitrile **4** in THF in the presence of NaH, afforded substituted enamino γ-lactams **(26c, 27c, 28c, 29c and 30c)**. 7-azaindole derivatives **(26d, 27d, 28d, 29d and 30d)** could be constructed through friedlander reaction of substituted enamino γ-lactams **(26c, 27c, 28c, 29c and 30c)** and ethyl acetoacetate with lewis acid ($AlCl_3$) under reflux condition.

General Procedure for the Synthesis of substituted-(benzylamino)-3-bromopropan-2-one 26b, 27b, 28b, 29b and 30b: 2-bromoacetyl bromide (1.40 ml, 16.1 mmol and 1.0 eq) was added dropwise to a solution of substituted-phenylmethanamine (2.30 ml, 21.1 mmol, and 1.0 eq) in DCM (10 mL) at 0 °C. The mixture was allowed to warm up to room temperature and stirred for another 2 h. After completion of the reaction (TLC showed no starting material),

the reaction was quenched with H_2O (5 mL). The precipitate was filtered off, the organic phase was washed with H_2O, dried over Na_2SO_4 and evaporated to afford the product as white solid.

General Procedure for the Synthesis of Substituted N-alkyl-α-bromoacetamide 26c, 27c, 28c, 29c and 30c: Malononitrile (3.47 g, 52.5 mmol, 2.5 eq.) was added in small fraction to a suspension of NaH (1.22 g, 50.8 mmol, and 2.5 eq.) in THF (40 mL) at 0 °C. The mixture was allowed to warm up to room temperature and stirred for 30 minutes. After addition of Substituted-(benzylamino)-3-bromopropan-2-one (4.80 g, 21.0 mmol, 1.0 eq.), the mixture was stirred at room temperature overnight. After completion of the reaction, the reaction was quenched with ammonium chloride aqueous solution (40 ml) and extracted with ethyl acetate for two times. The organic phase was combined, dried over Na_2SO_4 and concentrated under reduced pressure. The residue was purified by column chromatography to give product as white solid.

General Procedure for the Synthesis of ethyl 1-benzyl-6-methyl-2-oxo-2,3-dihydro-1H-pyrrolo[2,3-b]pyridine-5-carboxylate (26d, 27d, 28d, 29d and 30d) Ethyl 3-oxobutanoate (1.60 ml, 12.5 mmol, 2.2 eq.) was added to a suspension of aluminium trichloride (1.50 g, 11.2 mmol, 2.2 eq.) in 1,2-dichloroethane. The mixture was reflux at 110 °C for 5 h. 2-amino-1-benzyl-5-oxo-4,5-dihydro-1H-pyrrole-3-carbonitrile (1.20 g, 5.60 mmol) was added to the above reaction mixture and stirred at this temperature for 5 h. The reaction was quenched by adding an ammonium chloride aqueous solution (40 mL) with ice cooling and then extracted with ethyl acetate. The organic phase was washed with brine and dried over Na_2SO_4. The solvent was concentrated under reduced pressure to give a white solid.

26b) N-benzyl-2-bromoacetamide:

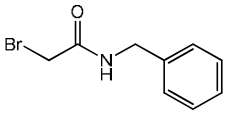

White solid (Crude); ^{1}H NMR (600 MHz, DMSO-*d6*) δ 8.81 (s, 1H, NH), 7.44 (dt, J = 10.7, 5.1 Hz, 2H), 7.32 (d, J = 7.3 Hz, 2H), 7.27 (s, 1H), 4.29 (d, J = 5.9 Hz, 2H), 3.92 (s, 2H); ^{13}C NMR (151 MHz, DMSO-*d6*) δ 166.1, 138.8, 134.0, 128.9, 128.6, 128.5, 128.3, 127.3, 126.9, 42.5, 29.5.; ESI-MS: *m/z* 229.03 $[M+H]^+$

27b) 2-bromo-N-(4-fluorobenzyl)acetamide:

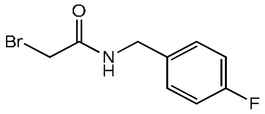

(Crude); White solid; ^{1}H NMR (600 MHz, DMSO-*d6*) δ 8.79 (s, 1H), 7.29 (d, J = 5.7 Hz, 2H), 7.16 (t, J = 8.5 Hz, 2H), 4.27 (d, J = 5.5 Hz, 2H), 3.90 (s, 2H).^{13}C NMR (151 MHz, DMSO-*d6*) δ 166.1, 135.1, 135.0, 129.3, 129.2, 115.2, 114.9, 41.8, 29.4; ESI-MS: *m/z* 247.03 [M+H]$^{+}$

28b) 2-bromo-N-(4-(trifluoromethyl) benzyl) acetamide:

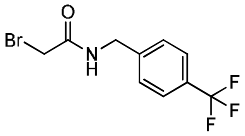

White solid (Crude); ^{1}H NMR (600 MHz, DMSO-*d6*) δ 8.90 (s, 1H), 7.70 (d, J = 7.9 Hz, 2H), 7.48 (d, J = 7.9 Hz, 2H), 4.39 (d, J = 5.7 Hz, 2H), 3.93 (s, 2H); ^{13}C NMR (151 MHz, DMSO-*d6*) δ 166.3, 143.8, 127.9, 125.3, 125.3, 125.2, 125.2, 125.2, 42.1, 29.3; ESI-MS: *m/z* 296.04 [M+H]$^{+}$

29b) 2-bromo-N-(3,4,5-trimethoxybenzyl)acetamide:

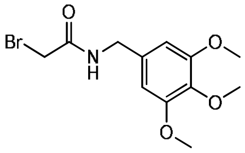

White solid (Crude); ^{1}H NMR (600 MHz, DMSO-*d6)* δ 6.70 (s, 2H), 6.57 (s, 1H), 4.74 (s, 2H), 3.73 (s, 6H), 3.63 (s, 3H), 3.93 (s, 2H); ^{13}C NMR (151 MHz, DMSO-*d6*) δ 174.30, 167.39, 187.94, 156.98, 136.75, 132.53, 105.13, 60.50, 59.30, 55.70, 32.7; ESI-MS: *m/z* 318.08 [M+H]$^{+}$

30b) 2-bromo-N-(4-methoxybenzyl)acetamide:

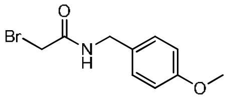

White solid (Crude); ^{1}H NMR (600 MHz, DMSO-*d6)* δ 8.70 (s, 1H), 7.21 (t, J = 8.8 Hz, 2H), 6.95 – 6.84 (m, 2H), 4.23 (d, J = 5.9 Hz, 2H), 3.90 (s, 2H), 3.75 (s, 3H).^{13}C NMR (151 MHz, DMSO-*d6*) δ 174.30, 167.39, 187.94, 156.98, 136.75, 132.53, 105.13, 60.50, 59.30, 55.70, 32.7; ESI-MS: *m/z* 258.11 [M+H]$^{+}$

26c) 2-amino-1-benzyl-5-oxo-4, 5-dihydro-1H-pyrrole-3-carbonitrile:

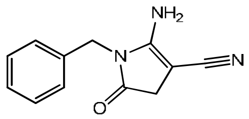

Orange solid (Crude); ^{1}H NMR (600 MHz, DMSO-*d6*) δ 7.71 (d, J = 8.1 Hz, 2H), 7.42 (d, J = 8.0 Hz, 2H), 7.27 (s, 2H), 4.84 (s, 2H), 3.30 (s, 2H).^{13}C NMR (101 MHz, DMSO-*d6*) δ 173.9, 157.5, 141.3, 127.6, 125.4, 125.4, 125.3, 123.3, 119.1, 47.3, 41.4, 34.3, 8.4; ESI-MS: *m/z* 214.03 $[M+H]^+$

27c) 2-amino-1-(4-fluorobenzyl)-5-oxo-4, 5-dihydro-1H-pyrrole-3-carbonitrile:

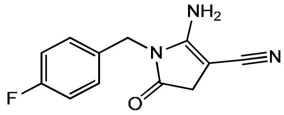

Orange solid (Crude); ^{1}H NMR (600 MHz, DMSO-*d6*) *δ* 7.27 (dd, *J* = 8.5, 5.6 Hz, 2H), 7.24 (s, 2H), 7.16 (t, *J* = 8.9 Hz, 2H), 4.71 (s, 2H), 3.27 (s, 2H). ^{13}C NMR (151 MHz, DMSO-*d6*) δ 173.9, 157.6, 132.7, 129.1, 119.2, 115.3, 115.1, 47.2, 41.0, 34.3, 8.4; ESI-MS: *m/z* 232.23 $[M+H]^+$

28c) 2-amino-5-oxo-1-(4-(trifluoromethyl)benzyl)-4,5-dihydro-1H-pyrrole-3-carbonitrile:

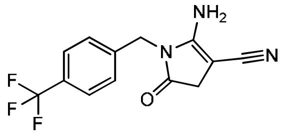

Orange solid (Crude); ^{1}H NMR (600 MHz, DMSO-*d6*) *δ* 7.71 (d, *J* = 8.1 Hz, 2H), 7.42 (d, *J* = 8.0 Hz, 2H), 7.27 (s, 2H), 4.84 (s, 2H), 3.30 (s, 2H).^{13}C NMR (151 MHz, DMSO-*d6*) *δ* 173.9, 157.5, 141.3, 127.6, 125.4, 125.4, 125.3, 123.3, 119.1, 47.3, 41.4, 34.3, 8.4; ESI-MS: *m/z* 282.31 $[M+H]^+$

29c) 2-amino-5-oxo-1-(3,4,5-trimethoxybenzyl)-4,5-dihydro-1H-pyrrole-3-carbonitrile:

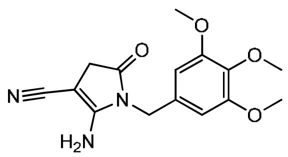

Orange solid (Crude); ^{1}H NMR (600 MHz, DMSO-*d6*) *δ* 7.23 (s, 2H), 7.19 (t, *J* = 5.8 Hz, 2H), 6.92 – 6.84 (m, 2H), 4.68 (s, 2H), 3.74 (s, 3H), 3.26 (s, 2H).).^{13}C NMR (151 MHz, DMSO-*d6*) *δ* 174.05, 158.64, 157.45, 130.61, 128.54, 118.97, 113.77, 54.83, 46.68, 34.35, 8.67; ESI-MS: *m/z* 304.21 $[M+H]^+$

30c) 2-amino-1-(4-methoxybenzyl)-5-oxo-4,5-dihydro-1H-pyrrole-3-carbonitrile:

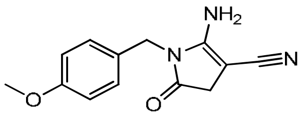

Orange solid (Crude); ^{1}H NMR (600 MHz, DMSO-*d6*) δ 7.21 (s, 2H), 6.60 (d, *J* = 8.5 Hz, 2H), 4.65 (d, *J* = 7.1 Hz, 2H), 4.45 (d, *J* = 6.1 Hz, 2H), 3.75 (s, 6H), 3.65 (s, 3H).^{13}C NMR (151 MHz, DMSO-*d6*) δ 173.84, 157.46, 152.67, 137.00, 132.00, 118.98, 112.21, 104.89, 59.63, 47.02, 41.84, 34.37, 8.63; ESI-MS: *m/z* 244.61 $[M+H]^+$

26d) ethyl 4-amino-1-benzyl-6-methyl-2-oxo-2,3-dihydro-1H-pyrrolo[2,3-b]pyridine-5-carboxylate:

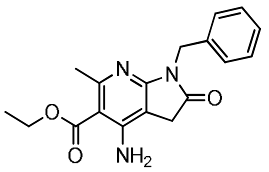

White solid (Crude); ^{1}H NMR (600 MHz, DMSO-*d6*) 7.27-7.39 (m, 5H), 6.58 (s br, 2H), 4.27 (q, *J*= 7.1 Hz, CH_2, 2H,), 3.43 (s, CH_2, 2H), 2.47 (s, CH_3, 3H), 1.31 (t, *J*= 7.1 Hz, CH_3, 3H,) ; 13C NMR (151 MHz, DMSO-*d6*) δ 174.4, 173.9, 149.4, 137.3, 137.5, 136.5, 128.4, 128.3, 127.4, 127.2, 127.1, 126.8, 97.4, 60.5, 41.6, 34.3, 32.9, 25.6, 14.0; ESI-MS: *m/z* 326.31 $[M+H]^+$

27d) ethyl 4-amino-1-(4-fluorobenzyl)-6-methyl-2-oxo-2,3-dihydro-1H-pyrrolo[2,3-b]pyridine-5-carboxylate:

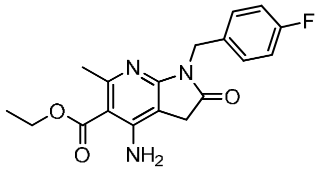

White solid (Crude); ^{1}H NMR (600 MHz, DMSO-*d6*) δ 7.35 (dd, *J* = 8.3, 5.8 Hz, 2H, Aromatic H), 7.12 (t, *J* = 8.9 Hz, 2H;aromatic H), 6.62 (s, 1H, NH), 4.80 (s, 2H, CH_2), 4.29 (q, *J* = 7.1 Hz, 2H, CH_2), 3.43 (d, *J* = 12.8 Hz, 2H, CH_2), 2.47 (s, 3H, CH_3), 1.30 (t, *J* = 7.1 Hz, 3H, CH_3).^{13}C NMR (151 MHz, DMSO-*d6*) δ 174.4, 167.6, 162.1, 160.5, 156.7, 149.4, 133.4, 129.6,129.5, 115.1, 115.0, 106.9, 97.4, 60.5, 41.0, 33.0, 25.4, 14.0; ESI-MS: *m/z* 344.56 $[M+H]^+$

28d) ethyl 4-amino-6-methyl-2-oxo-1-(4-(trifluoromethyl)benzyl)-2,3-dihydro-1H-pyrrolo[2,3-b]pyridine-5-carboxylate

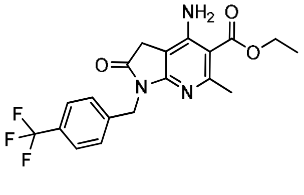

White solid (Crude); ^{1}H NMR (600 MHz, DMSO-*d6*) δ 7.66 (d, J = 8.1 Hz, 2H), 7.50 (d, J = 8.0 Hz, 2H), 6.60 (s, 2H), 4.90 (s, 2H), 4.29 (q, J = 7.1 Hz, 2H), 3.45 (s, 2H), 2.45 (s, 3H), 1.29 (s, 3H).^{13}C NMR (151 MHz, DMSO-*d6*) δ 174.5, 167.7, 158.4, 156.8, 149.4, 142.0, 128.1, 125.3, 123.3, 106.9, 97.5, 60.4, 49.6, 41.2, 33.0, 30.0, 25.6, 14.0; ESI-MS: *m/z* 394.46 [M+H]$^+$

29d) ethyl 4-amino-6-methyl-2-oxo-1-(3,4,5-trimethoxybenzyl)-2,3-dihydro-1H-pyrrolo[2,3-b]pyridine-5-carboxylate:

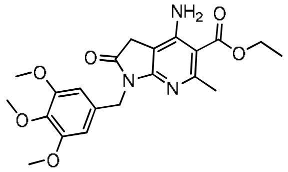

White solid (Crude); ^{1}H NMR (600 MHz, DMSO-*d6*) δ 6.69 (d, J = 11.6 Hz, 2H), 6.58 (bs, 2H), 4.74 (s, 2H), 4.31 (q, J = 7.1 Hz, 2H), 3.72 (d, J = 7.0 Hz, 6H), 3.66 – 3.59 (m, 3H), 3.43 (s, 3H), 1.32 (t, J = 7.1 Hz, 2H).^{13}C NMR (151 MHz, DMSO-*d6*) δ 174.5, 167.7, 158.4, 156.8, 149.4, 142.0, 128.1, 125.3, 123.3, 106.9, 97.5, 60.4, 49.6, 41.2, 33.0, 30.0, 25.6, 14.0; ESI-MS: *m/z* 416.43 [M+H]$^+$

30d) ethyl 4-amino-1-(4-methoxybenzyl)-6-methyl-2-oxo-2,3-dihydro-1H-pyrrolo[2,3-b]pyridine-5-carboxylate:

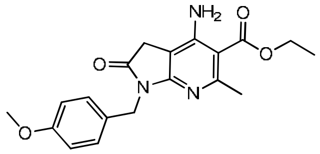

White solid (Crude); ^{1}H NMR (600 MHz, DMSO-*d6*) δ 7.46 (d, J = 7.7 Hz, 2H), 6.83 (d, J = 8.0 Hz, 2H), 5.74 (s, 2H), 4.92 (s, 2H), 4.38 (d, J = 6.9 Hz, 2H), 3.78 (s, 3H), 3.29 (s, 2H), 2.72 (s, 3H), 1.42 (t, J = 6.8 Hz, 3H).^{13}C NMR (151 MHz, DMSO-*d6*) δ 174.5, 167.7, 158.4, 156.8, 149.4, 142.0, 128.1, 125.3, 123.3, 106.9, 97.5, 60.4, 49.6, 41.2, 33.0, 30.0, 25.6, 14.0; ESI-MS: *m/z* 356.49[M+H]$^+$

31) 1H-indol-5-amine:

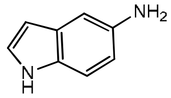

Brown (Crude): ^{1}H NMR (600 MHz, DMSO-*d6*) δ 10.57 (s, 1H), 7.16 – 7.07 (m, 2H), 6.70 (d, *J* = 2.0 Hz, 1H), 6.51 (dd, *J* = 8.5, 2.1 Hz, 1H), 6.14 (d, *J* = 2.2 Hz, 1H), 4.41 (s, 2H). ^{13}C NMR (151 MHz, DMSO-*d6*) δ 141.22, 129.63, 128.36, 124.57, 111.79, 111.49, 103.24, 99.46;

General Procedure and Synthetic Scheme 5

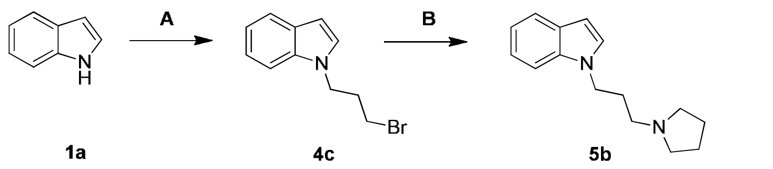

Scheme 5: A) K_2CO_3, DMF, 1,3-Dibromopropane, r.t, 4 h, 65 %; B) Dry ACN, Pyrrolidine, reflux, 4 h, 42% yield

1-(3-bromopropyl)-1H-indole (4c): 1H-indole (2.01 g, 17.09 mmol) was added to a solution of anhydrous KOH (17.09 mmol) in DMF (100 ml) at room temperature for 30 min, followed by the addition of 1,3-dibromopropane (34.18 mmol). The reaction mixture was then allowed to stir overnight. Solvent was removed under reduced pressure, and the crude product was purified on silica as a stationary phase; Yield 66 % as white crystals; R_f = 0.61 (eluent EA/n-Hex 1:4); mp 92°C; ^{1}H NMR (400 MHz, DMSO-d6): δ 7.33-7.38 (m, 3H), 6.66 (d, *J* = 9.1 Hz, 1H), 6.41 -6.42 (d, *J* = 7.1 Hz, 1H), 6.23 (d, *J* = 7.2 Hz, 1H), 4.40 (t, *J* = 6.5 Hz, 2H), 3.28-3.31 (m, 2H), 2.33-2.44 (m, 2H) ppm; ESI-MS: *m/z* 239.0 [M+H]$^+$

1-(3-(pyrrolidin-1-yl)propyl)-1H-indole (5b): Purified 1-(3-bromopropyl)-1H-indole (4c) (0.42 g, 0.88 mmol) was dissolved in dry ACN (6 mL/mmol) and to this pyrrolidine (3 -10 equiv) was added, and the mixture was refluxed for 3−4 h. Solvent was concentrated under reduced pressure. The crude product was purified by column chromatography (eluent MeOH/DCM 1:3); using silica as a stationary phase; Yield 36 % as yellow viscous solid; R_f(eluent MeOH/ DCM 1:4) 0.45; mp 102-104 °C; 1H NMR (400 MHz, DMSO-*d6*): δ 7.31-7.33 (m, 3H), 6.66 (d, *J* = 8.1 Hz, 1H), 6.39 (d, *J* = 7.6 Hz, 1H), 6.19 (d, *J* = 7.2 Hz, 1H), 4.32 (t, *J* = 6.9 Hz, 2H), 3.72 (t, *J* = 6.9 Hz, 2H), 3.13 - 3.27 (m, 2H), 2.78 (bs, 2H), 2.09 - 2.27 (m, 6H), ppm; 13C NMR (101 MHz, DMSO-*d6*): δ 140.56, 139.65, 127.49, 126.18, 116.57, 115.96, 115.56, 115.22, 111.71, 54.12, 53.72, 46.46, 43.32, 26.50, 23.06 ppm; ESI-MS: *m/z* 229.33 [M+H]$^+$

HPLC methods used to assess the purity of compounds:

For all key target compounds purity is > 95%. The retention time of these compounds was between (15 and 25 minutes). Detected by HPLC system [Waters e2695 (alliance)] consisting of low pressure gradient pump plus auto sampler and Photo Diode Array (PDA) detector. The output signal was monitored and processed using Empower 2 software.

Column: GRACE-C18 (250 mm x 4.6 mm x 5 µm)

Mobile phase: 1% TFA Buffer and ACN & Methanol

Flow rate: 1 mL/min

Purity of key target compounds purity > 95%.

HPLC Chromatogram for 3, 9a, 5, 7 and 12

1) HPLC Chromatogram for 3 (96.30 % pure)

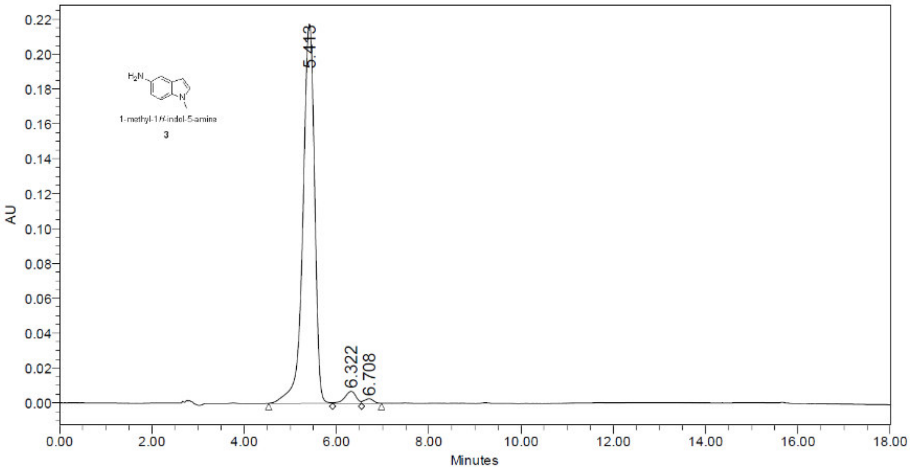

	RT	Area	% Area	Purity1 Threshold	Purity1 Angle
1	5.413	3828028	96.30	0.262	0.712
2	6.322	113200	2.85	1.200	15.996
3	6.708	33962	0.85	2.600	7.905

2) HPLC Chromatogram for 9a (97.10 % pure)

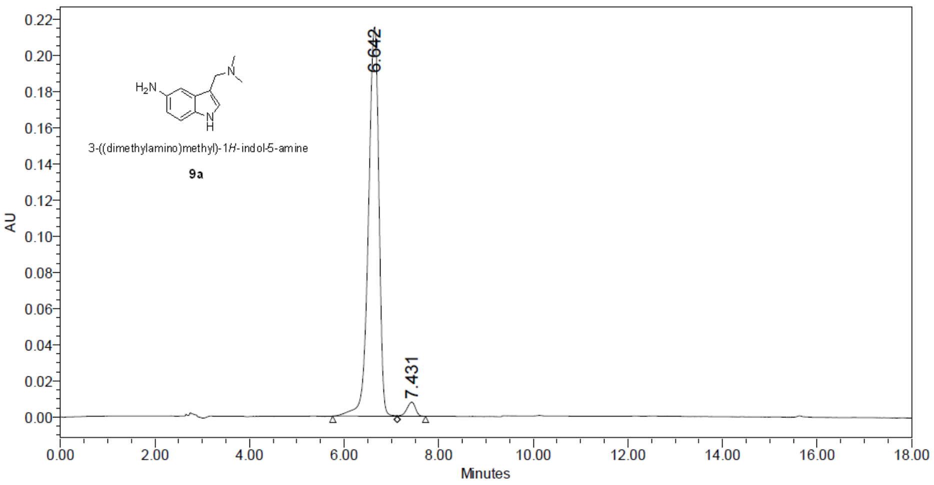

	RT	Area	% Area	Purity1 Threshold	Purity1 Angle
1	6.642	3312686	97.10	0.276	0.141
2	7.431	99059	2.90	1.511	3.976

3) HPLC Chromatogram for 5 (96.41 % pure)

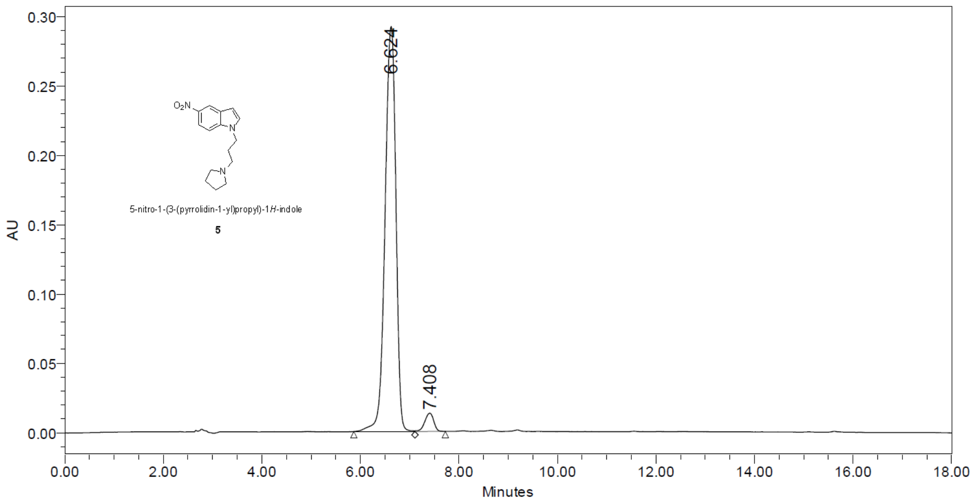

	RT	Area	% Area	Purity1 Threshold	Purity1 Angle
1	6.624	4452891	96.41	0.250	0.087
2	7.408	165931	3.59	0.674	0.605

4) HPLC Chromatogram for 7 (97.77 % pure)

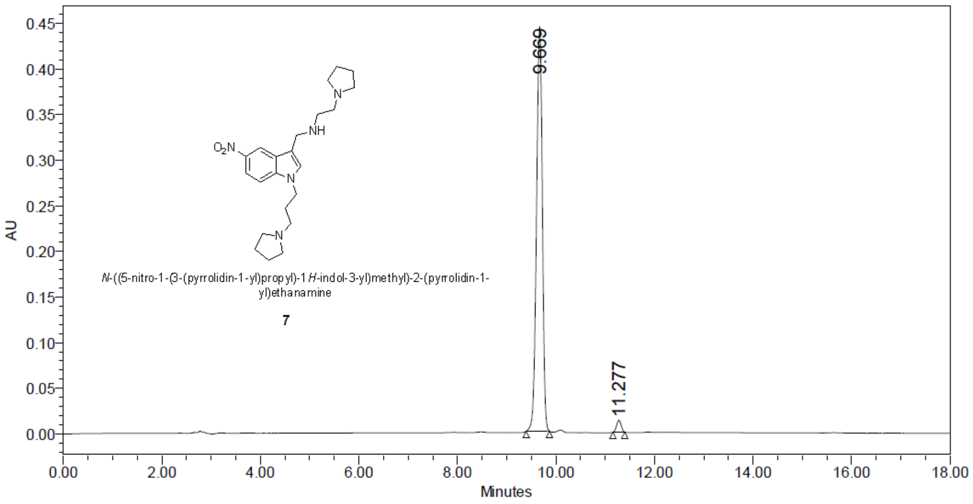

	RT	Area	% Area
1	9.669	3658421	97.77
2	11.277	83563	2.23

5) HPLC Chromatogram for 12 (97.61 % pure)

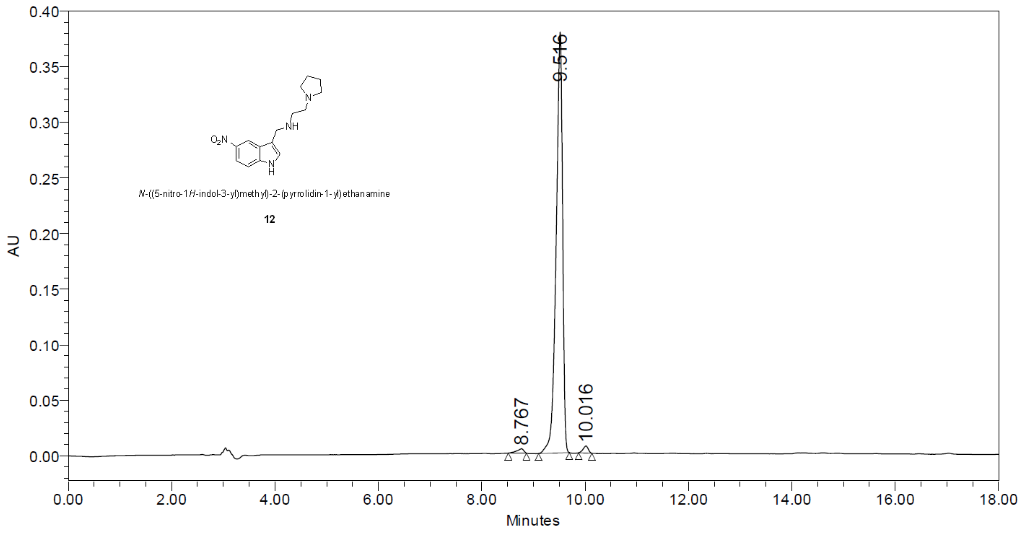

	RT	Area	% Area
1	8.767	36154	1.08
2	9.516	3269877	97.61
3	10.016	44032	1.31

6) HPLC Chromatogram for 5a (98.41 % pure)

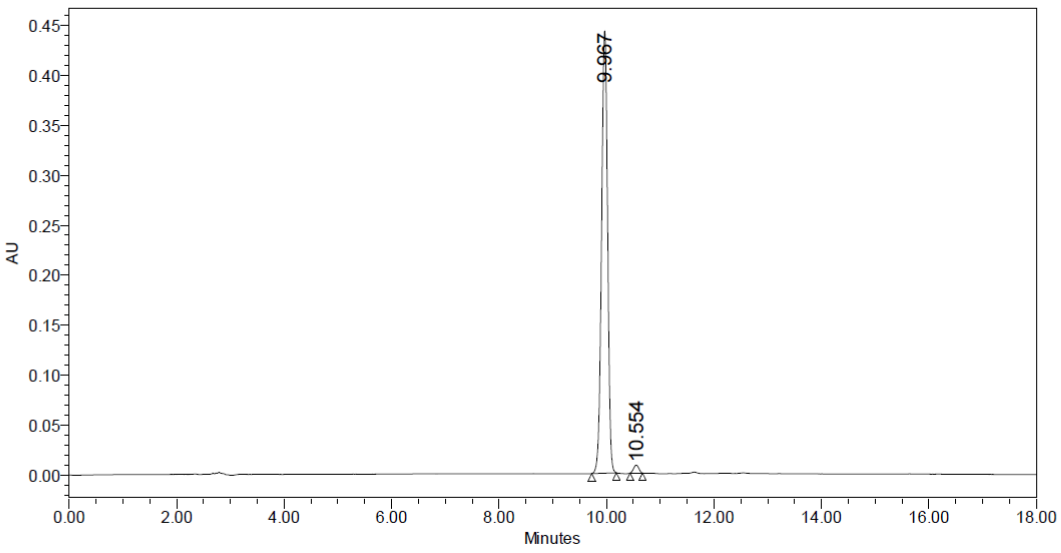

	RT	Area	% Area
1	9.967	3455981	98.48
2	10.554	53512	1.52

Supplementary data and NMR spectra

1-methyl-5-Amino-1H-indole (3)

^{1}H-NMR (600 MHz, DMSO-d_6)

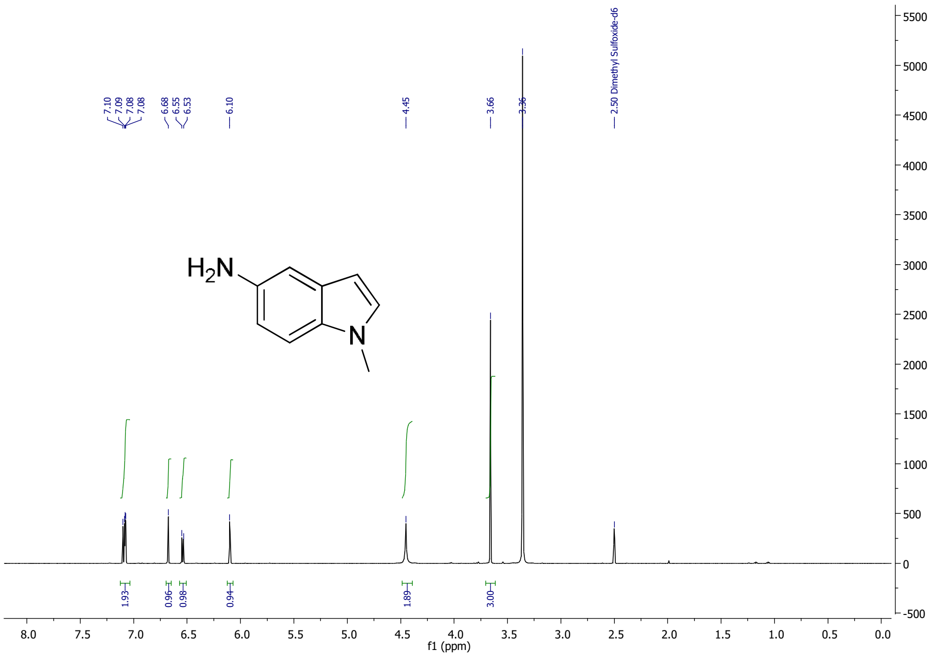

^{13}C NMR (151 MHz, DMSO-d_6)

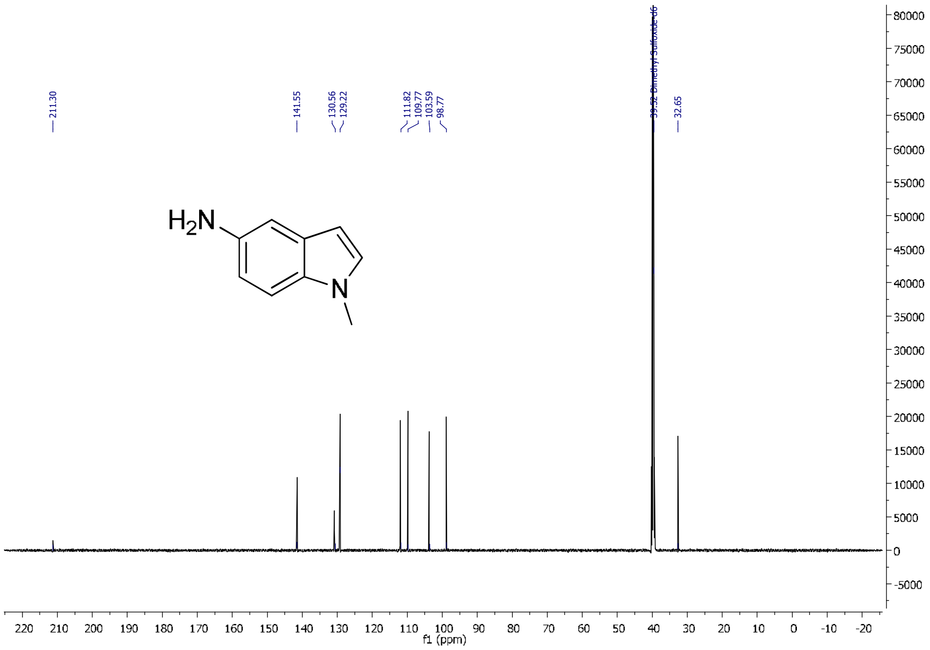

5-nitro-1-(3-bromopropyl)-1H-Indole (4a)

^{1}H NMR (600 MHz, $CDCl_3$)

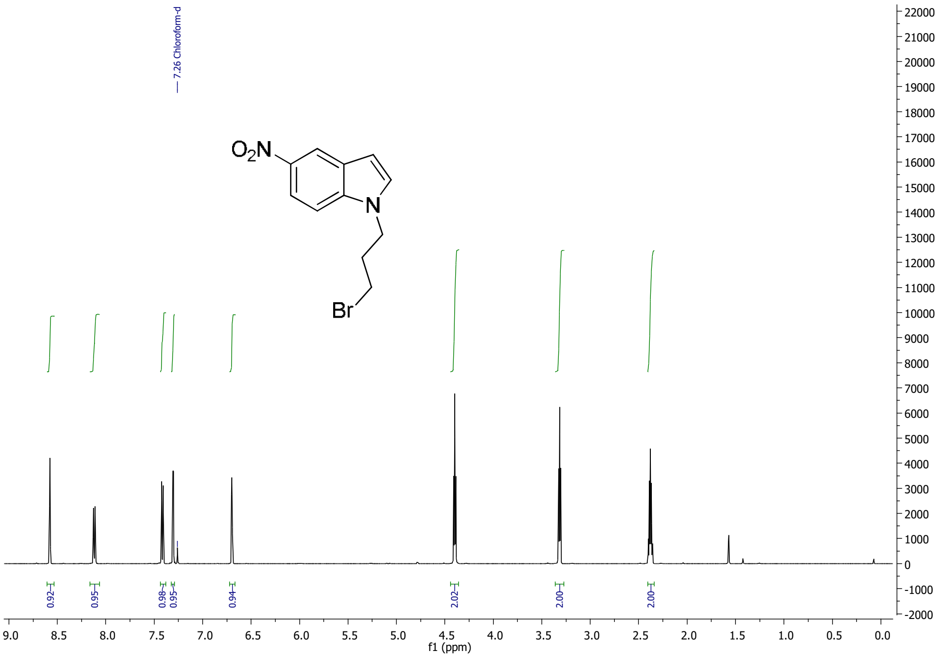

^{13}C NMR (151 MHz, $CDCl_3$)

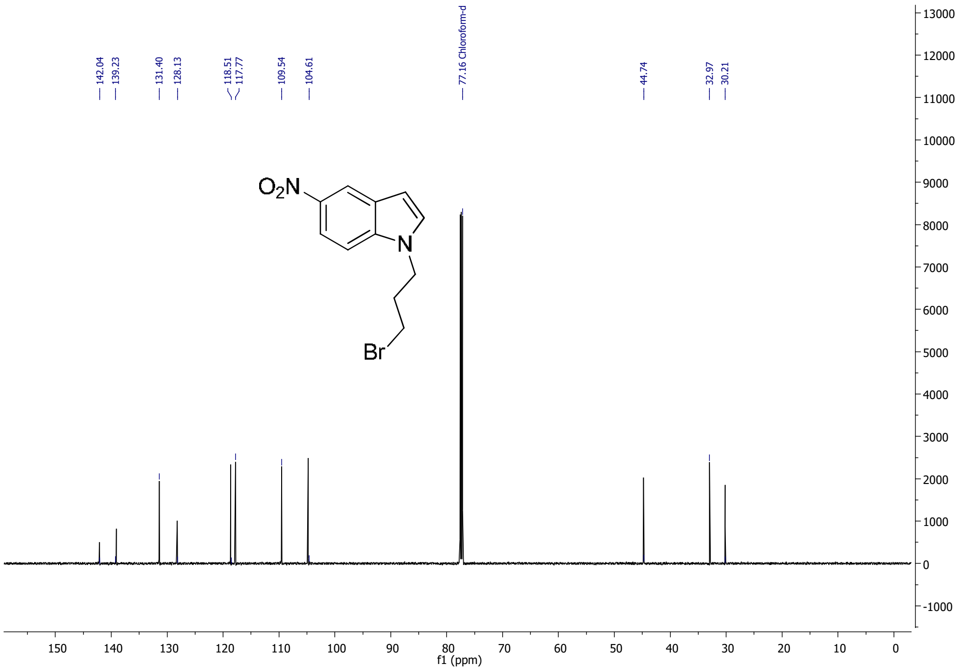

1, 3-bis(5-nitro-1H-indol-1-yl)propane (17a)

^{1}H NMR (600 MHz, $CDCl_3$)

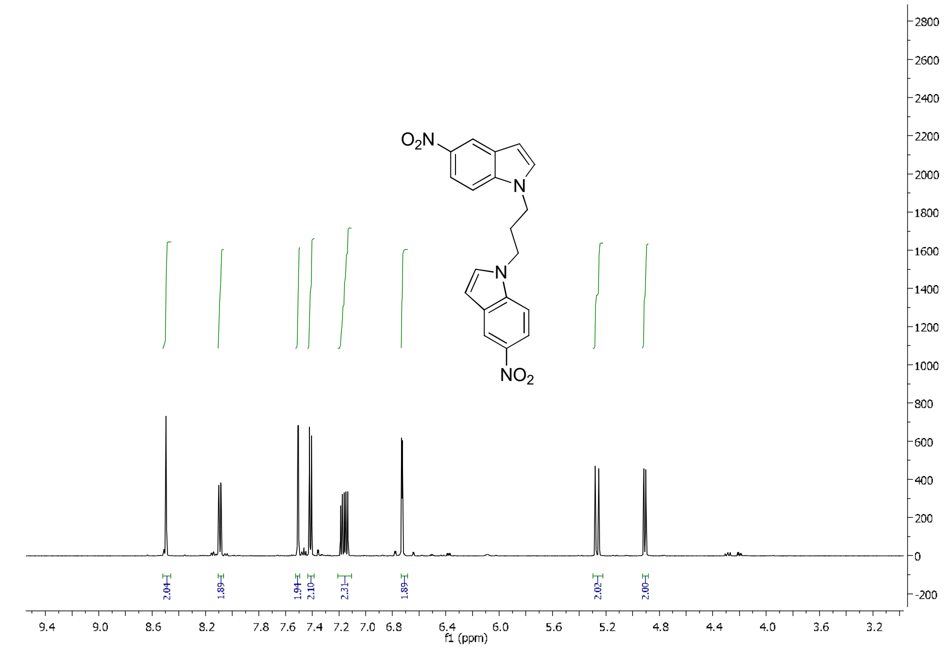

^{13}C NMR (151 MHz, $CDCl_3$)

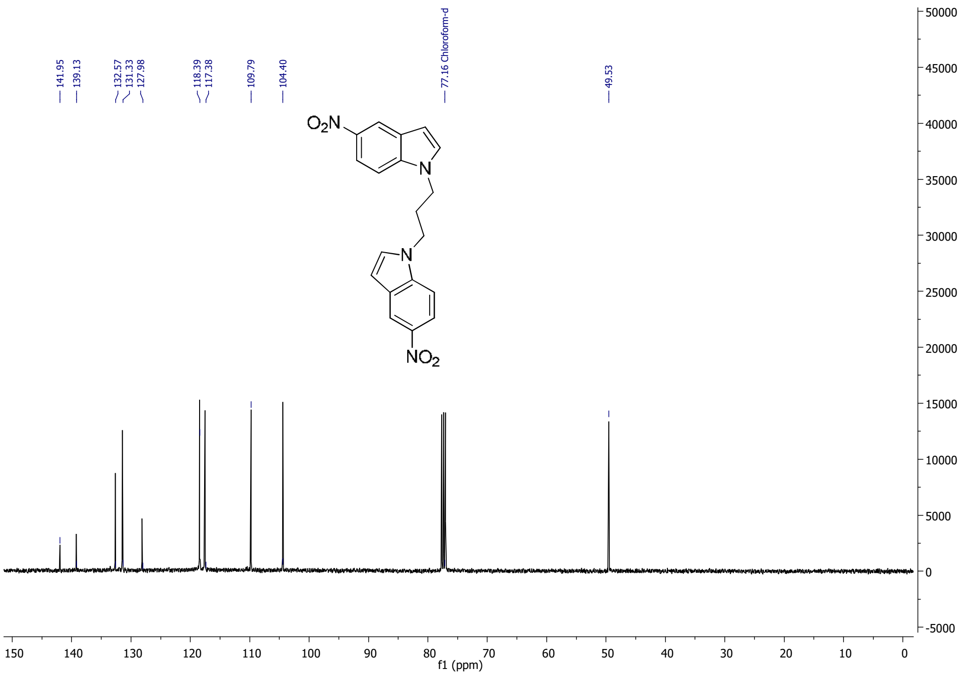

1-(2-bromoethyl)-5-nitro-1H-indole (4b)

^{1}H NMR (600 MHz, $CDCl_3$)

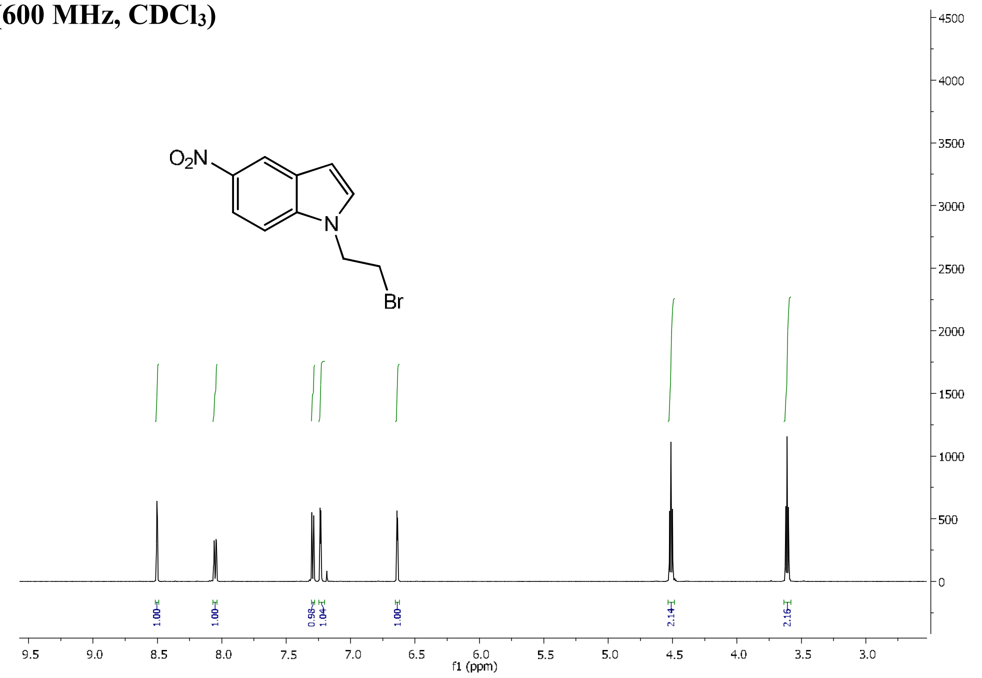

^{13}C NMR (151 MHz, $CDCl_3$)

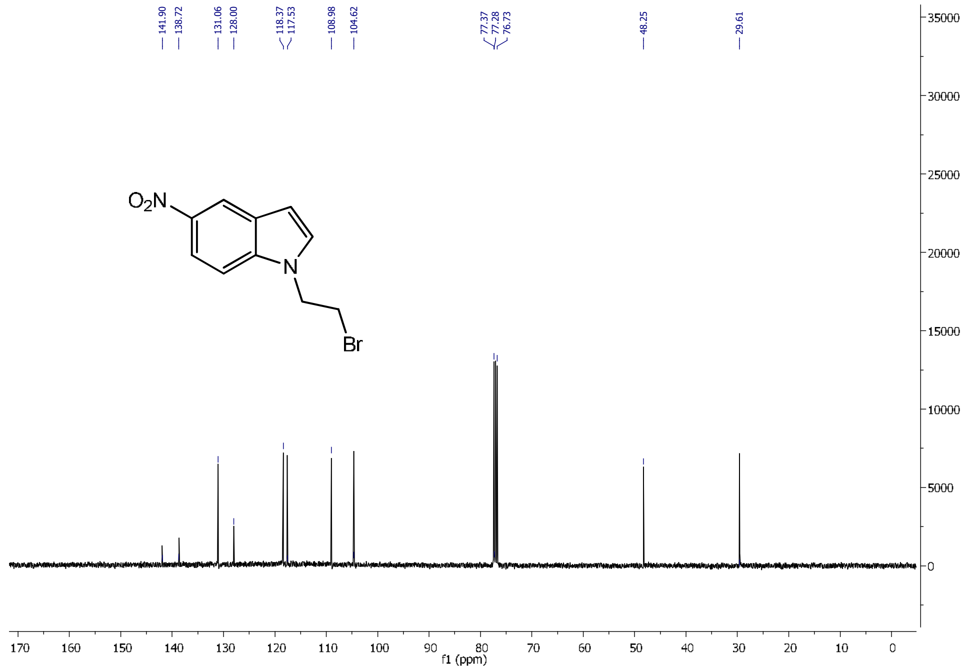

5-nitro-1-(3-(pyrrolidin-1-yl) propyl)-1H-indole (5)

^{1}H NMR (600 MHz, DMSO-d_6)

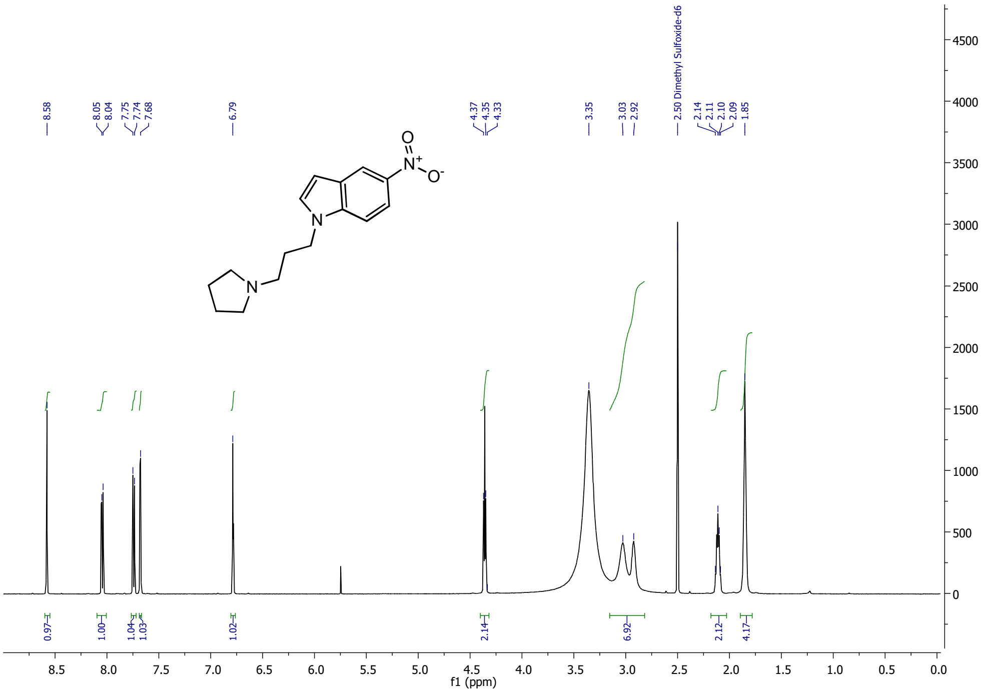

^{13}C NMR (151 MHz, DMSO-d_6)

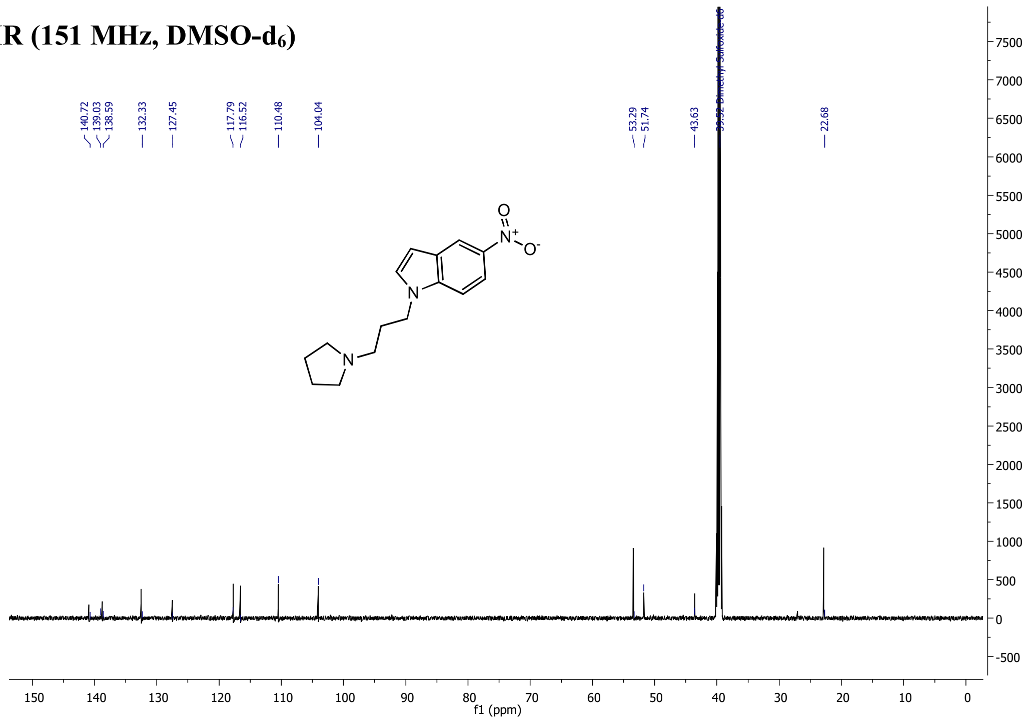

5-nitro-1-(3-(pyrrolidin-1-yl) propyl)-1H-indole-3-carbaldehyde (6)

^{1}H NMR (600 MHz, DMSO-d_6)

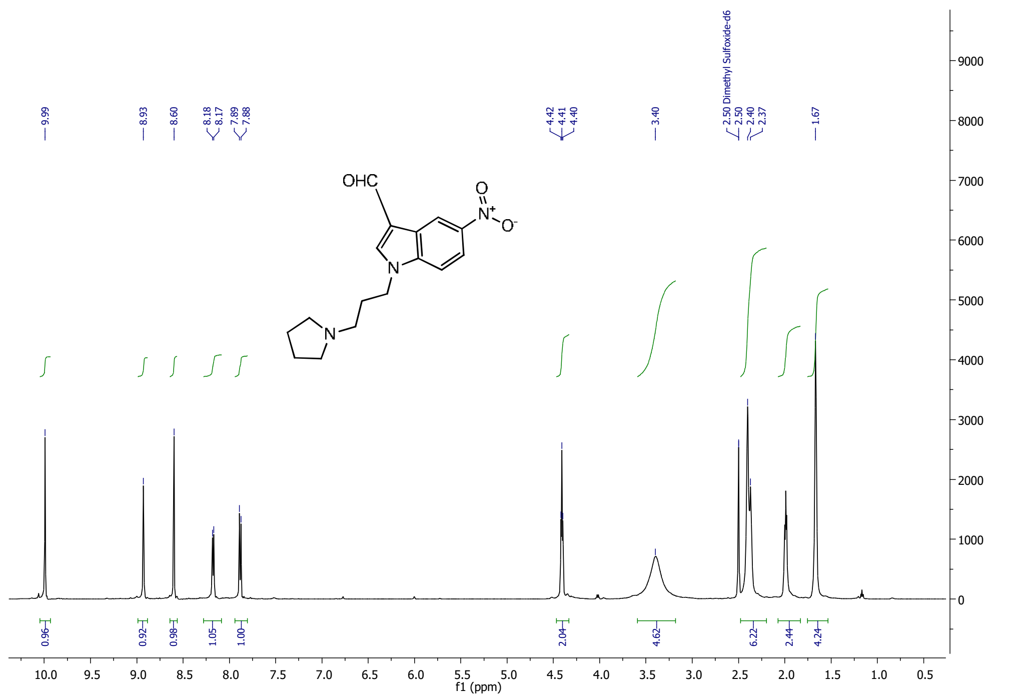

^{13}C NMR (151 MHz, DMSO-d_6)

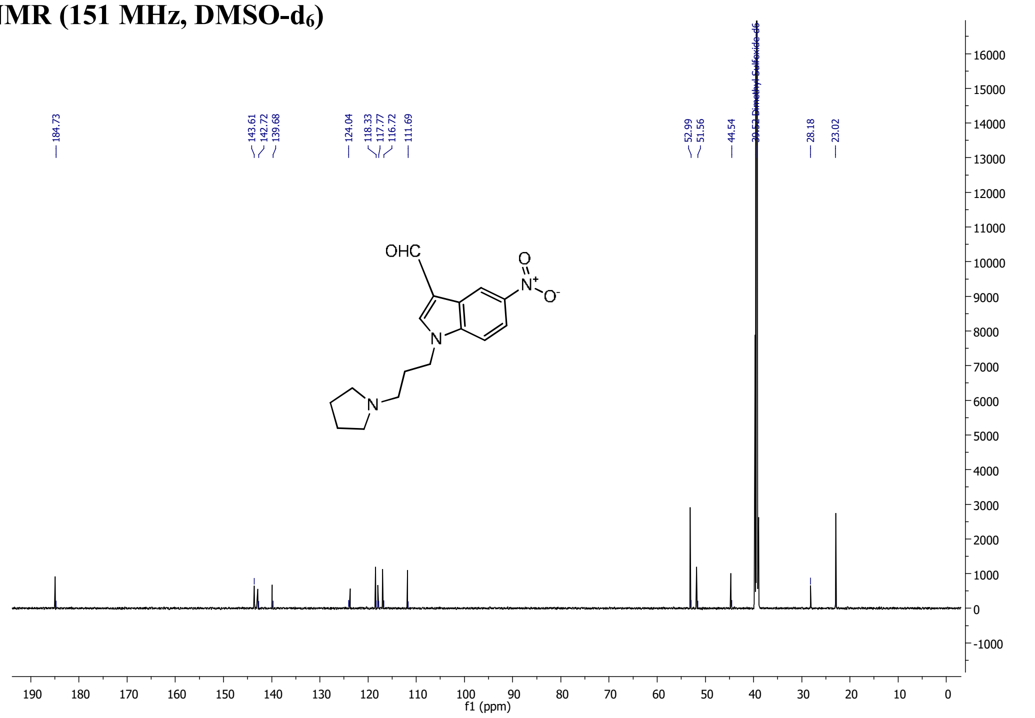

N-((5-nitro-1-(3-(pyrrolidin-1-yl)propyl)-1H-indol-3-yl)methyl)-2-(pyrrolidin-1-yl)ethanamine (7)

^{1}H NMR (600 MHz, DMSO-d$_6$)

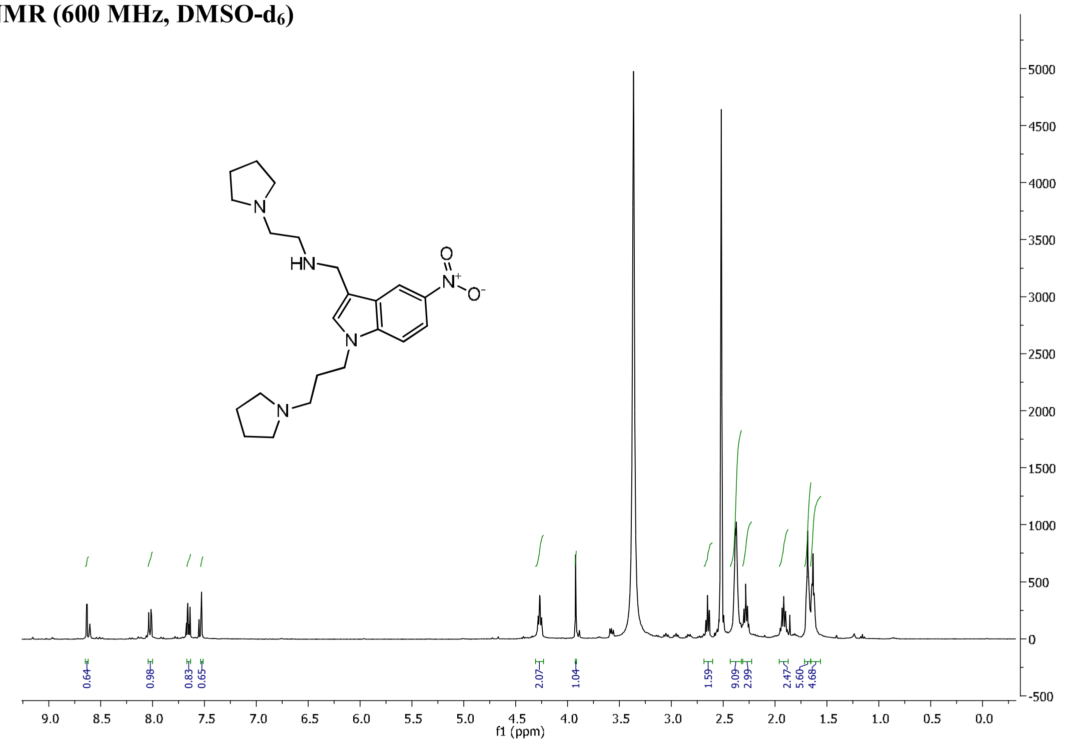

^{13}C NMR (151 MHz, DMSO-d$_6$)

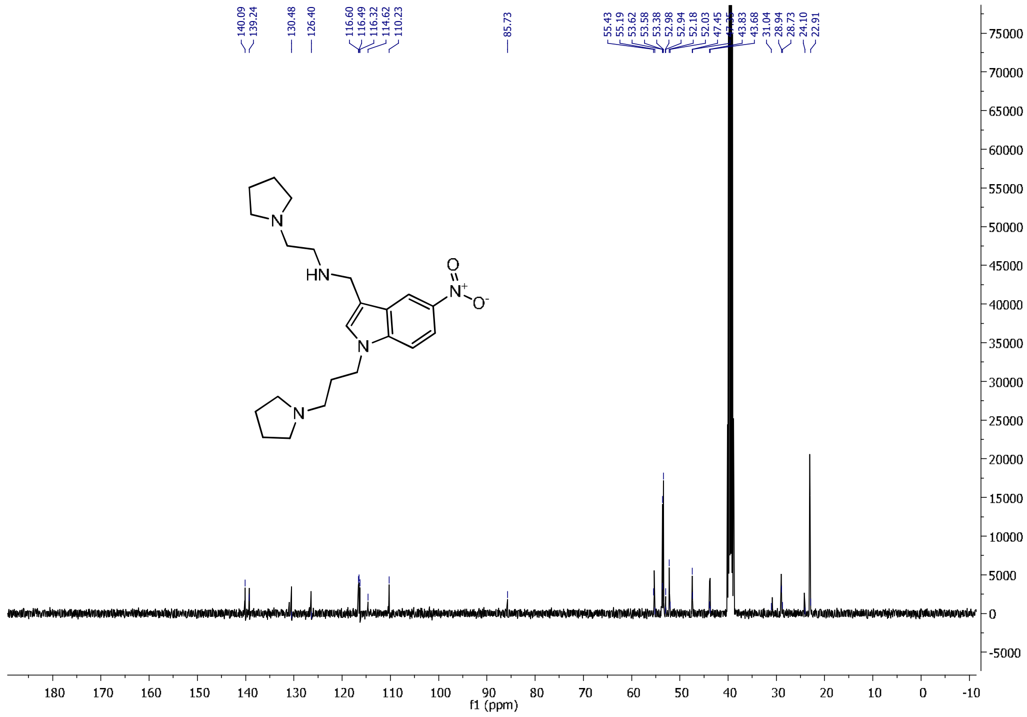

5-Nitroindole-3-carboxaldehyde (8)

^{1}H NMR (600 MHz, DMSO-d_6)

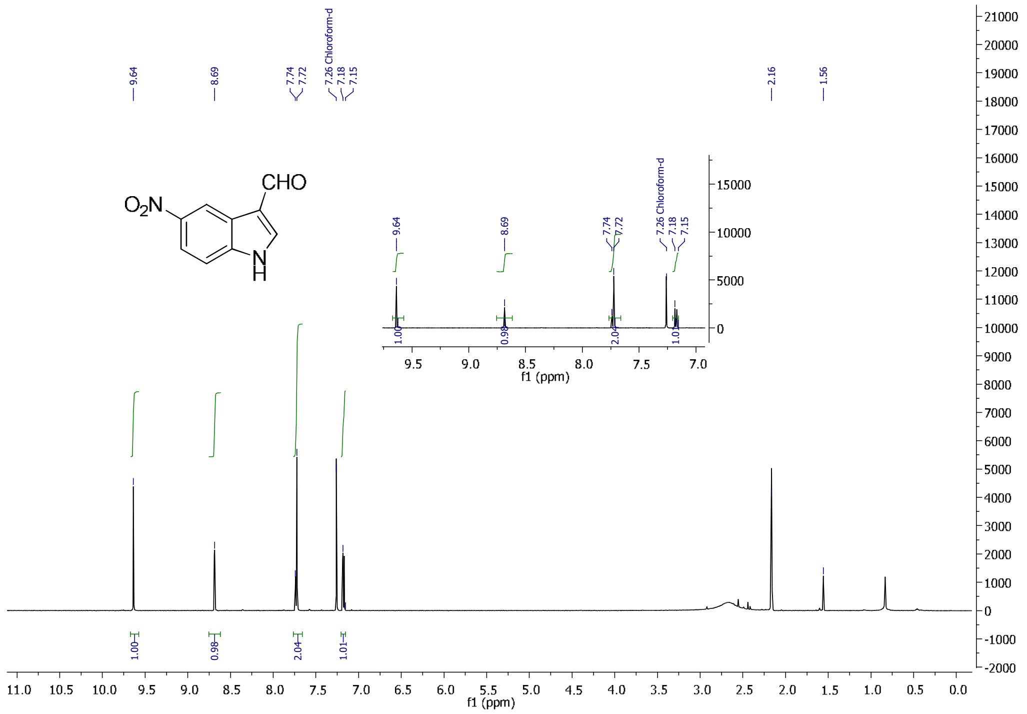

^{13}C NMR (151 MHz, DMSO-d_6)

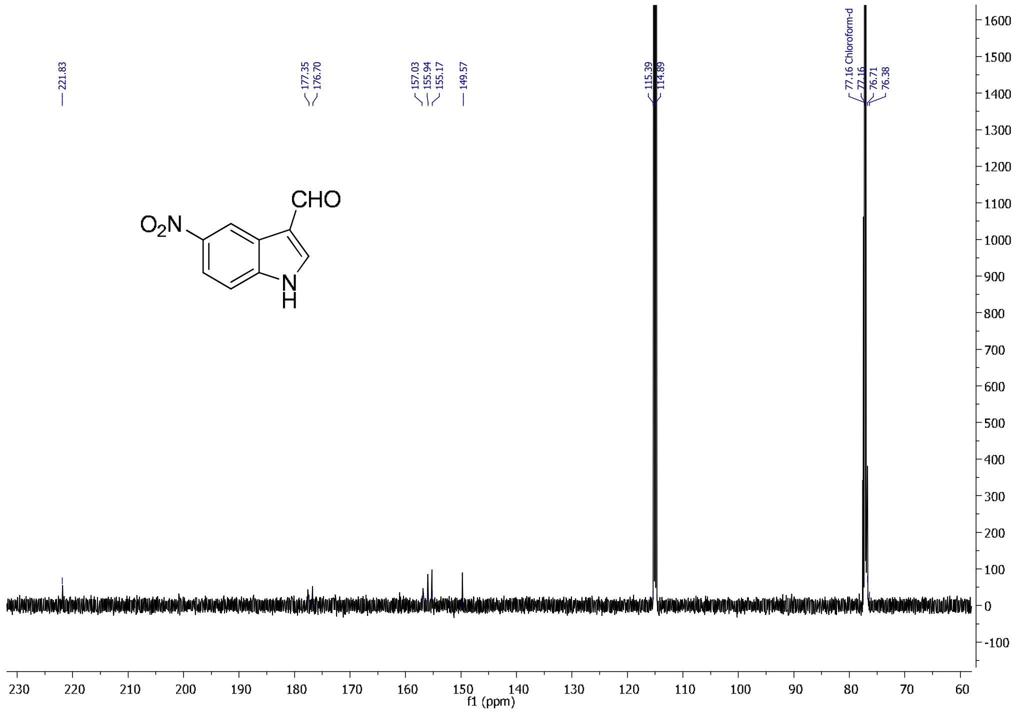

N,N-dimethyl-1-(5-nitro-1H-indol-3-yl)methanamine (9)

^{1}H NMR (600 MHz, DMSO-d_6)

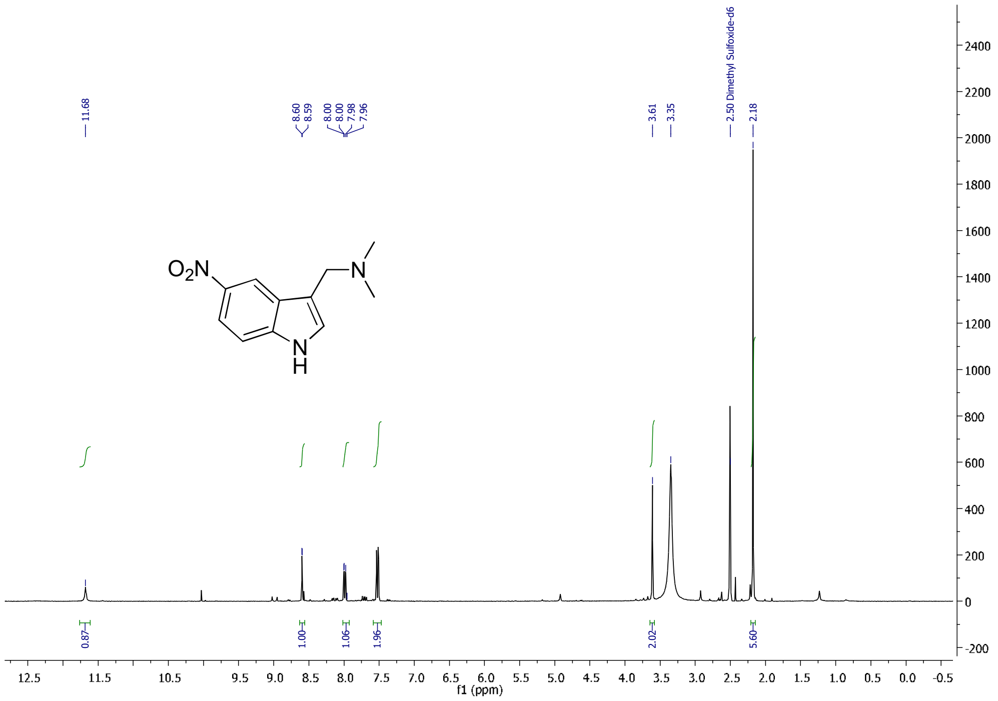

^{13}C NMR (151 MHz, DMSO-d_6)

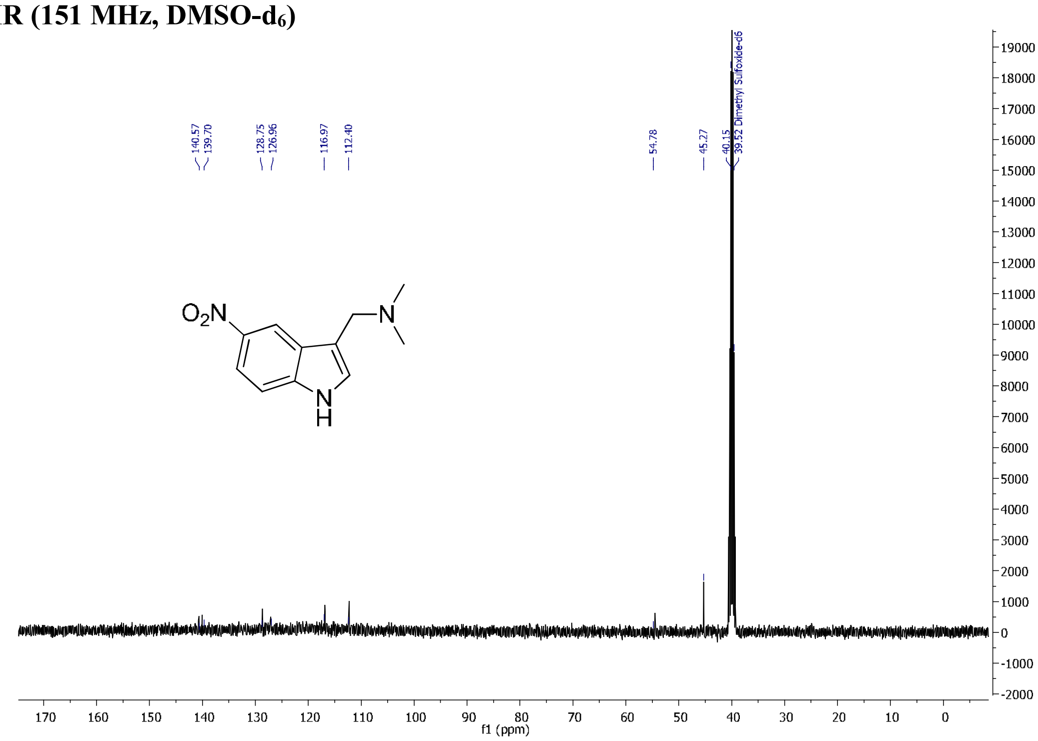

N,N-dimethyl-1-(5-amino-1H-indol-3-yl)methanamine (9a)

^{1}H NMR (600 MHz, DMSO-d$_6$)

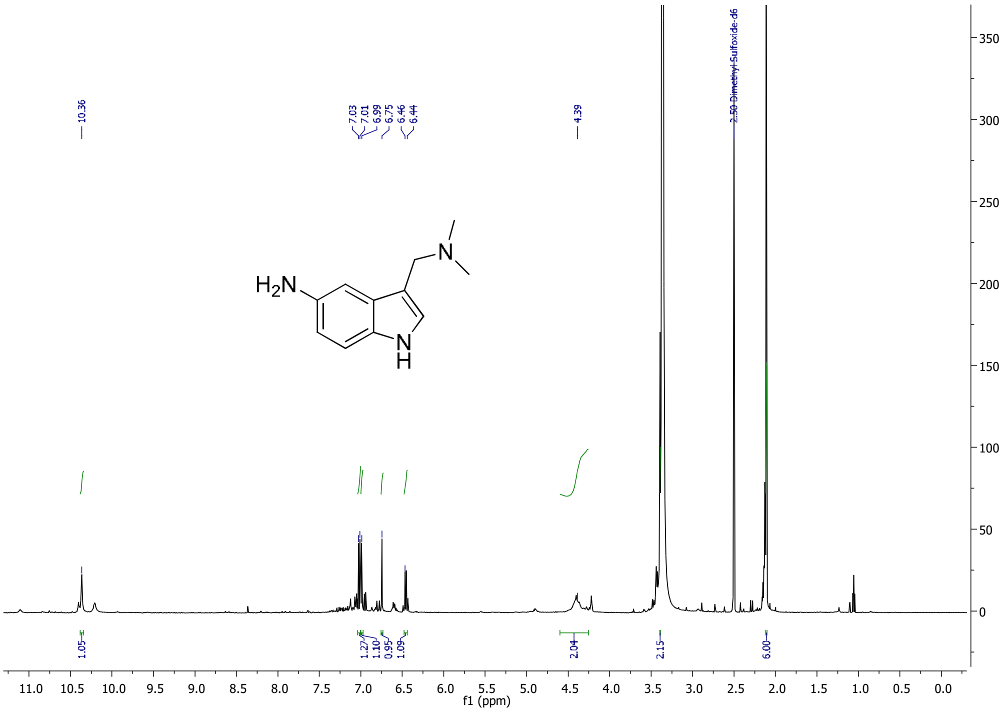

N1-((5-amino-1H-indol-3-yl)methyl)-N2,N2-diethylethane-1,2-diamine (11a)

^{1}H NMR (600 MHz, DMSO-d$_6$)

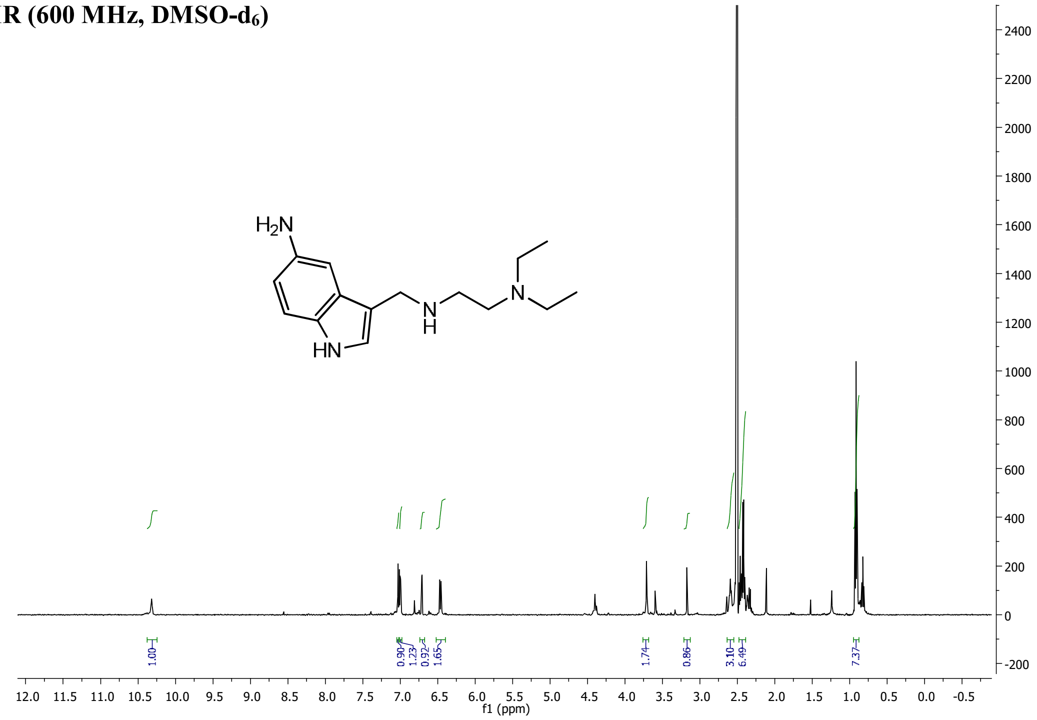

N1,N1-dimethyl-N2-((5-nitro-1H-indol-3-yl)methyl)ethane-1,2-diamine (10)

^{1}H NMR (600 MHz, DMSO-d$_6$)

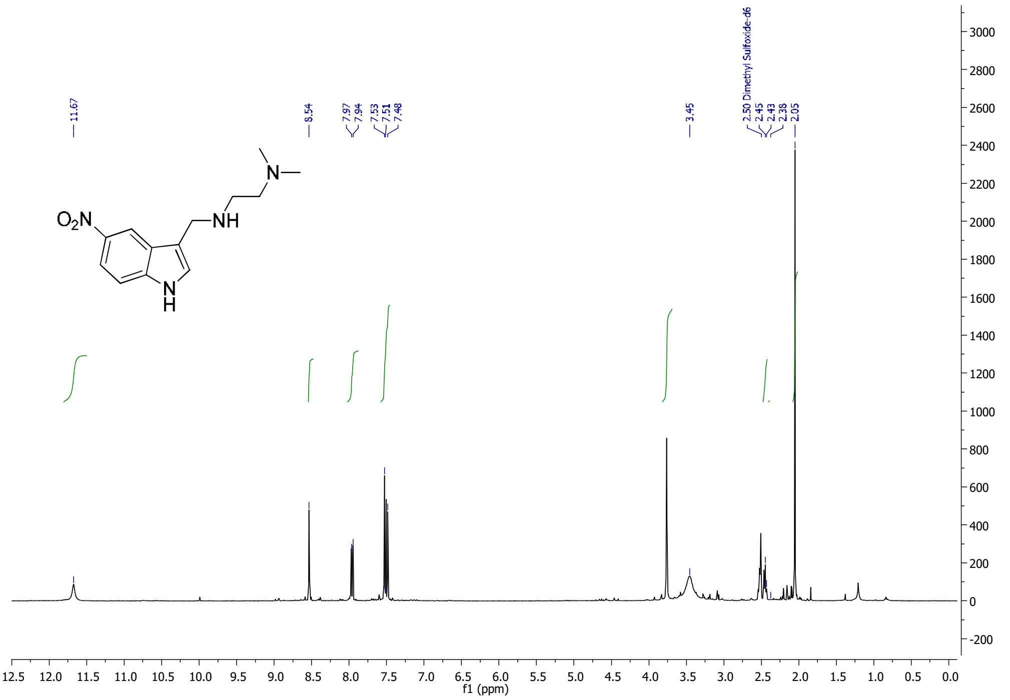

^{13}C NMR (151 MHz, DMSO-d$_6$)

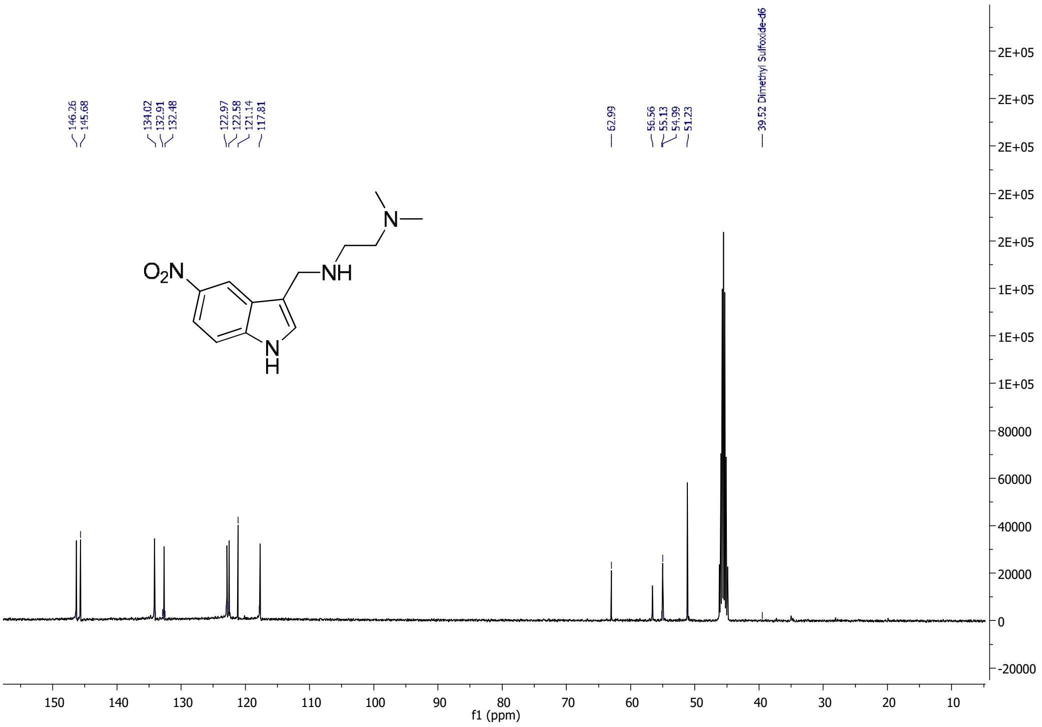

N1,N1-diethyl-N2-((5-nitro-1H-indol-3-yl)methyl)ethane-1,2-diamine (11)

^{1}H-NMR (600 MHz, DMSO-D$_6$)

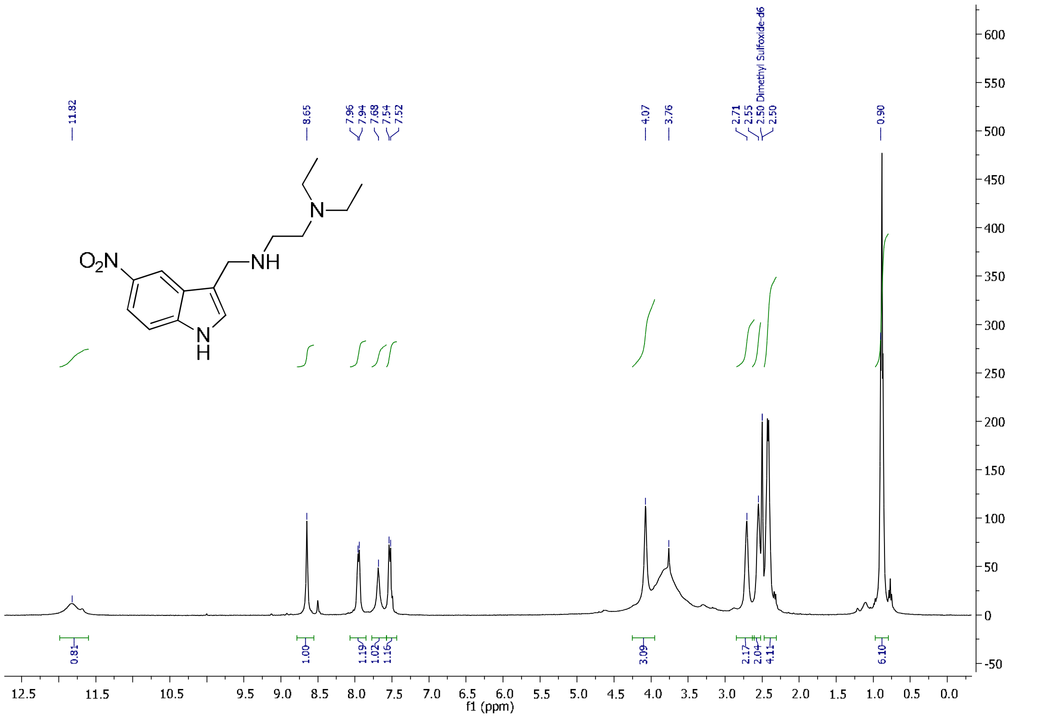

^{13}C NMR (151 MHz, DMSO-d$_6$)

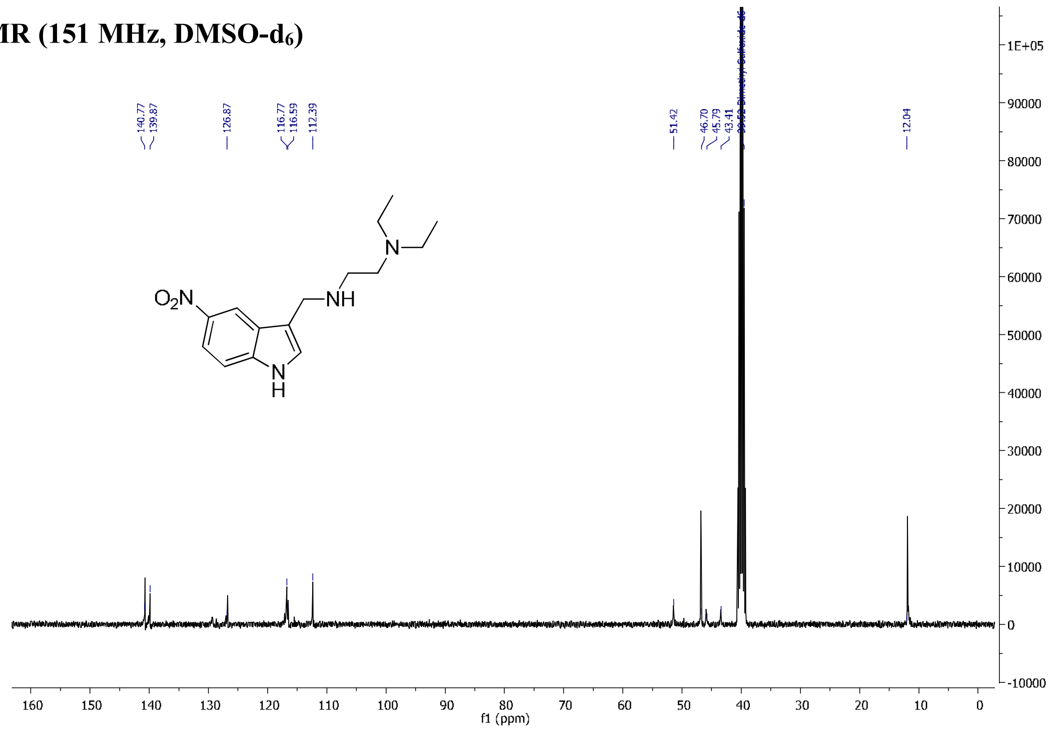

N-((5-nitro-1H-indol-3-yl)methyl)-2-(pyrrolidin-1-yl)ethanamine (12)

^{1}H-NMR (600 MHz, DMSO-D$_6$)

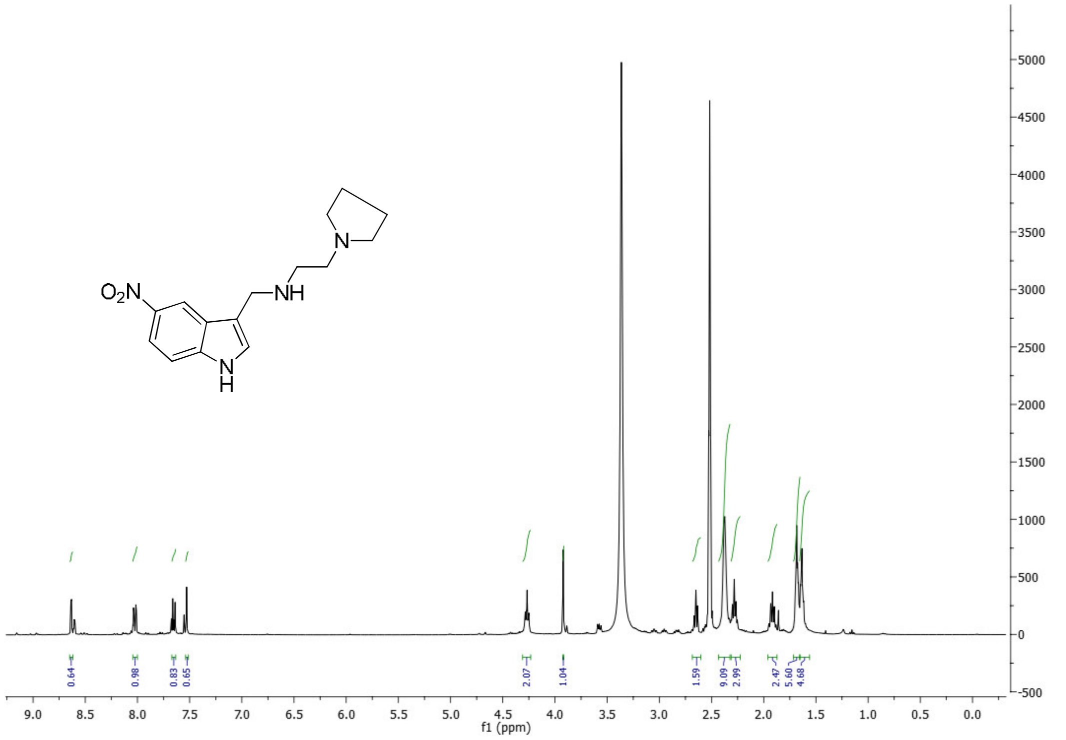

^{13}C NMR (151 MHz, DMSO-d$_6$)

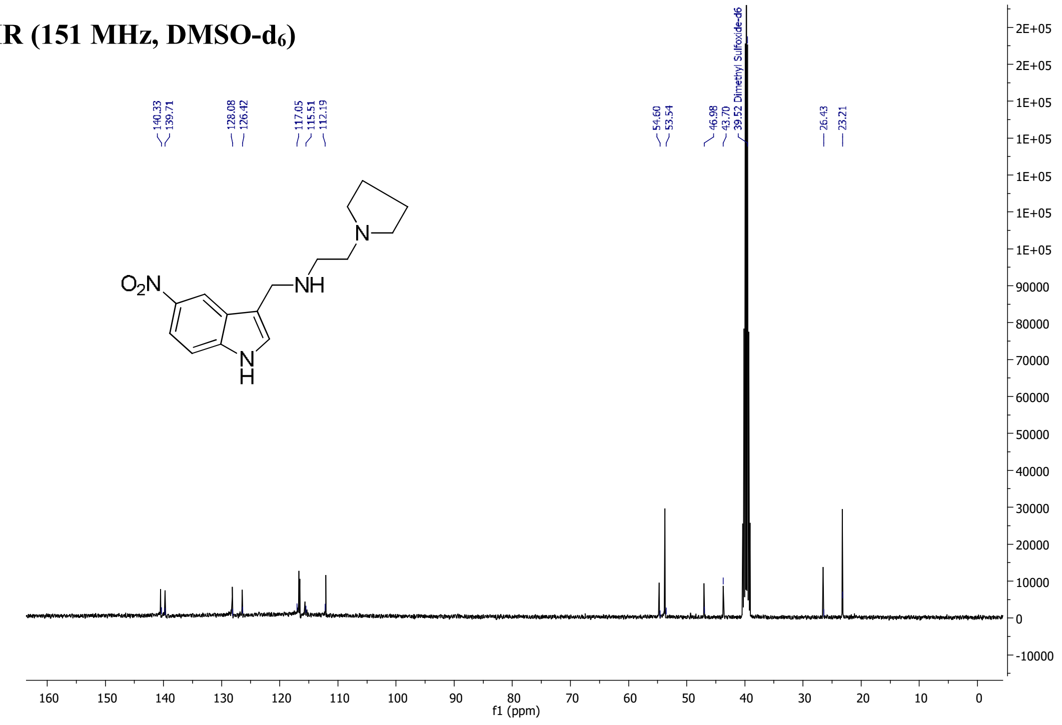

N1-(2-aminoethyl)-N2-(2-((2-(((5-nitro-1H-indol-3-yl)methyl)amino)ethyl)amino)ethyl)ethane-1,2-diamine (13)

1H NMR (600 MHz, D_2O)

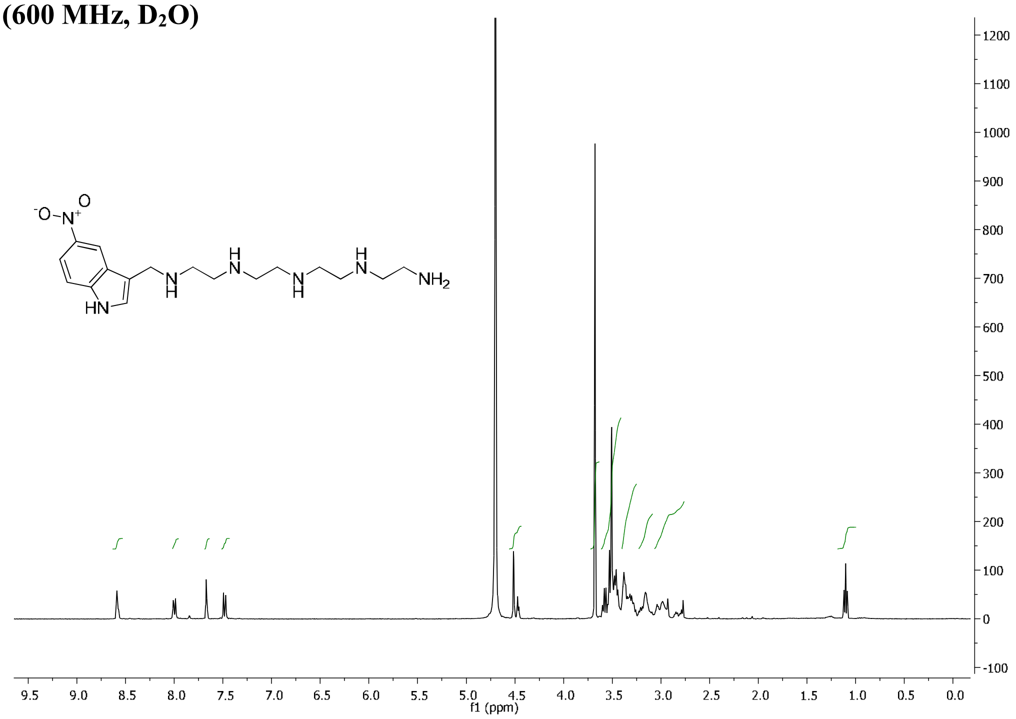

^{13}C NMR (151 MHz, D_2O)

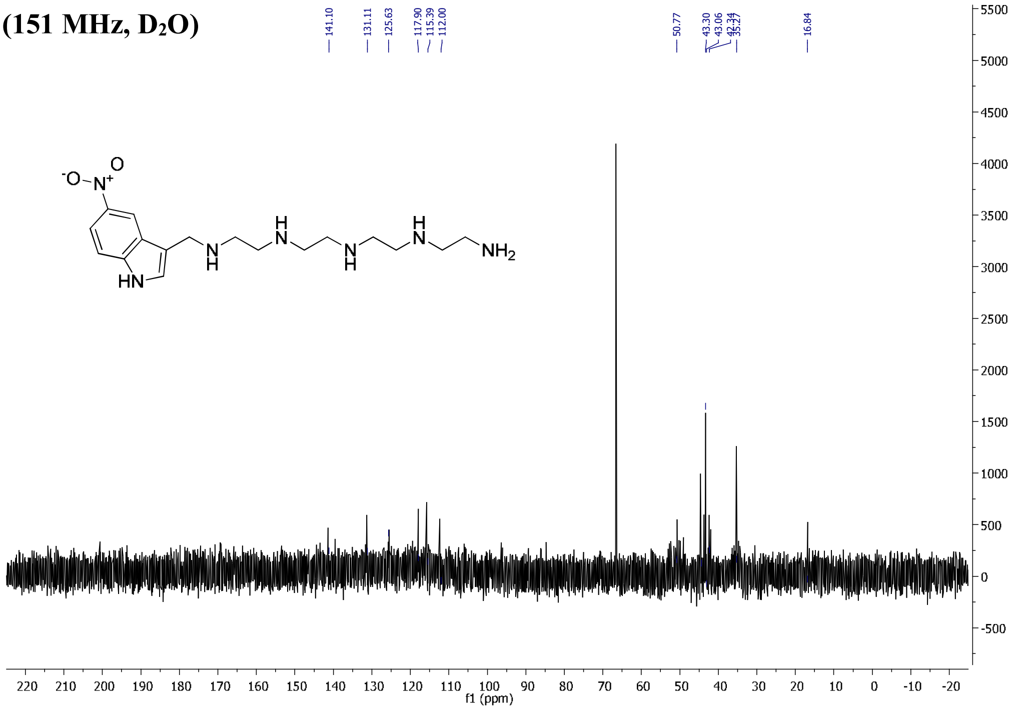

1-(3-azidopropyl)-5-nitro-1H-indole (14)

^{1}H-NMR (600 MHz, DMSO-D$_6$)

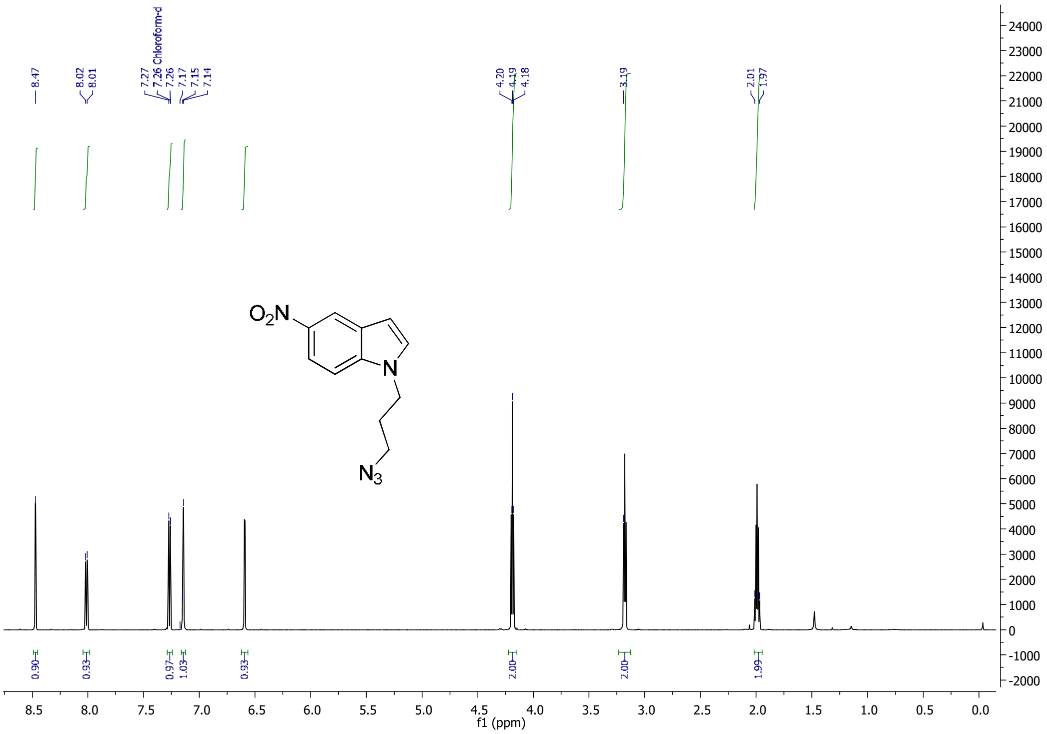

^{13}C NMR (151 MHz, DMSO-d$_6$)

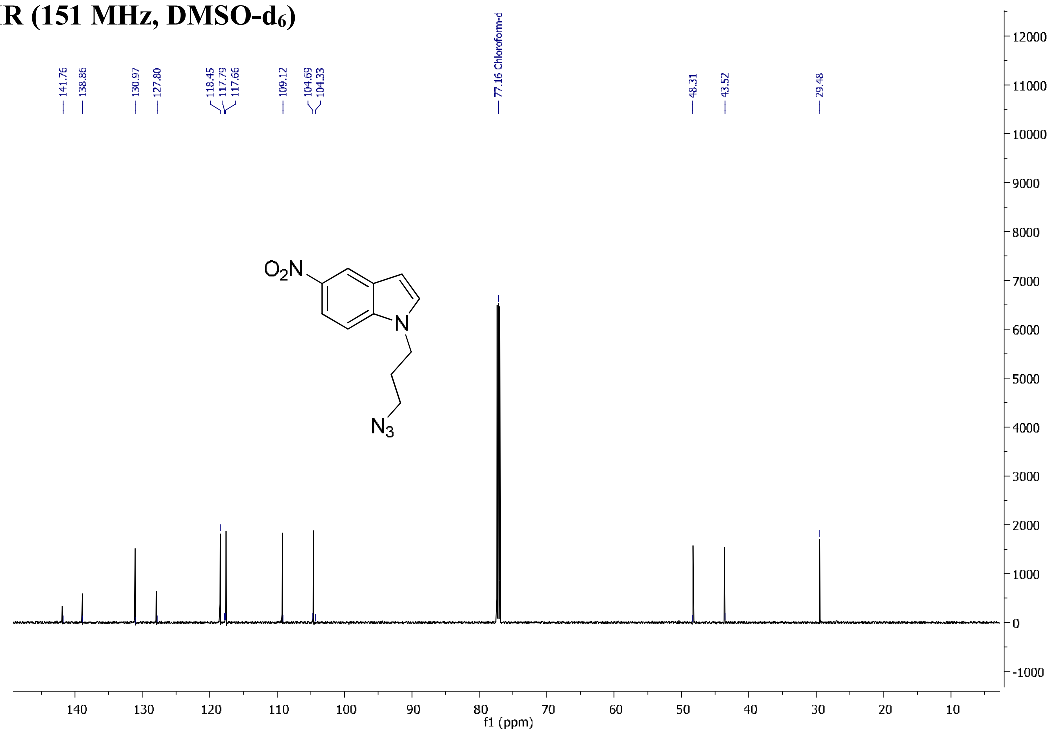

1-(3-azidopropyl)-5-nitro-1H-indole-3-carbaldehyde (15)

^{1}H-NMR (600 MHz, DMSO-D$_6$)

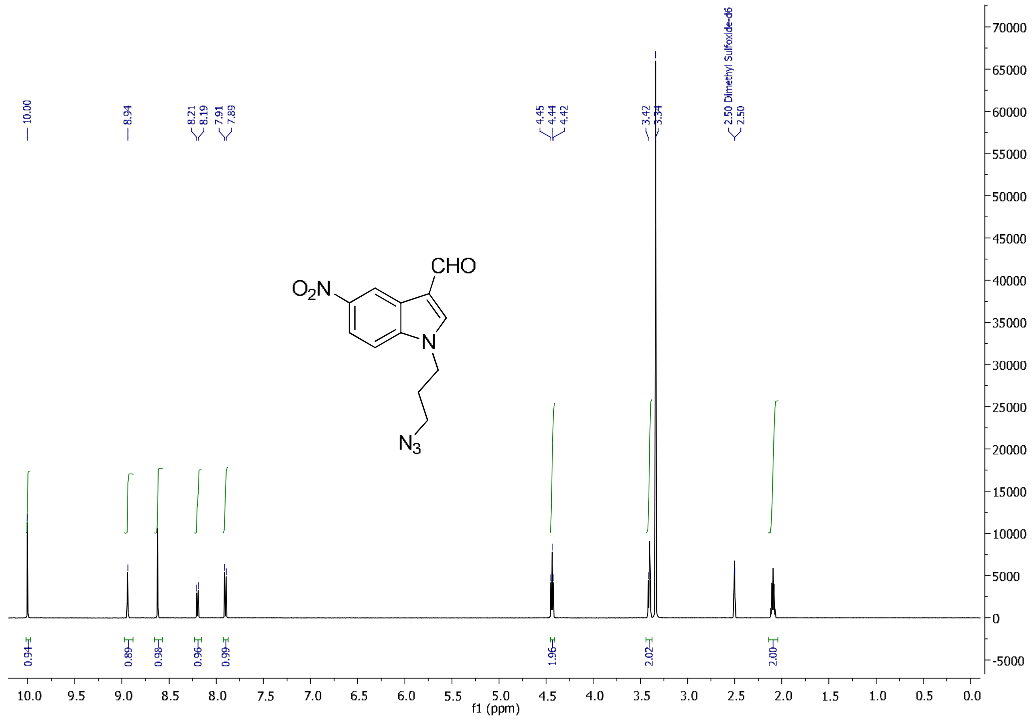

^{13}C NMR (151 MHz, DMSO-d$_6$)

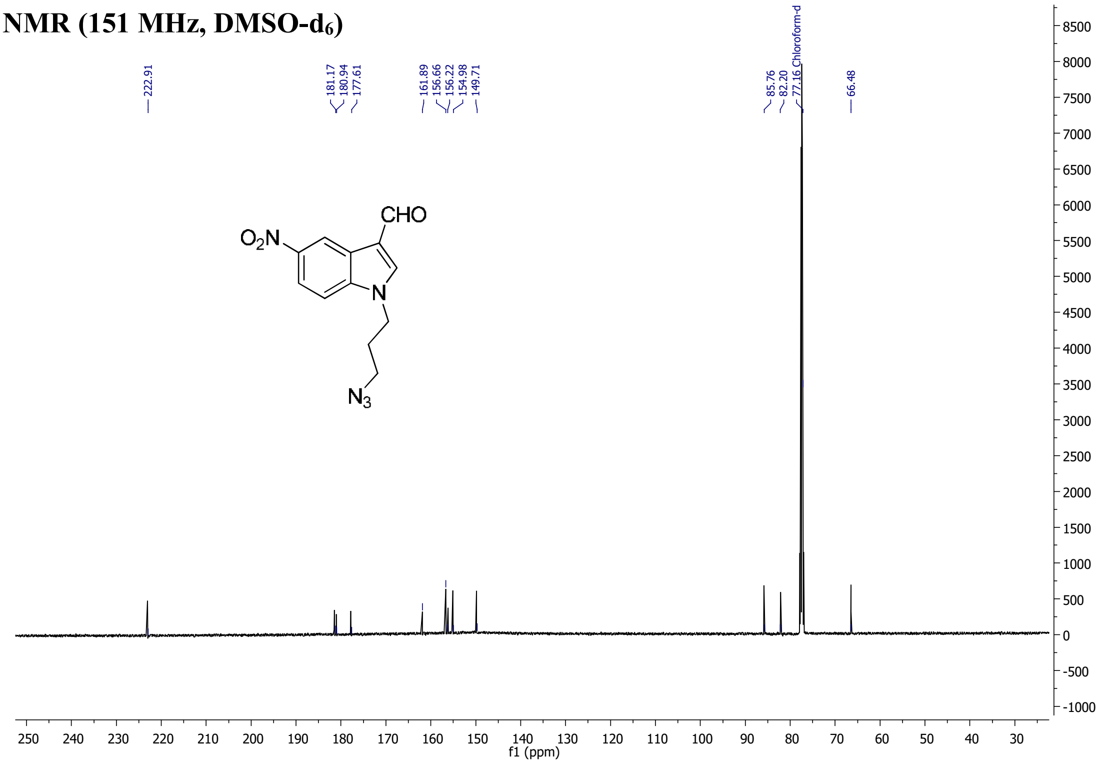

1-(1-(3-azidopropyl)-5-nitro-1H-indol-3-yl)-N,N-dimethylmethanamine (16a)

^{1}H-NMR (600 MHz, DMSO-D$_6$)

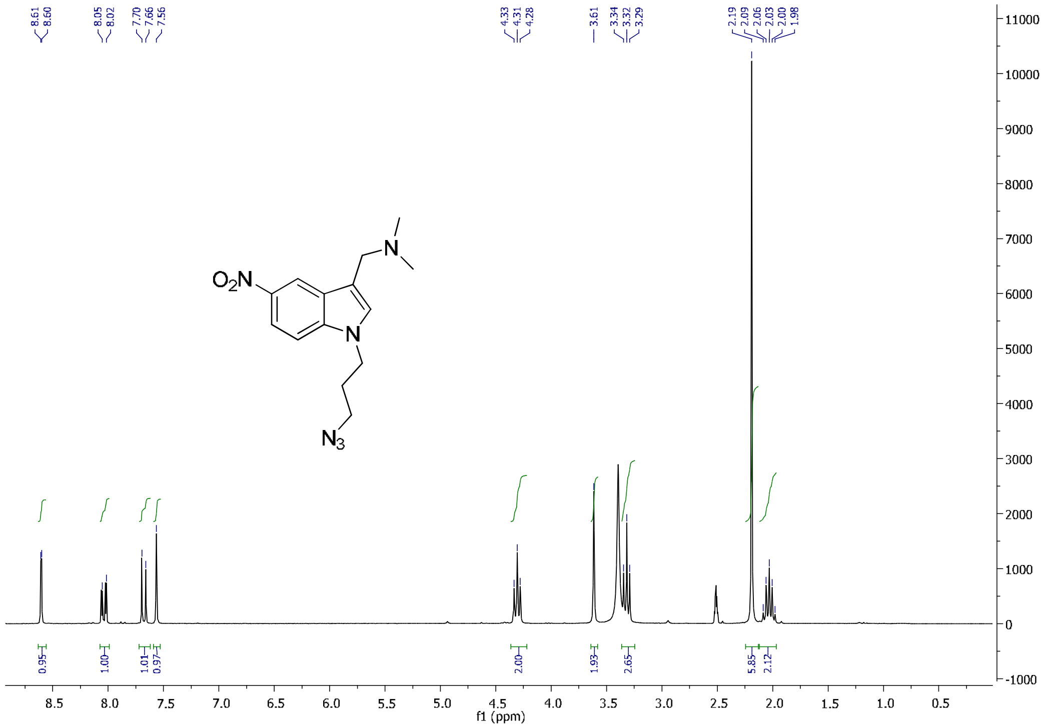

^{13}C NMR (151 MHz, DMSO-d$_6$)

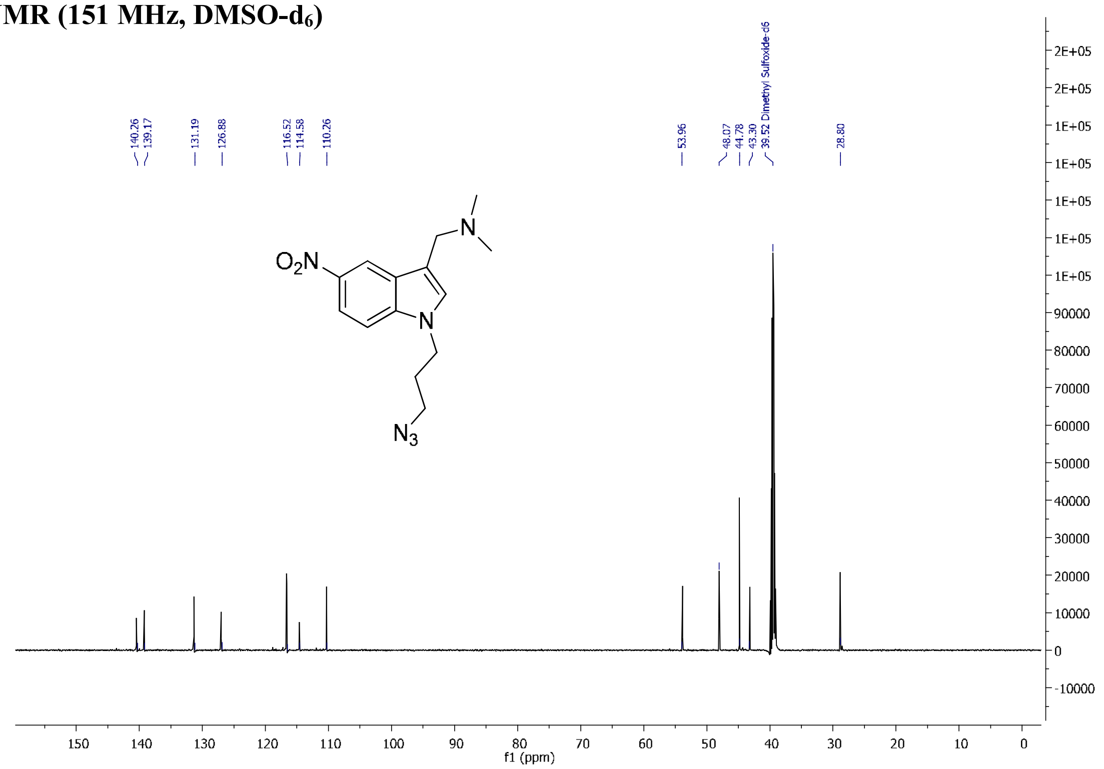

N,N-dimethyl-N'-[[5-nitro-1-(3-Azidopropyl)-1H-indole]methyl]-1,2-Ethanediamine (16b)

^{1}H-NMR (600 MHz, DMSO-D$_6$)

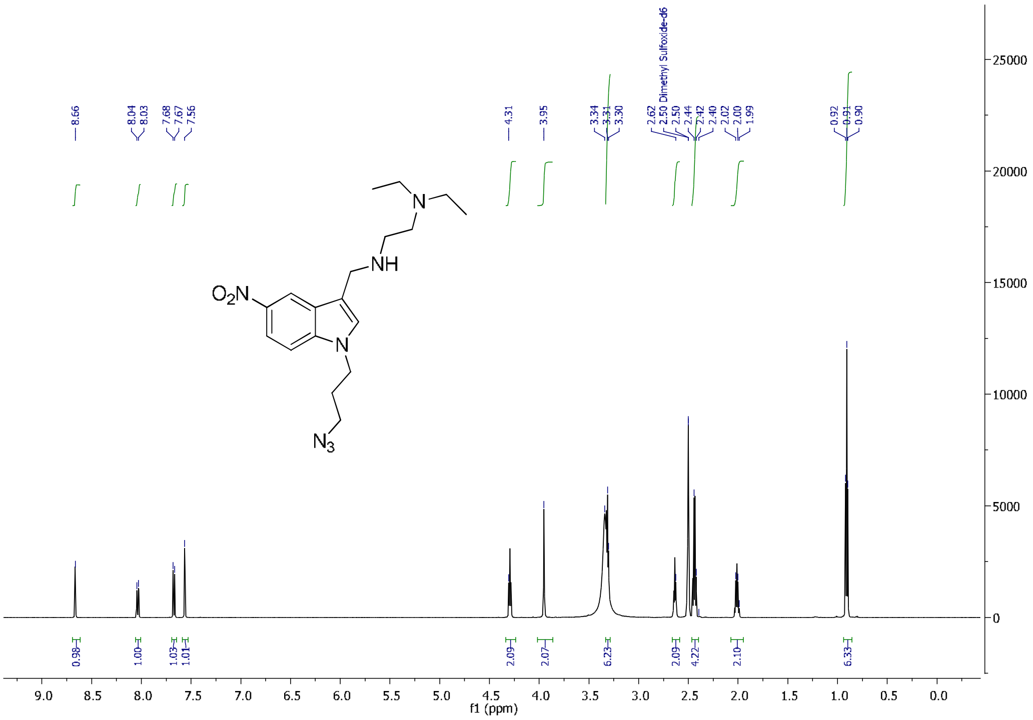

^{13}C NMR (151 MHz, DMSO-d$_6$)

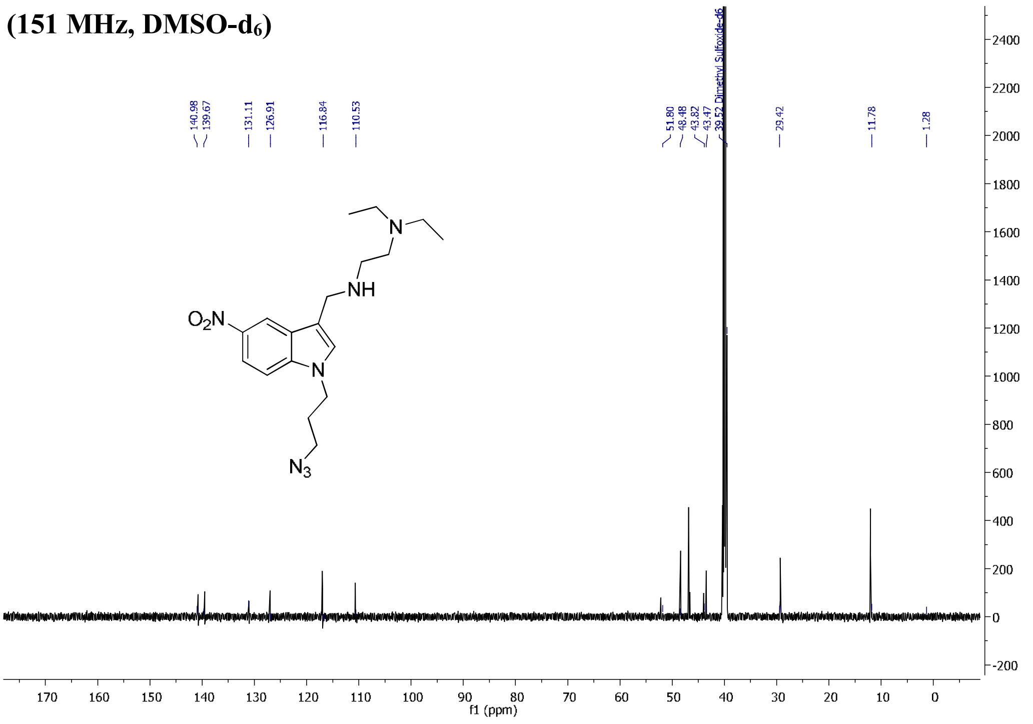

5-nitro-1-(prop-2-yn-1-yl)-1H-indole (19a)

^{1}H NMR (600 MHz, $CDCl_3$)

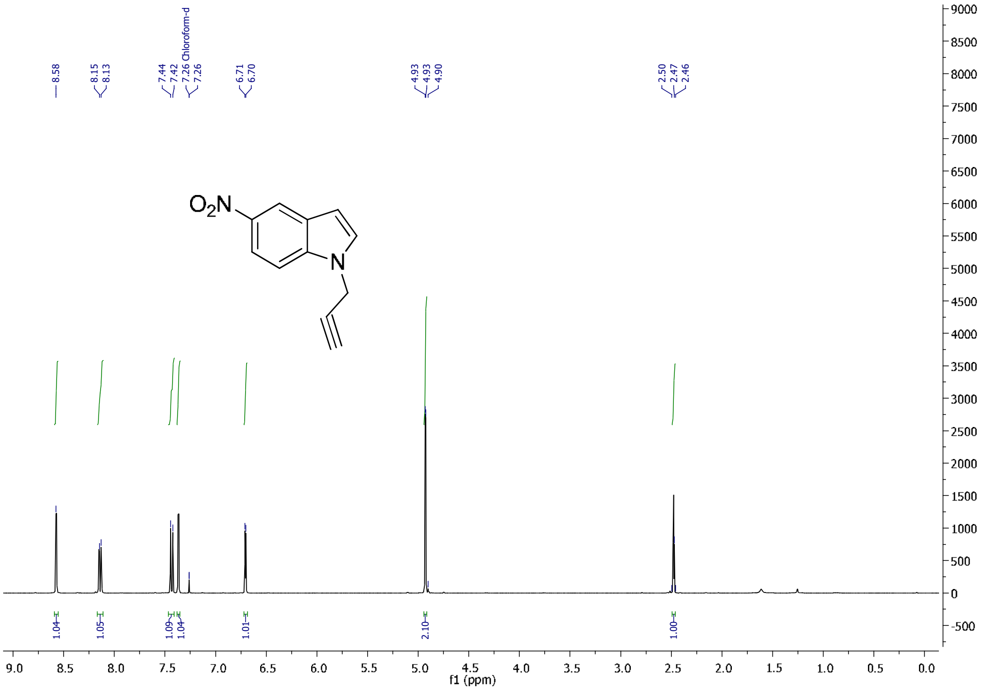

^{13}C NMR (151 MHz, $CDCl_3$)

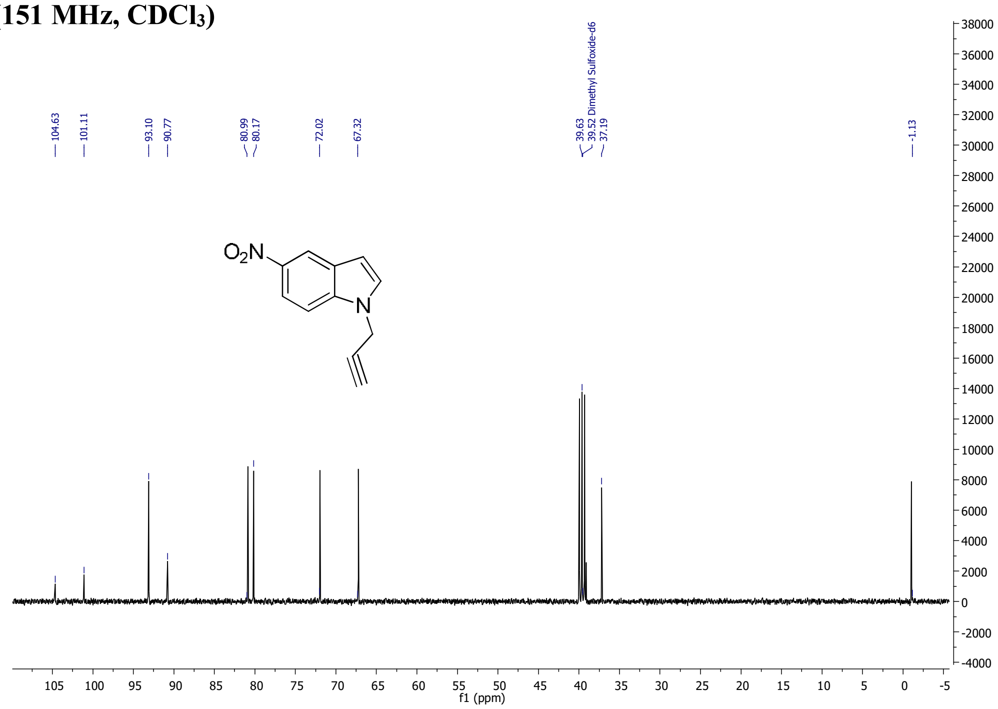

1-(but-3-yn-1-yl)-5-nitro-1H-indole (19b)

^{1}H-NMR (600 MHz, DMSO-d_6)

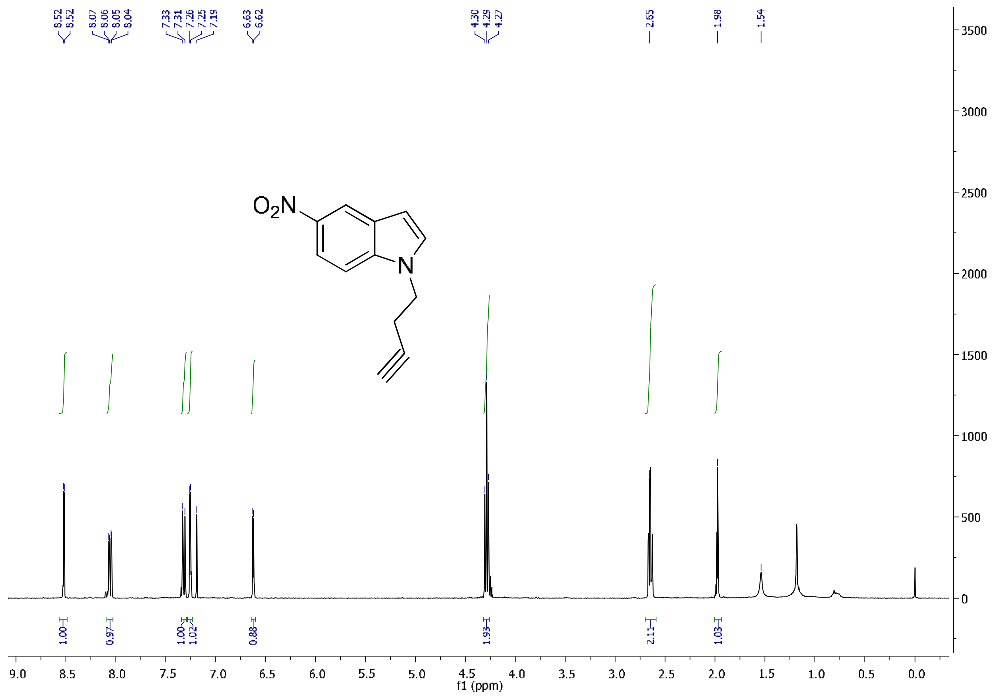

^{13}C NMR (151 MHz, DMSO-d6)

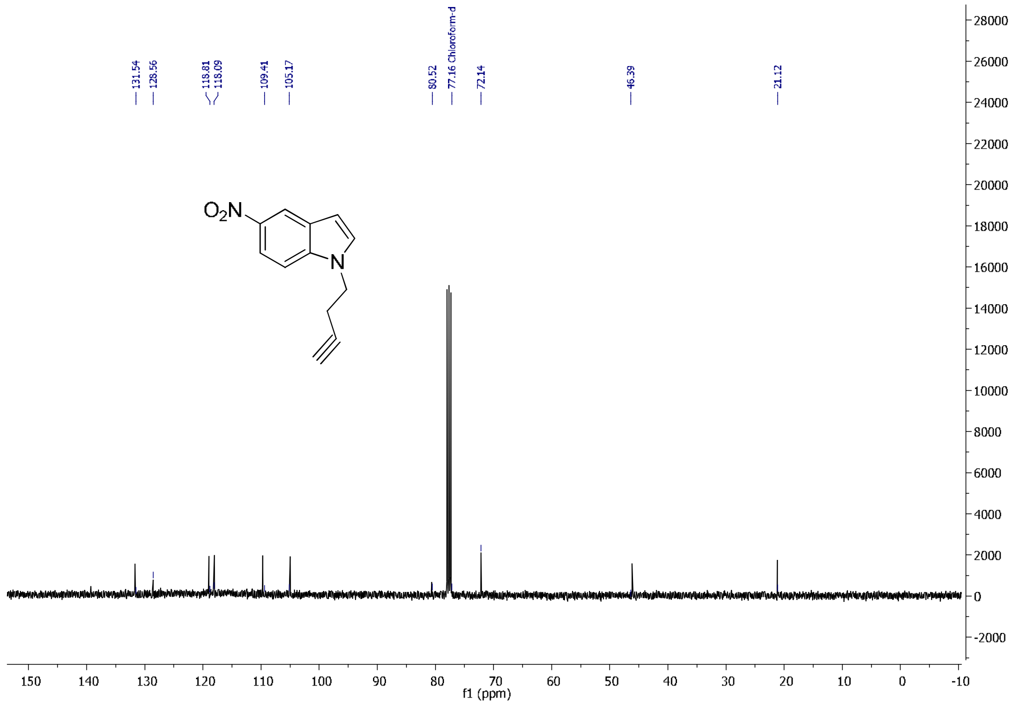

5-nitro-1-(prop-2-yn-1-yl)-1H-indole-3-carbaldehyde (20)

^{1}H-NMR (600 MHz, DMSO-d$_6$)

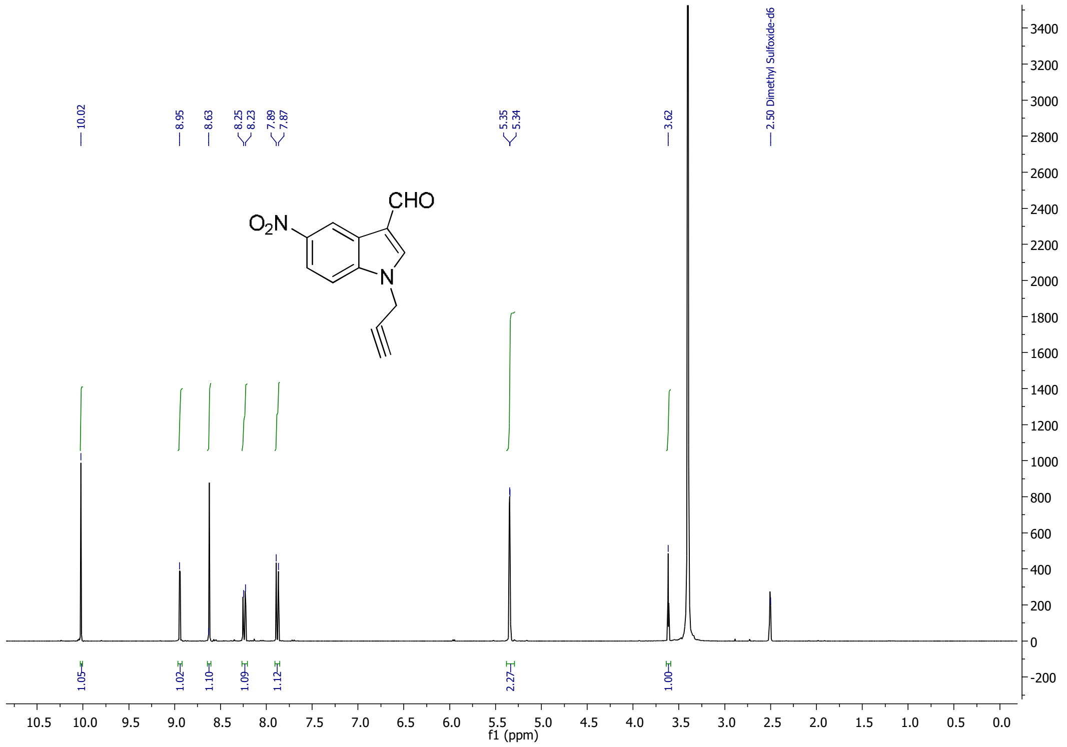

^{13}C NMR (151 MHz, DMSO-d$_6$)

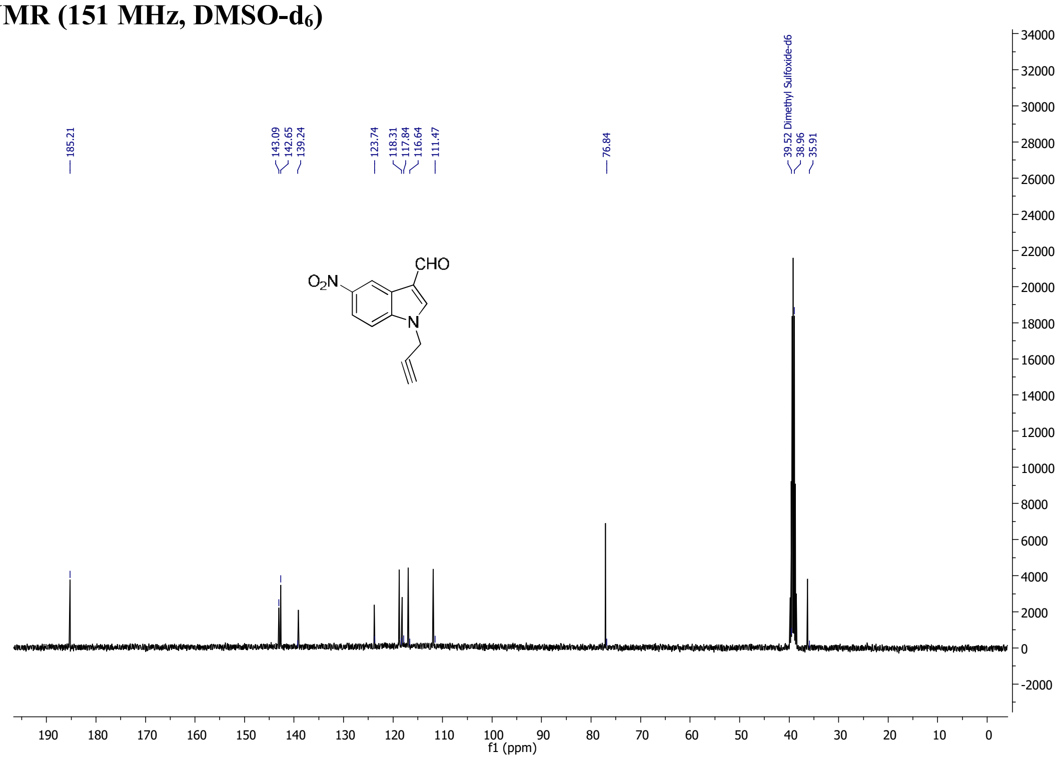

N,N-dimethyl-1-(5-nitro-1-(prop-2-yn-1-yl)-1H-indol-3-yl)methanamine (21a)

^{1}H-NMR (600 MHz, DMSO-d$_6$)

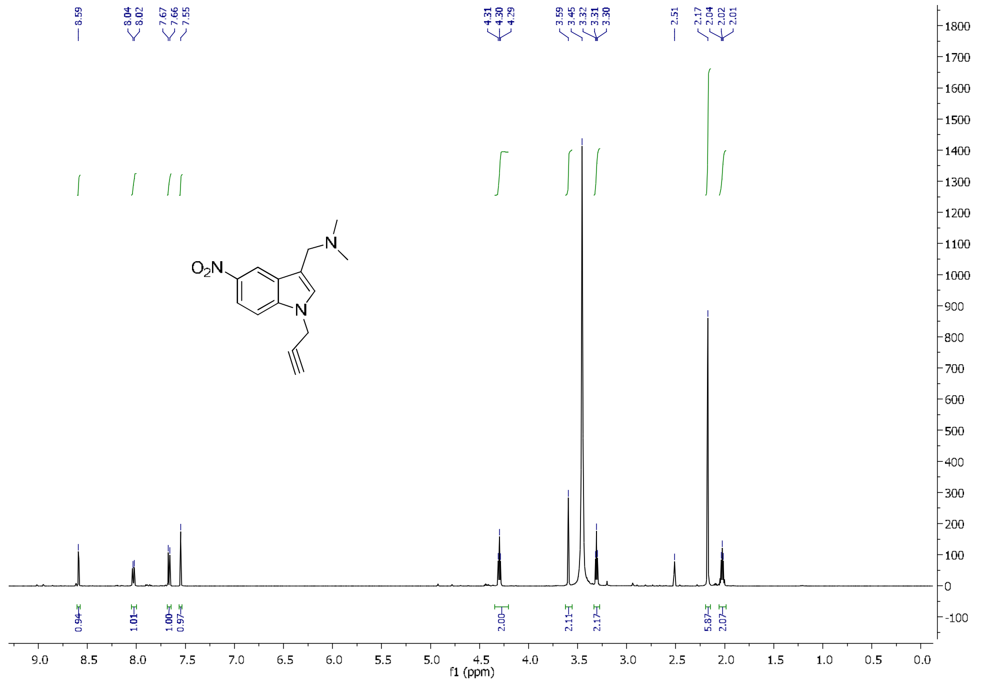

^{13}C NMR (151 MHz, DMSO-d$_6$)

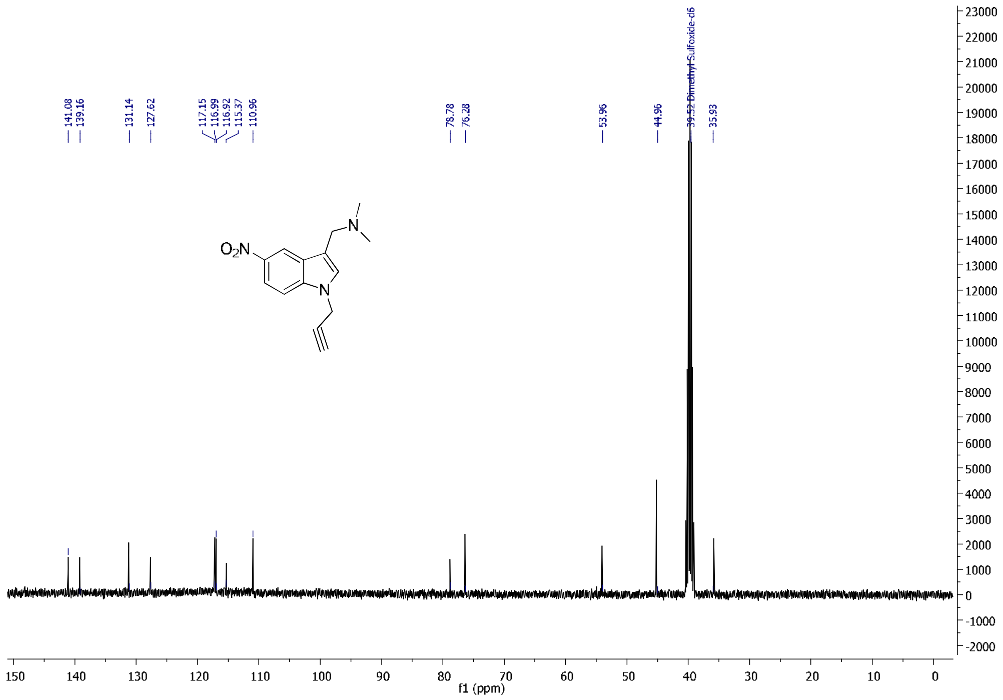

N1,N1-diethyl-N2-((5-nitro-1-(prop-2-yn-1-yl)-1H-indol-3-yl)methyl)ethane-1,2-diamine (21b):

^{1}H-NMR (600 MHz, DMSO-d$_6$)

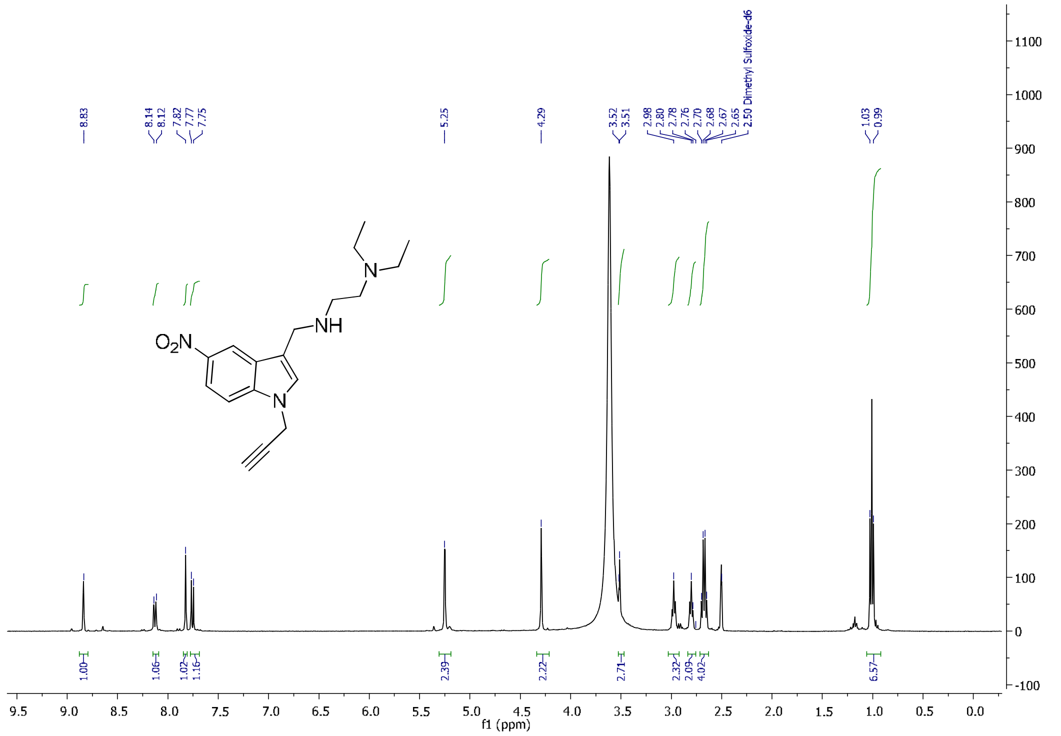

^{13}C NMR (151 MHz, DMSO-d$_6$)

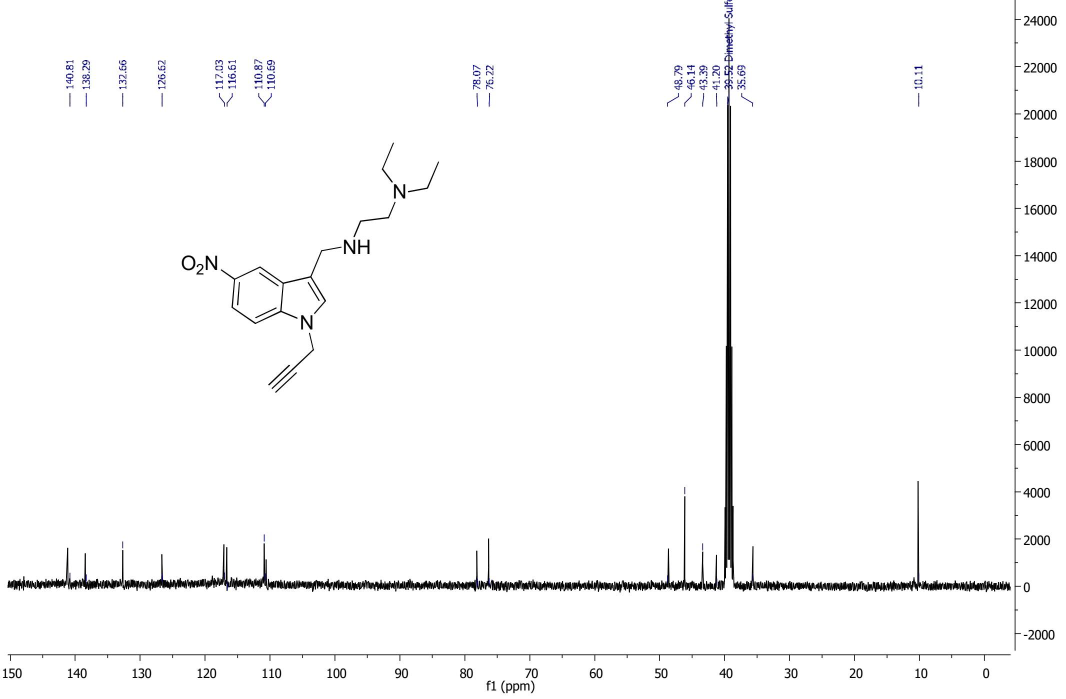

1H-indole-3-carbaldehyde (2a)

^{1}H NMR (600 MHz, CDCl$_3$)

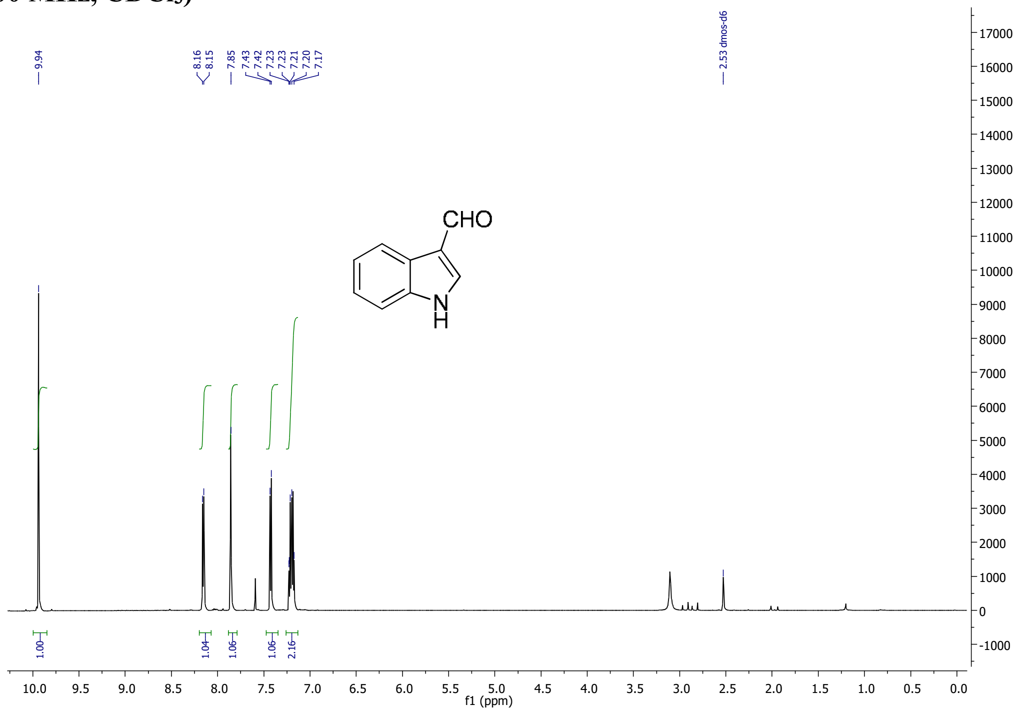

^{13}C NMR (151 MHz, CDCl$_3$)

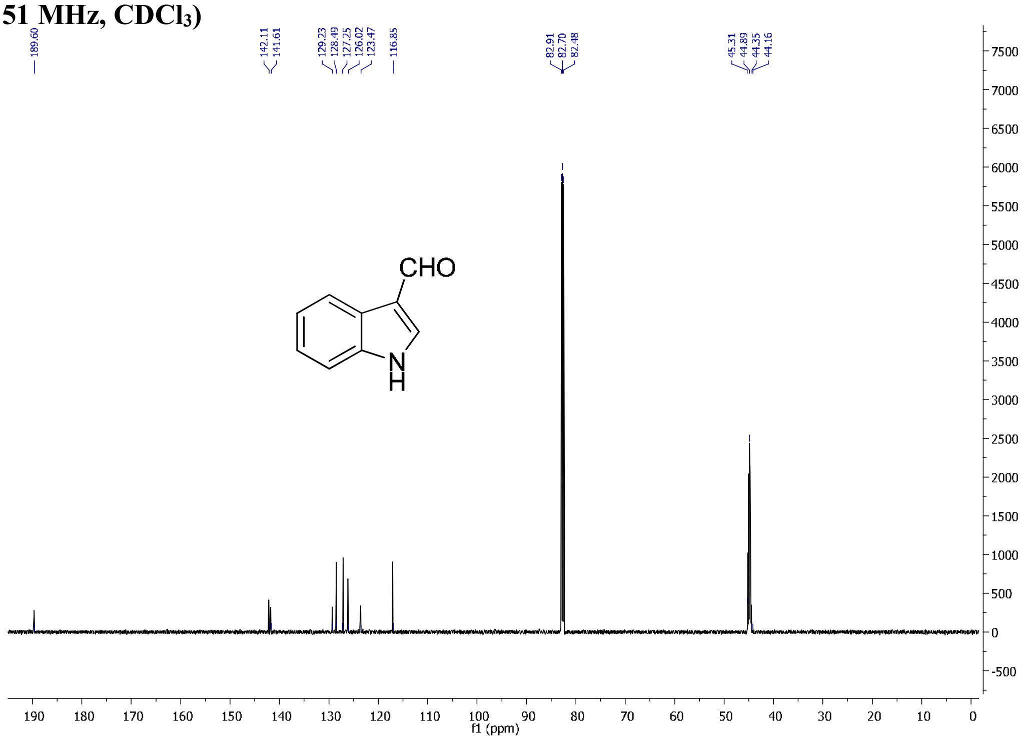

1-(1H-indol-3-yl)-N, N-dimethylmethanamine (3a)

^{1}H-NMR (600 MHz, DMSO-d$_6$)

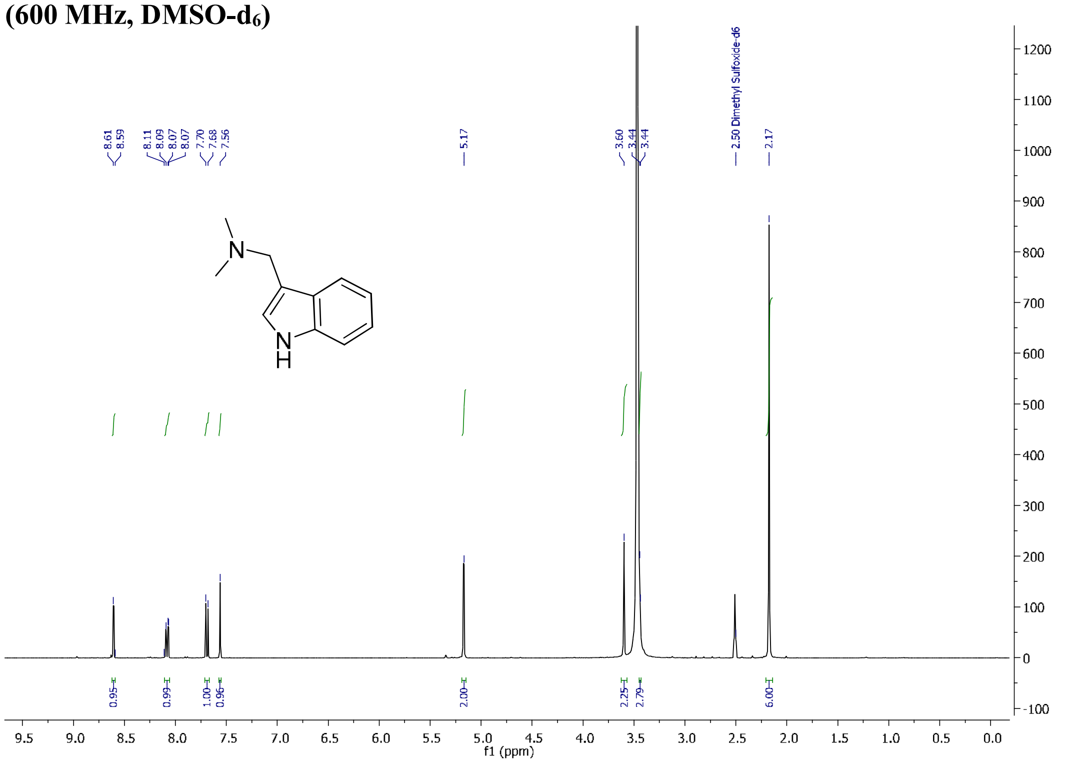

^{13}C NMR (151 MHz, DMSO-d6)

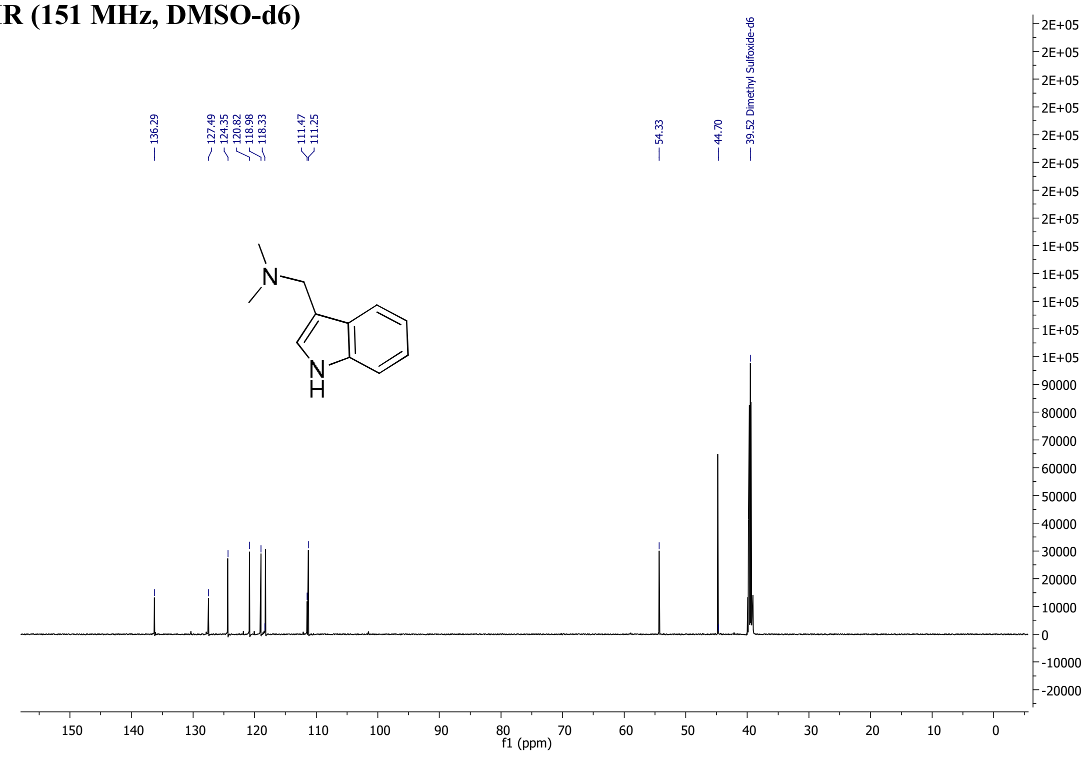

N1-((1H-indol-3-yl)methyl)-N2-(2-((2-((2-((2 aminoethyl)amino)ethyl)amino)ethyl)amino)ethyl)ethane-1,2-diamine (3b)

1H-NMR (600 MHz, D_2O)

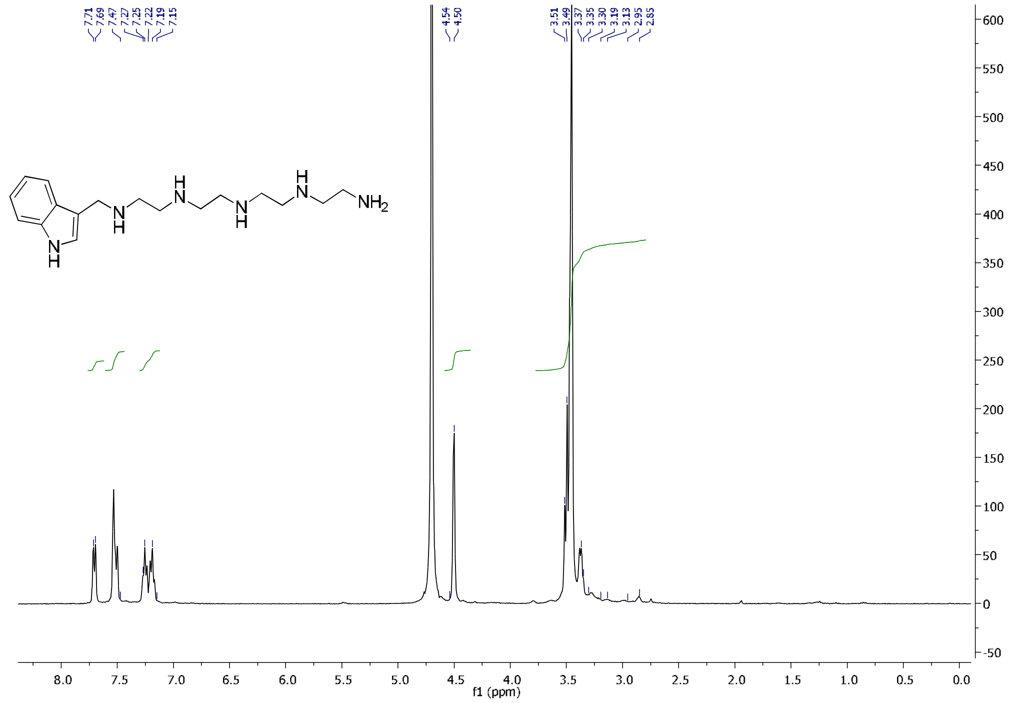

^{13}C NMR (151 MHz, D_2O)

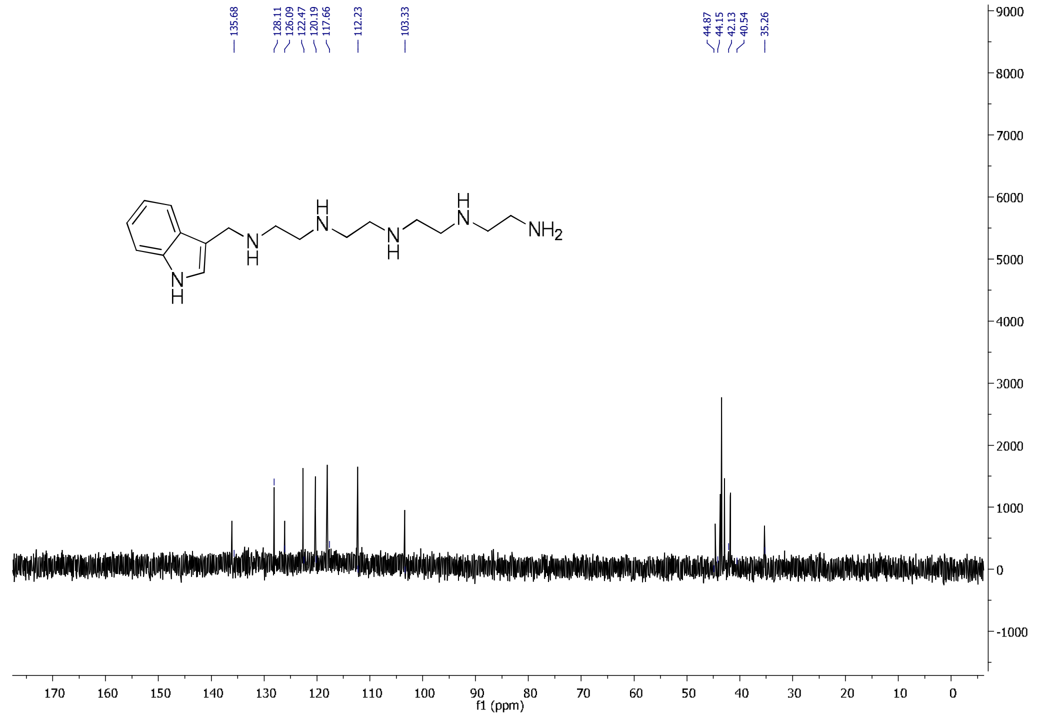

1-(1H-indazol-3-yl)-N,N-dimethylmethanamine (23a)

^{1}H-NMR (600 MHz, DMSO-d$_6$)

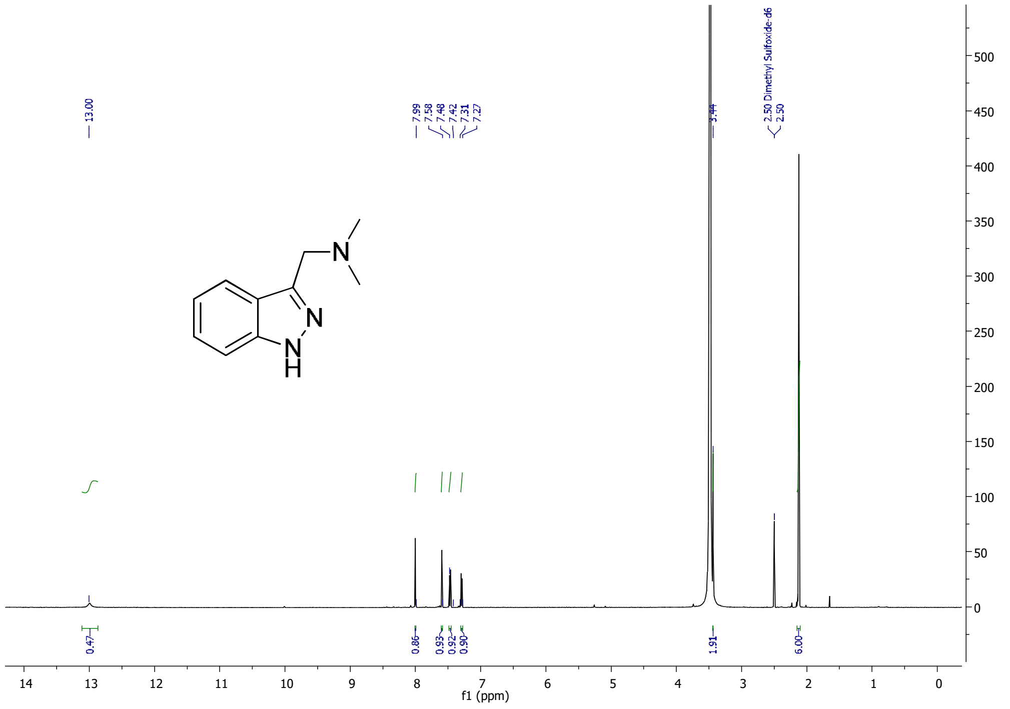

^{13}C NMR (151 MHz, DMSO-d6)

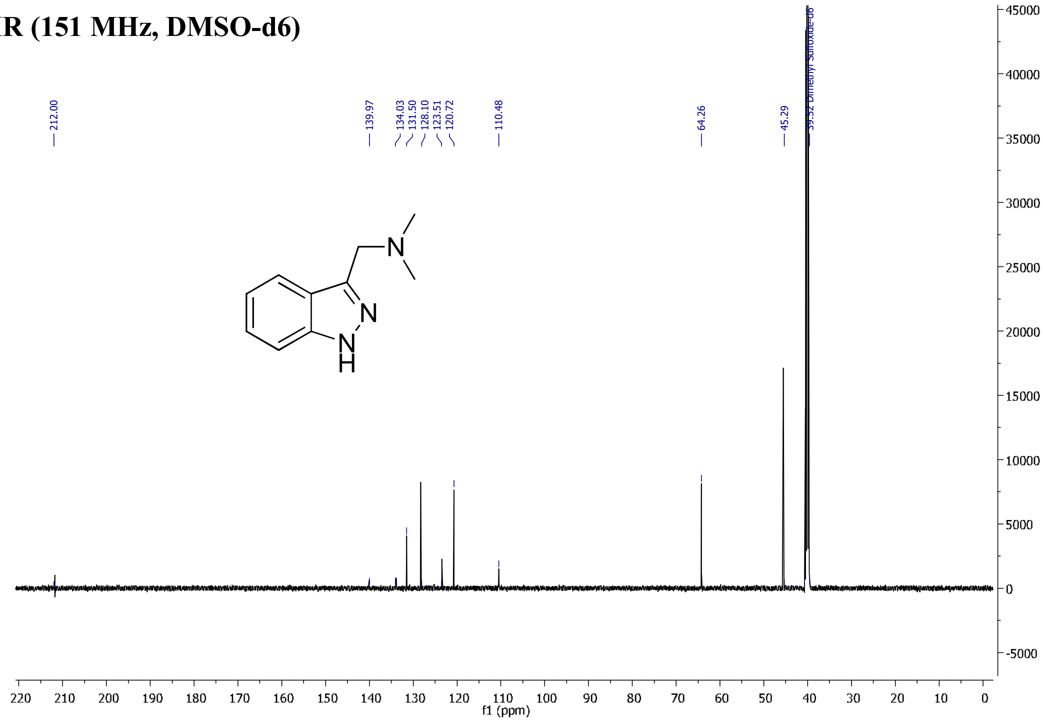

N1-((1H-indazol-3-yl)methyl)-N2-(2-((2-((2-aminoethyl)amino)ethyl)amino)ethyl)ethane-1,2-diamine (23c)

^{1}H-NMR (600 MHz, D_2O)

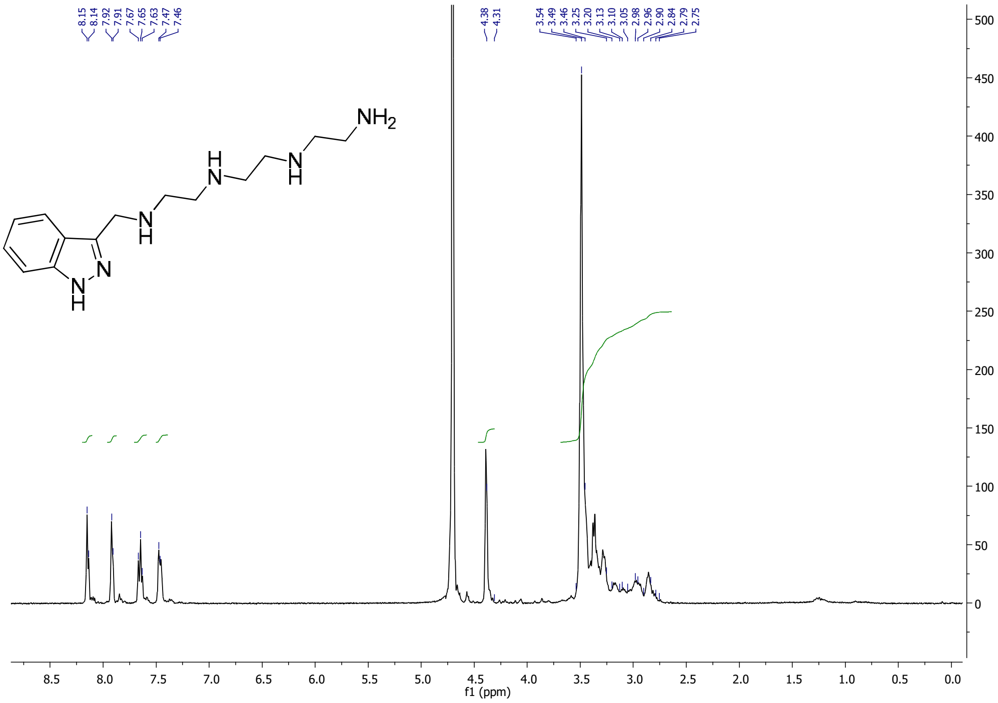

^{13}C NMR (151 MHz, D_2O)

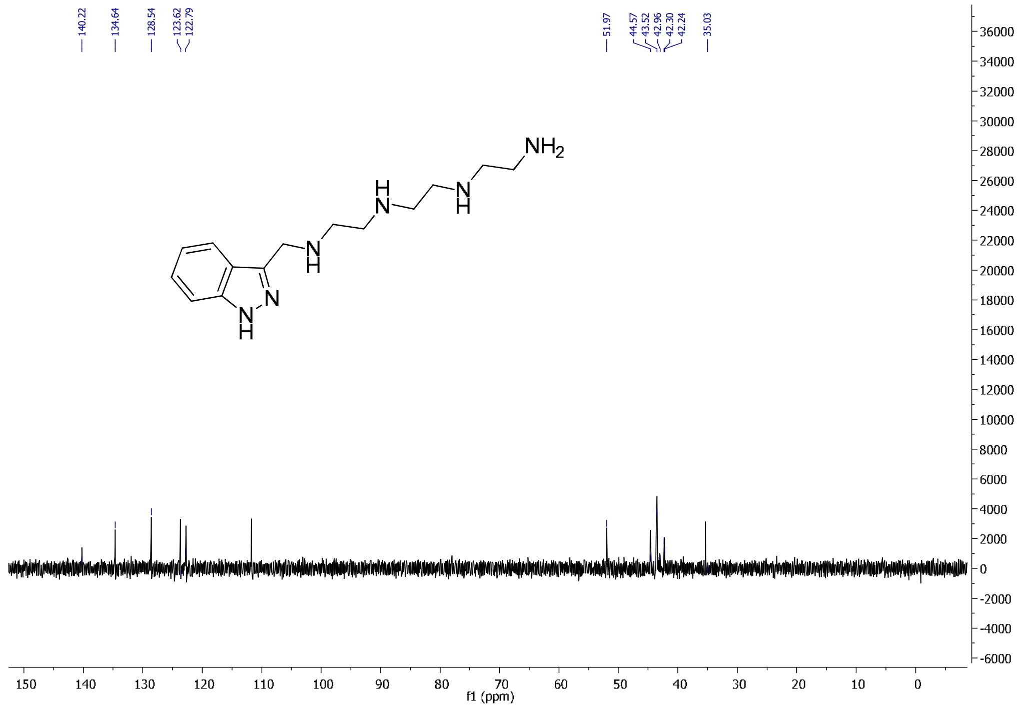

N-((1H-indazol-3-yl)methyl)-2-(pyrrolidin-1-yl)ethanamine (23e)

^{1}H-NMR (600 MHz, DMSO-d$_6$)

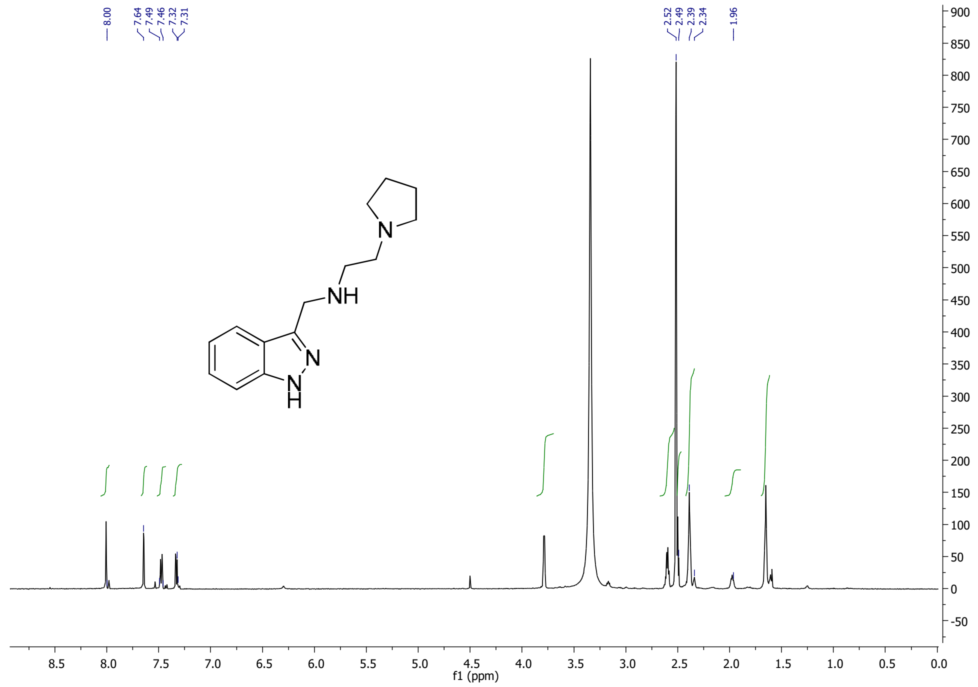

^{13}C NMR (151 MHz, DMSO-d$_6$)

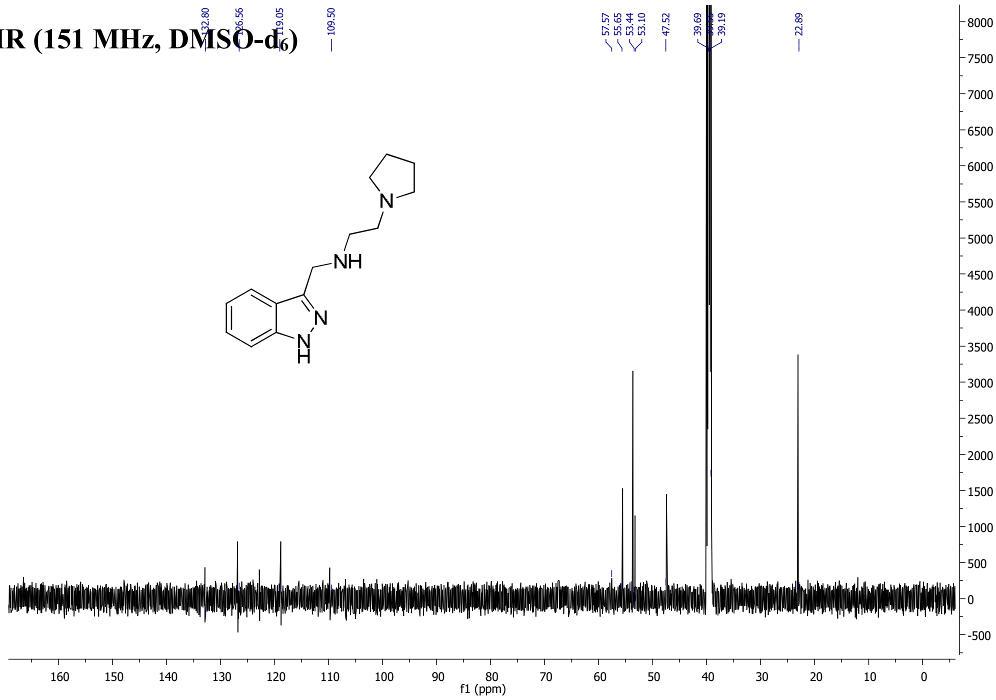

N,N-dimethyl-1-(1H-pyrrolo[2,3-b]pyridin-3-yl)methanamine (24a)

^{1}H NMR (600 MHz, DMSO-d$_6$)

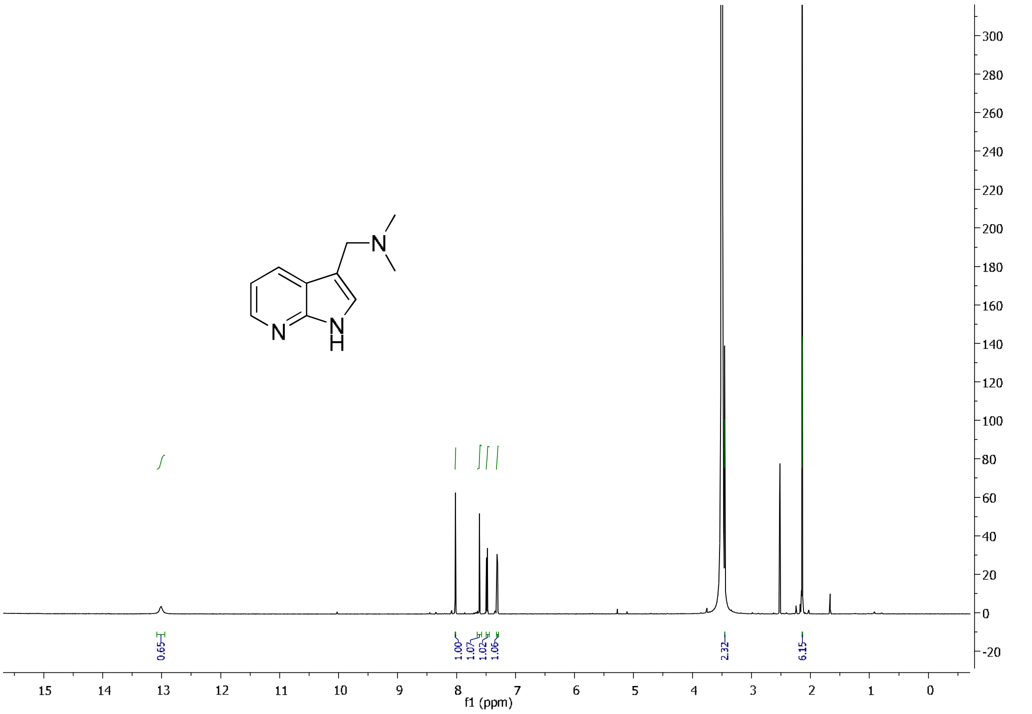

^{13}C NMR (151 MHz, DMSO-d$_6$

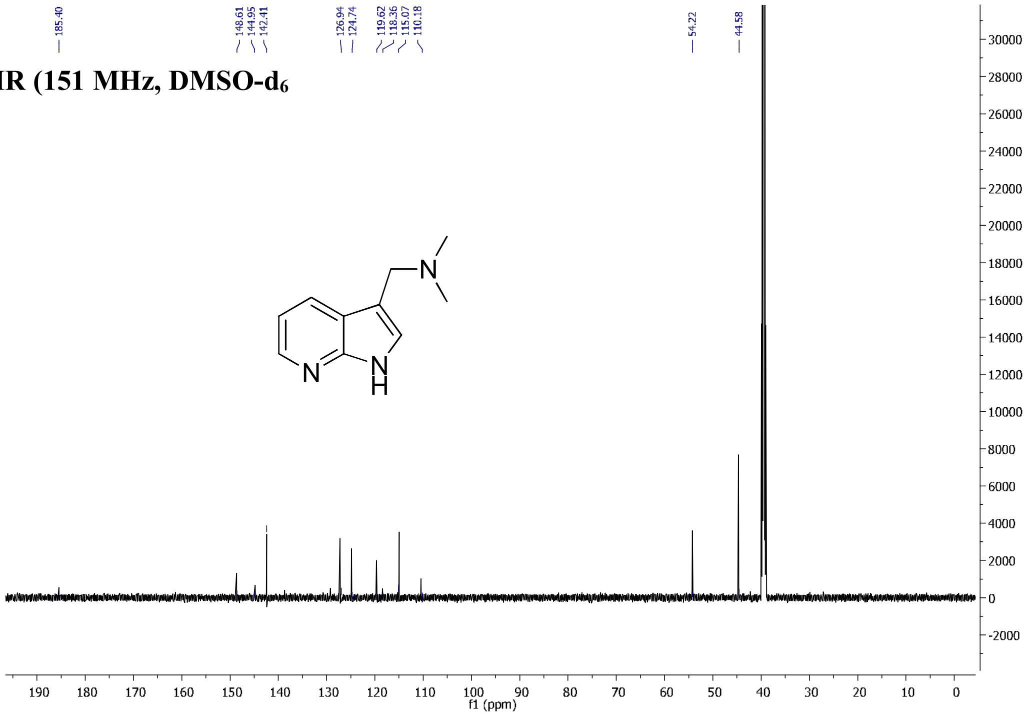

N1-((1H-pyrrolo[2,3-b]pyridin-3-yl)methyl)-N2-(2-((2-((2-aminoethyl)amino)ethyl)amino)ethyl)ethane-1,2-diamine (24b) CRUDE HCl salt compound

^{1}H NMR (600 MHz, D_2O)

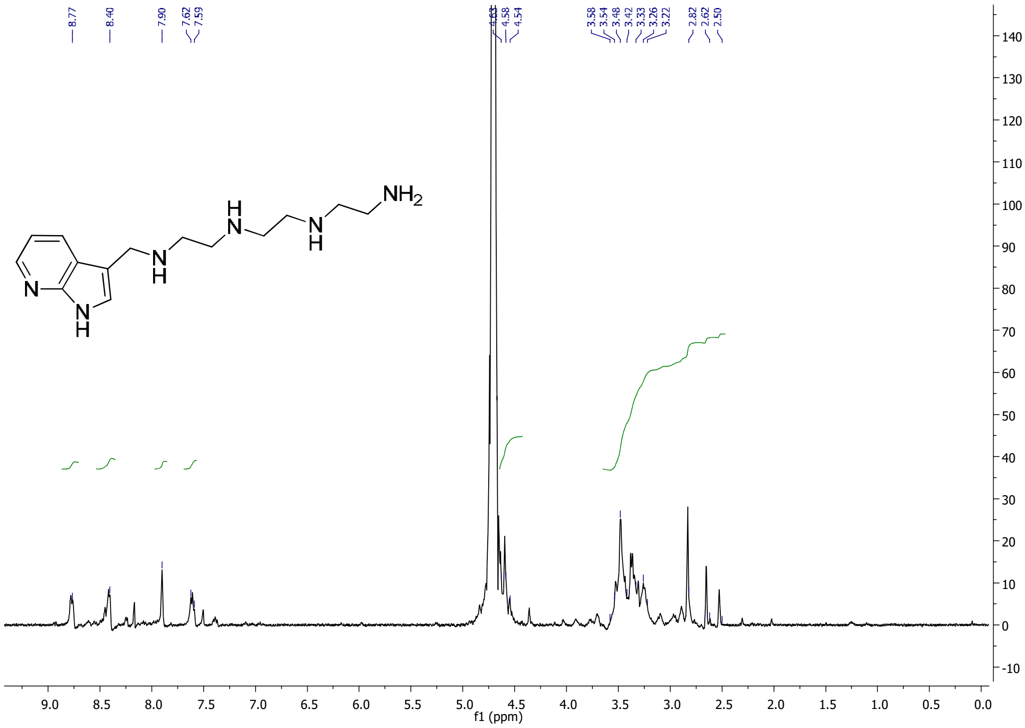

^{13}C NMR (151 MHz, D_2O)

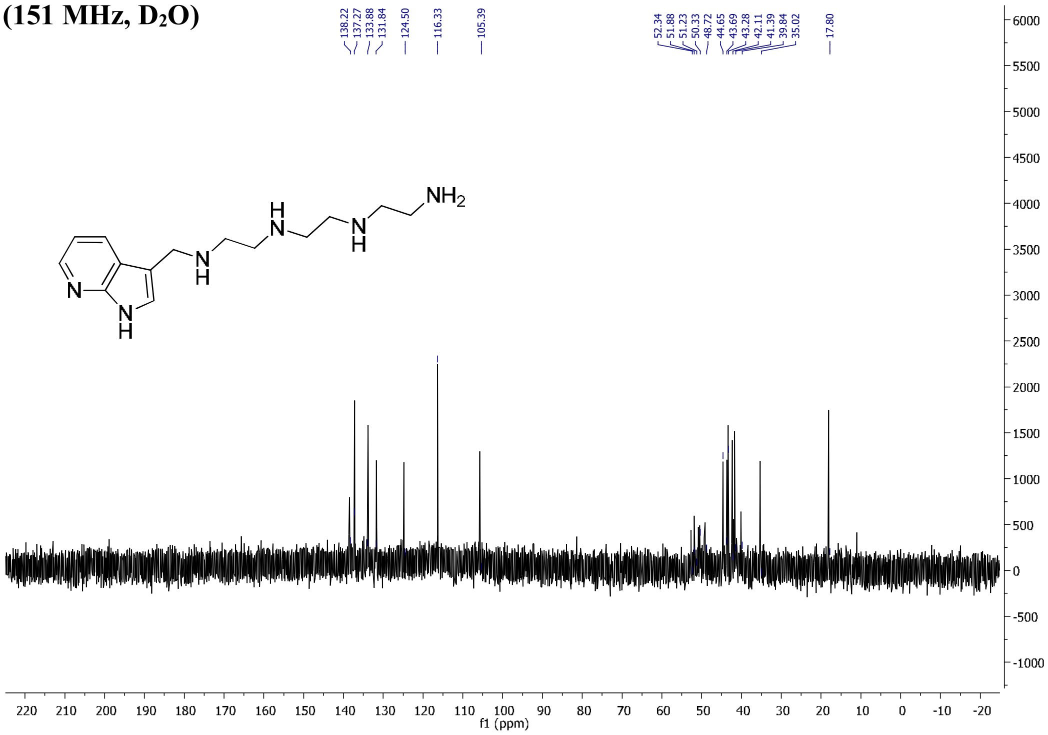

N1-((1H-pyrrolo[2,3-b]pyridin-3-yl)methyl)-N2-(2-((2-aminoethyl)amino)ethyl)ethane-1,2-diamine (24c)

^{1}H NMR (600 MHz, D_2O)

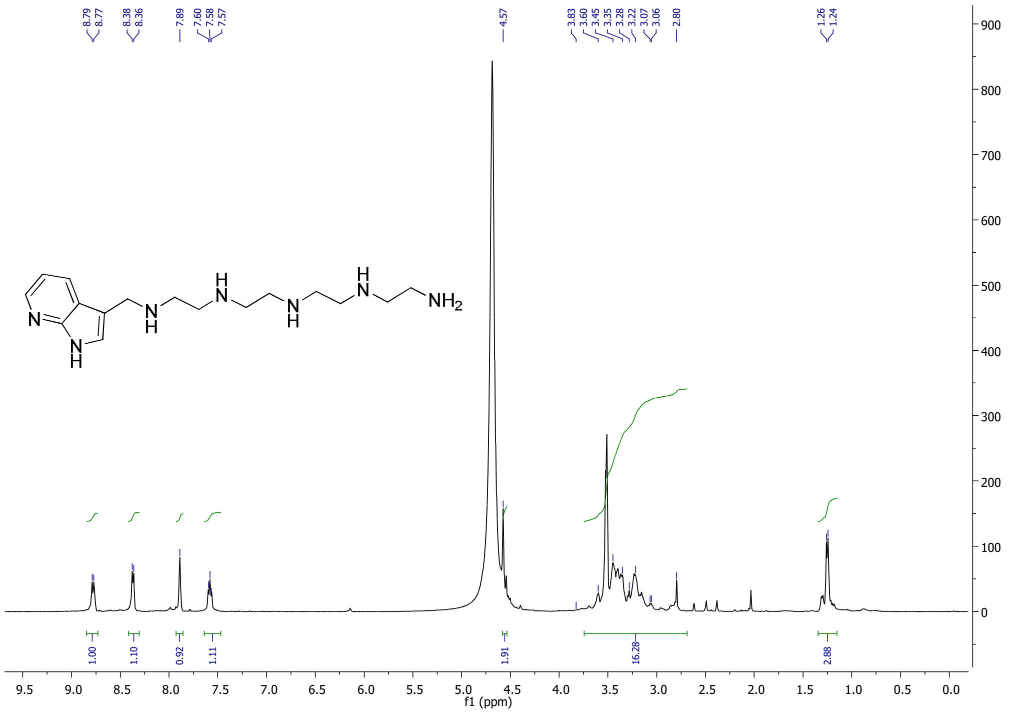

^{13}C NMR (151 MHz, D_2O)

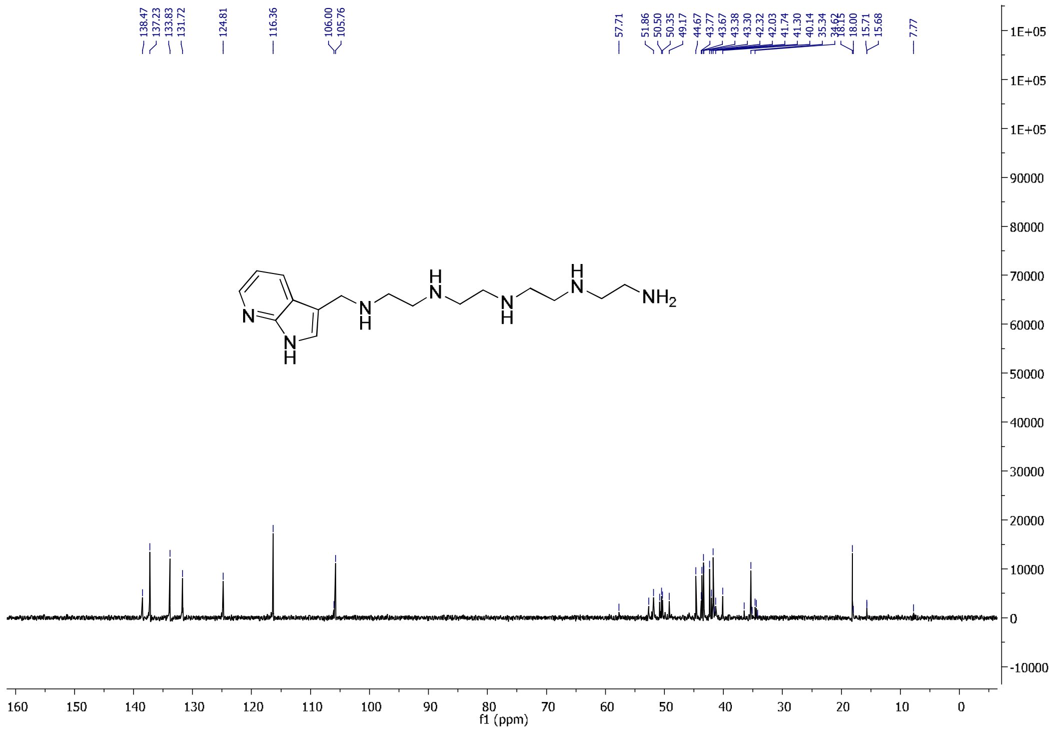

N-((1H-pyrrolo[2,3-b]pyridin-3-yl)methyl)-2-(pyrrolidin-1-yl)ethanamine (24e)

^{1}H NMR (600 MHz, DMSO-d$_6$)

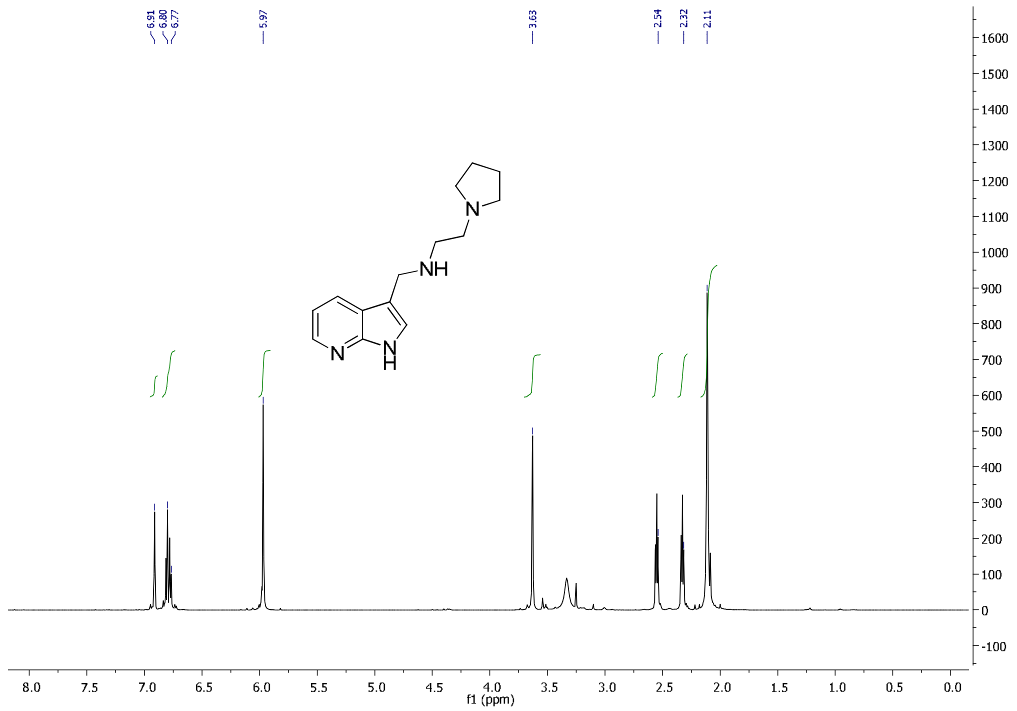

c^{13}C NMR (151 MHz, DMSO-d$_6$)

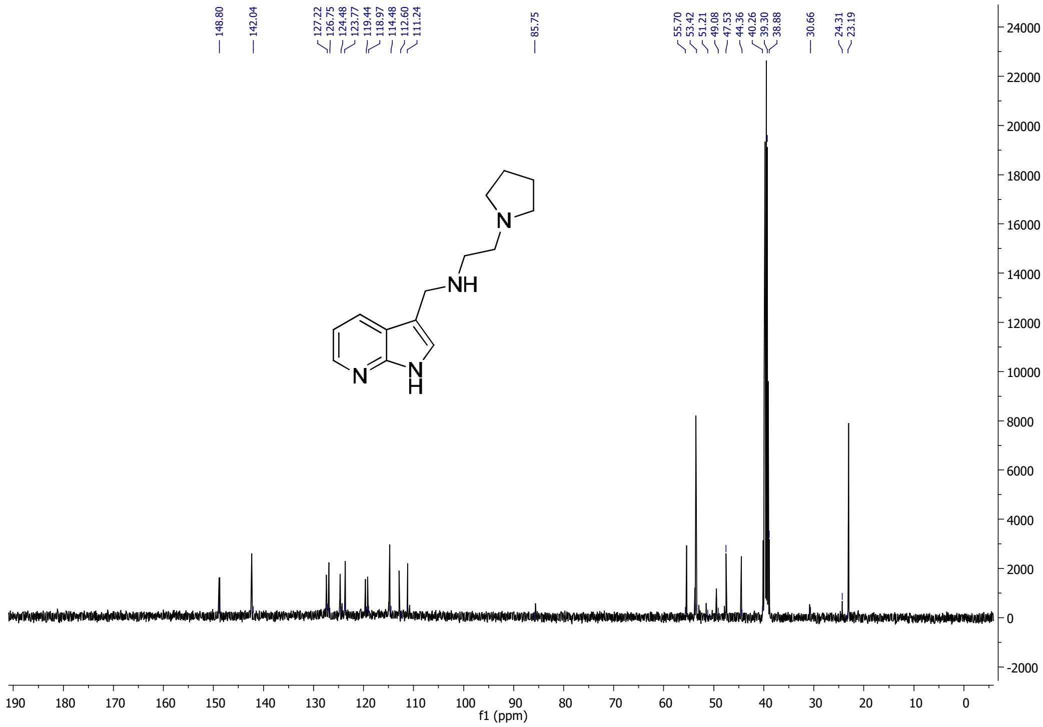

N1-(2-aminoethyl)-N2-(2-((2-((benzo[d][1,3]dioxol-5-ylmethyl)amino)ethyl)amino)ethyl)ethane-1,2-diamine (25b)

1H NMR (600 MHz, D_2O)

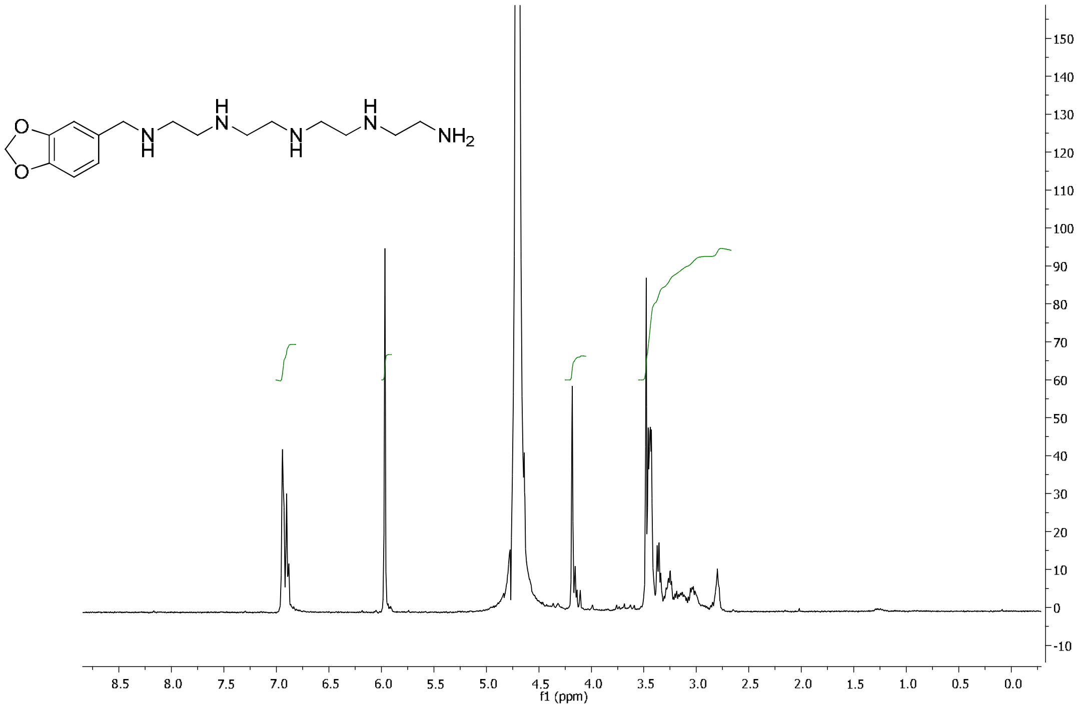

^{13}C NMR (151 MHz, D_2O)

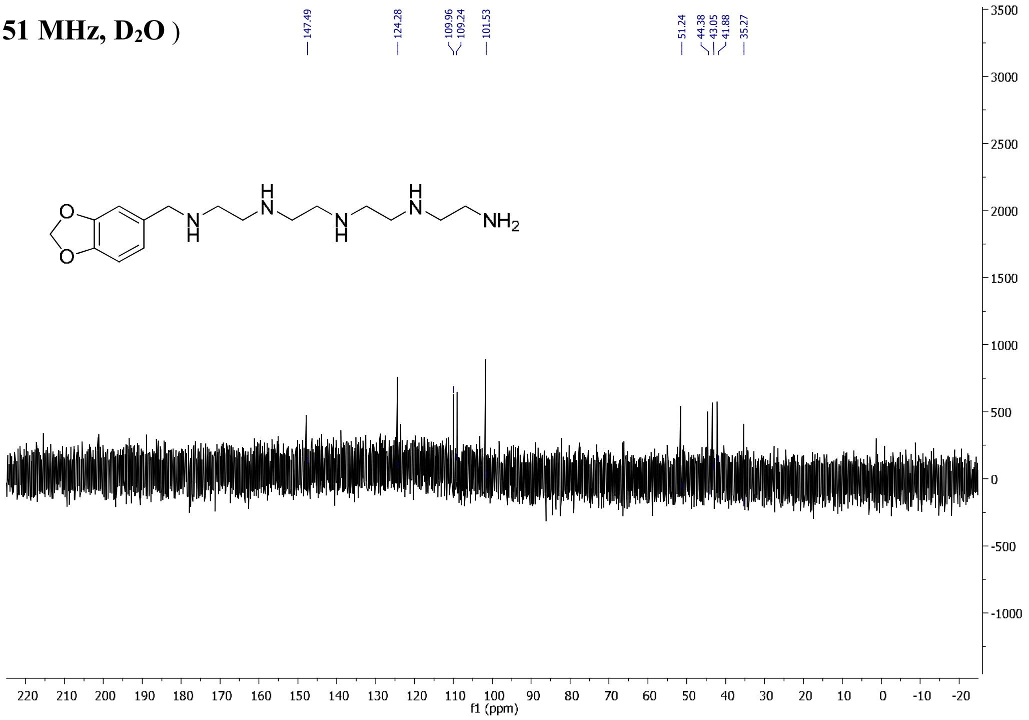

N1-(benzo[d][1,3]dioxol-5-ylmethyl)-N2,N2-dimethylethane-1,2-diamine (25d)

^{1}H NMR (600 MHz, DMSO-d_6)

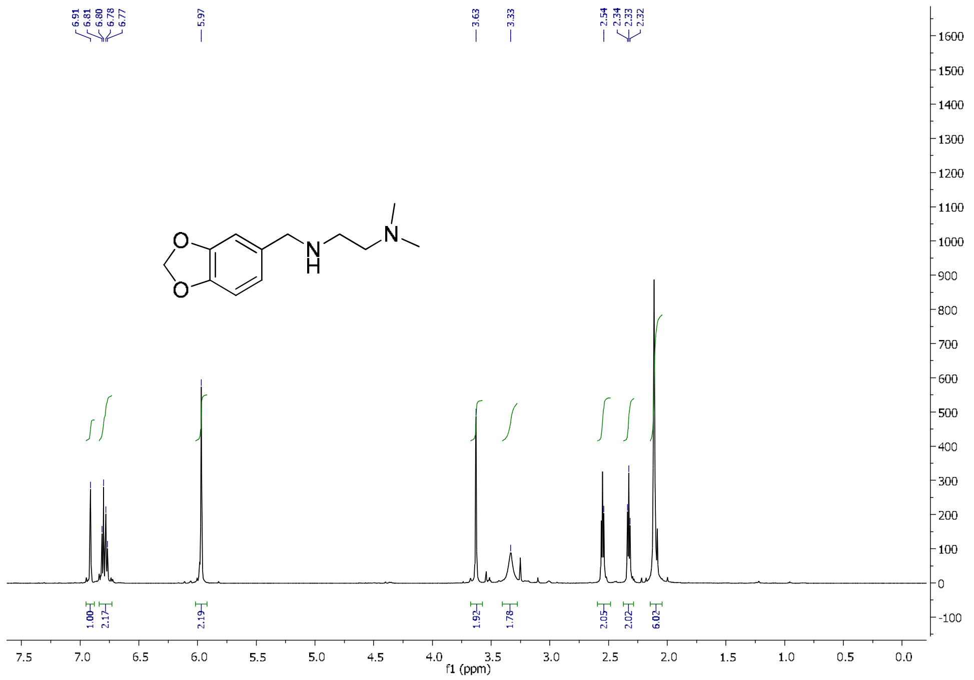

^{13}C NMR (151 MHz, DMSO-d_6)

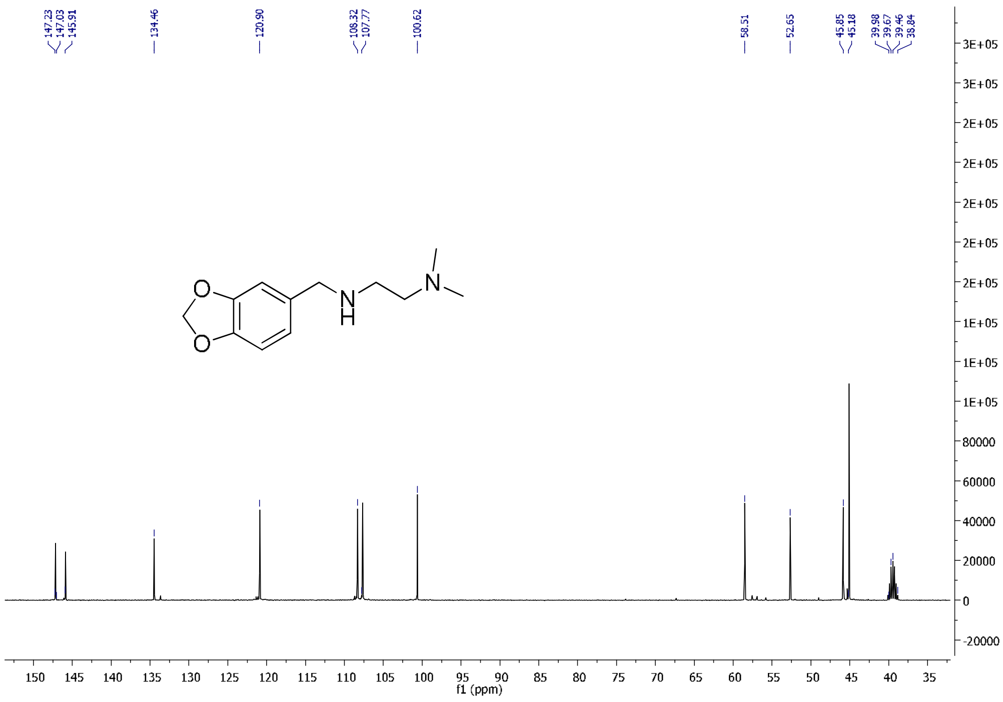

N-(benzo[d][1,3]dioxol-5-ylmethyl)-2-(pyrrolidin-1-yl)ethanamine (25e)

1H NMR (600 MHz, D_2O)

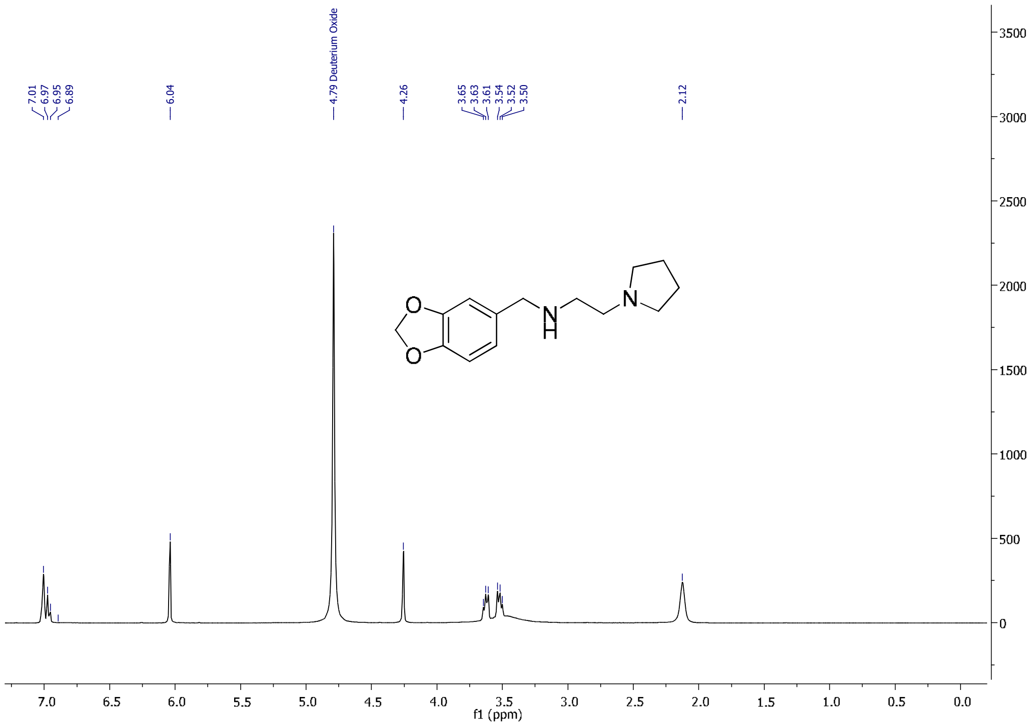

^{13}C NMR (151 MHz, D_2O)

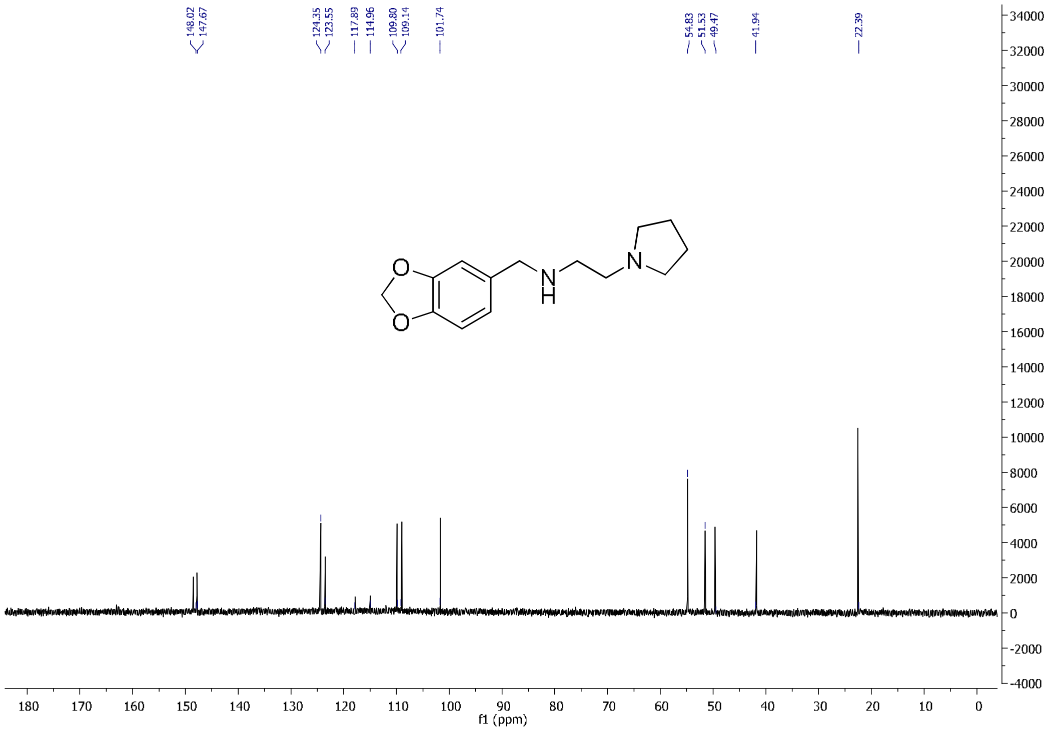

1-(3-(pyrrolidin-1-yl)propyl)-1H-indol-5-amine (5a)

^{1}H NMR (600 MHz, DMSO-d$_6$)

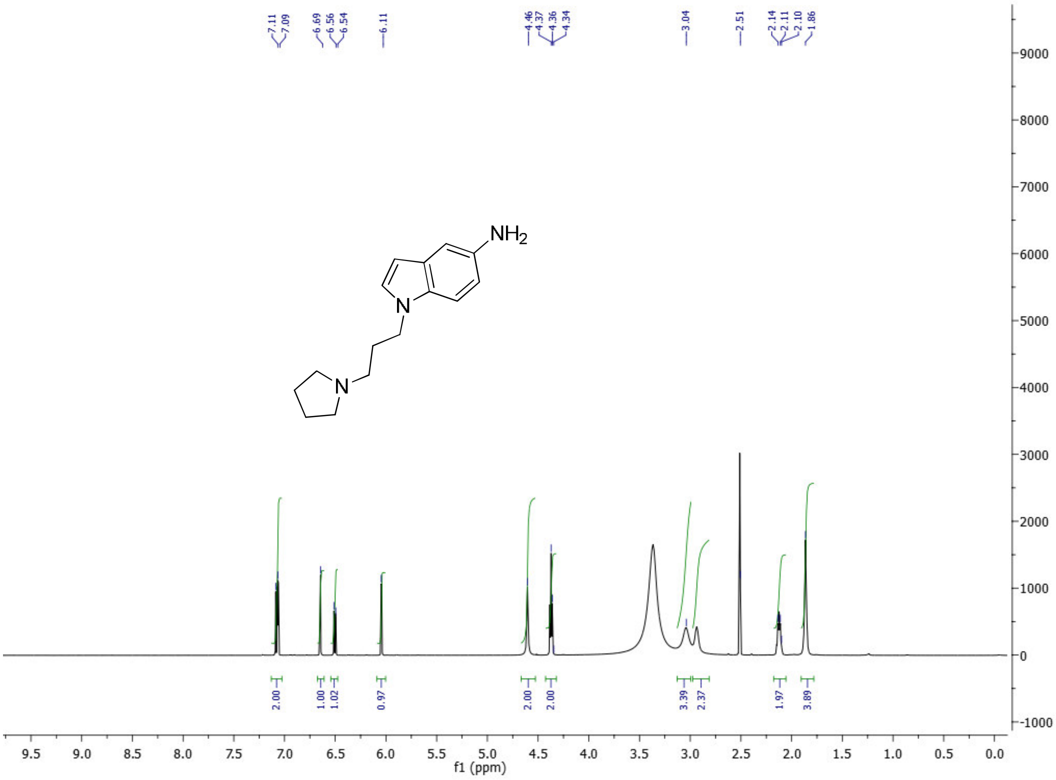

^{13}C NMR (101 MHz, DMSO-d$_6$)

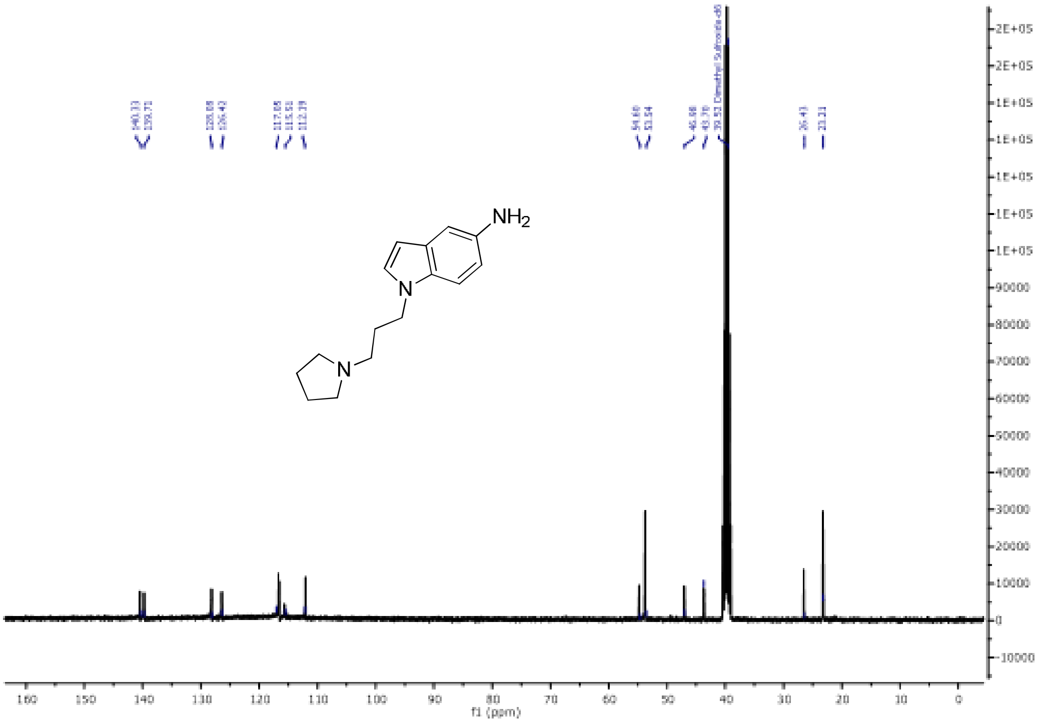

26B) ^{1}H-NMR (400 MHz, DMSO-d$_6$)

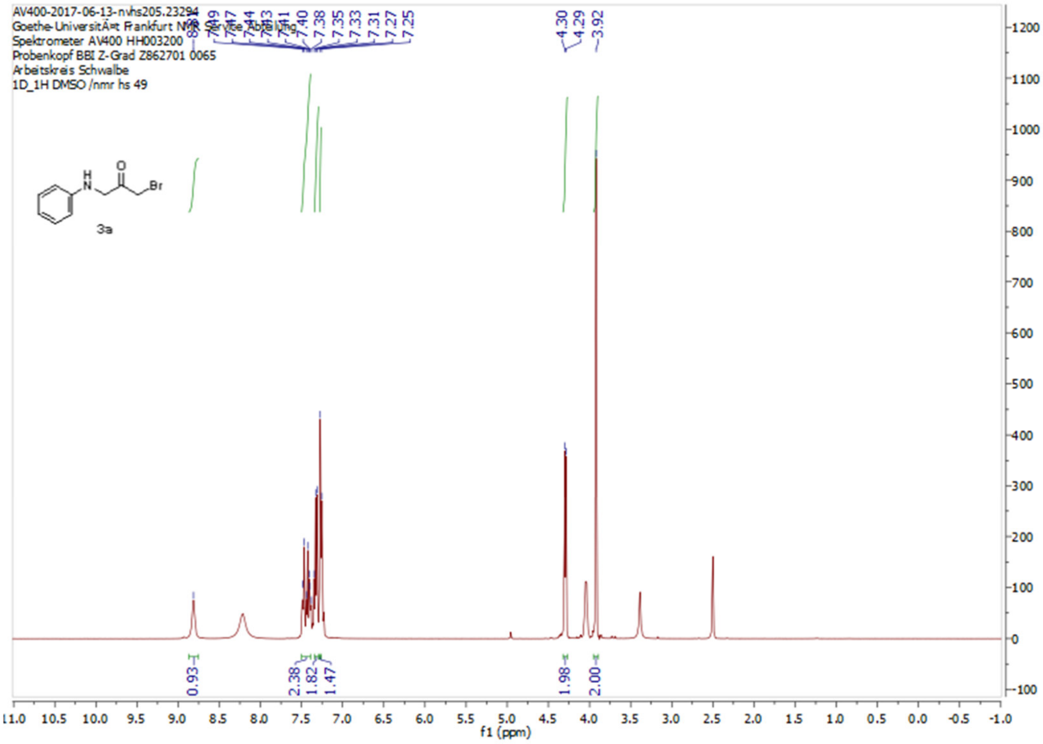

^{13}C NMR (101 MHz, DMSO-d$_6$)

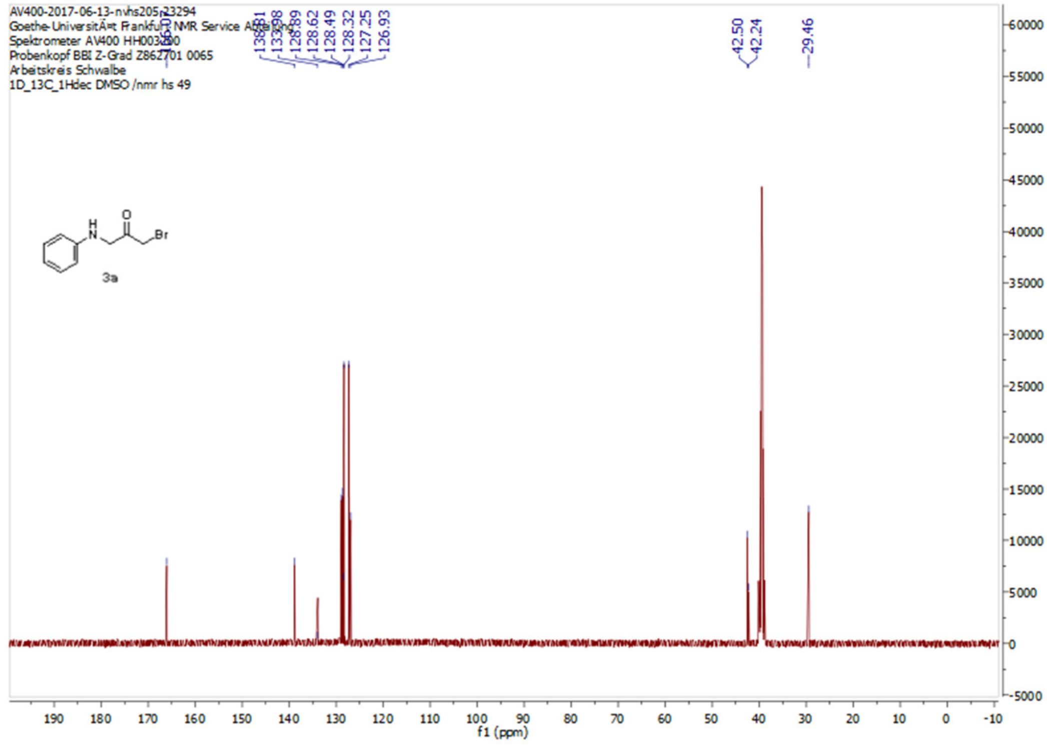

27B) ^{1}H-NMR (400 MHz, DMSO-d$_6$)

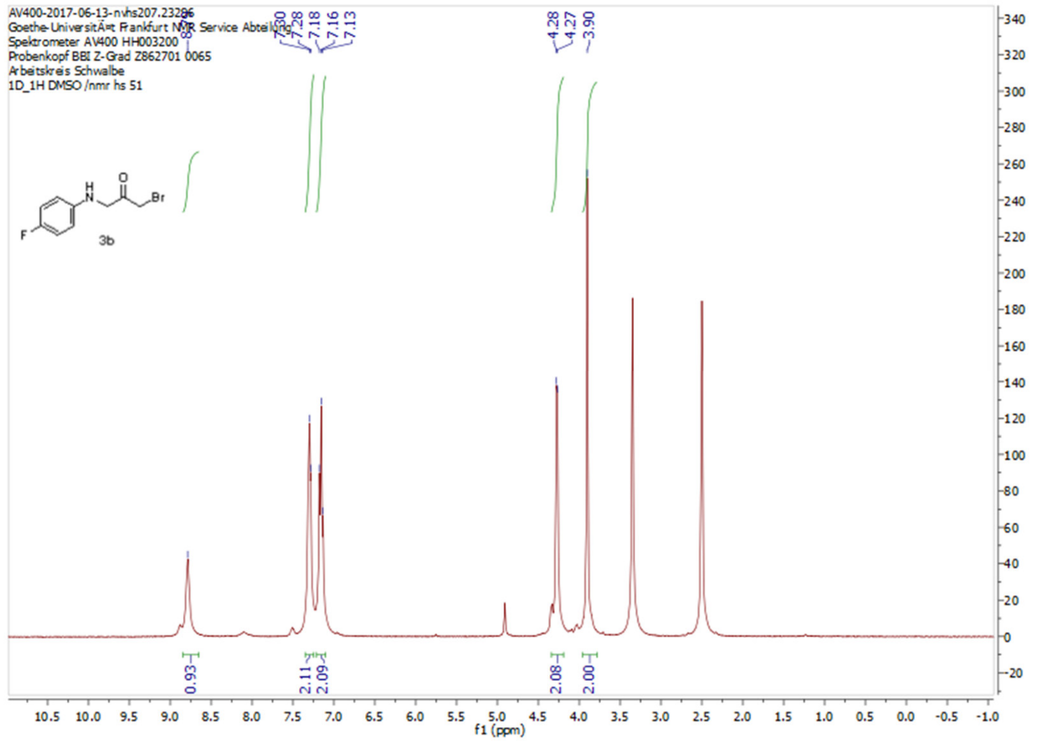

27B) ^{13}C NMR (101 MHz, DMSO-d$_6$)

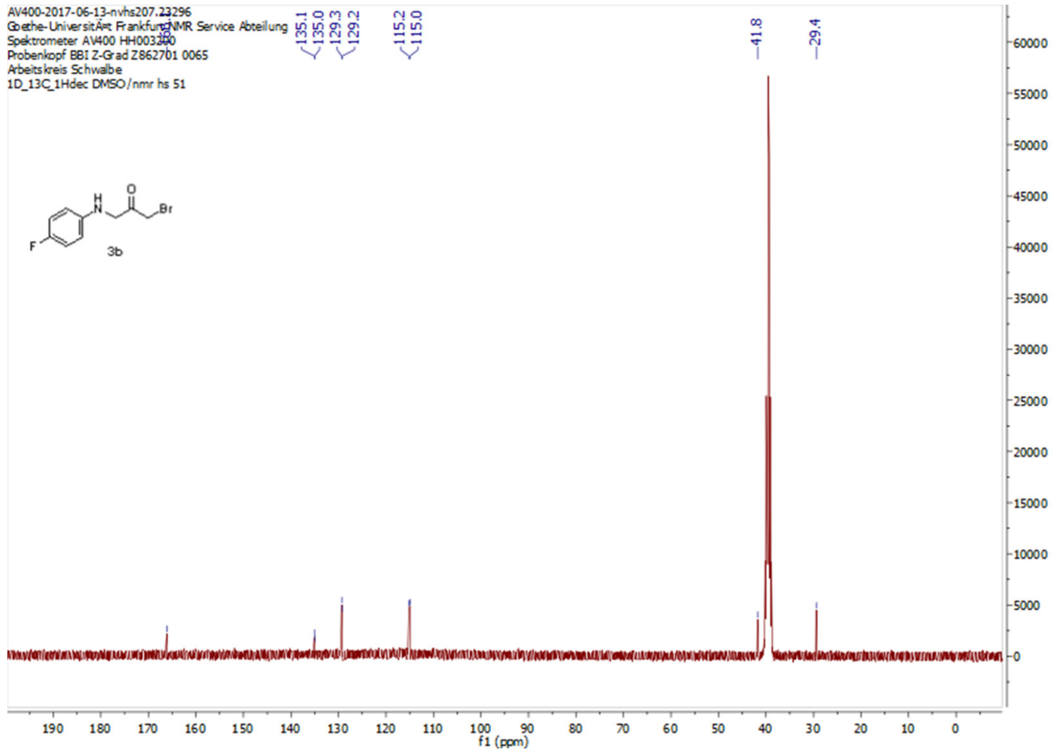

28B) ^{1}H-NMR (400 MHz, DMSO-d$_6$)

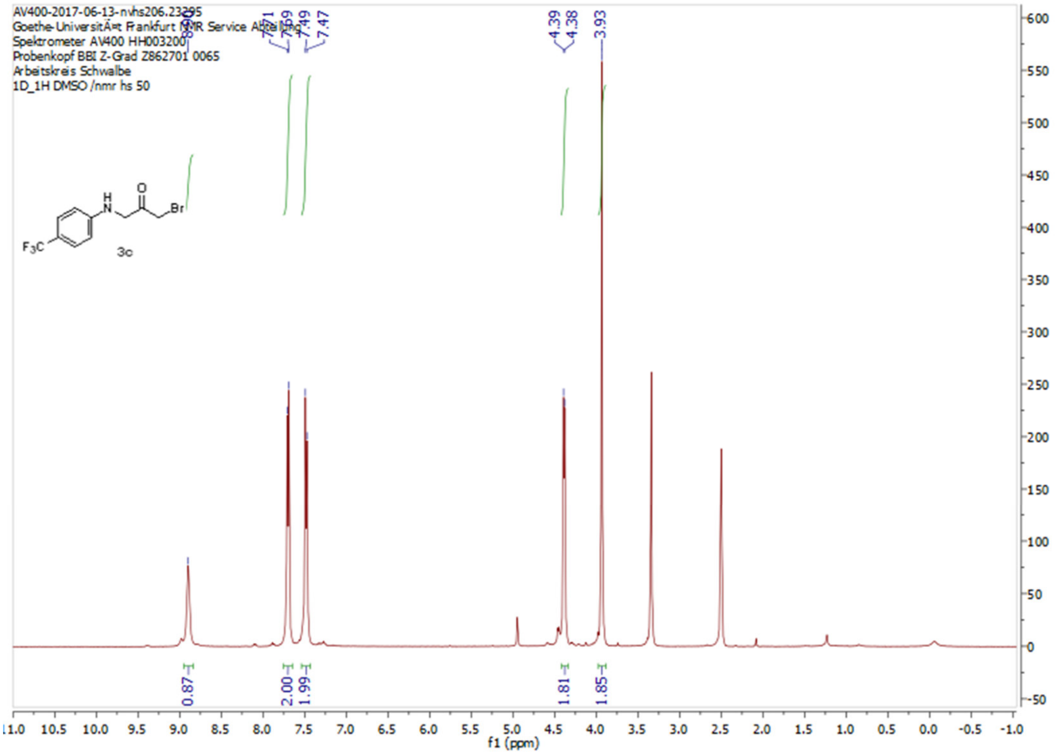

^{13}C NMR (101 MHz, DMSO-d$_6$)

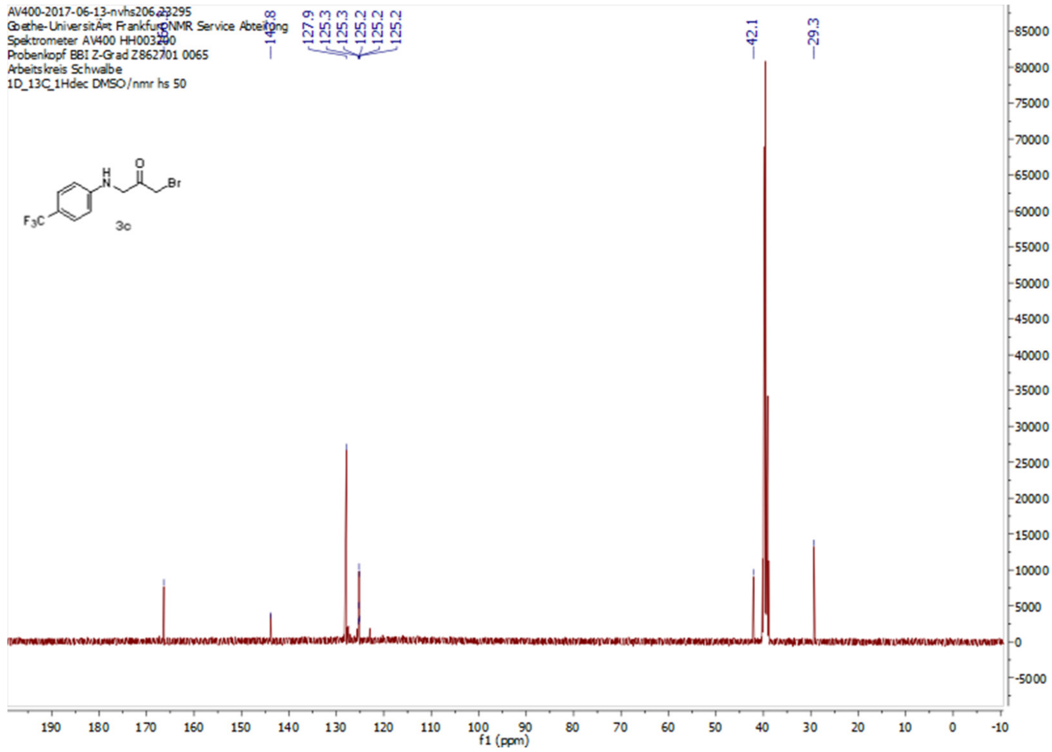

29B) ^{1}H-NMR (600 MHz, DMSO-d_6)

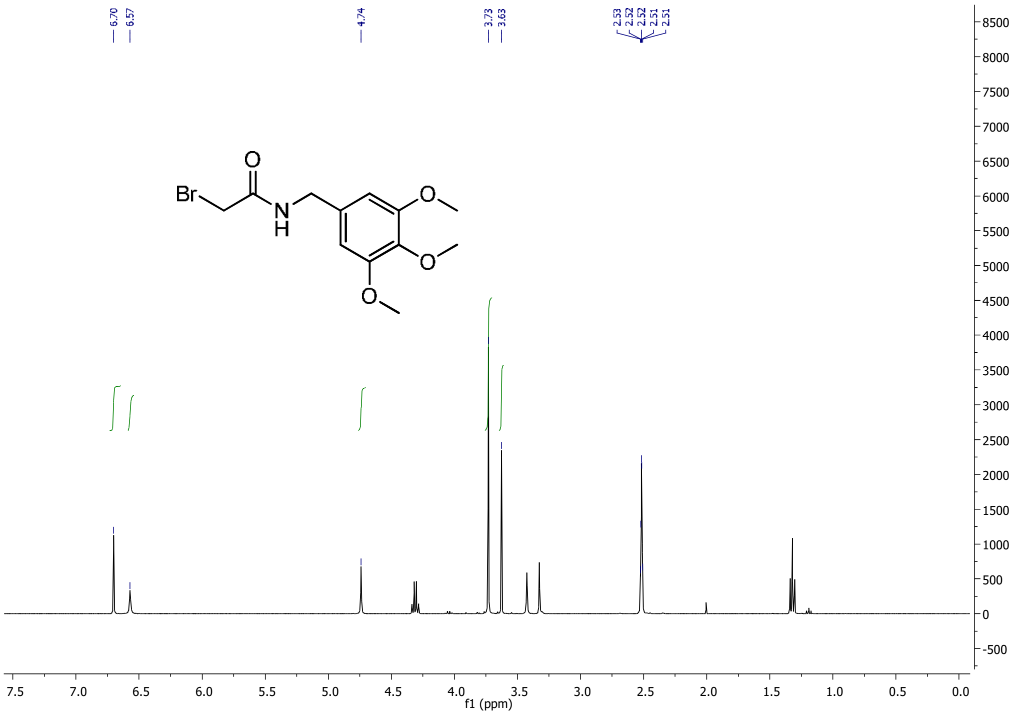

^{13}C NMR (151 MHz, DMSO-d_6)

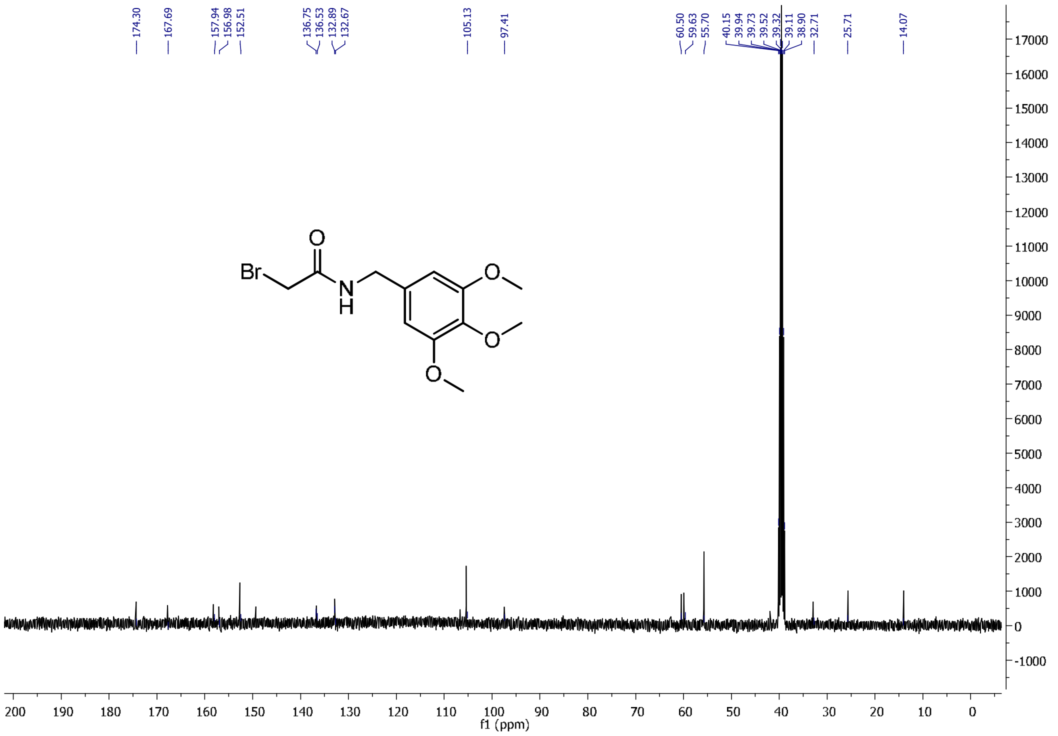

30B) ^{1}H-NMR (600 MHz, DMSO-d_6)

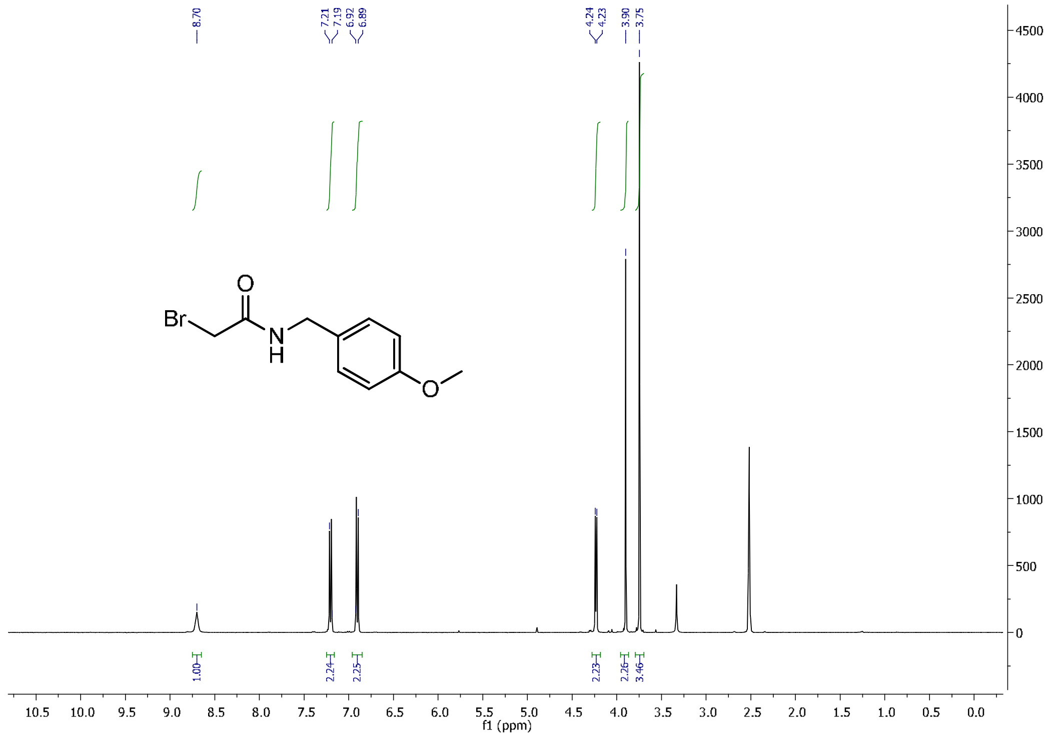

^{13}C NMR (151 MHz, DMSO-d_6)

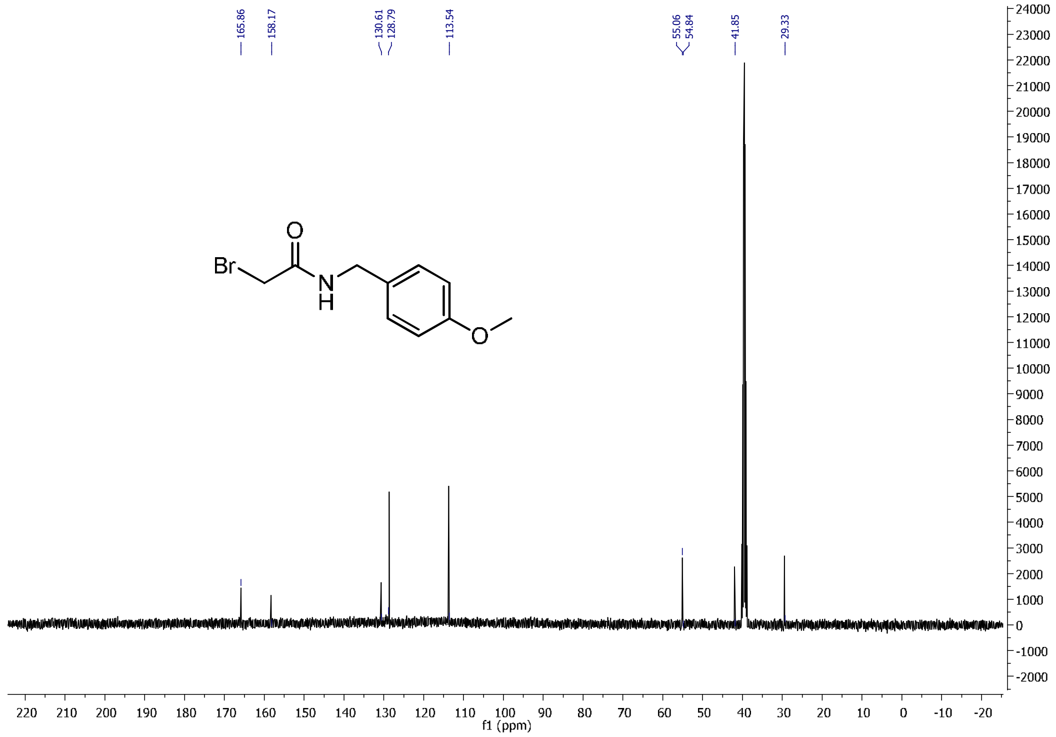

26C) ^{1}H-NMR (600 MHz, DMSO-d$_6$)

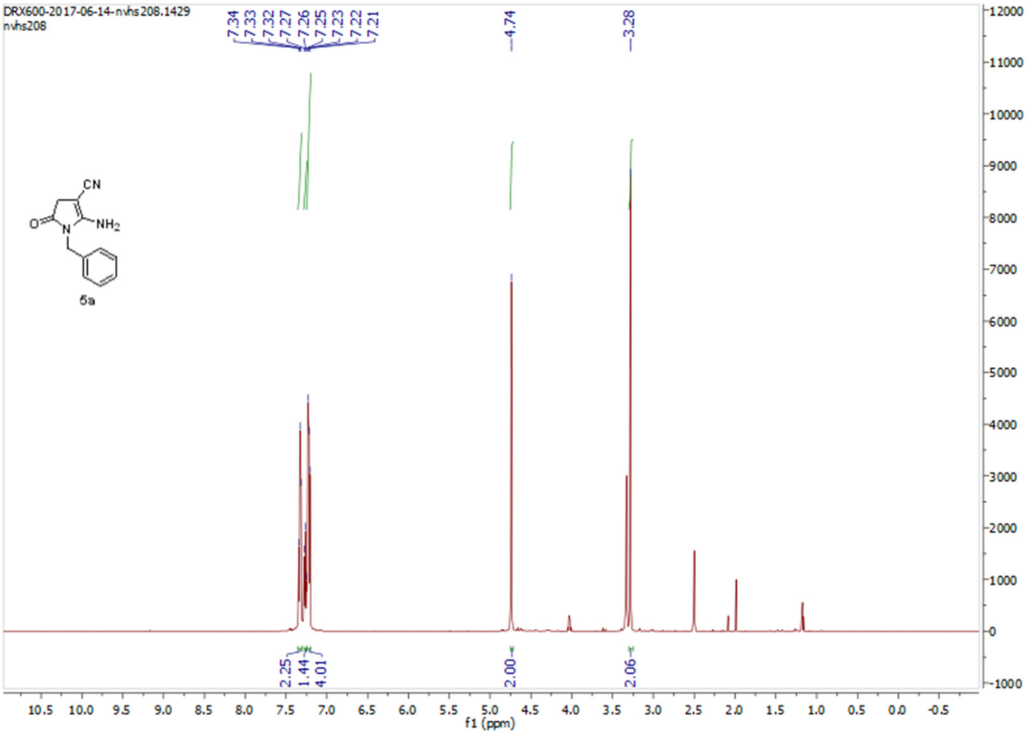

^{13}**C NMR (151 MHz, DMSO-d$_6$)**

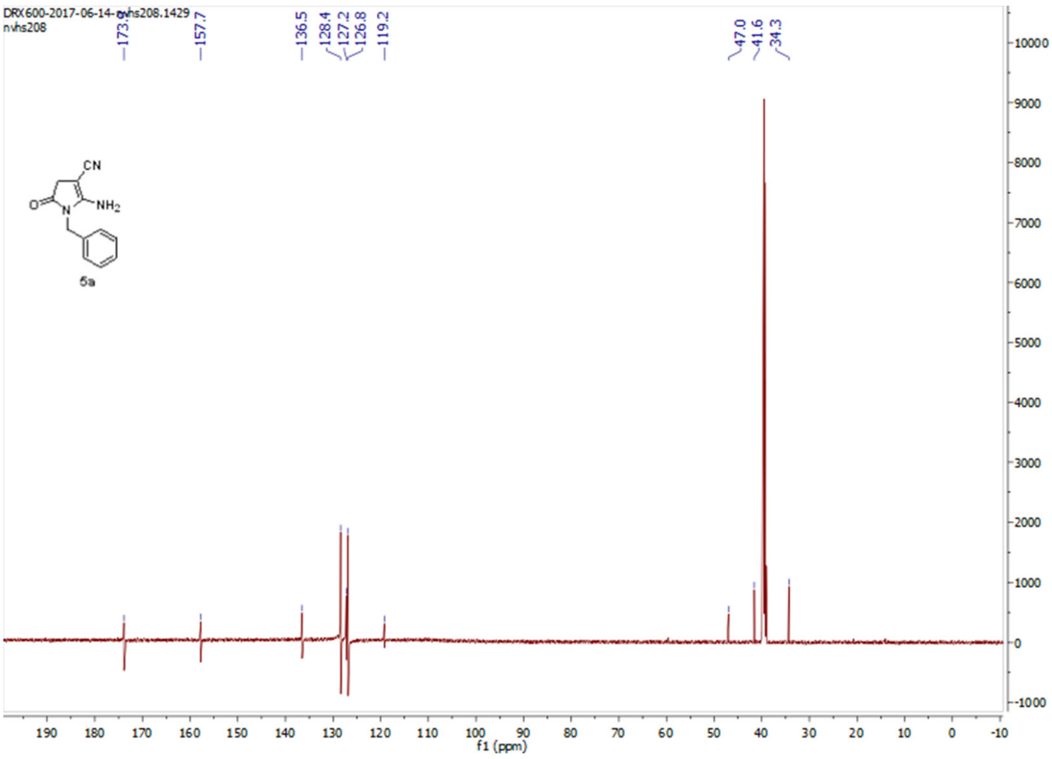

27C) ^{1}H-NMR (600 MHz, DMSO-d$_6$)

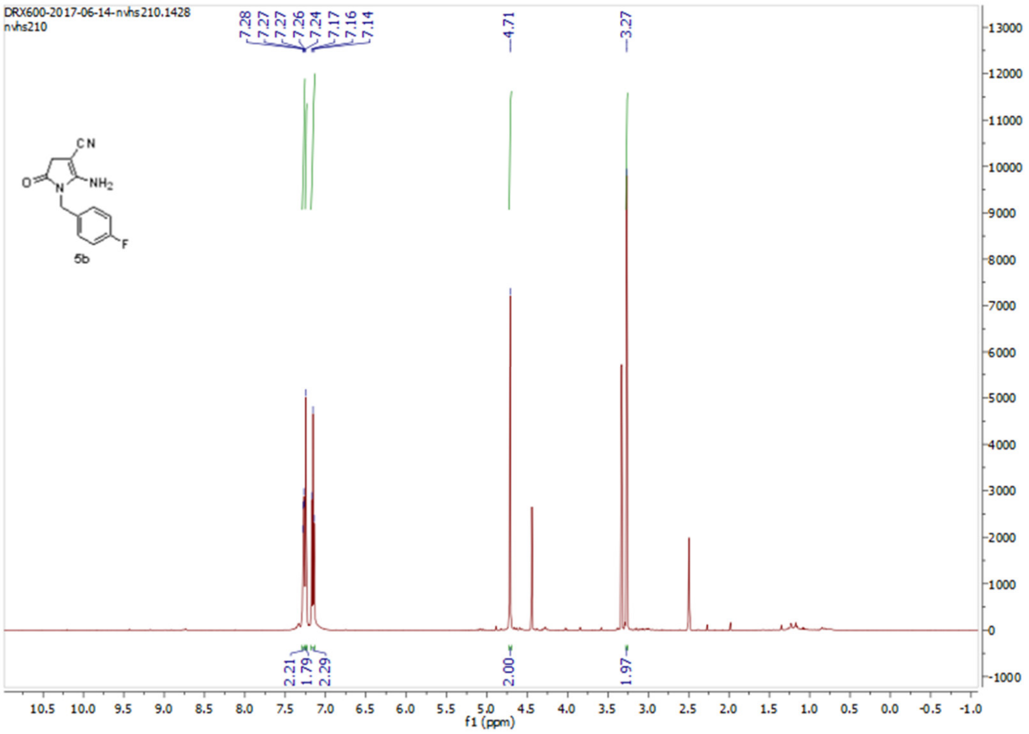

^{13}C NMR (151 MHz, DMSO-d$_6$)

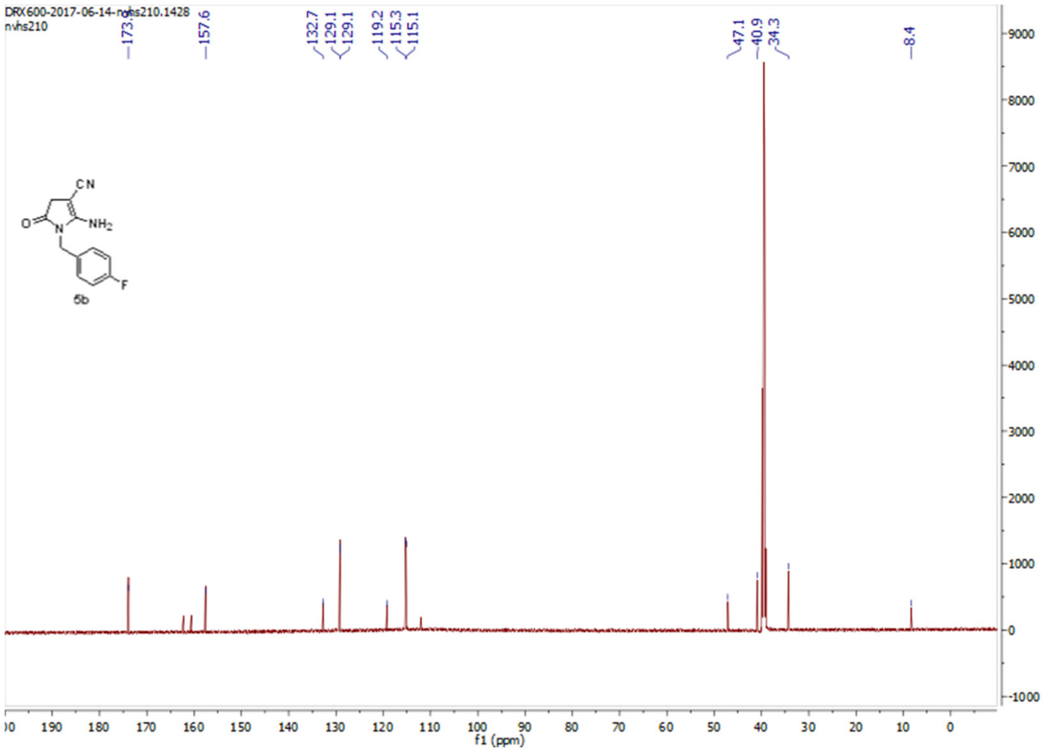

28C) ^{1}H-NMR (600 MHz, DMSO-d_6)

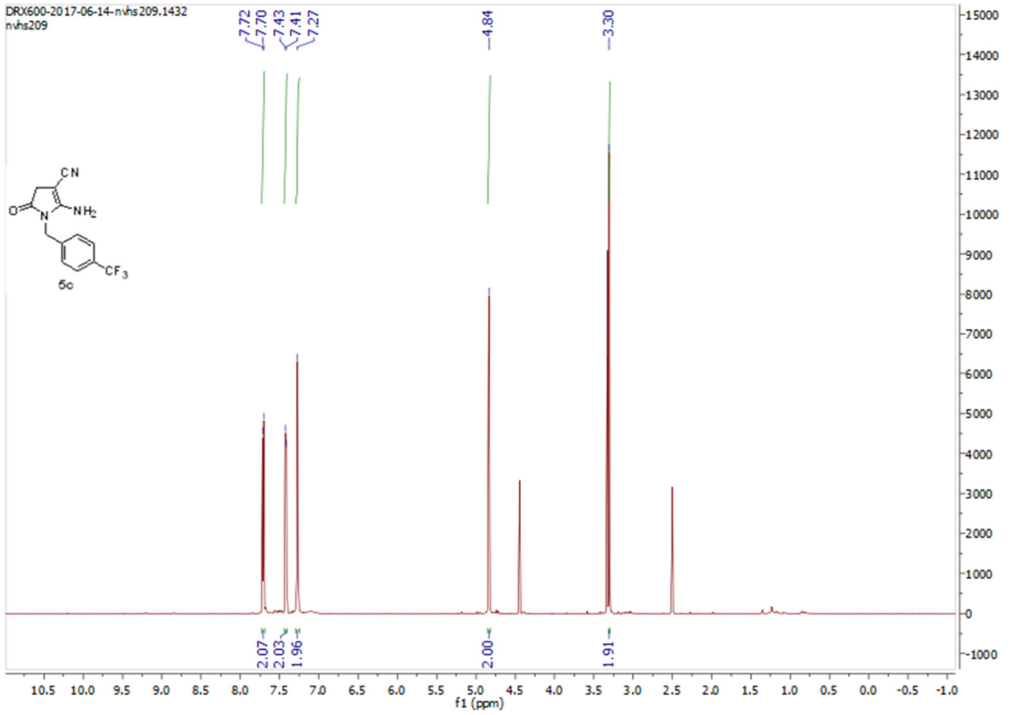

^{13}C NMR (151 MHz, DMSO-d_6)

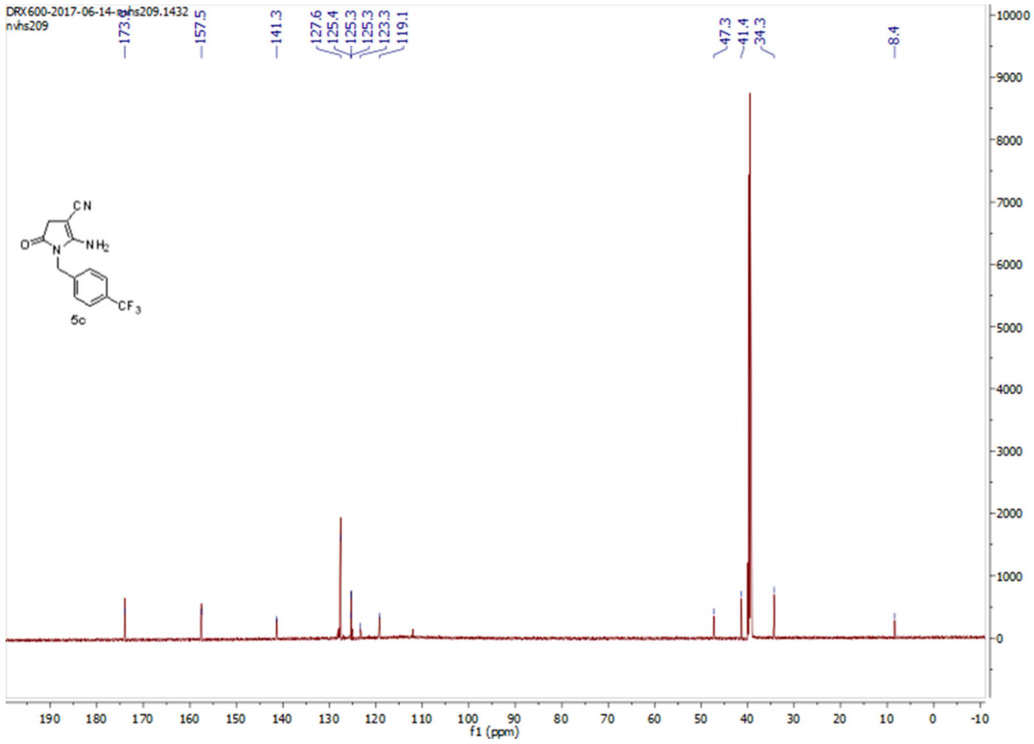

29C) ^{1}H-NMR (600 MHz, DMSO-d_6)

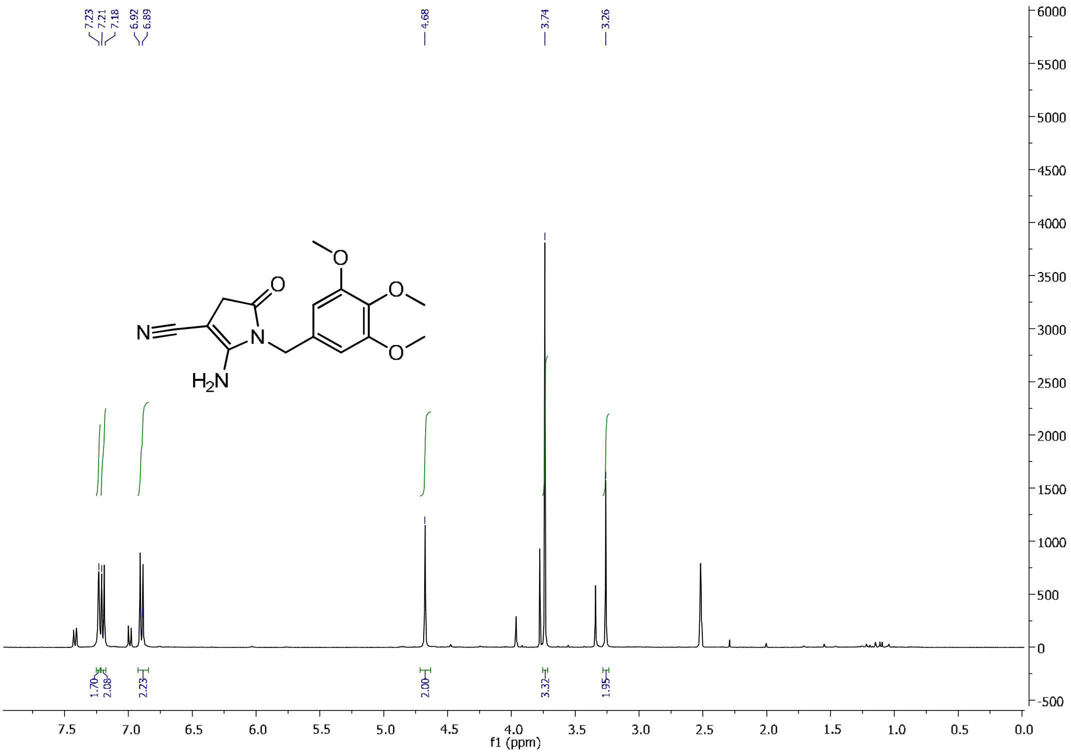

^{13}C NMR (151 MHz, DMSO-d_6)

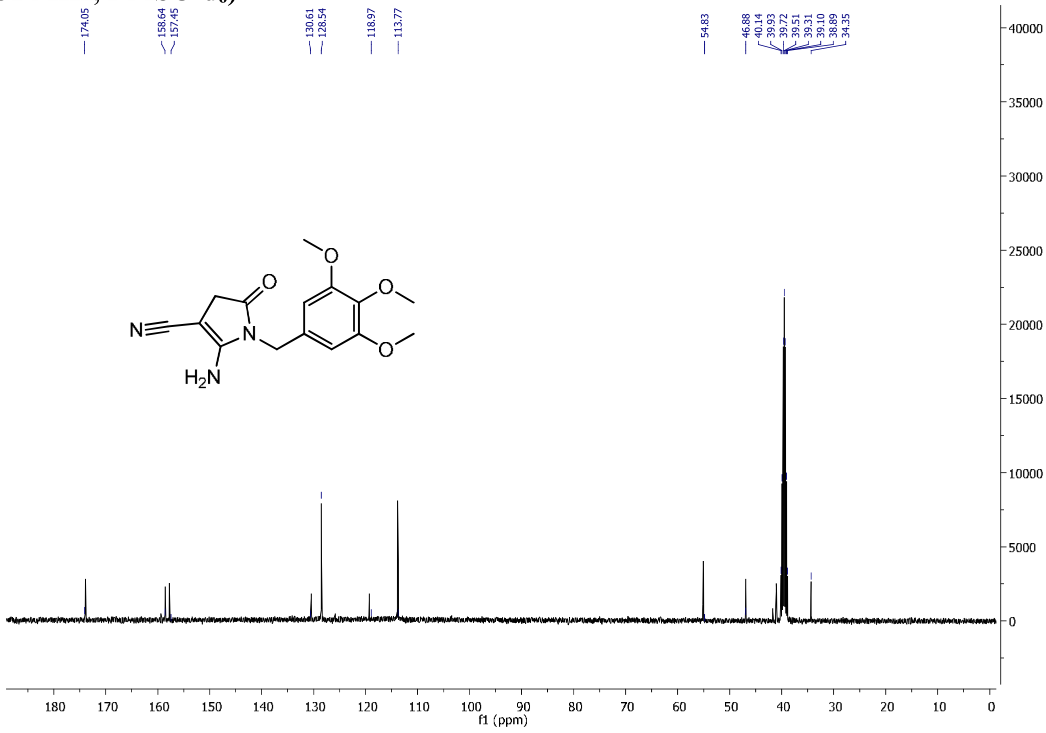

30C) ^{1}H-NMR (600 MHz, DMSO-d$_6$)

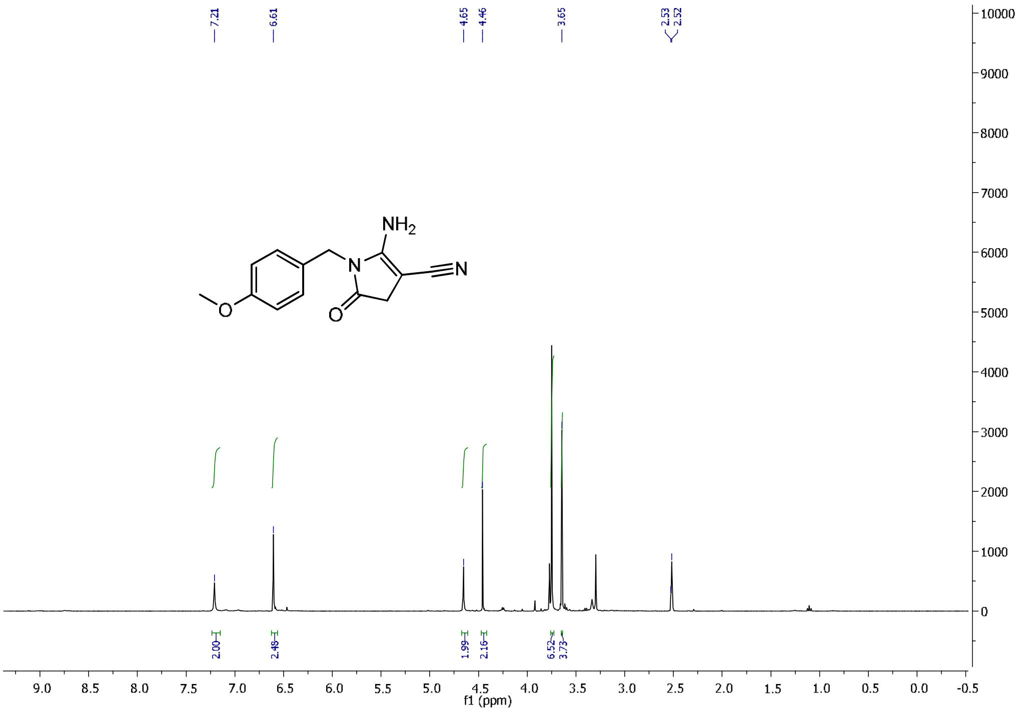

^{13}C NMR (151 MHz, DMSO-d$_6$)

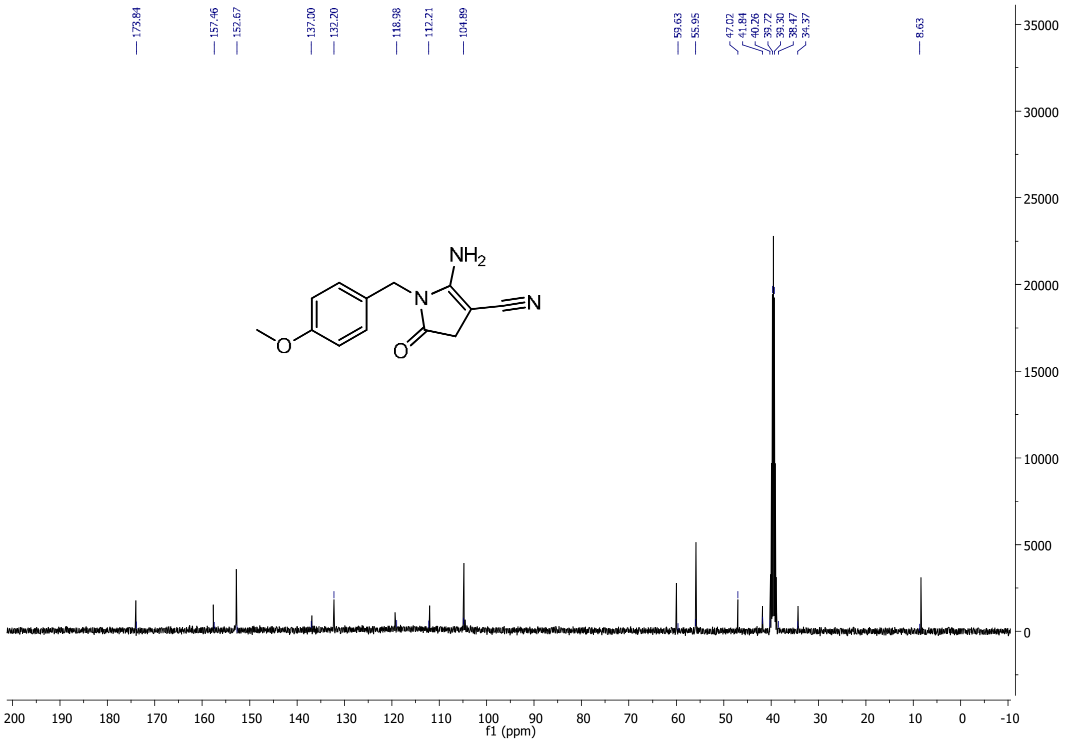

26d) ^{1}H-NMR (400 MHz, DMSO-d$_6$)

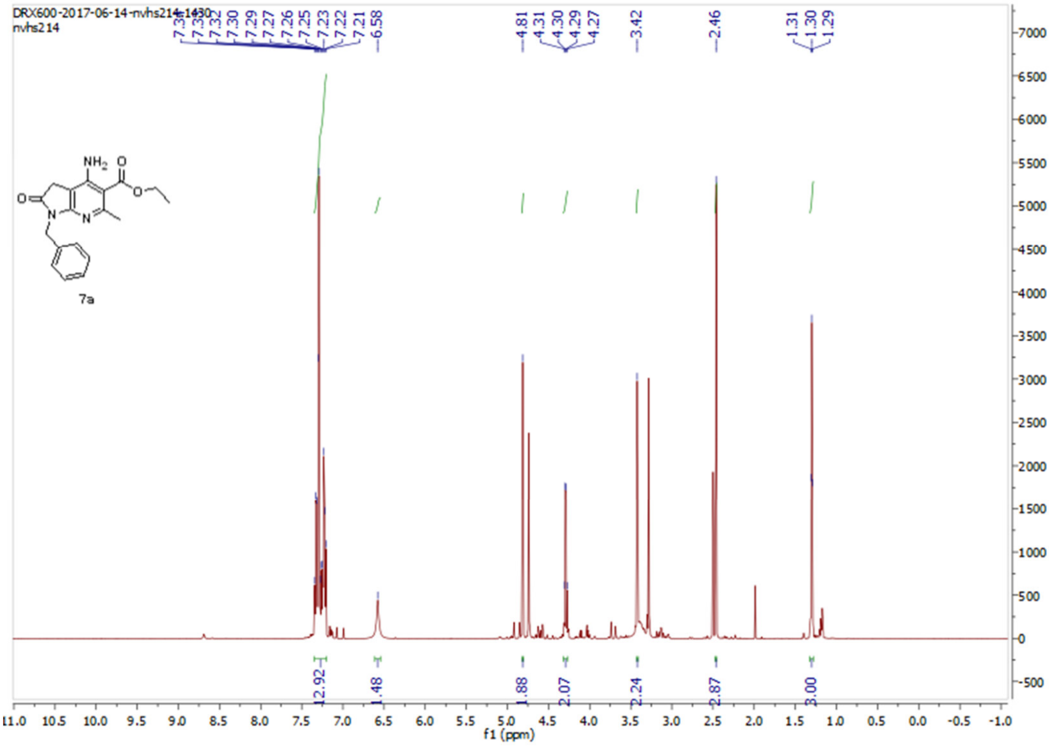

^{13}C NMR (101 MHz, DMSO-d$_6$)

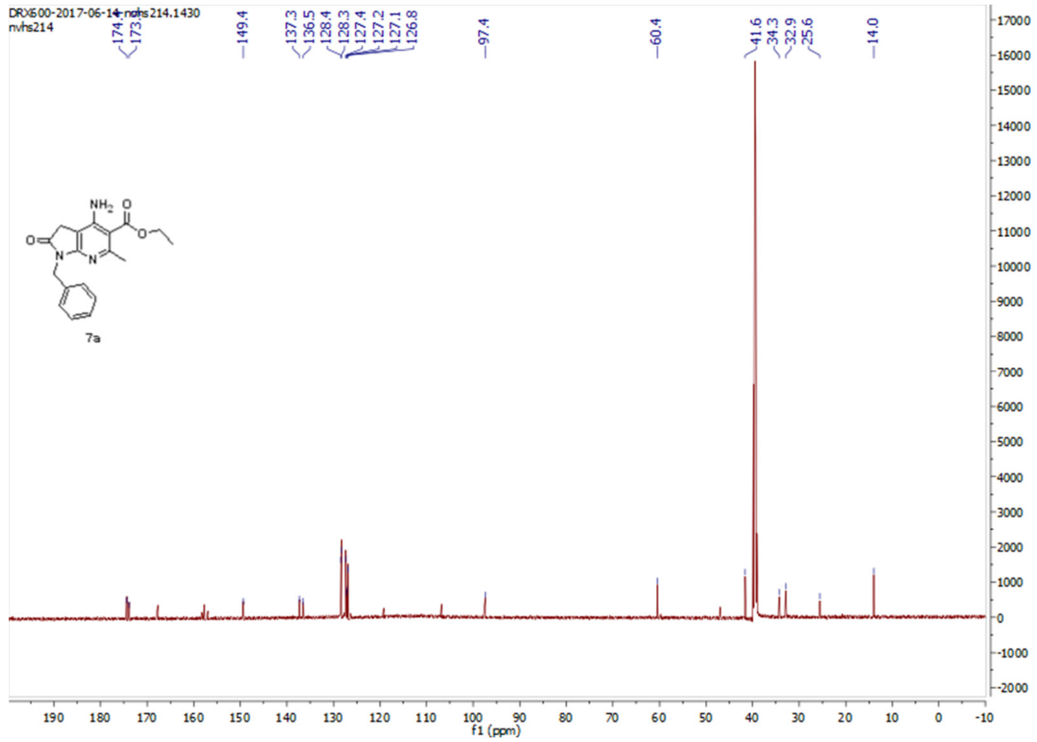

27d) ^{1}H-NMR (400 MHz, DMSO-d$_6$)

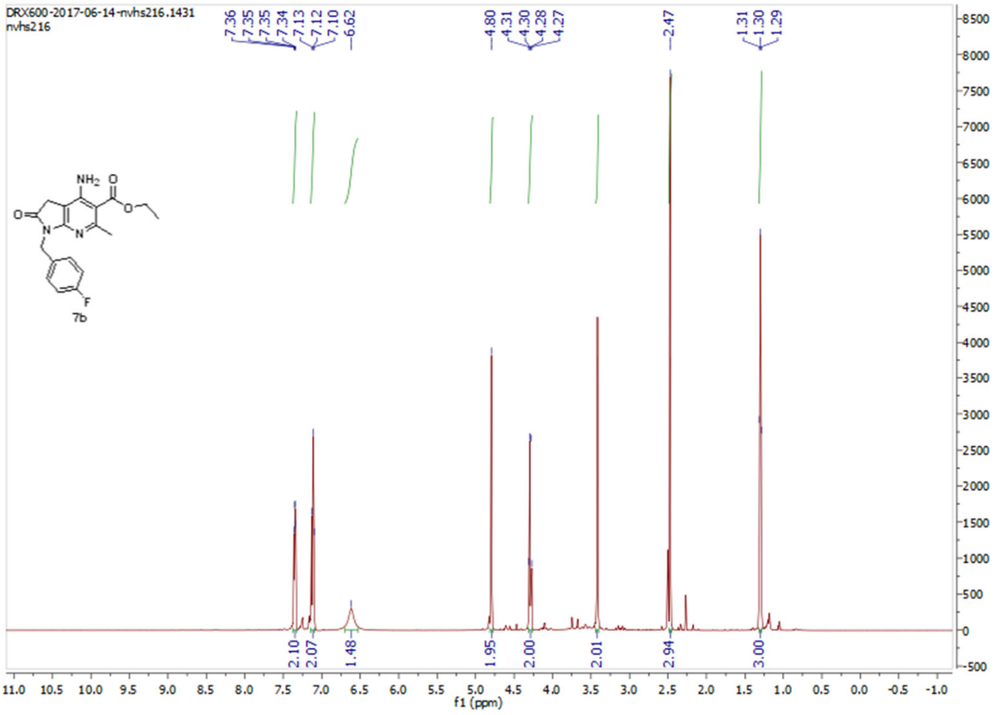

^{13}C NMR (101 MHz, DMSO-d$_6$)

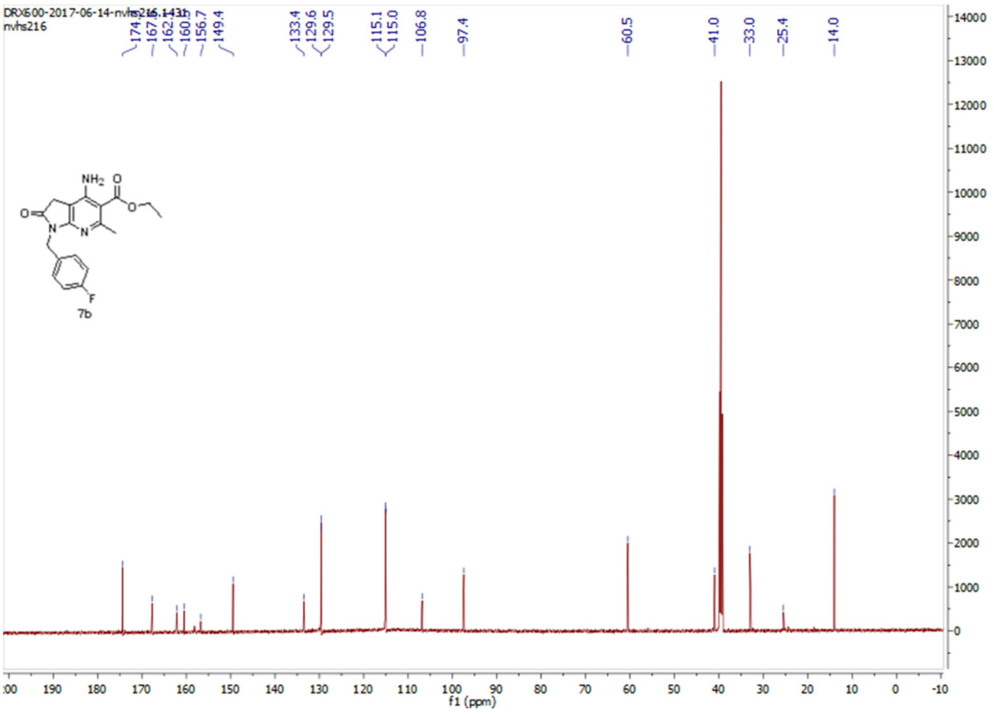

28d) ^{1}H-NMR (400 MHz, DMSO-d$_6$)

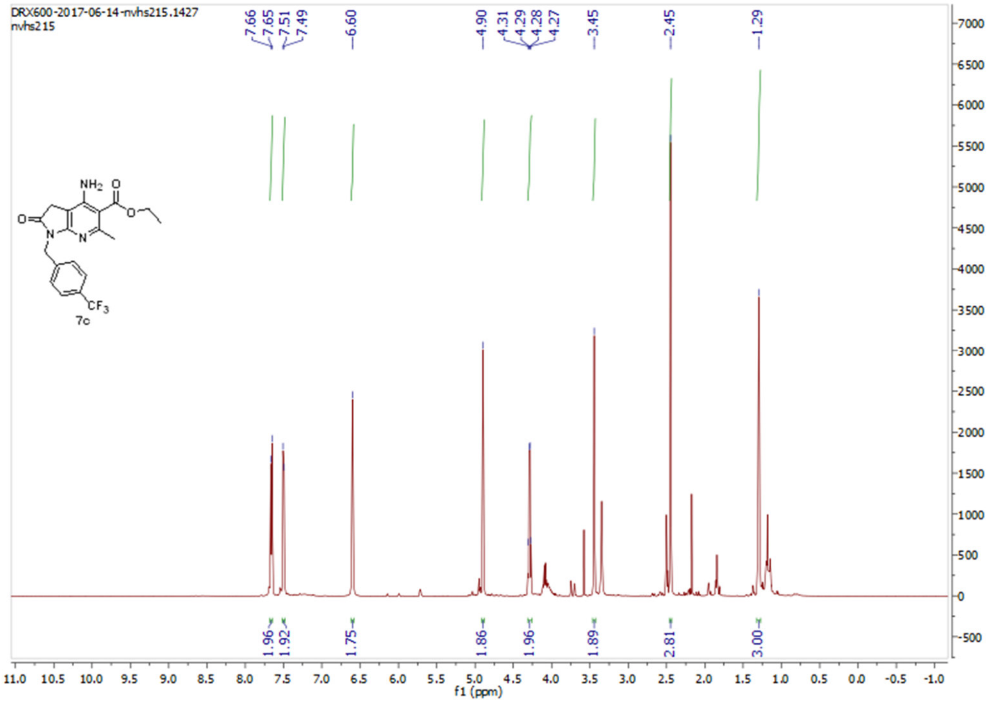

^{13}C NMR (101 MHz, DMSO-d$_6$)

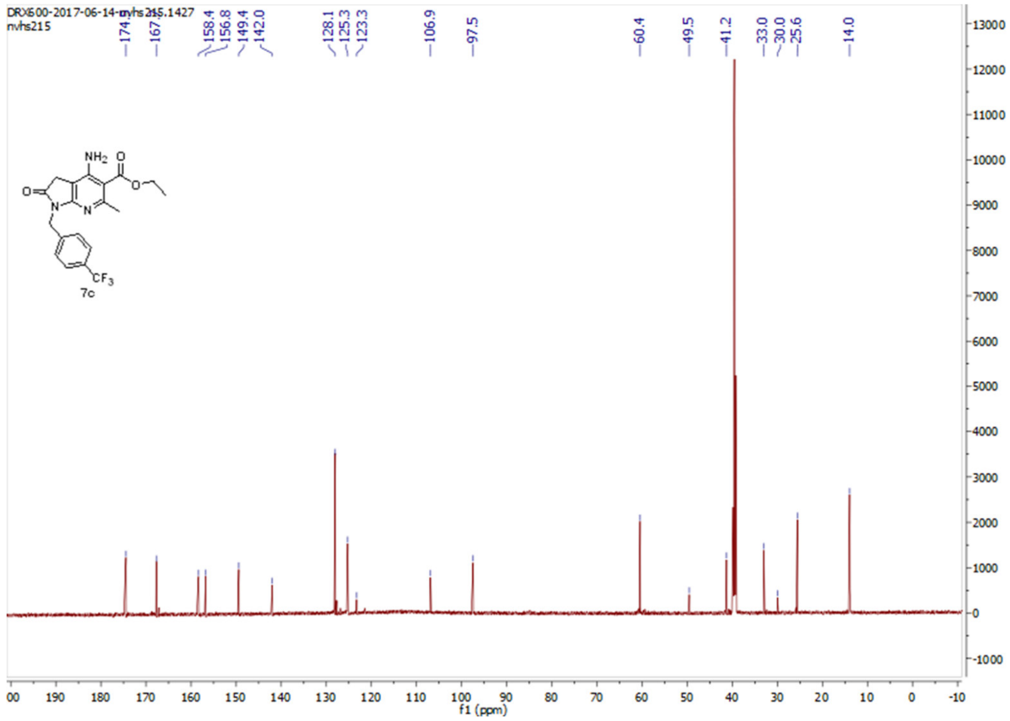

29d) ^{1}H-NMR (400 MHz, DMSO-d$_6$)

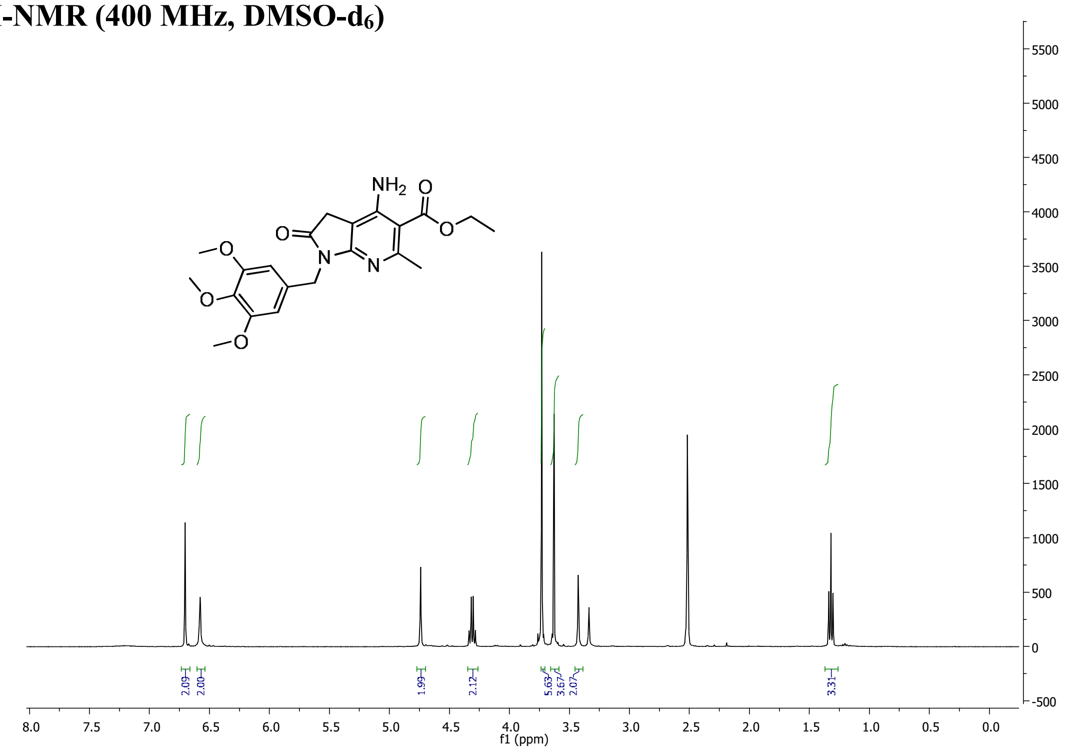

^{13}C NMR (101 MHz, DMSO-d$_6$)

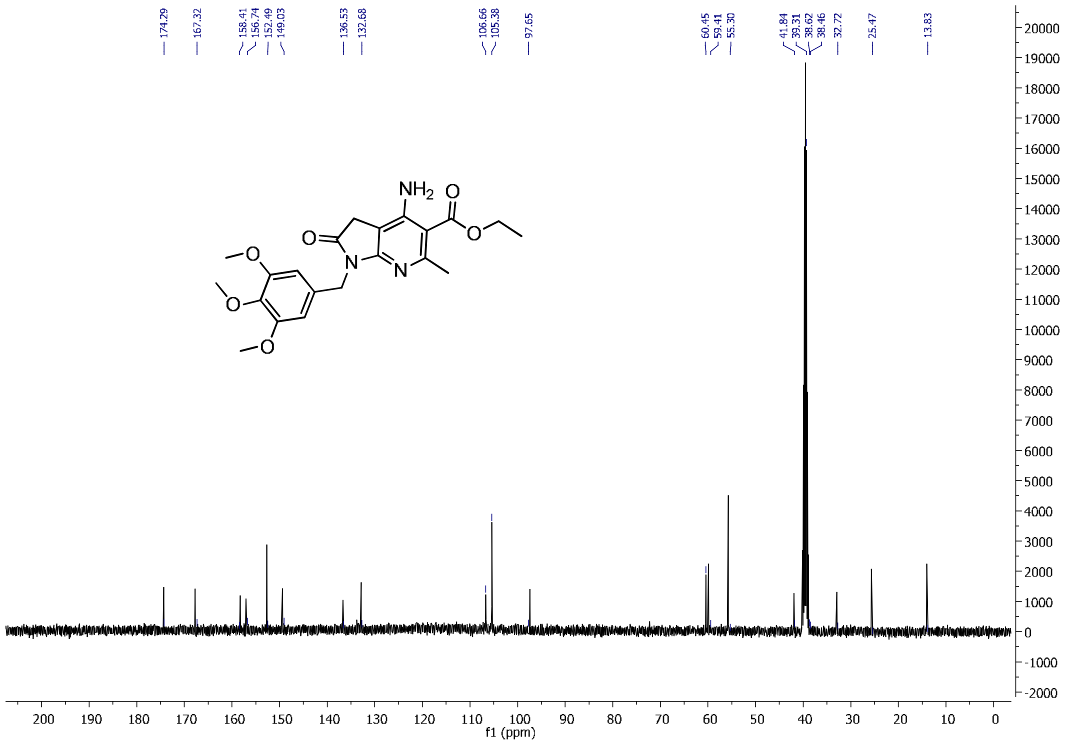

30d) ^{1}H-NMR (400 MHz, DMSO-d$_6$)

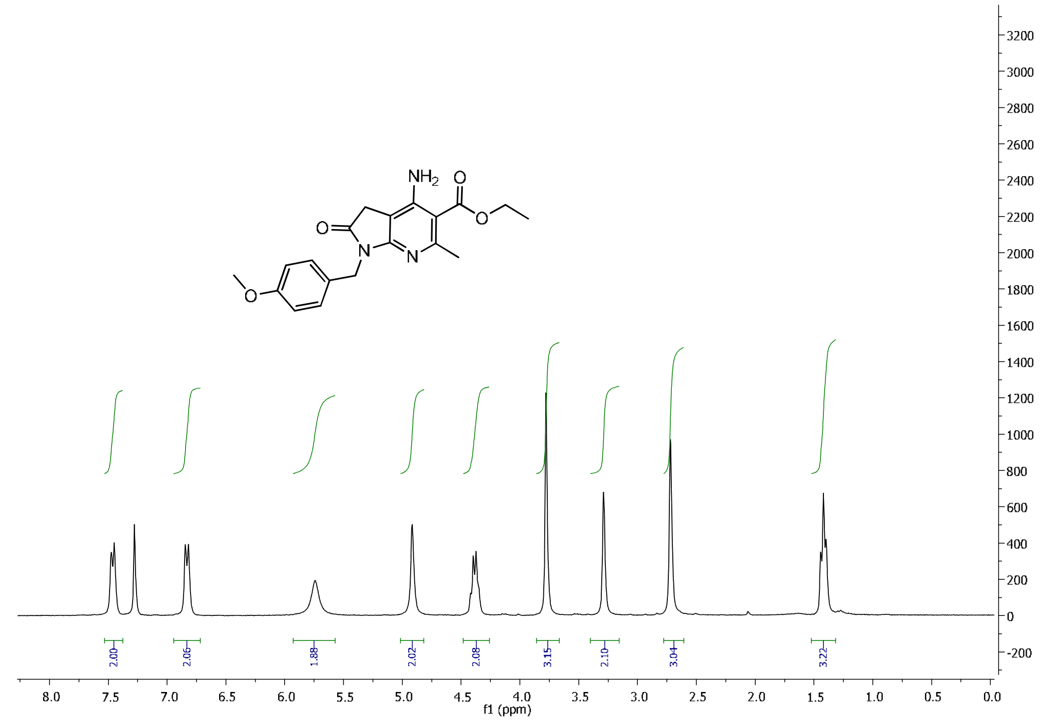

^{13}C NMR (101 MHz, DMSO-d$_6$)

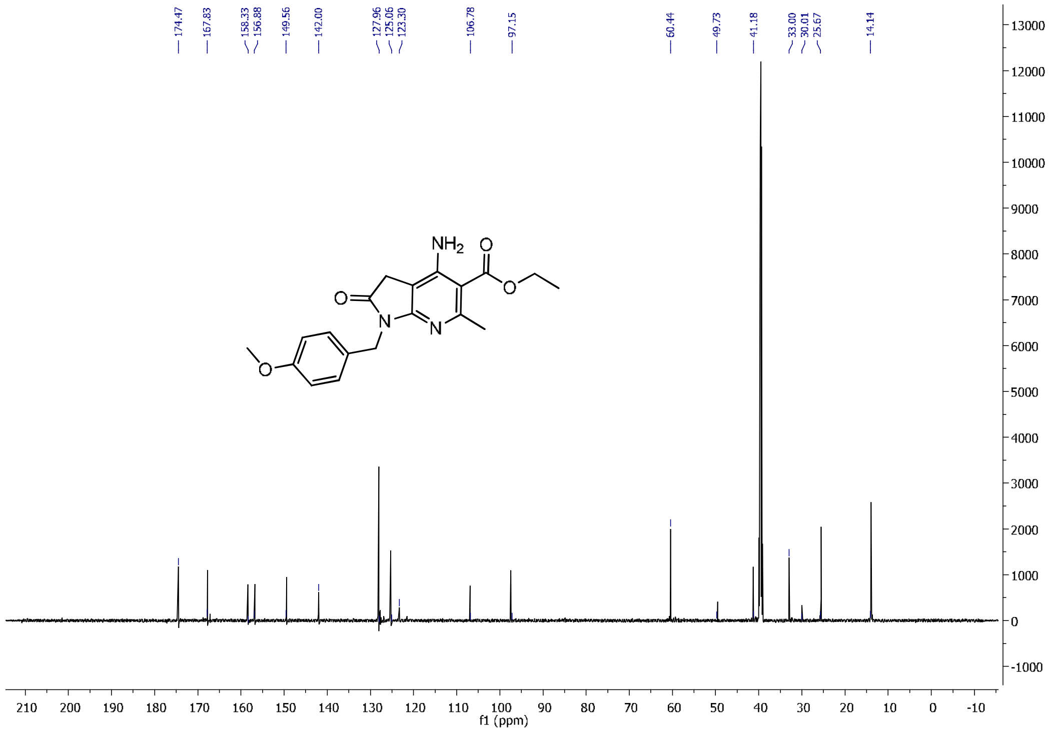

31) 1H-indol-5-amine ^{1}H-NMR (400 MHz, DMSO-d$_6$)

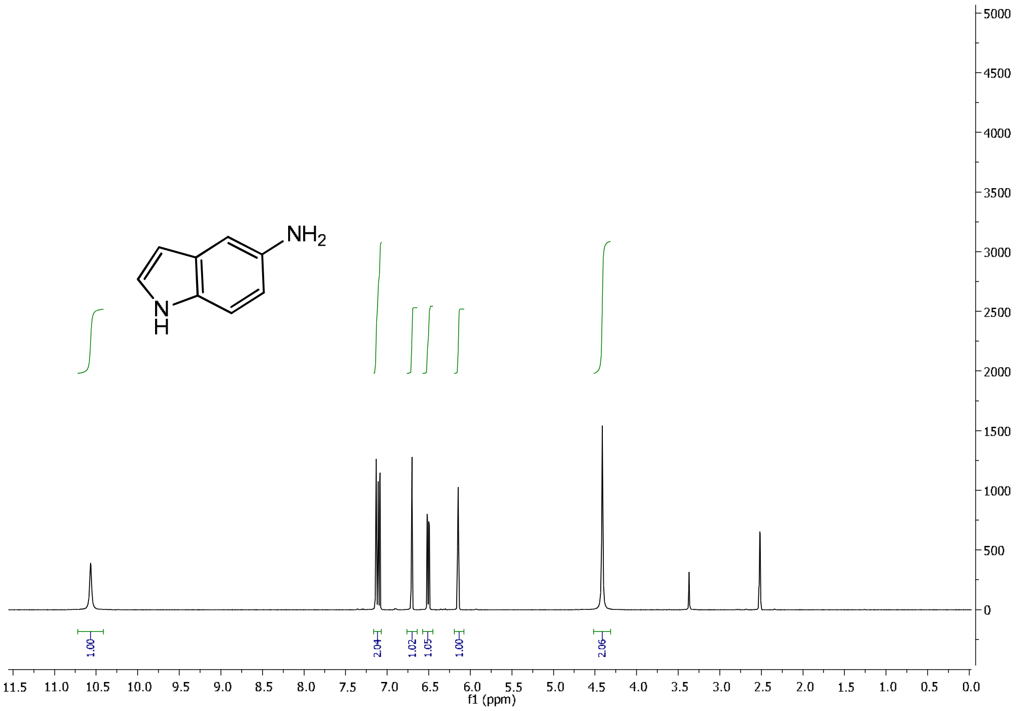

^{13}C-NMR (101 MHz, DMSO-d$_6$)

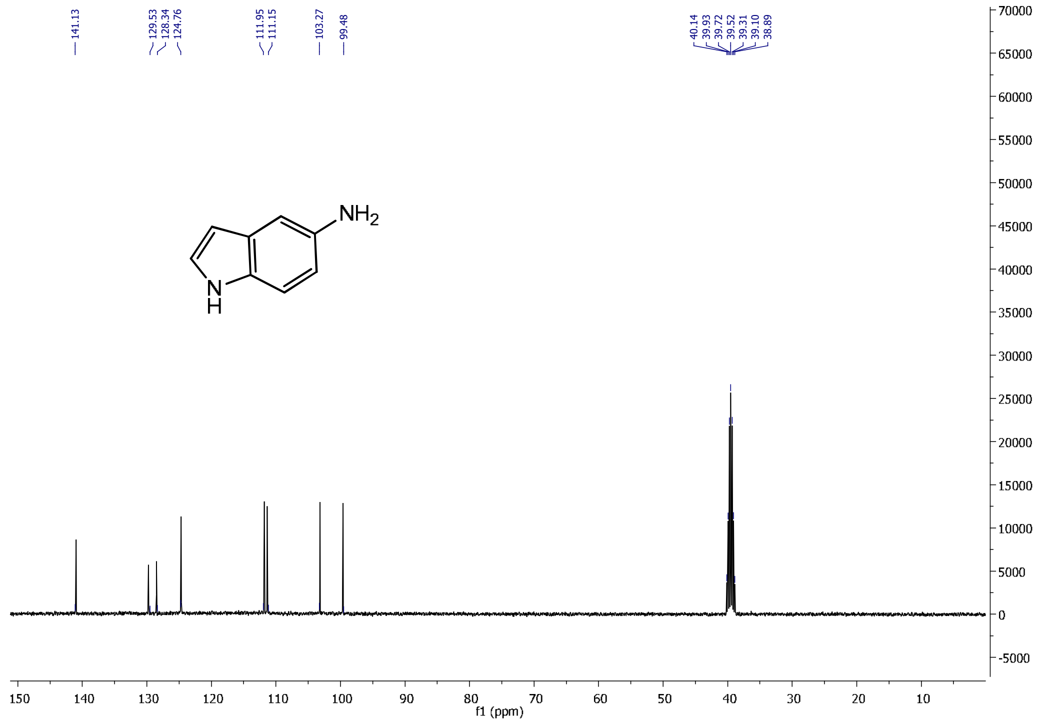

1-(3-bromopropyl)-1H-indole (4c): ^{1}H-NMR (400 MHz, DMSO-d_6)

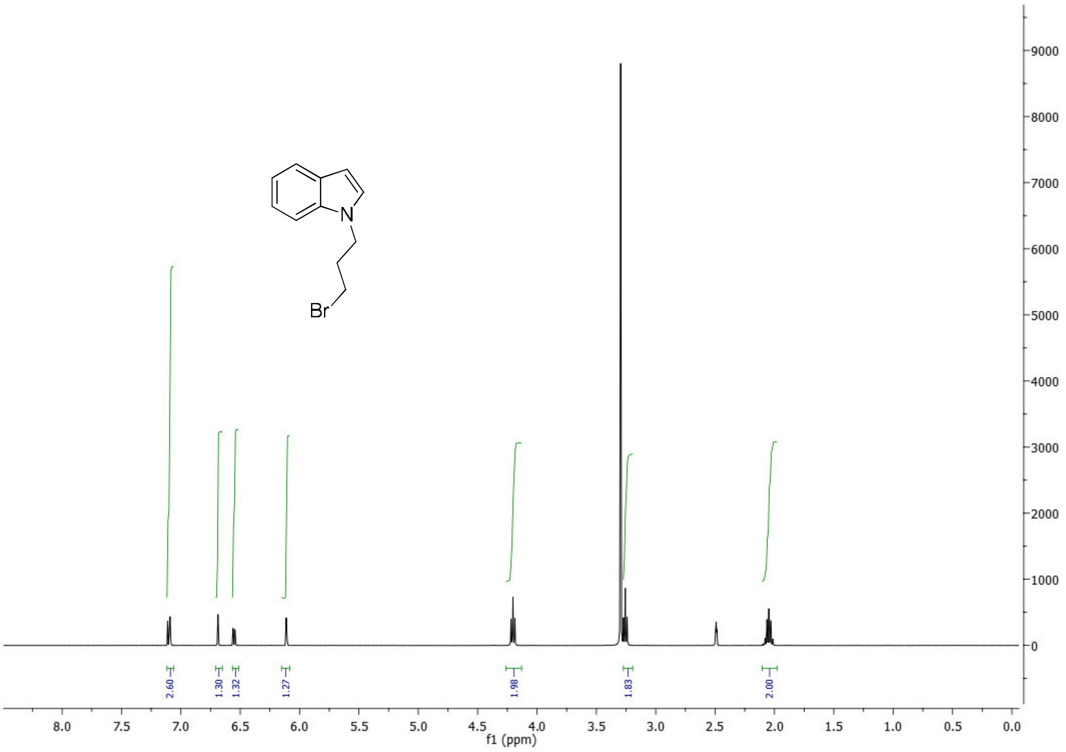

1-(3-(pyrrolidin-1-yl)propyl)-1H-indole (5b) ^{1}H-NMR (400 MHz, DMSO-d$_6$)

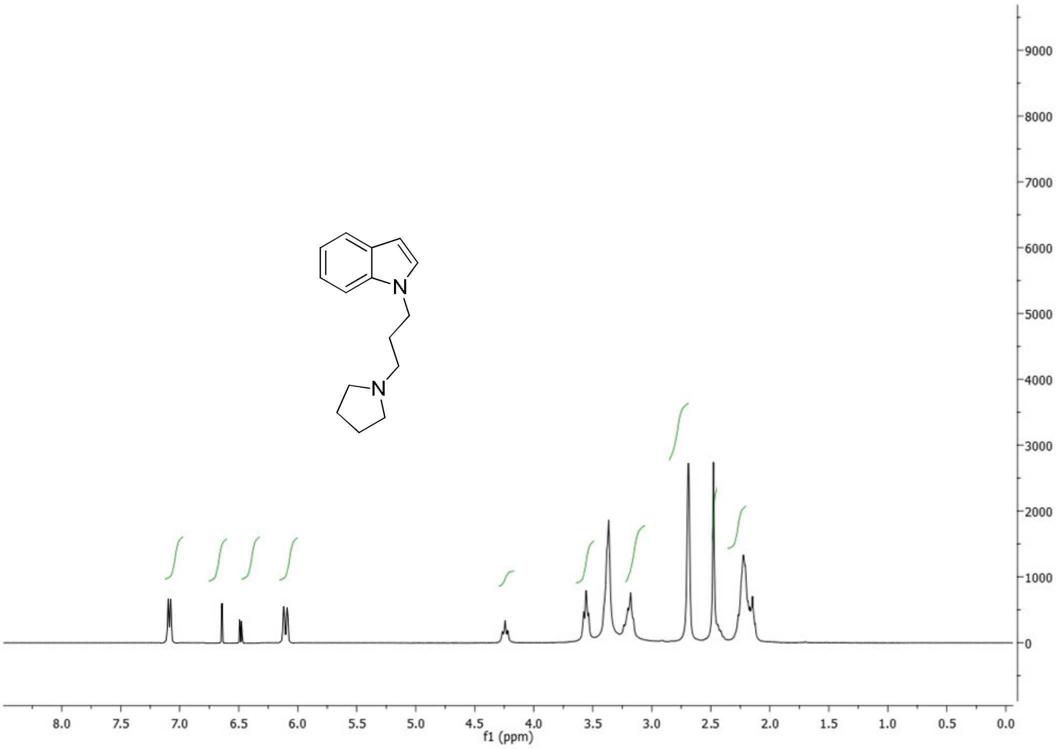

^{13}C-NMR (101 MHz, DMSO-d$_6$)

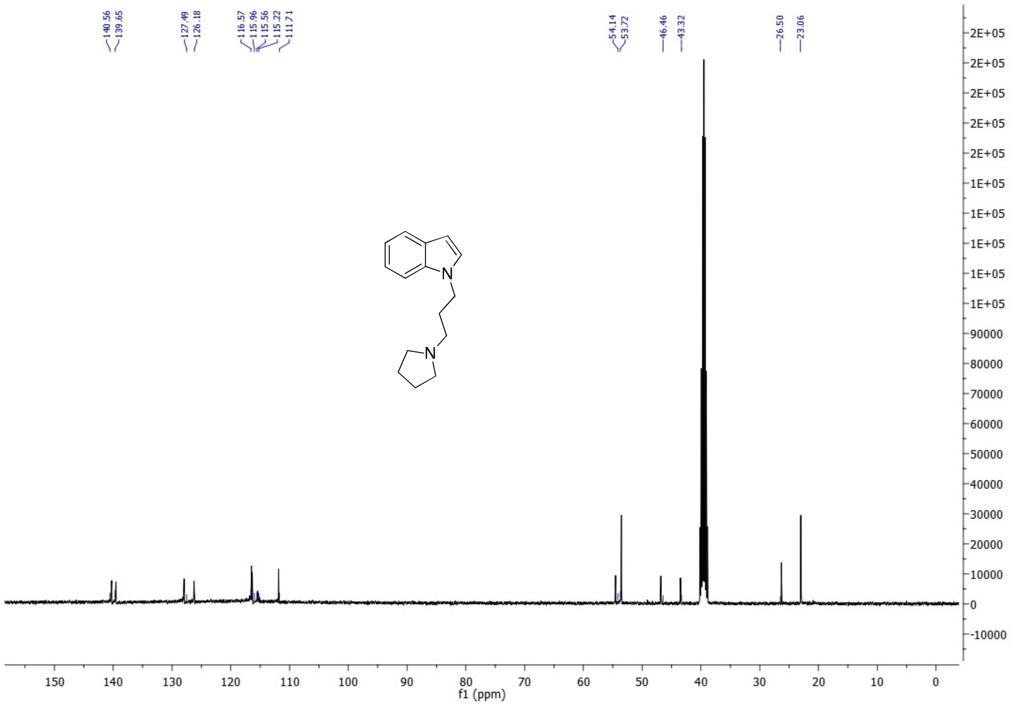

Supplementary data and HRMS Spectra

1-methyl-5-Amino-1H-indole (3)

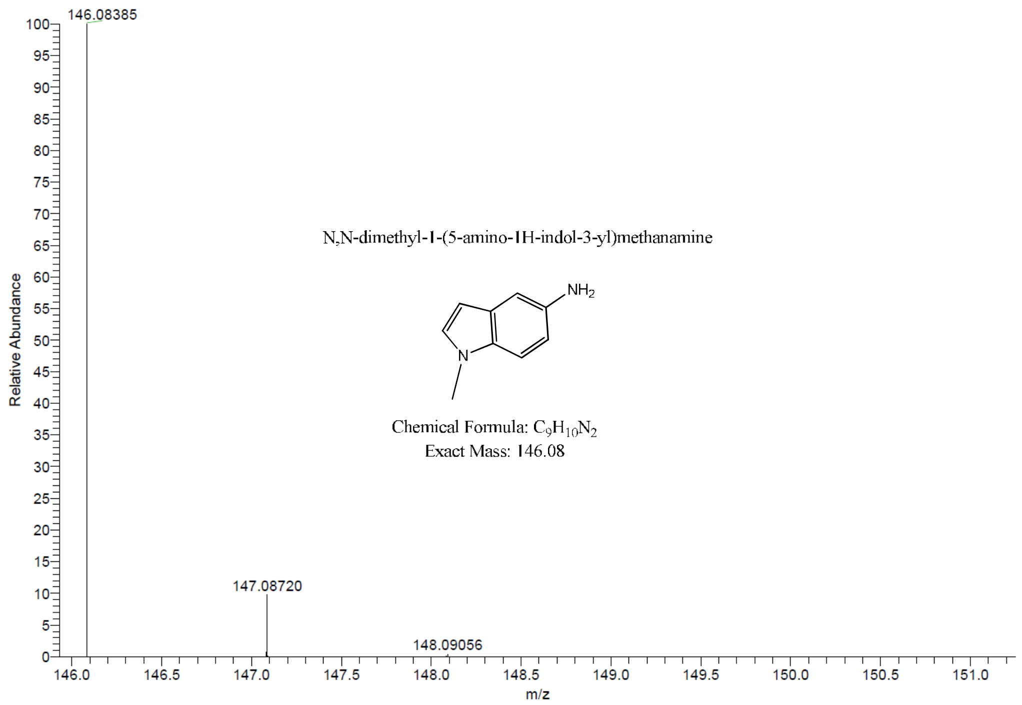

5-nitro-1-(3-bromopropyl)-1H-Indole (4a)

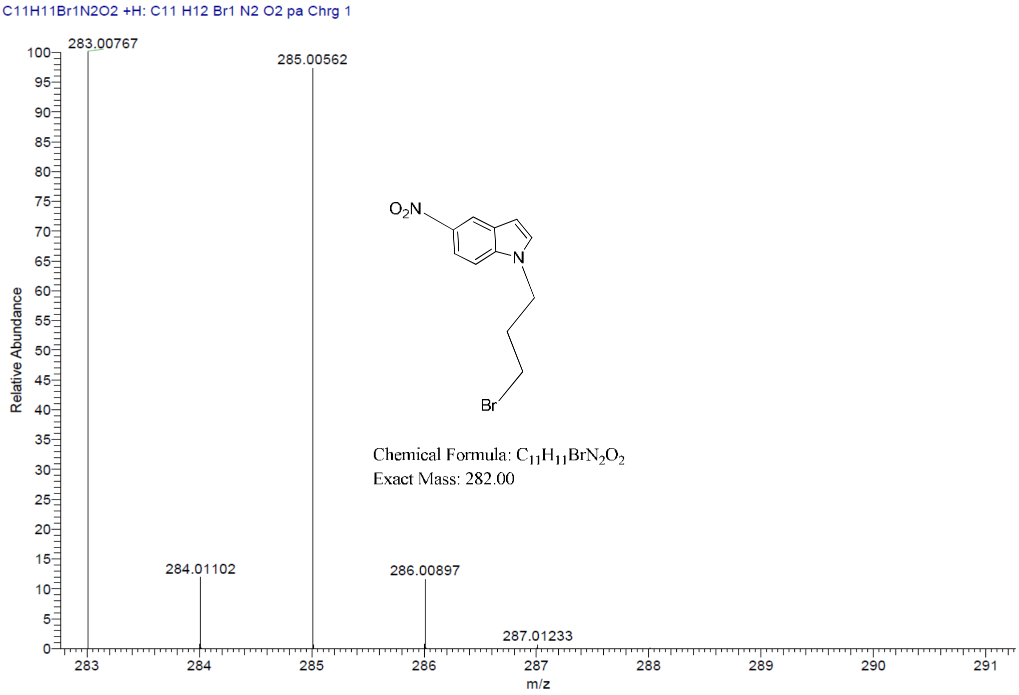

1, 3-bis(5-nitro-1H-indol-1-yl)propane (17a)

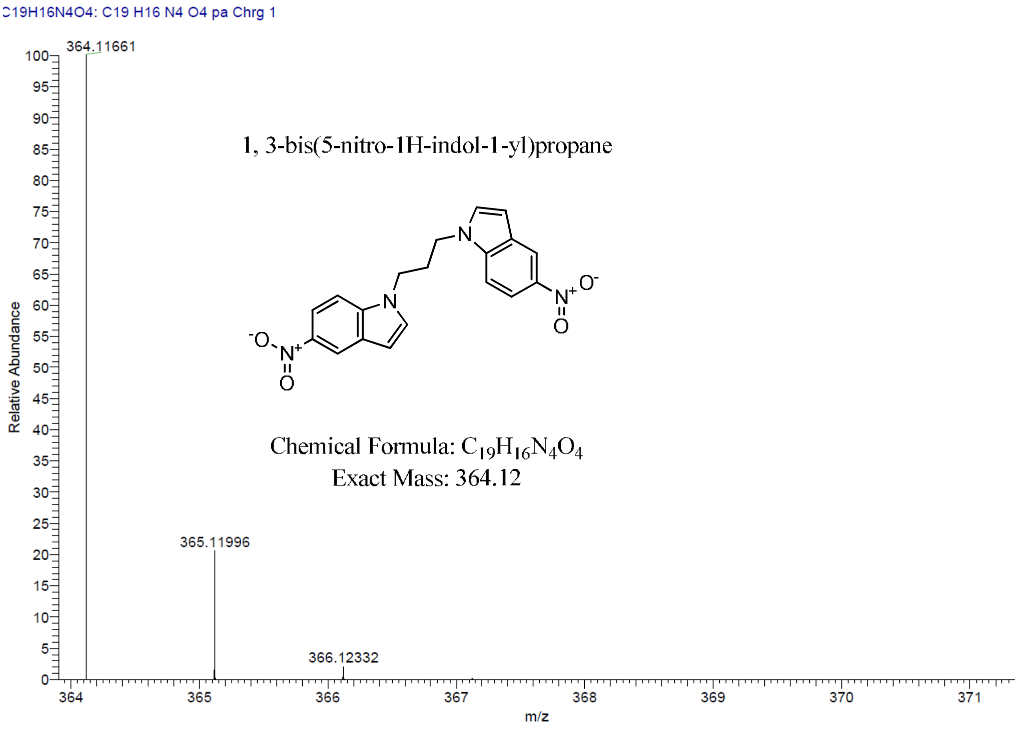

1-(2-bromoethyl)-5-nitro-1H-indole (4b)

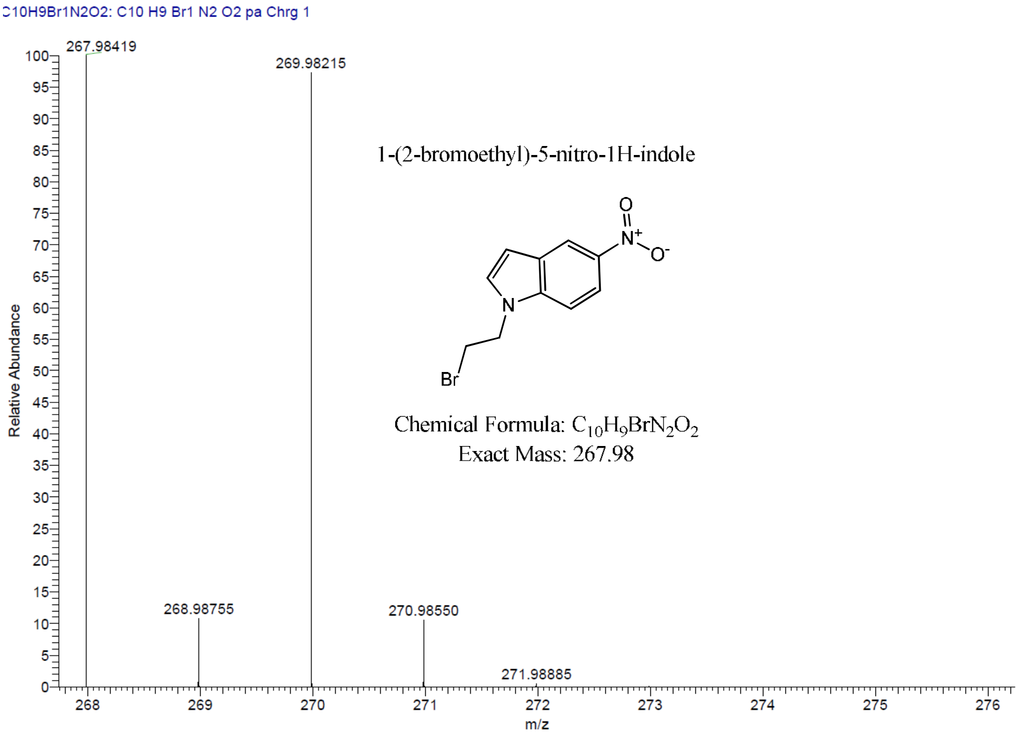

5-nitro-1-(3-(pyrrolidin-1-yl) propyl)-1H-indole (5)

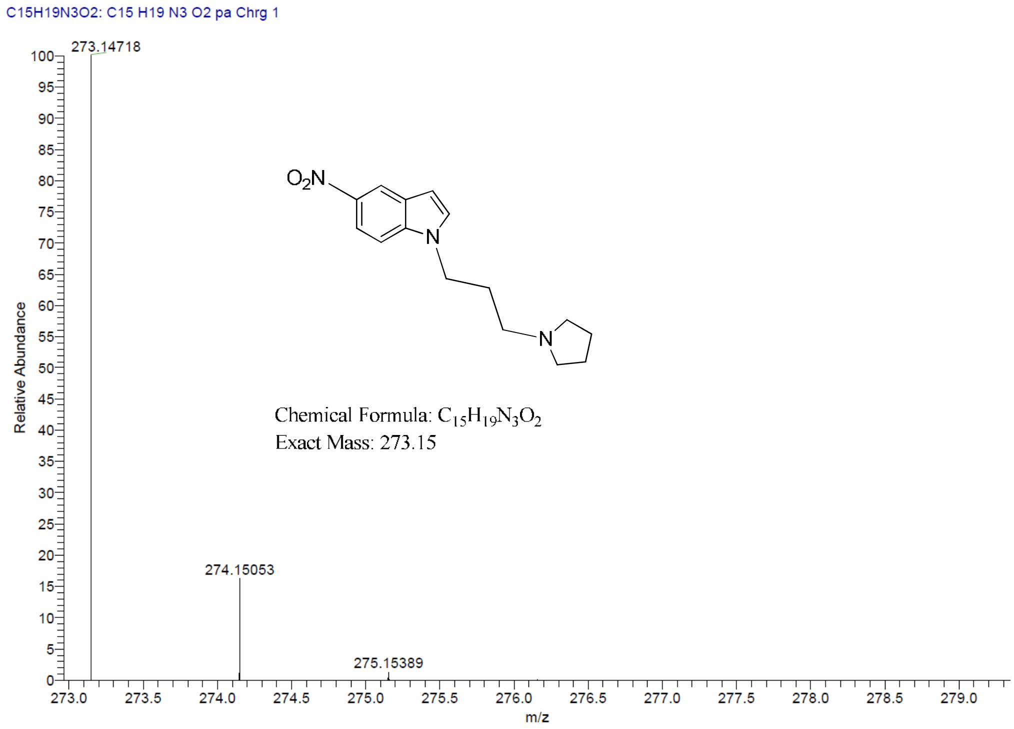

8-carboxaldehyde-5-nitro-1-[3-(1-pyrrolidinyl)propyl]-1H-Indole (6)

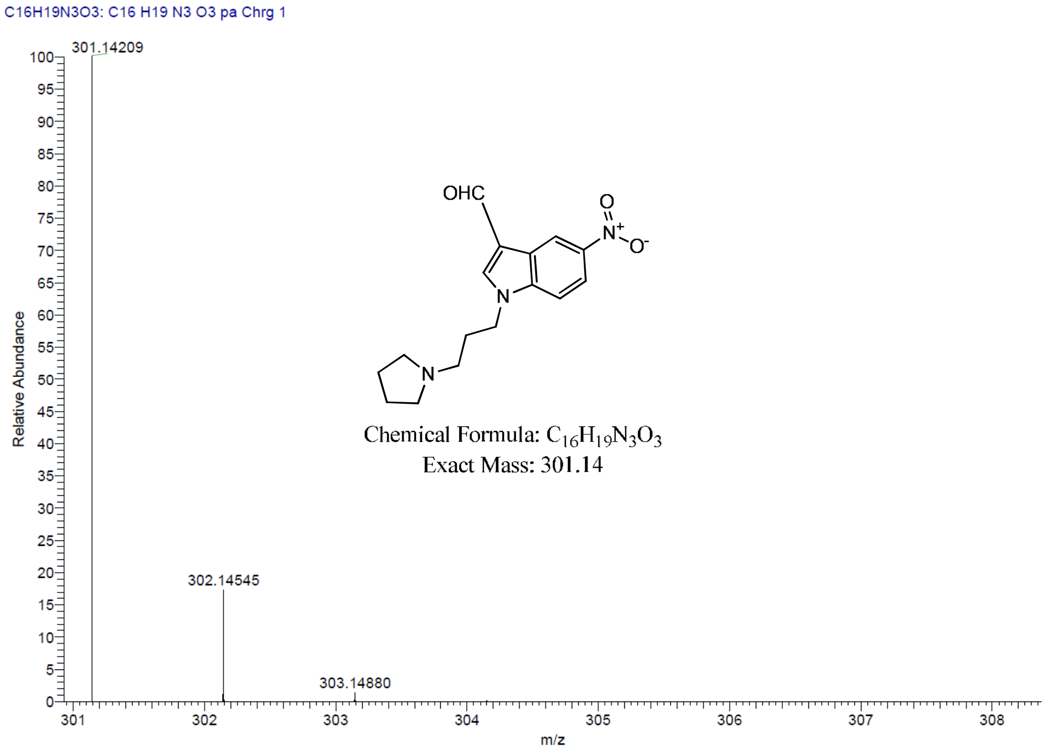

N-((5-nitro-1-(3-(pyrrolidin-1-yl)propyl)-1H-indol-3-yl)methyl)-2-(pyrrolidin-1-yl)ethanamine (7)

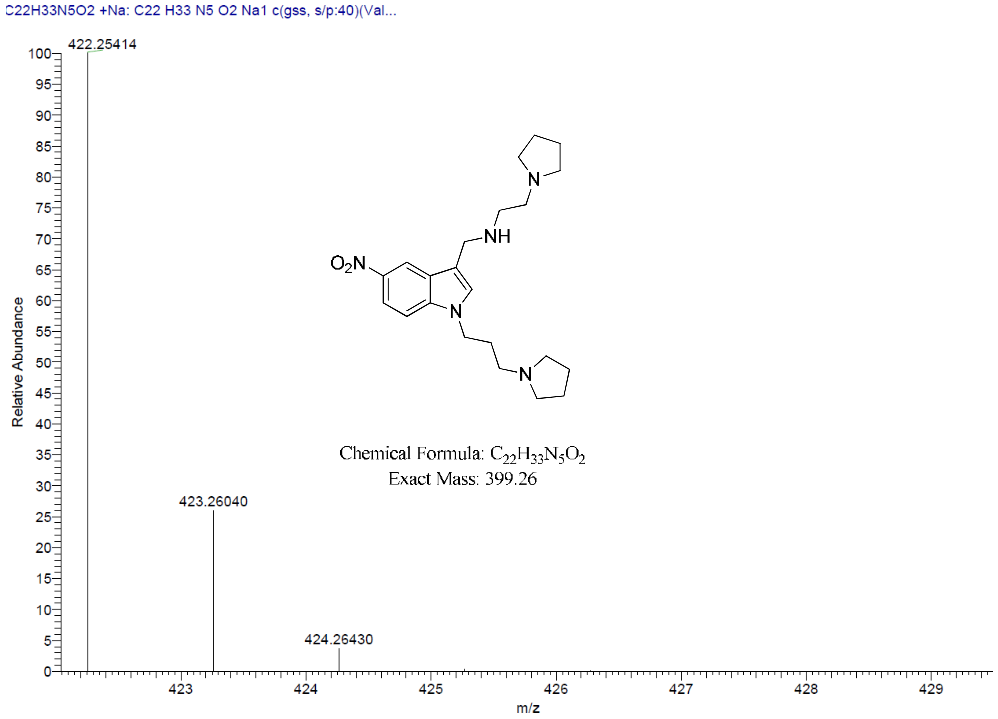

5-Nitroindole-3-carboxaldehyde (8)

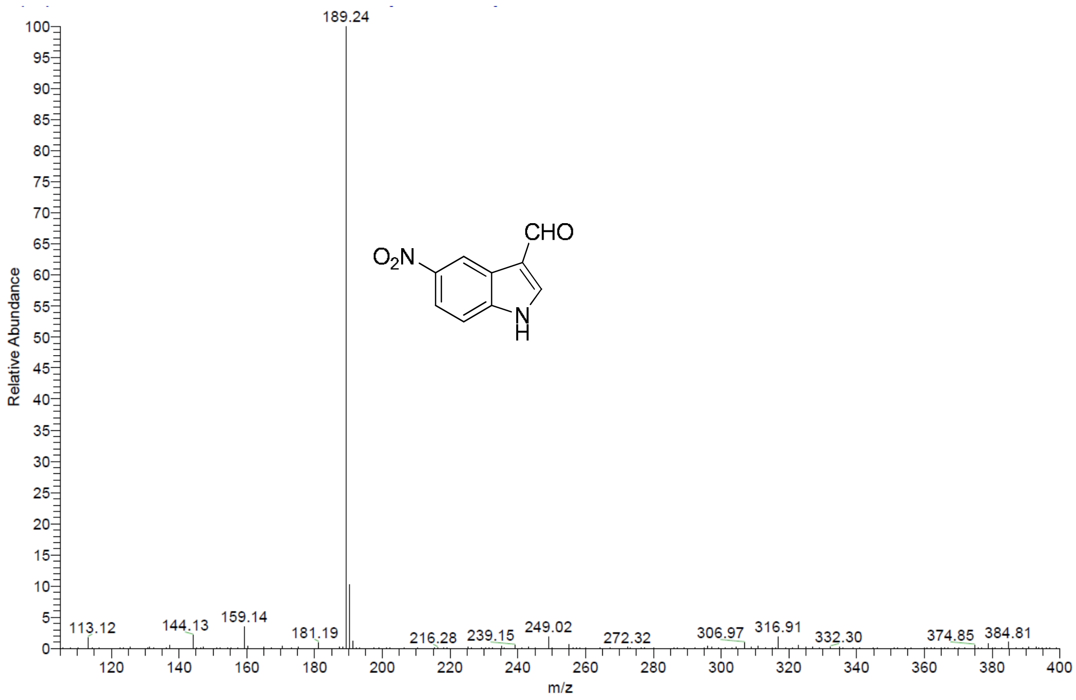

N,N-dimethyl-1-(5-nitro-1H-indol-3-yl)methanamine (9)

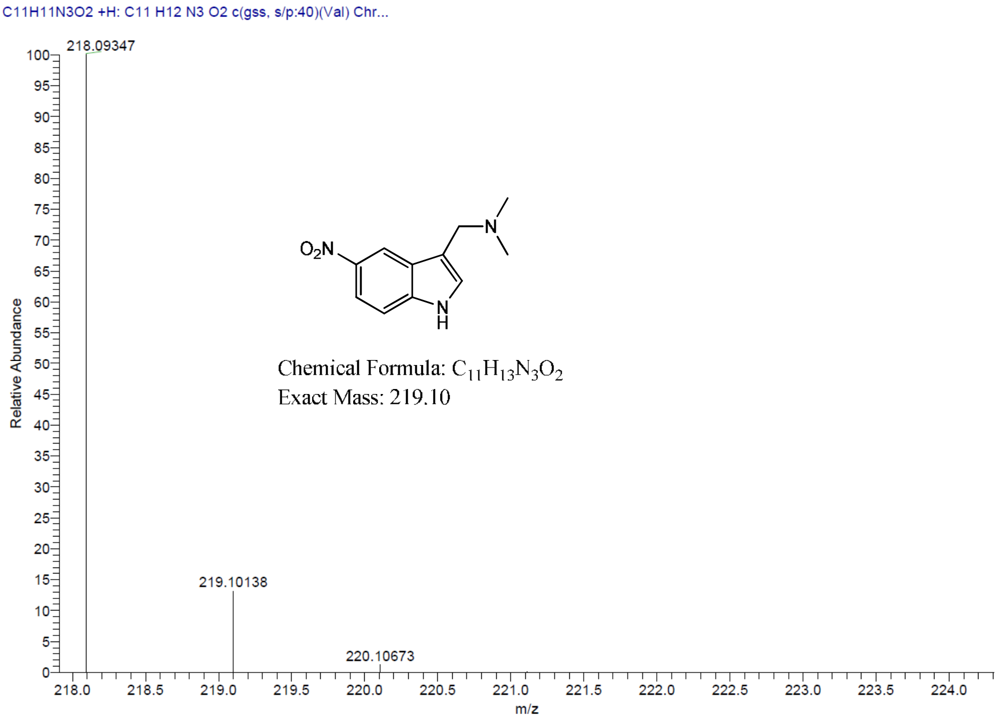

N,N-dimethyl-1-(5-amino-1H-indol-3-yl)methanamine (9a)

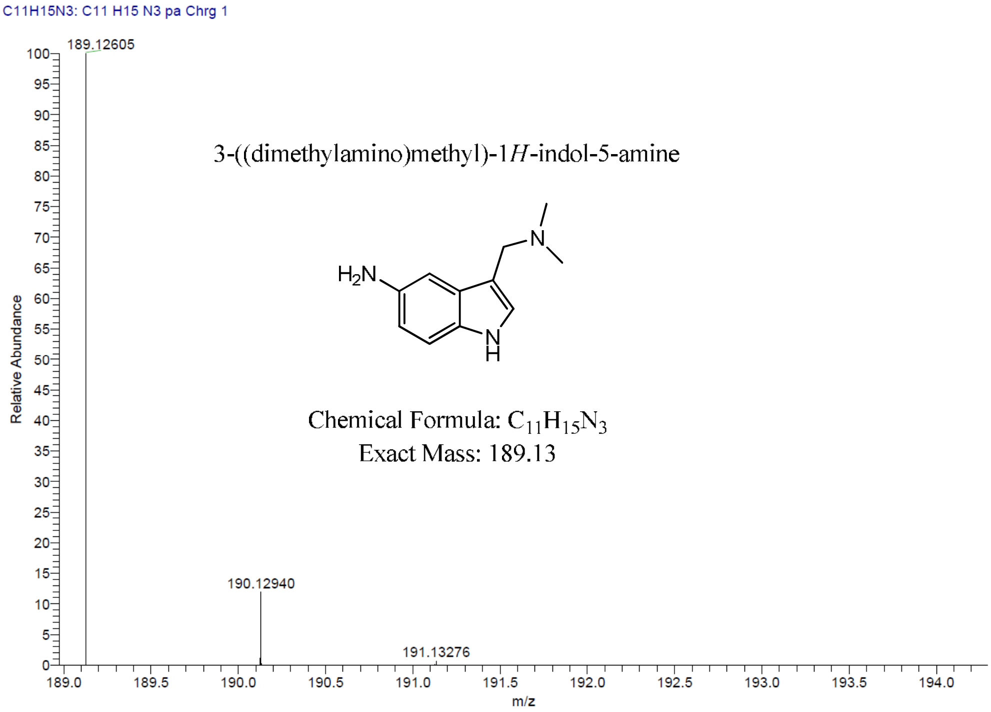

N1,N1-dimethyl-N2-((5-nitro-1H-indol-3-yl)methyl)ethane-1,2-diamine (10)

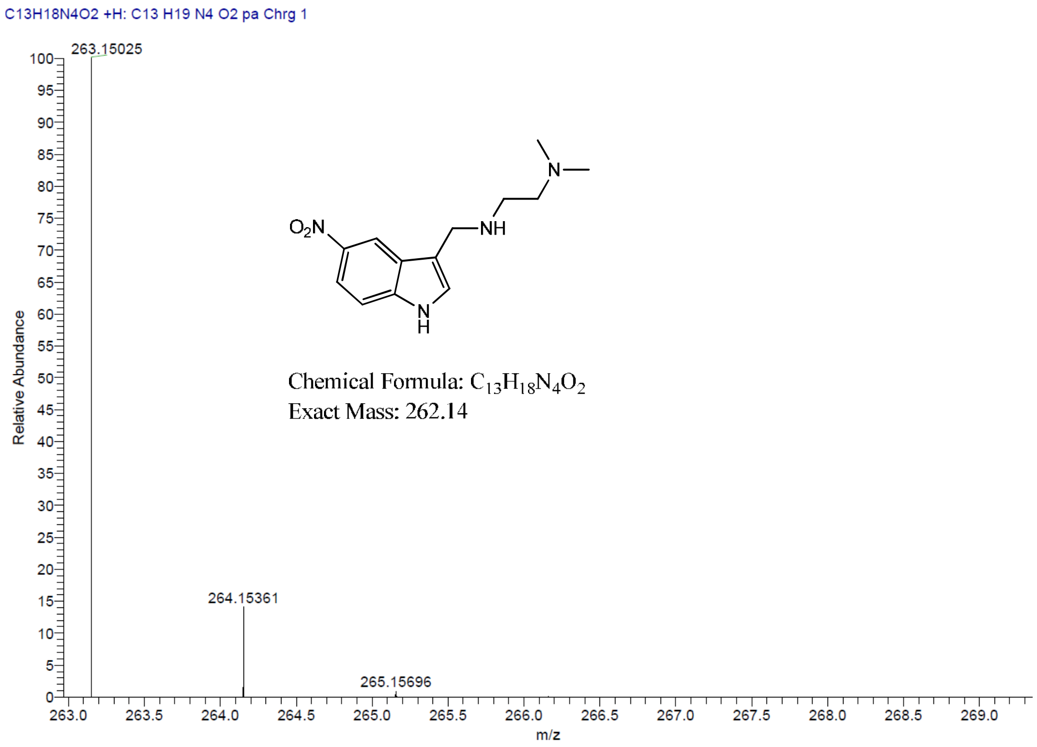

N1, N1-diethyl-N2-((5-nitro-1H-indol-3-yl)methyl)ethane-1,2-diamine (11)

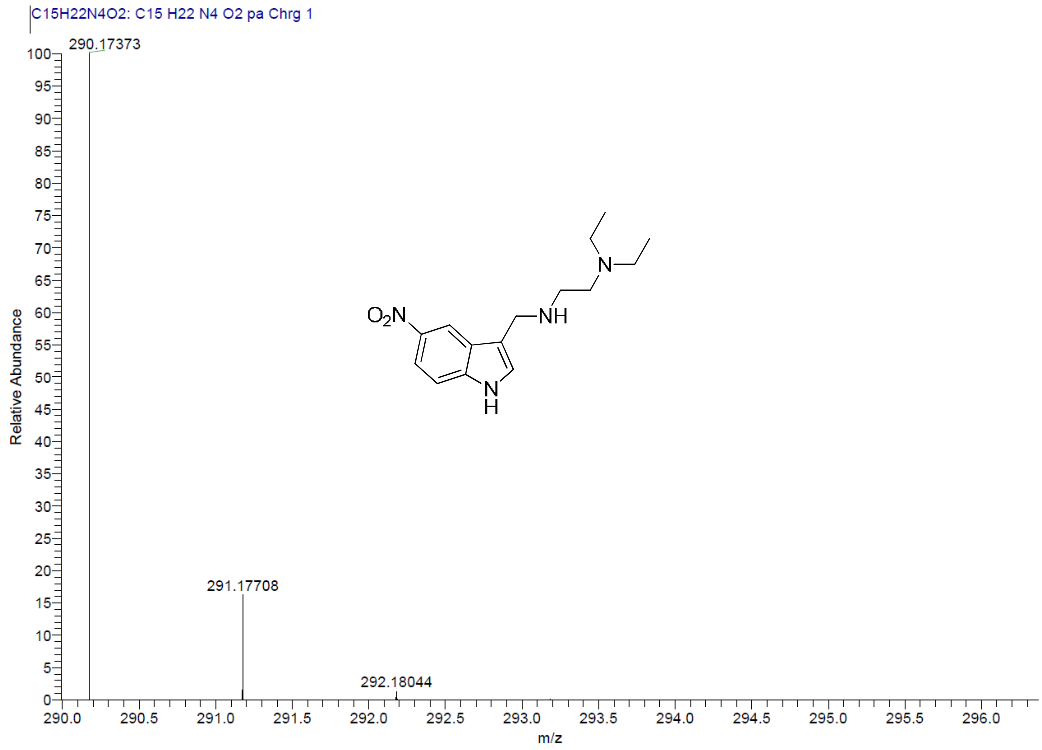

N-((5-nitro-1H-indol-3-yl)methyl)-2-(pyrrolidin-1-yl)ethanamine (12)

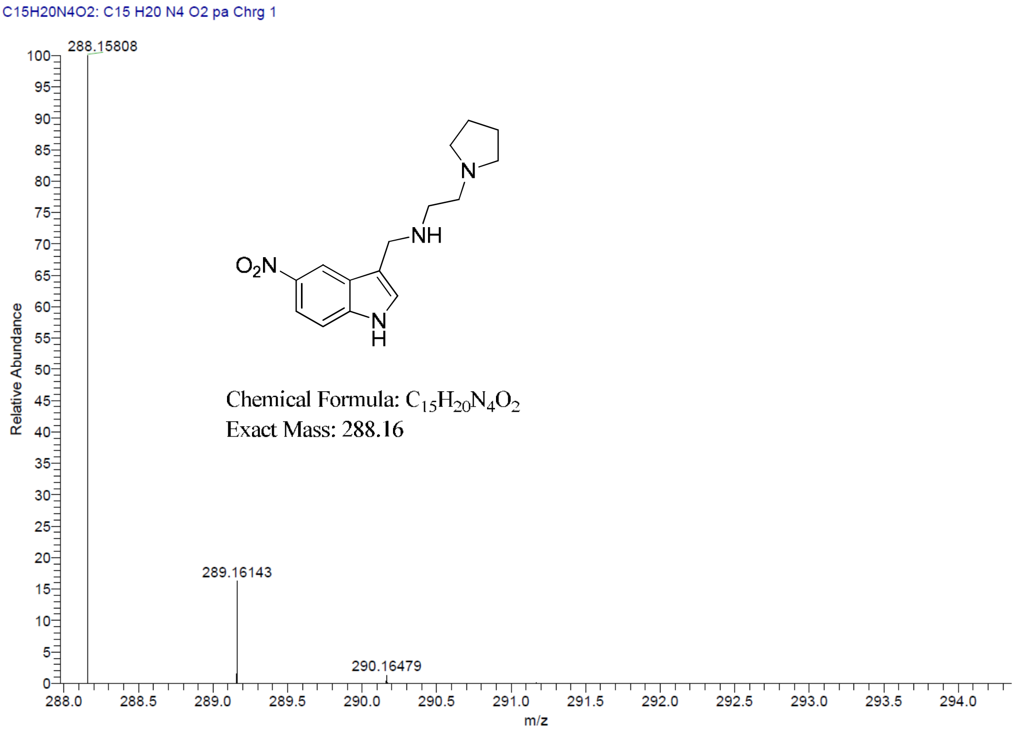

1-(3-azidopropyl)-5-nitro-1H-indole (13)

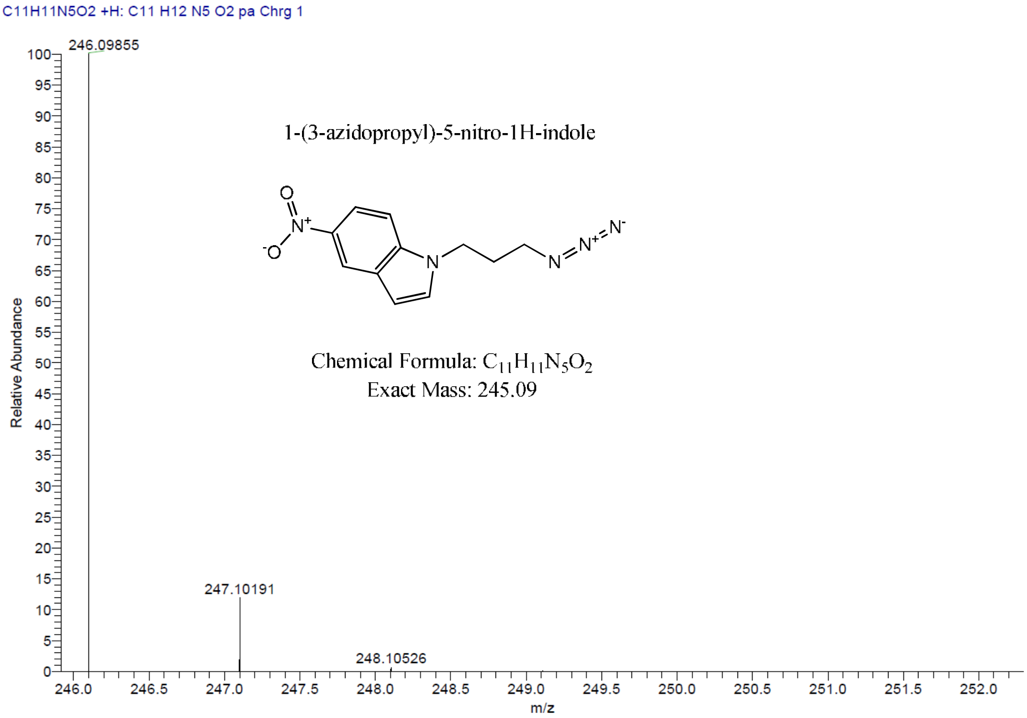

8-carboxaldehyde-5-nitro-1-(3-azidopropyl)-1H-Indole (15)

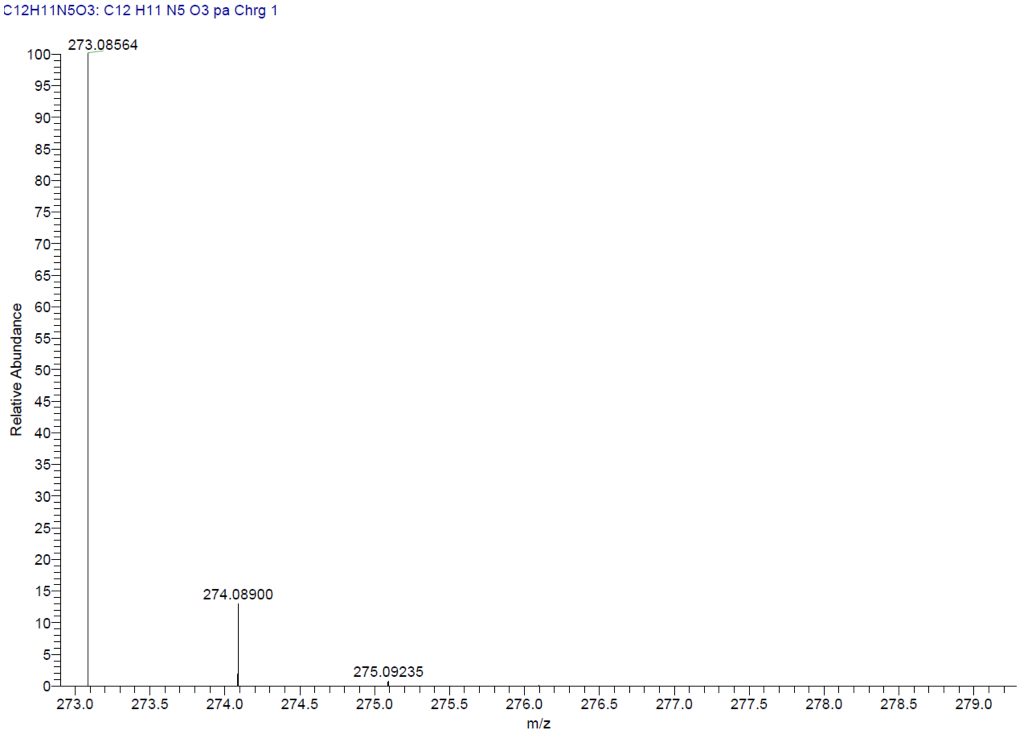

N,N-dimethyl-N'-[[5-nitro-1-(3-Azidopropyl)-1H-indole]methyl]-1,2-Ethanediamine (16)

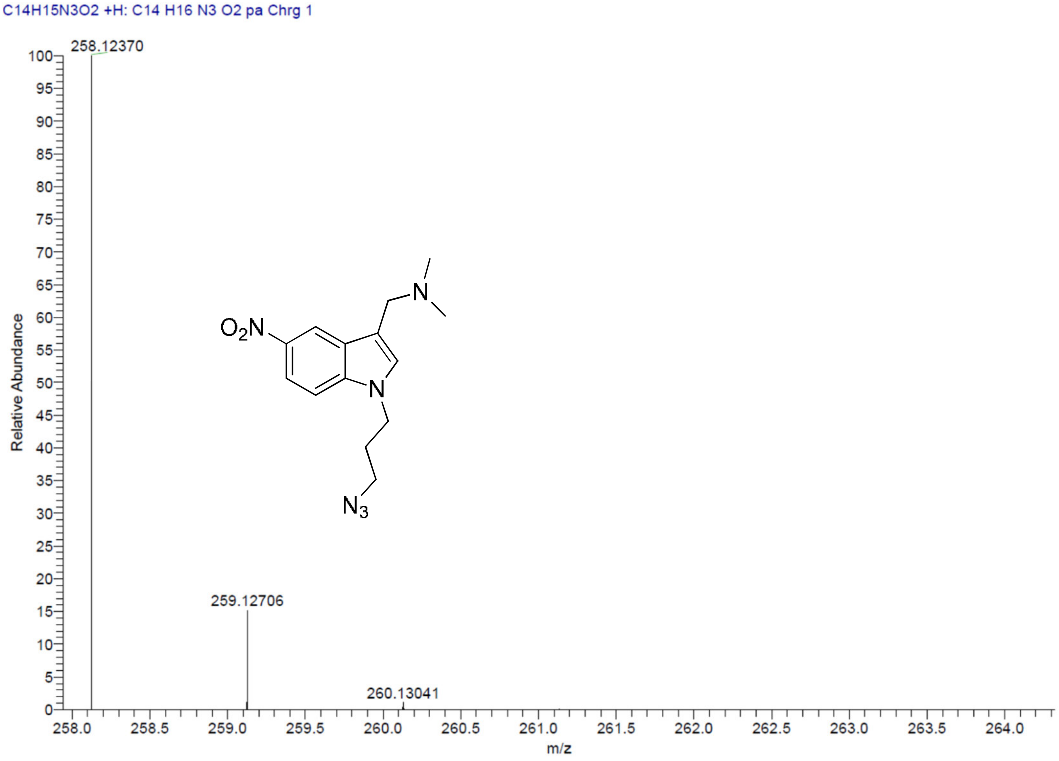

N,N-dimethyl-N'-[[5-nitro-1-(3-Azidopropyl)-1H-indole]methyl]-1,2-Ethanediamine (16a)

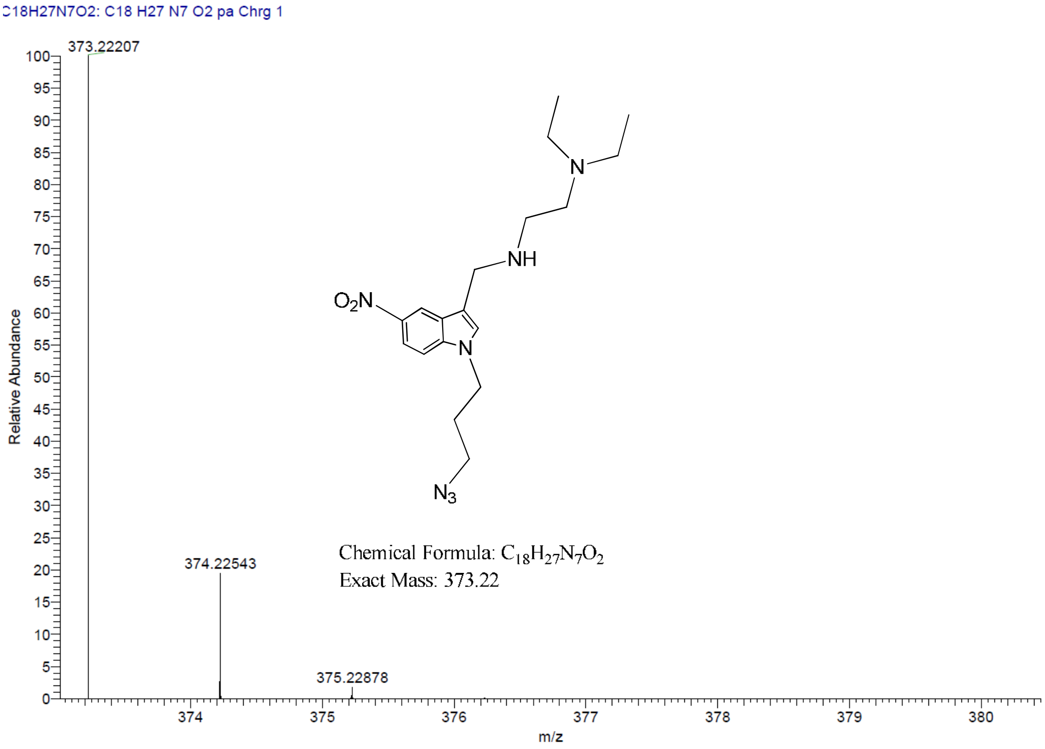

1H-indole-3-carbaldehyde (2a)

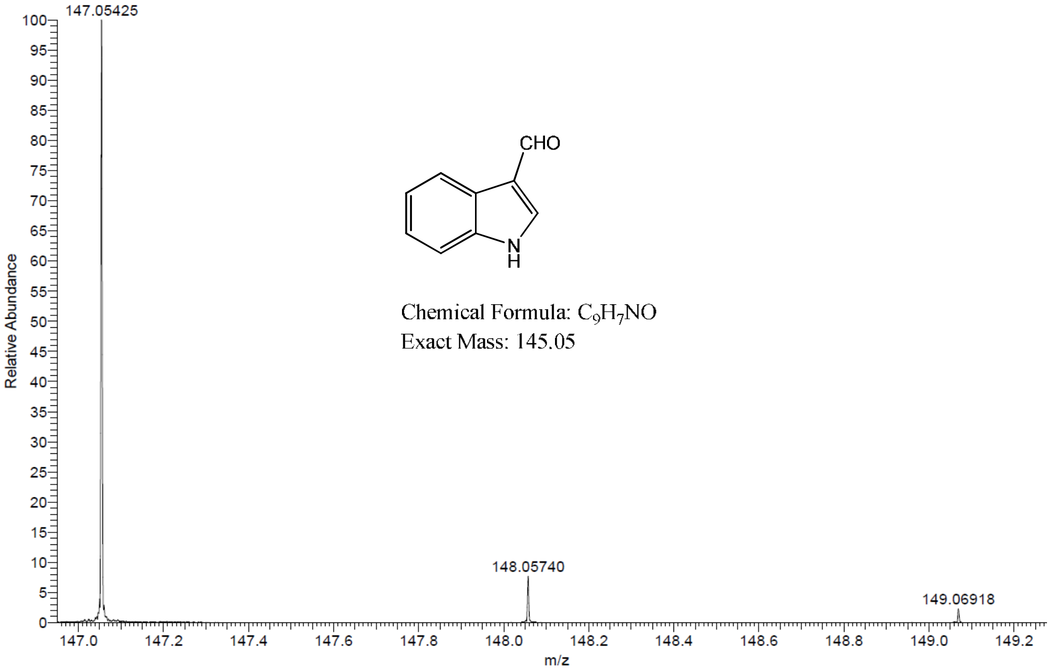

1-(1H-indol-3-yl)-N,N-dimethylmethanamine (3a)

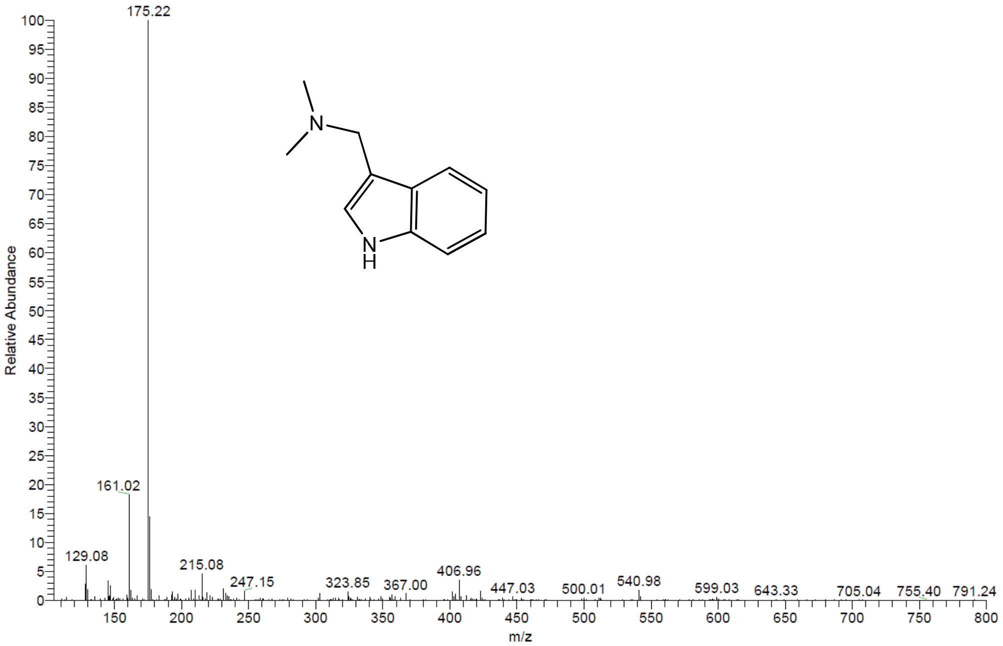

N1-((1H-indol-3-yl)methyl)-N2-(2-((2-((2-((2 aminoethyl)amino)ethyl)amino)ethyl)amino)ethyl)ethane-1,2-diamine (3b)

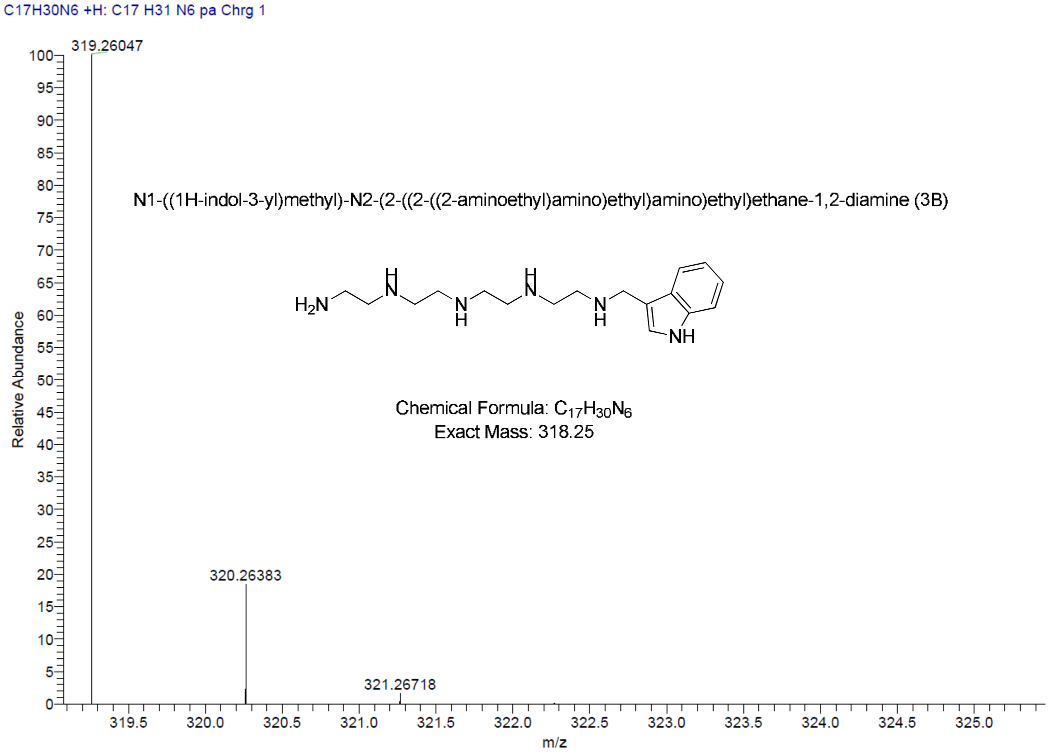

1-(1H-indazol-3-yl)-N,N-dimethylmethanamine (23a)

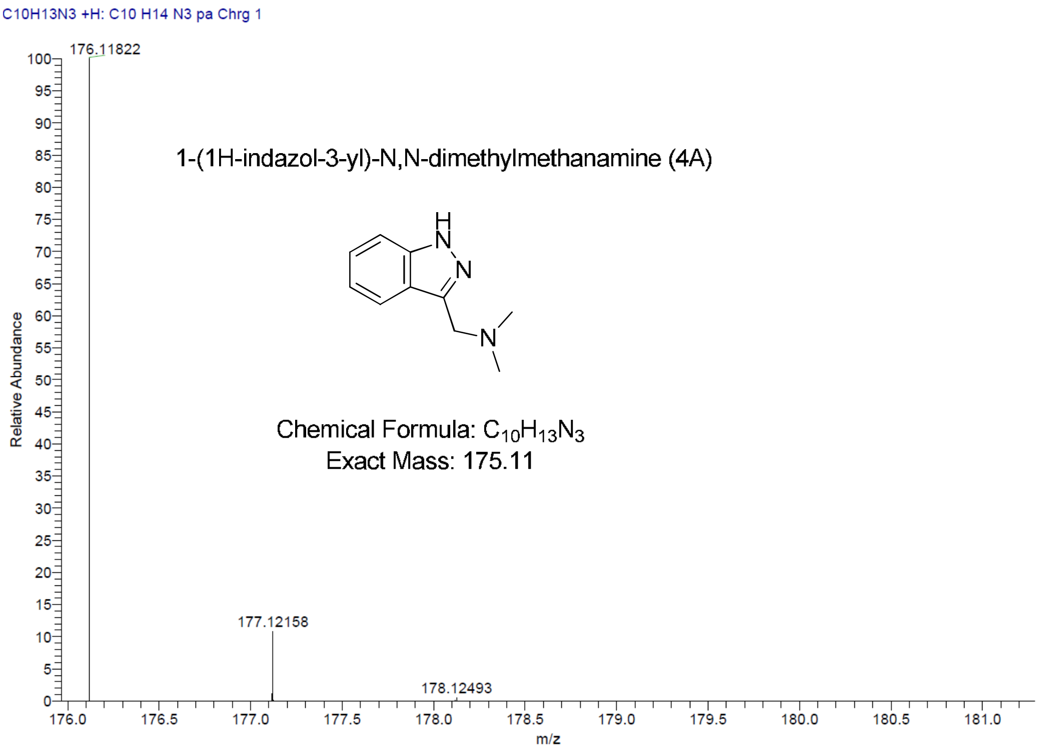

N1-((1H-indazol-3-yl)methyl)-N2-(2-((2-((2-aminoethyl)amino)ethyl)amino)ethyl)ethane-1,2-diamine (23b)

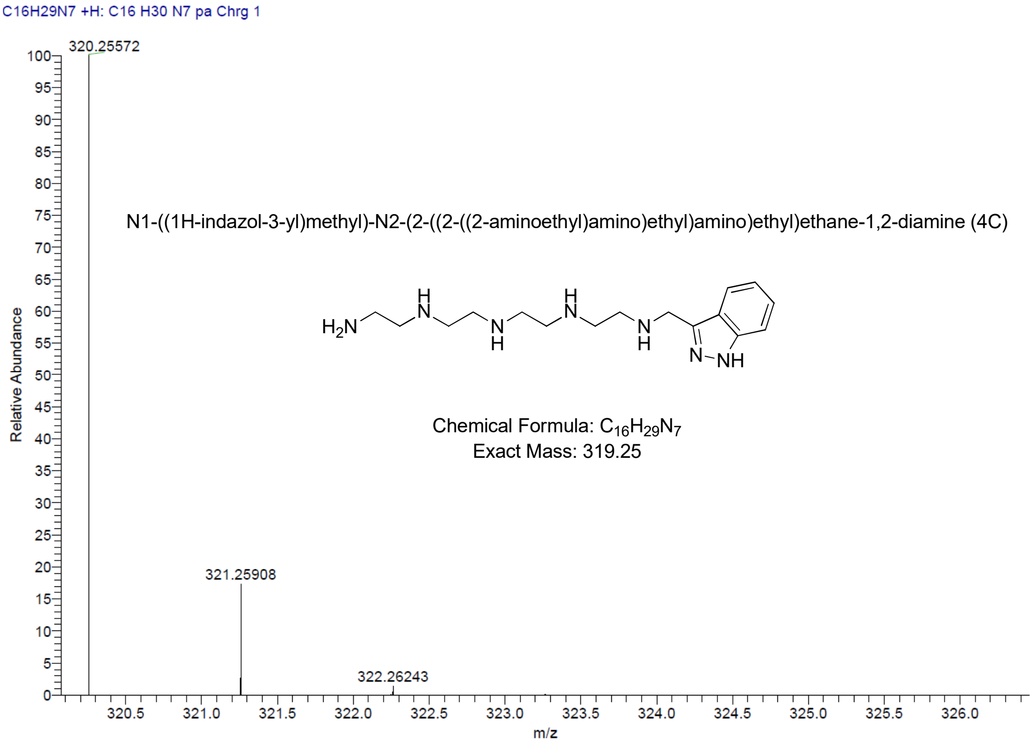

N-((1H-indazol-3-yl)methyl)-2-(pyrrolidin-1-yl)ethanamine (23f)

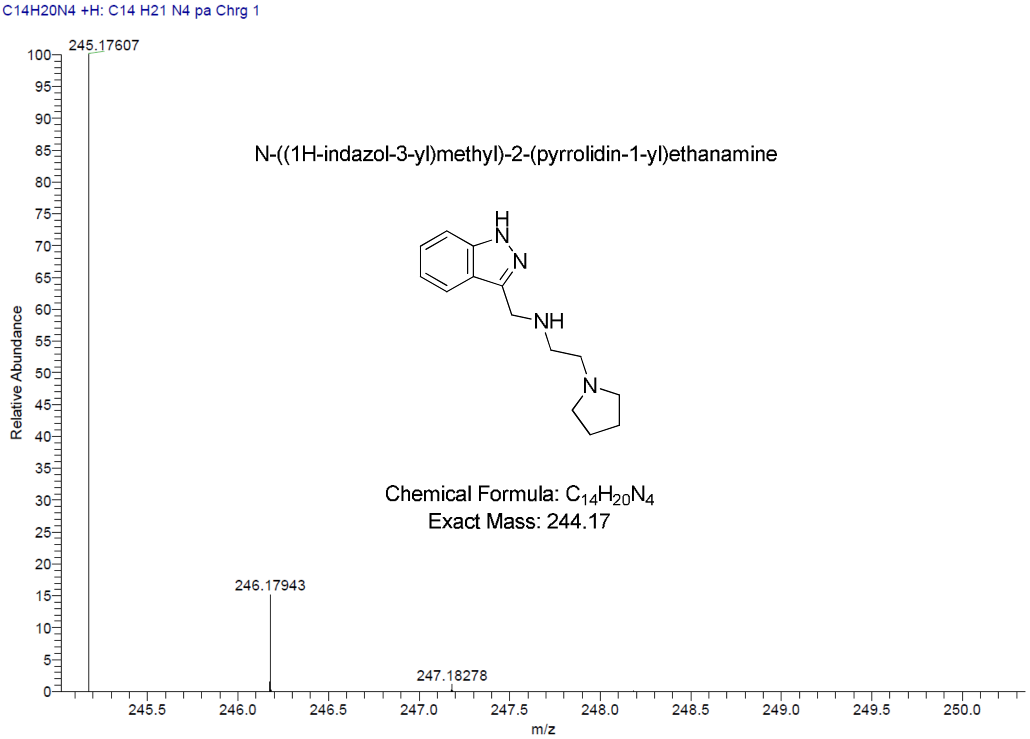

N,N-dimethyl-1-(1H-pyrrolo[2,3-b]pyridin-3-yl)methanamine (24a)

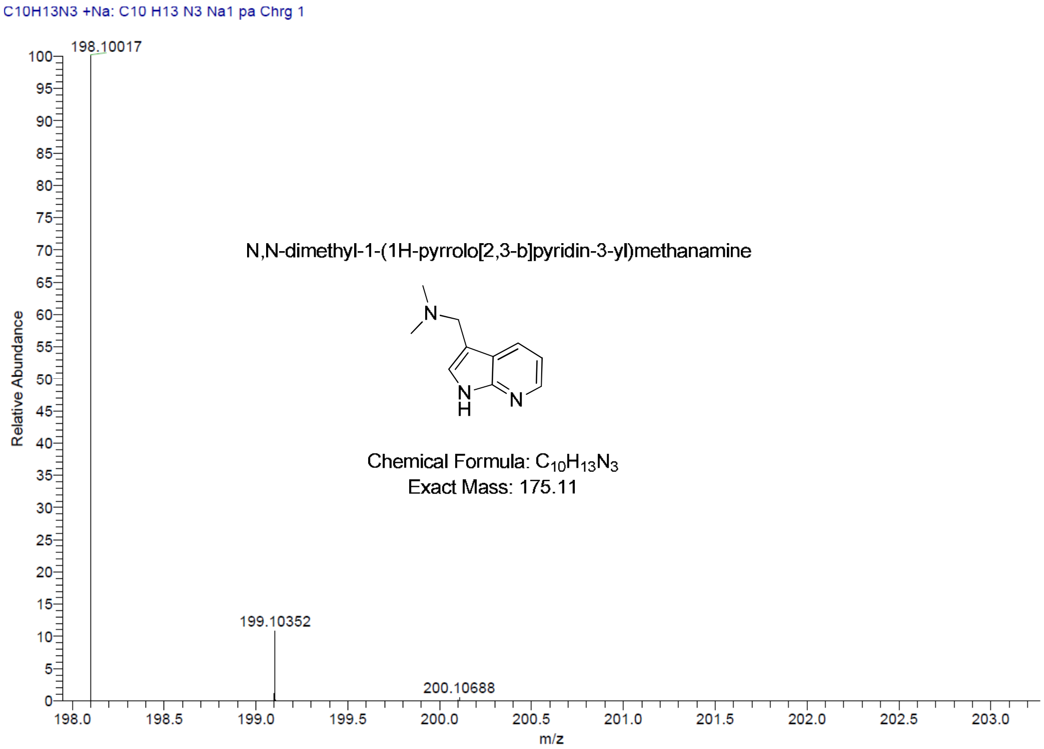

N1-((1H-pyrrolo[2,3-b]pyridin-3-yl)methyl)-N2-(2-((2-((2-aminoethyl)amino)ethyl)amino)ethyl)ethane-1,2-diamine (24b)

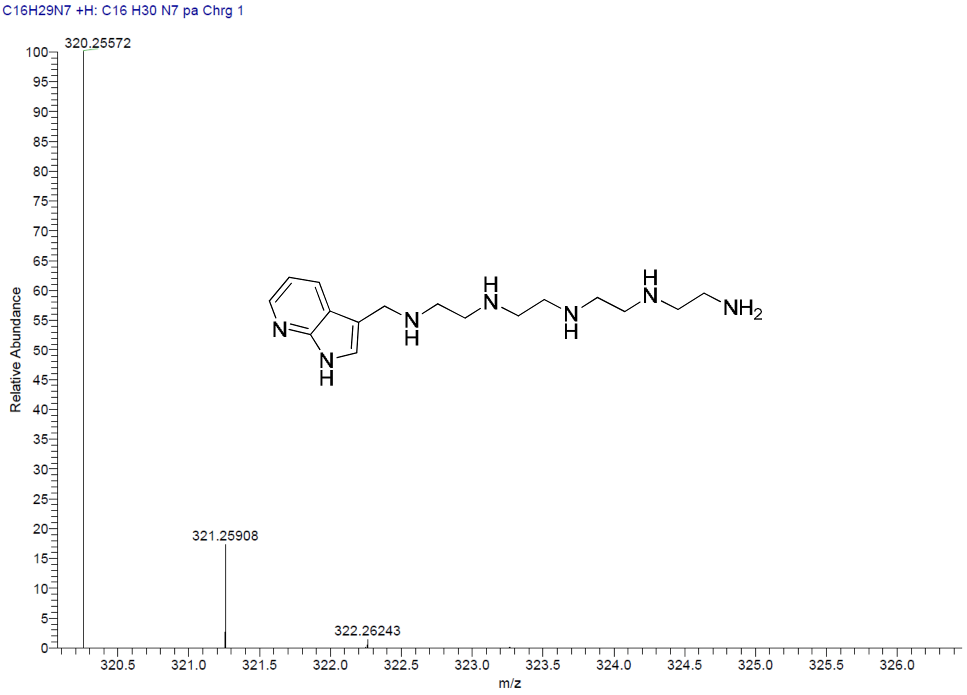

N1-((1H-pyrrolo[2,3-b]pyridin-3-yl)methyl)-N2-(2-((2-aminoethyl)amino)ethyl)ethane-1,2-diamine (24c)

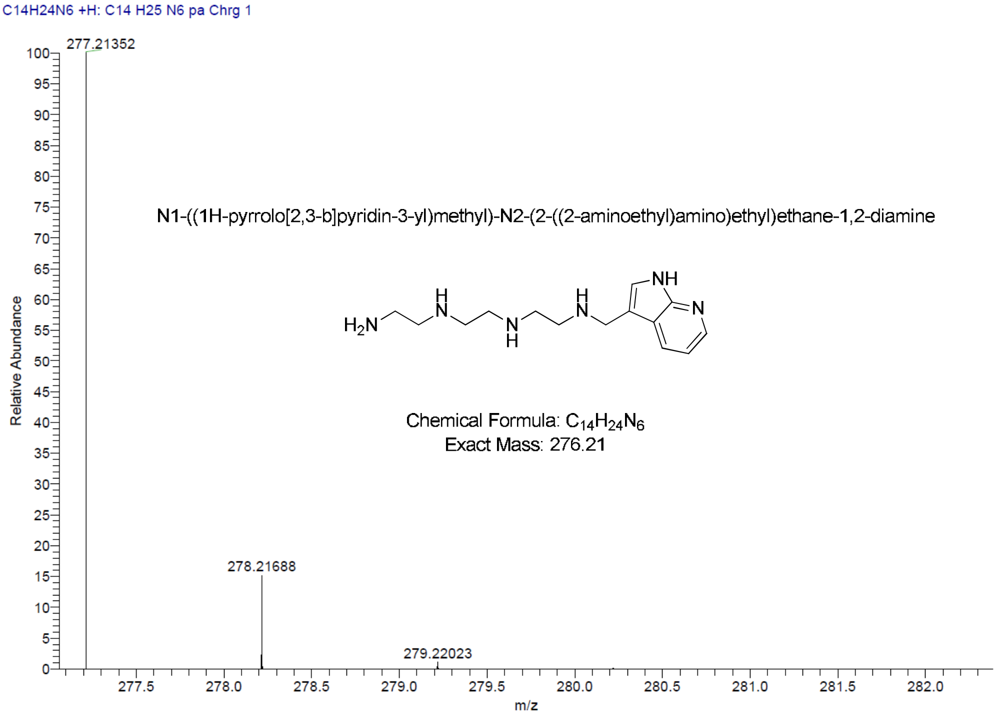

N-((1H-pyrrolo[2,3-b]pyridin-3-yl)methyl)-2-(pyrrolidin-1-yl)ethanamine (24f)

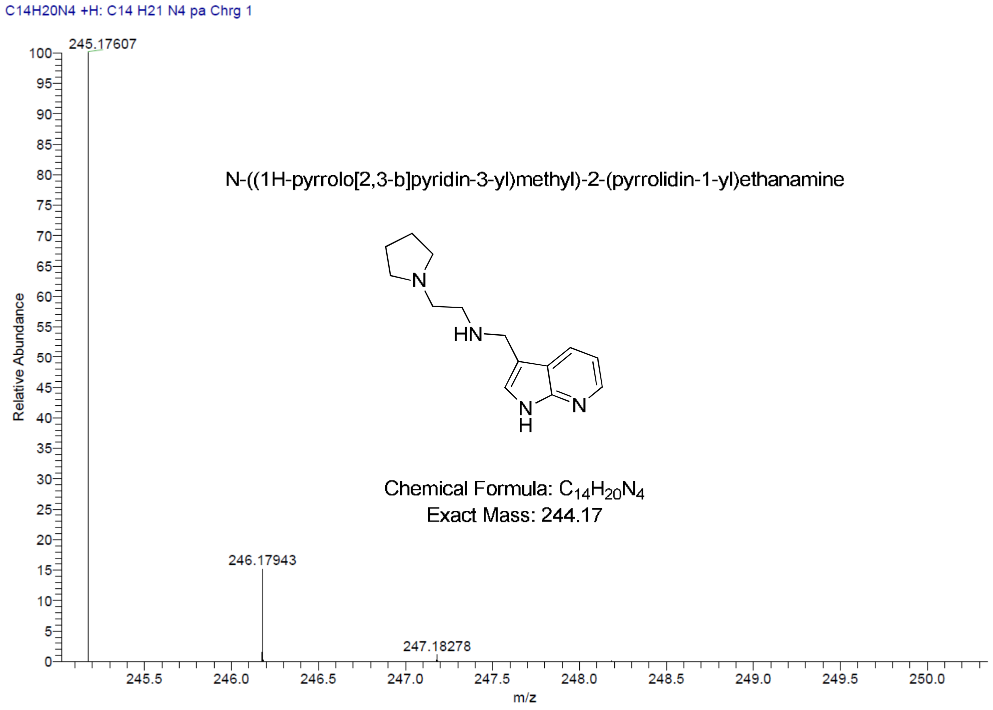

N1-(2-aminoethyl)-N2-(2-((2-((benzo[d][1,3]dioxol-5-ylmethyl)amino)ethyl)amino)ethyl)ethane-1,2-diamine (25b)

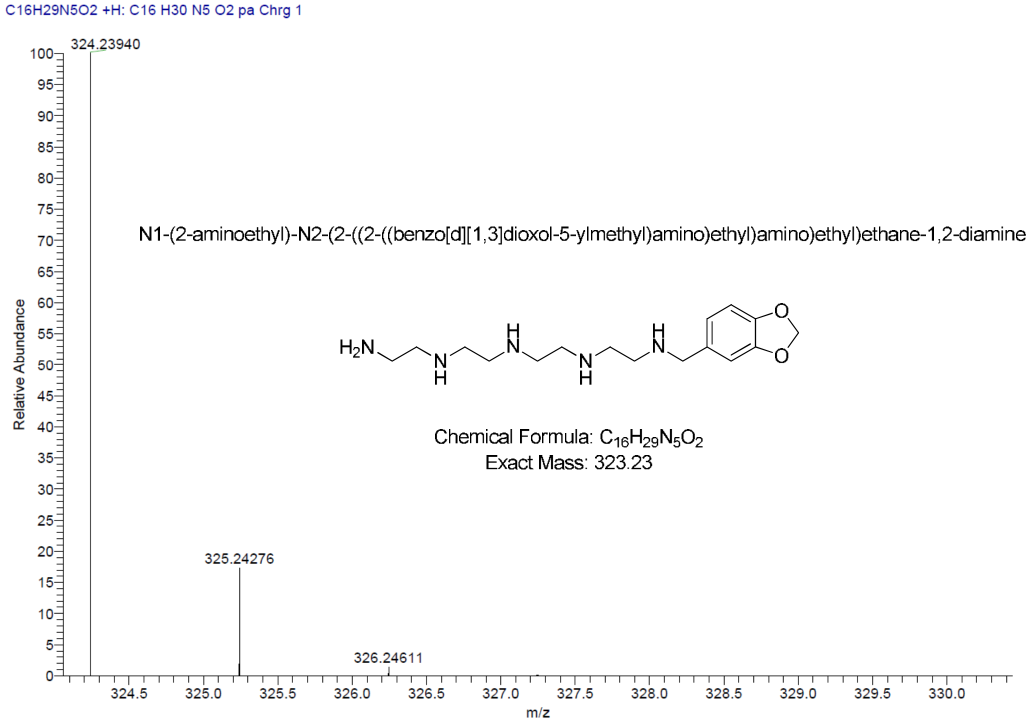

N1-(benzo[d][1,3]dioxol-5-ylmethyl)-N2,N2-dimethylethane-1,2-diamine (25e)

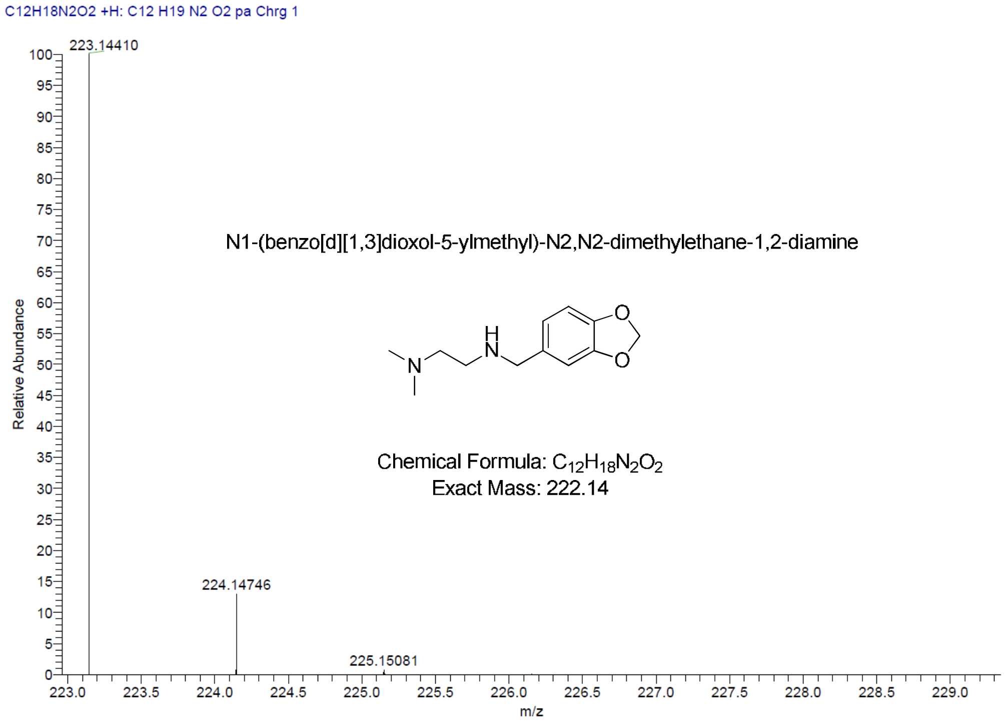

N-(benzo[d][1,3]dioxol-5-ylmethyl)-2-(pyrrolidin-1-yl)ethanamine (25f)

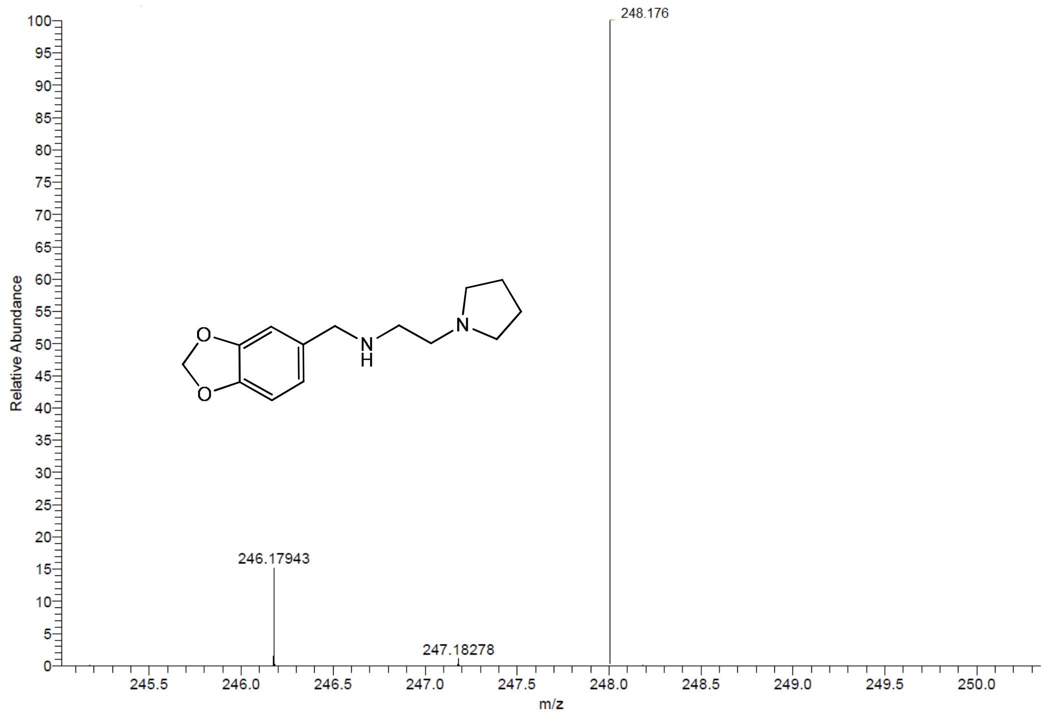

N-(benzo[d][1,3]dioxol-5-ylmethyl)-2-(pyrrolidin-1-yl)ethanamine (13)

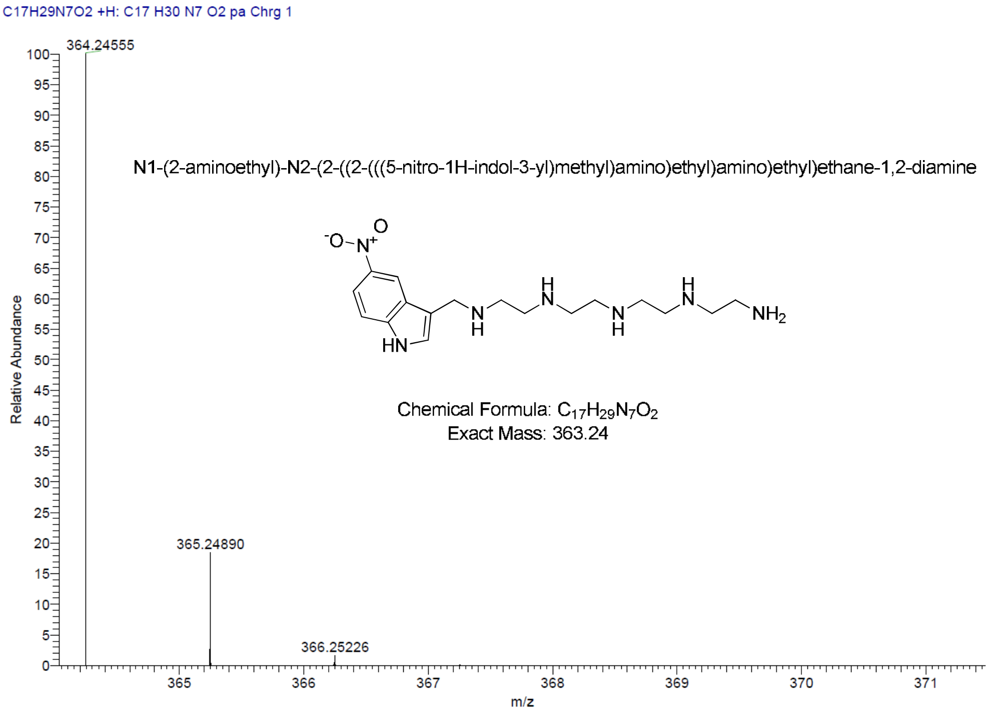

5-nitro-1-(prop-2-yn-1-yl)-1H-indole (19a)

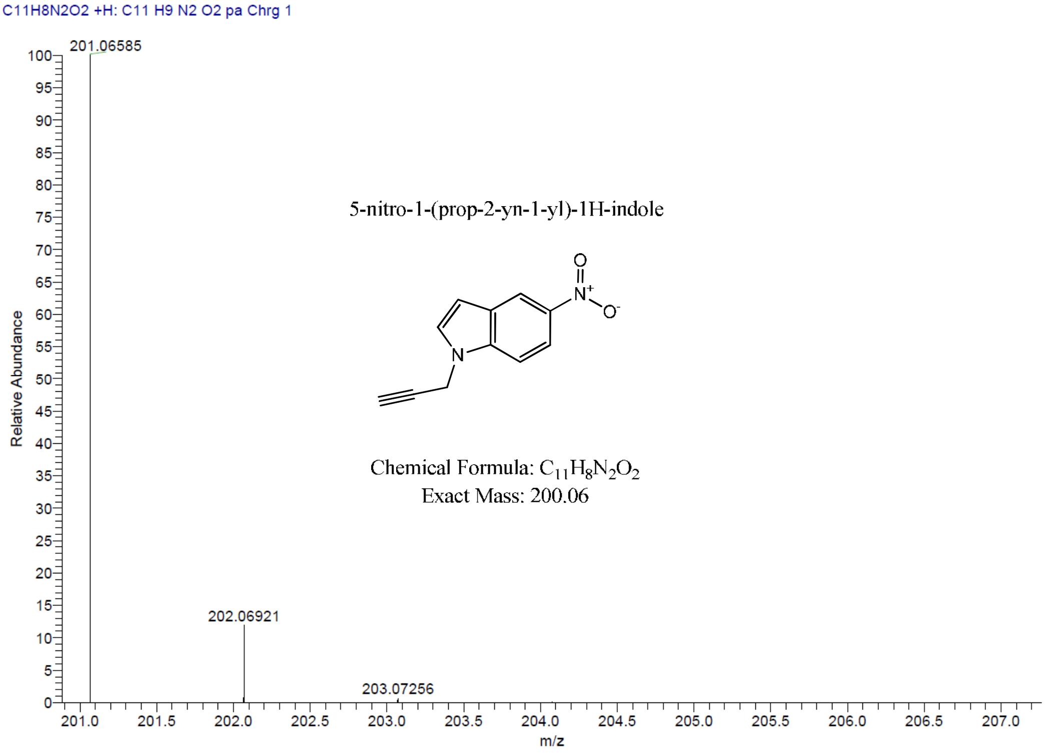

5-nitro-1-(prop-2-yn-1-yl)-1H-indole (19b)

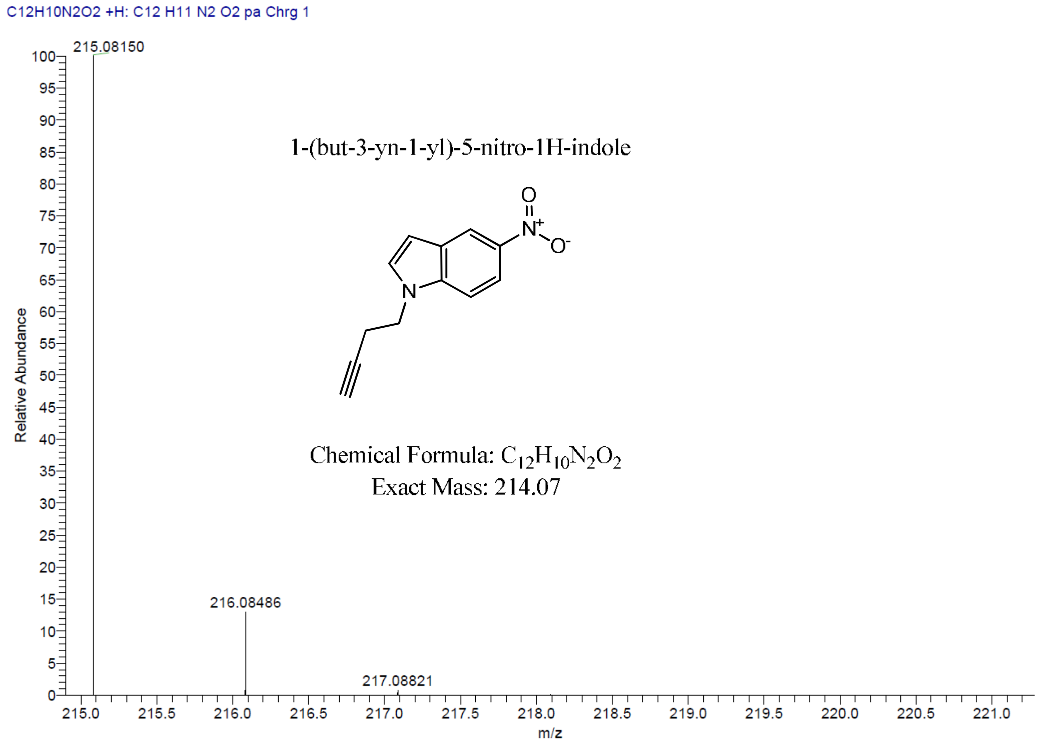

5-nitro-1-(prop-2-yn-1-yl)-1H-indole-3-carbaldehyde (20)

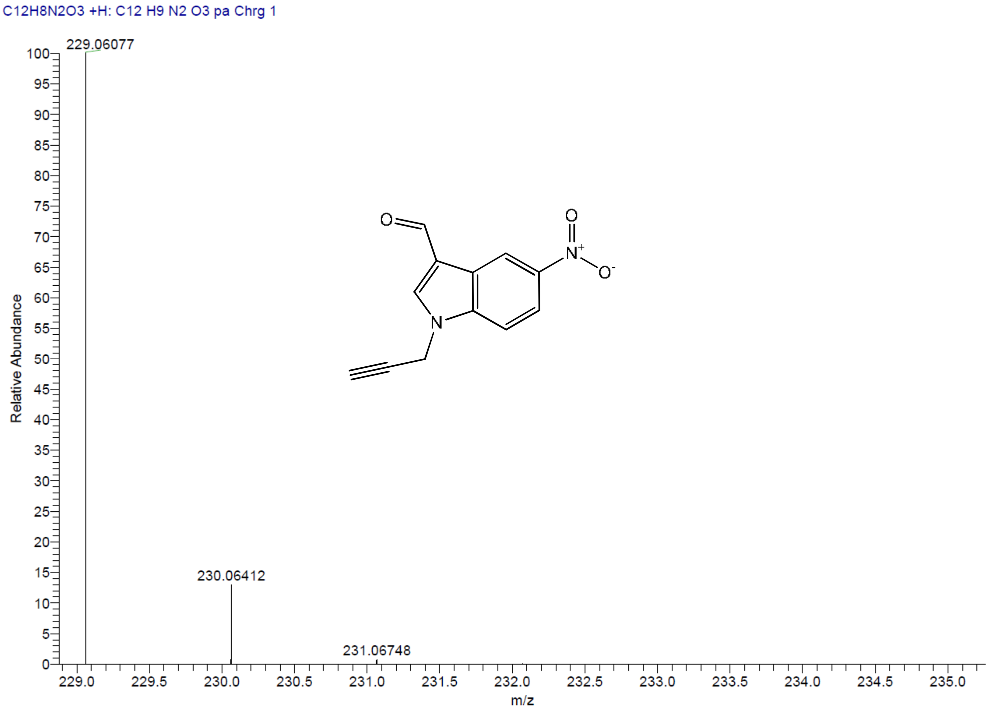

N,N-dimethyl-1-(5-nitro-1-(prop-2-yn-1-yl)-1H-indol-3-yl)methanamine (21a)

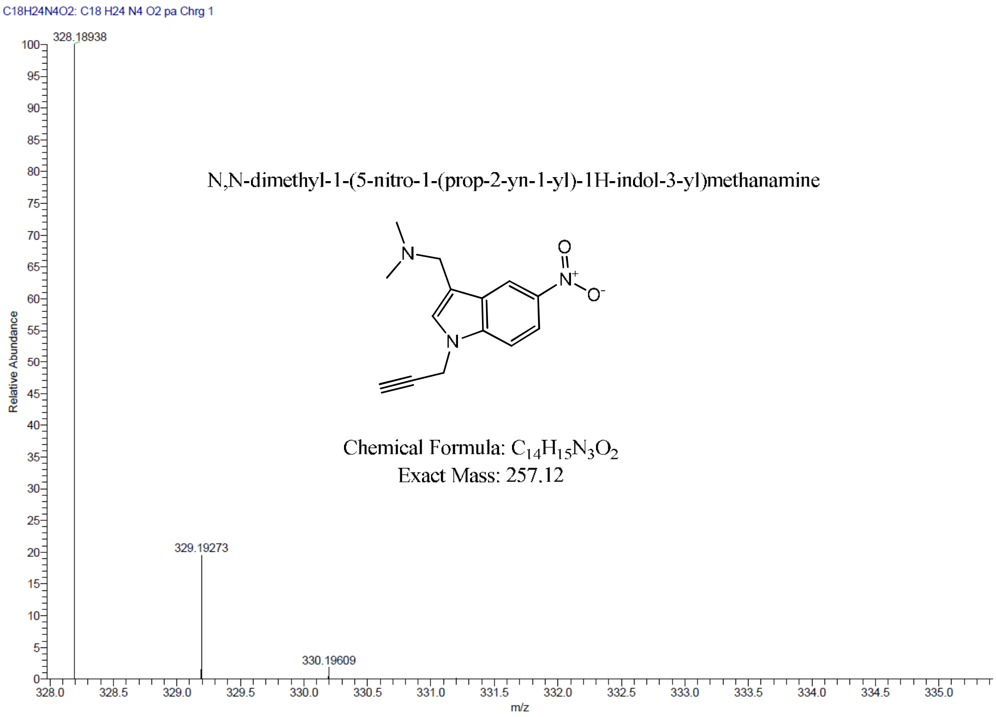

N1,N1-diethyl-N2-((5-nitro-1-(prop-2-yn-1-yl)-1H-indol-3-yl)methyl)ethane-1,2-diamine (21b)

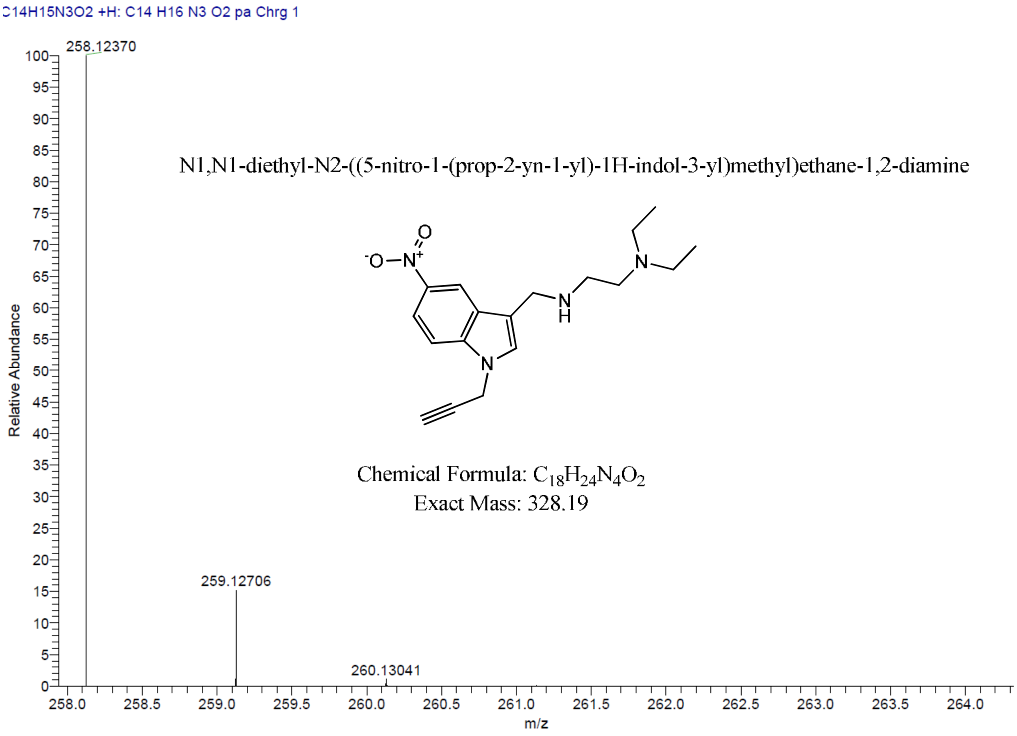

26b) N-benzyl-2-bromoacetamide:

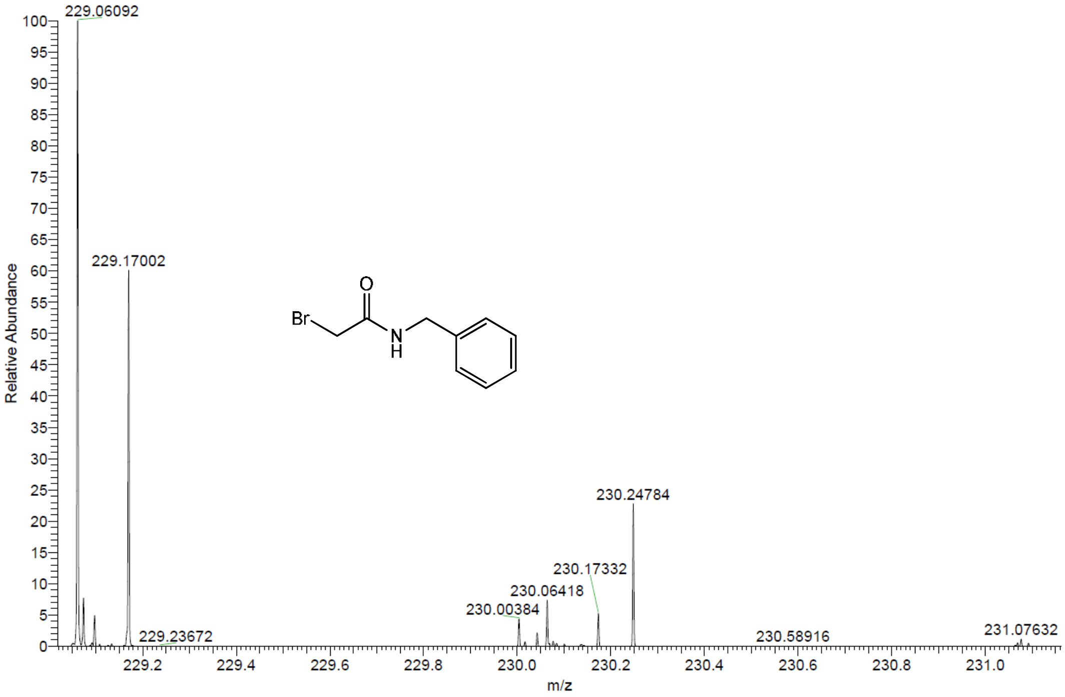

27b) 2-bromo-N-(4-fluorobenzyl)acetamide:

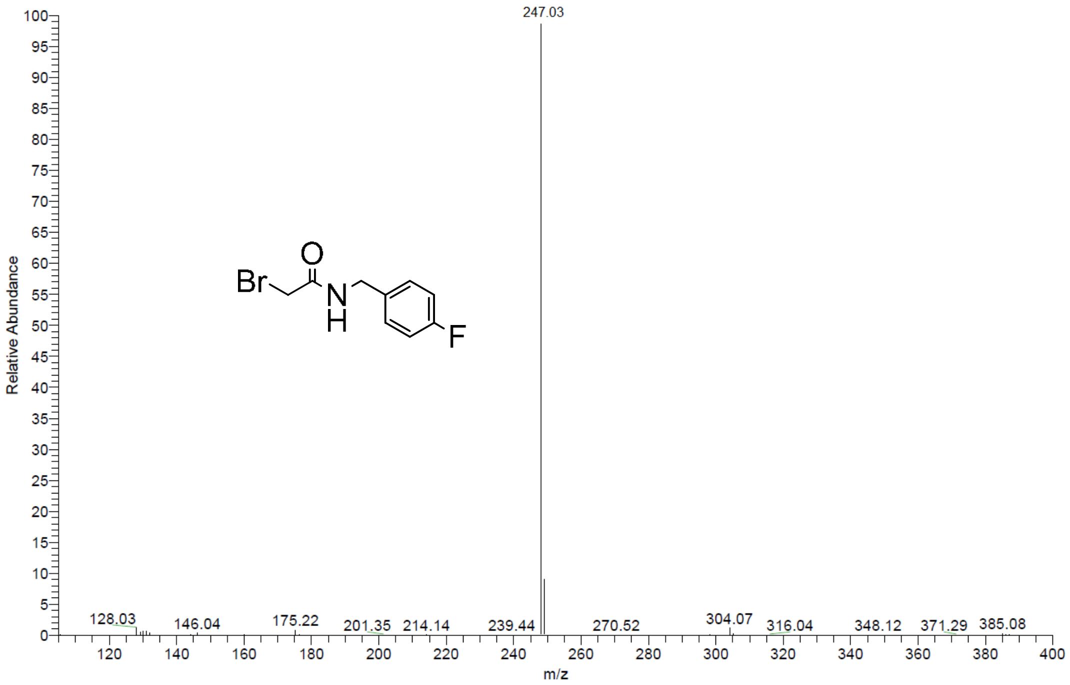

28b) 2-bromo-N-(4-(trifluoromethyl) benzyl) acetamide:

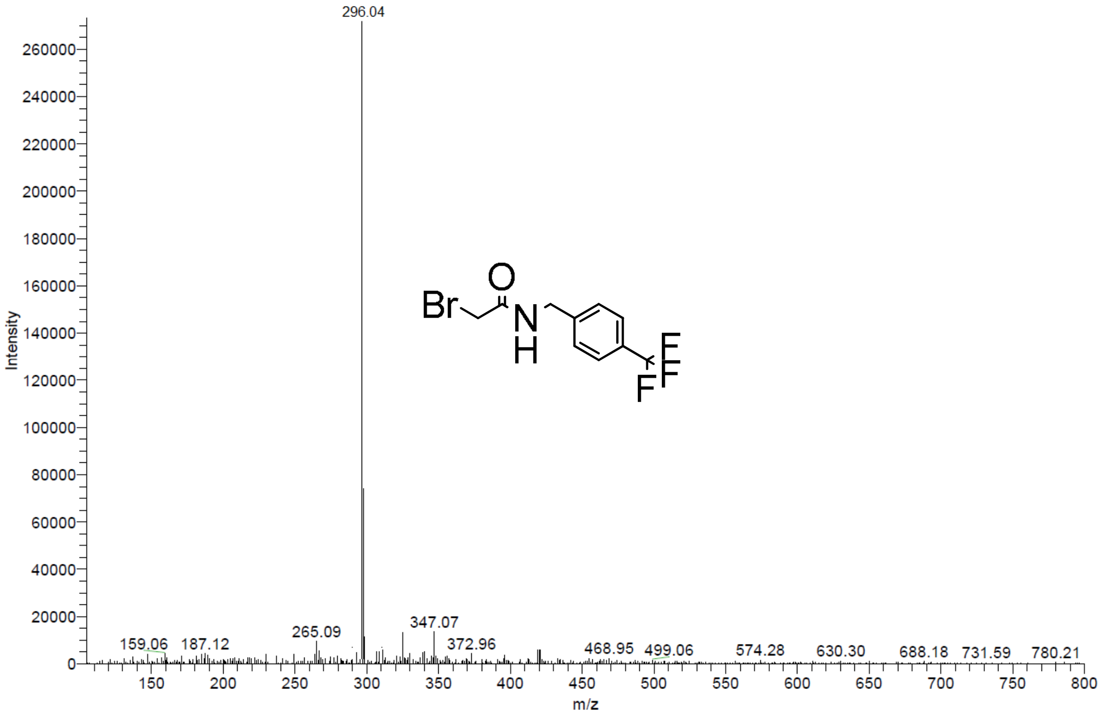

29b) 2-bromo-N-(3,4,5-trimethoxybenzyl)acetamide:

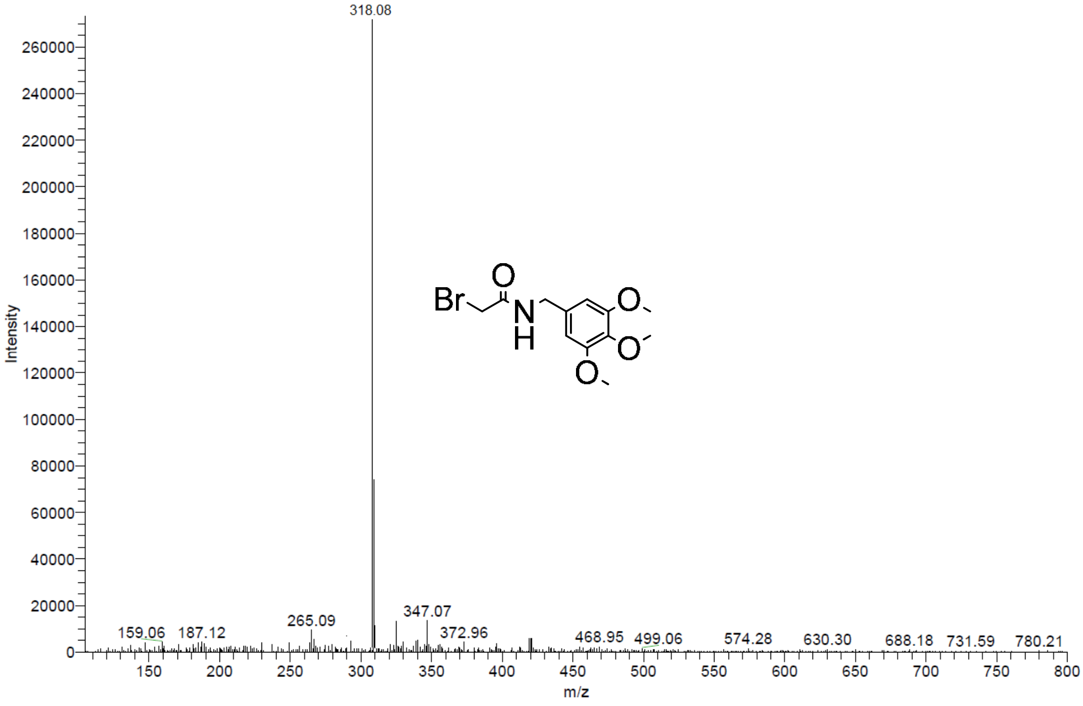

30b) 2-bromo-N-(4-methoxybenzyl)acetamide:

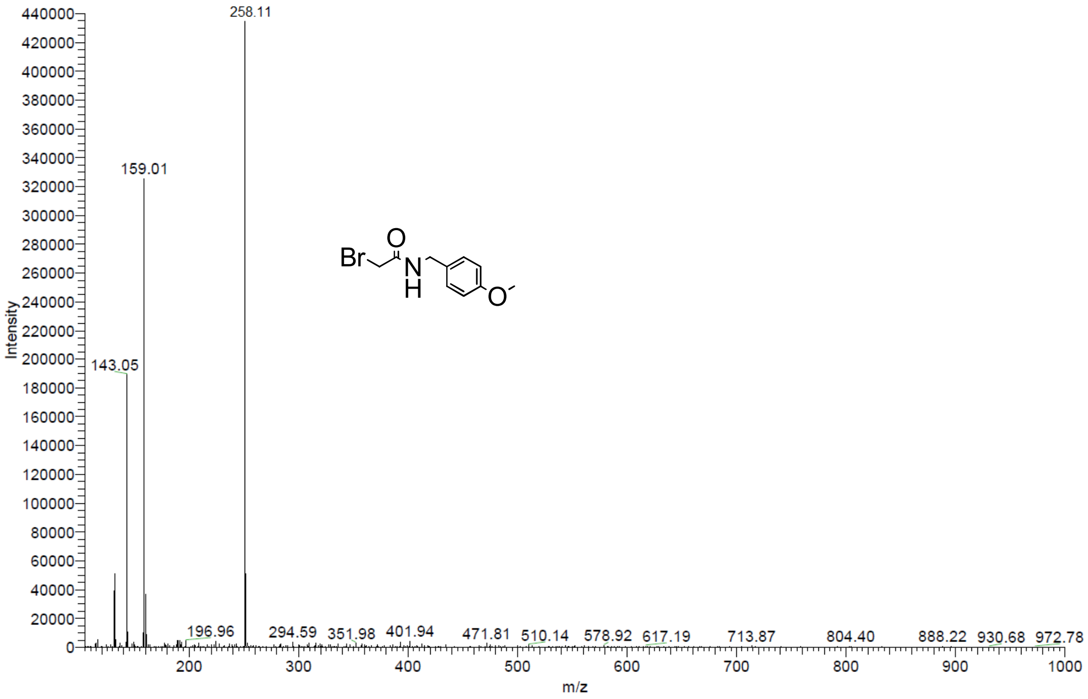

26c) 2-amino-1-benzyl-5-oxo-4, 5-dihydro-1H-pyrrole-3-carbonitrile

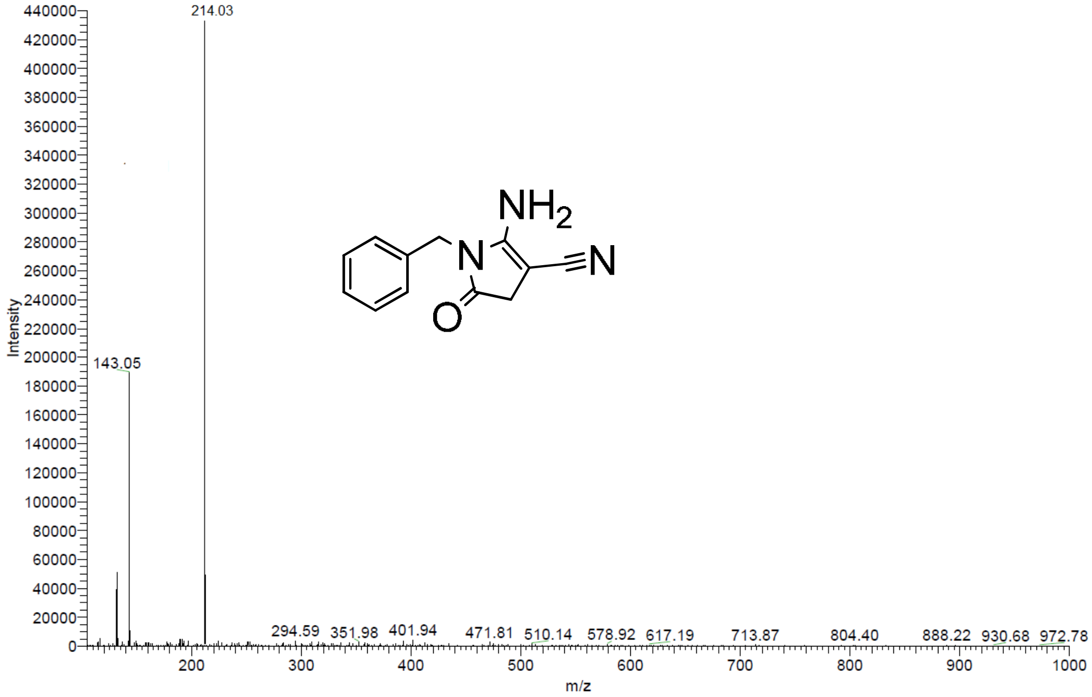

27c) 2-amino-1-(4-fluorobenzyl)-5-oxo-4, 5-dihydro-1H-pyrrole-3-carbonitrile:

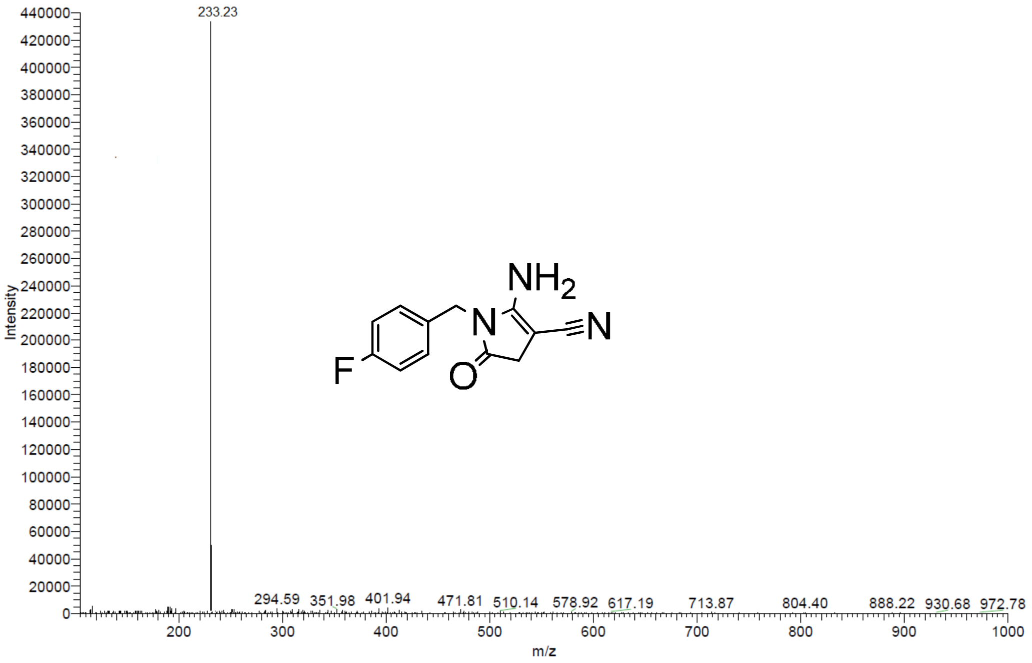

28c) 2-amino-5-oxo-1-(4-(trifluoromethyl)benzyl)-4,5-dihydro-1H-pyrrole-3-carbonitrile:

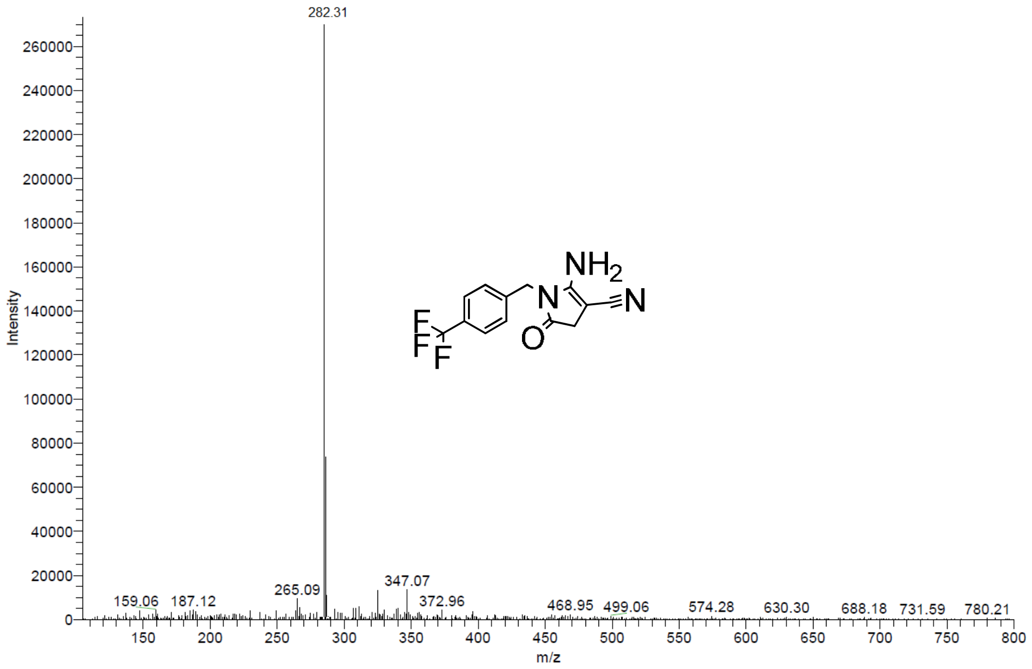

29c) 2-amino-5-oxo-1-(3,4,5-trimethoxybenzyl)-4,5-dihydro-1H-pyrrole-3-carbonitrile:

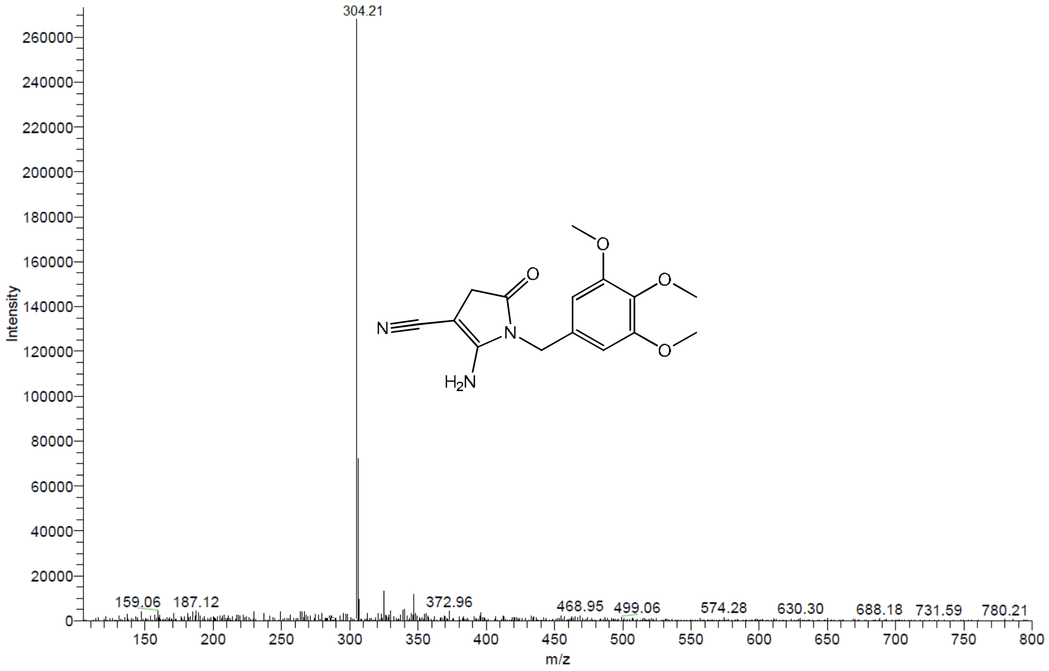

30c) 2-amino-1-(4-methoxybenzyl)-5-oxo-4,5-dihydro-1H-pyrrole-3-carbonitrile:

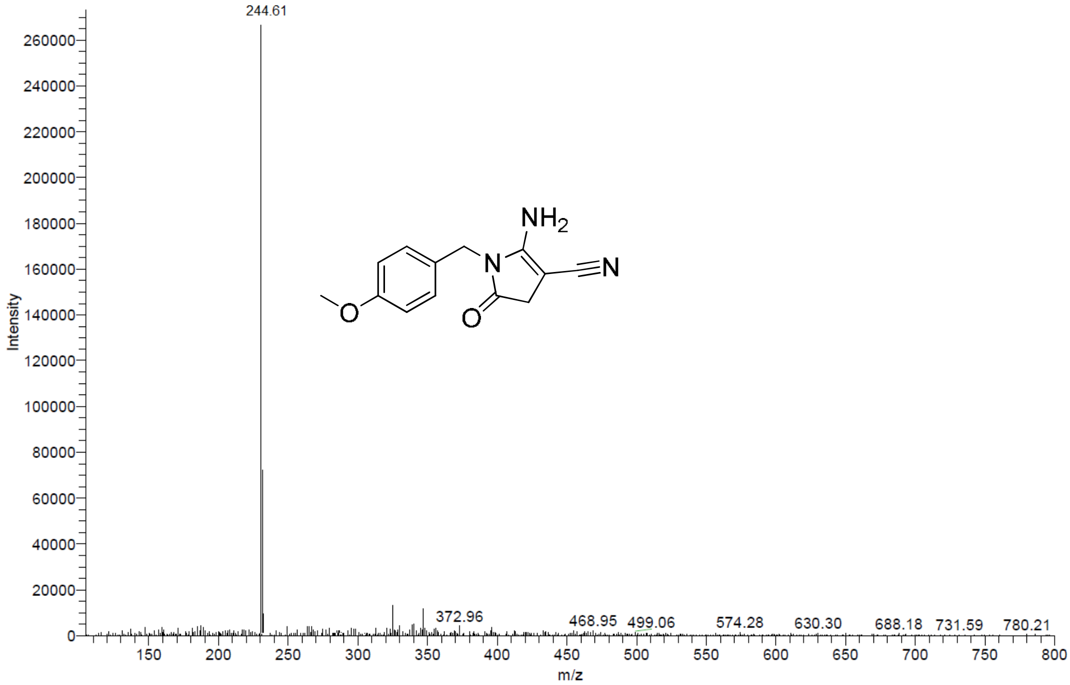

26d) ethyl 4-amino-1-benzyl-6-methyl-2-oxo-2,3-dihydro-1H-pyrrolo[2,3-b]pyridine-5-carboxylate:

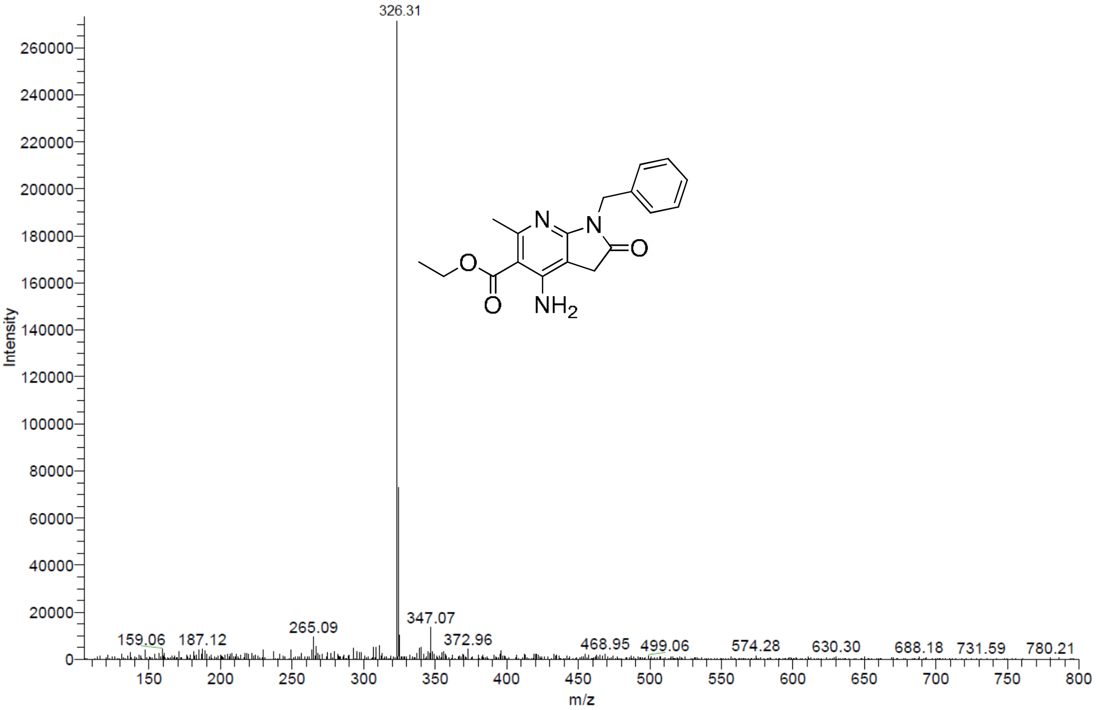

27d) ethyl 4-amino-1-(4-fluorobenzyl)-6-methyl-2-oxo-2,3-dihydro-1H-pyrrolo[2,3-b]pyridine-5-carboxylate:

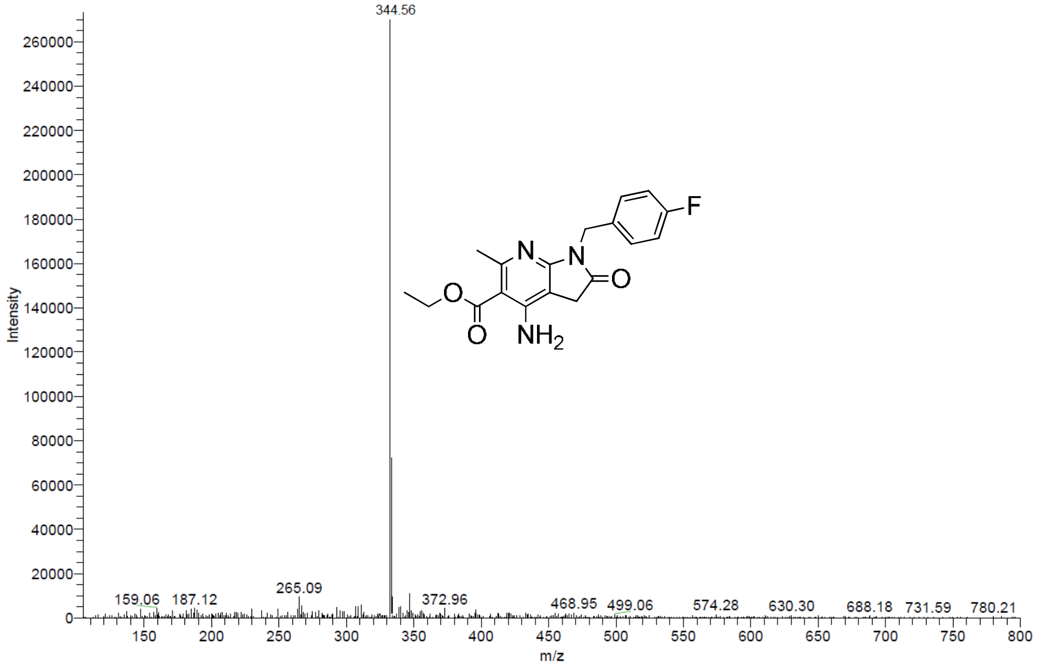

28d) ethyl 4-amino-6-methyl-2-oxo-1-(4-(trifluoromethyl)benzyl)-2,3-dihydro-1H-pyrrolo[2,3-b]pyridine-5-carboxylate

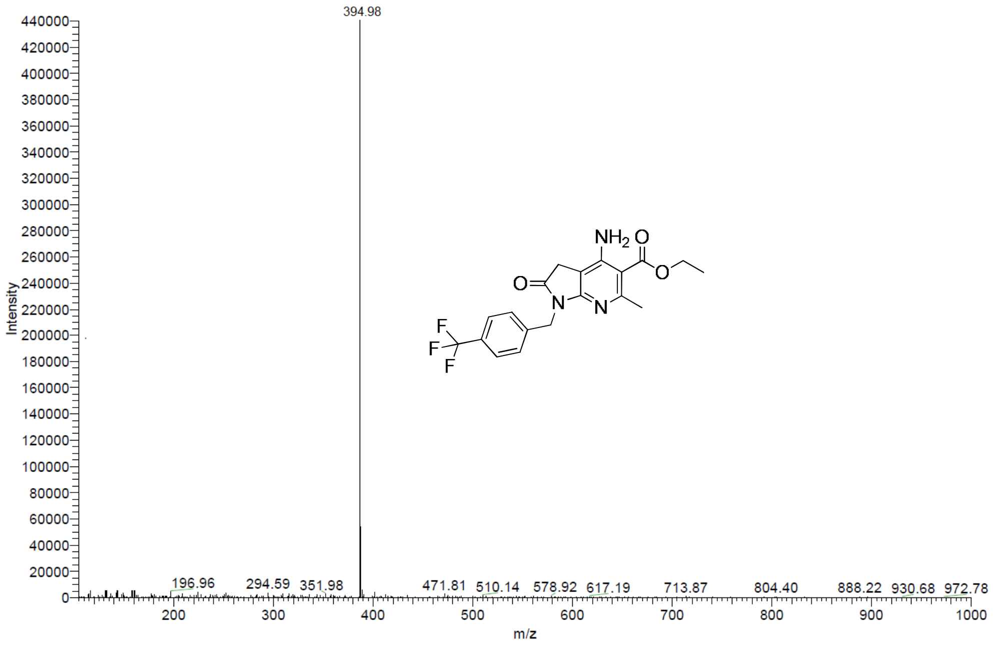

29d) ethyl 4-amino-6-methyl-2-oxo-1-(3,4,5-trimethoxybenzyl)-2,3-dihydro-1H-pyrrolo[2,3-b]pyridine-5-carboxylate:

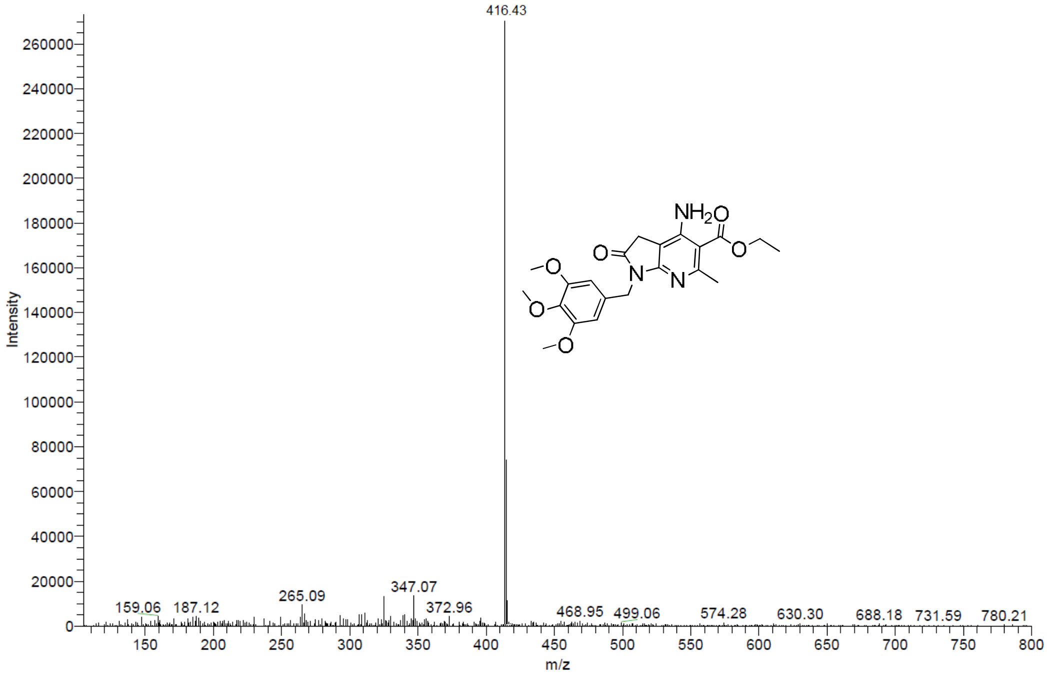

30d) ethyl 4-amino-1-(4-methoxybenzyl)-6-methyl-2-oxo-2,3-dihydro-1H-pyrrolo[2,3-b]pyridine-5-carboxylate:

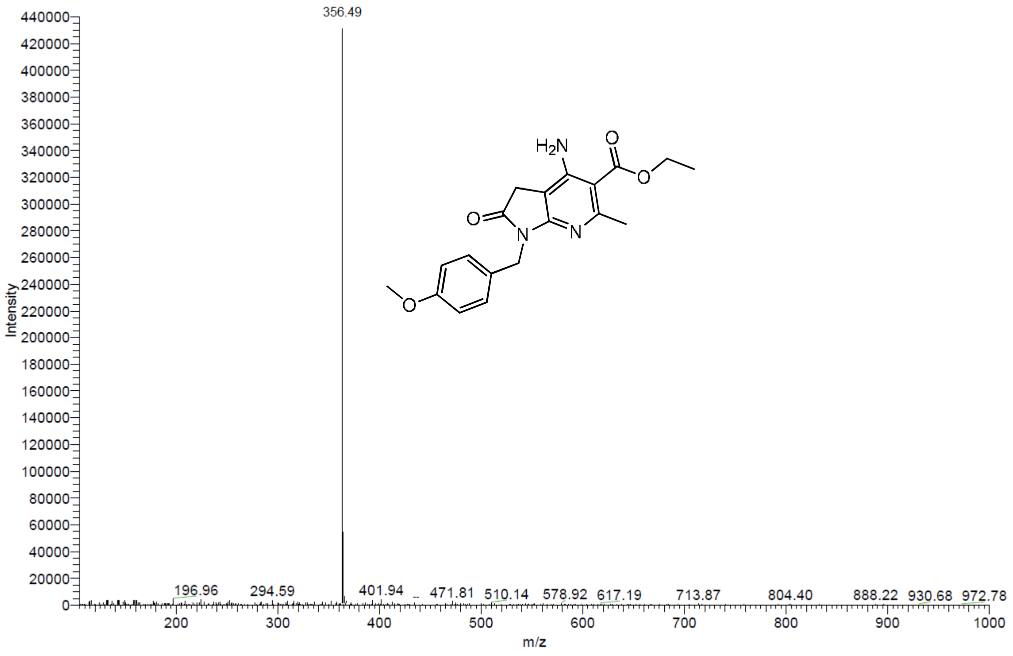

5b) 1-(3-(pyrrolidin-1-yl)propyl)-1H-indole

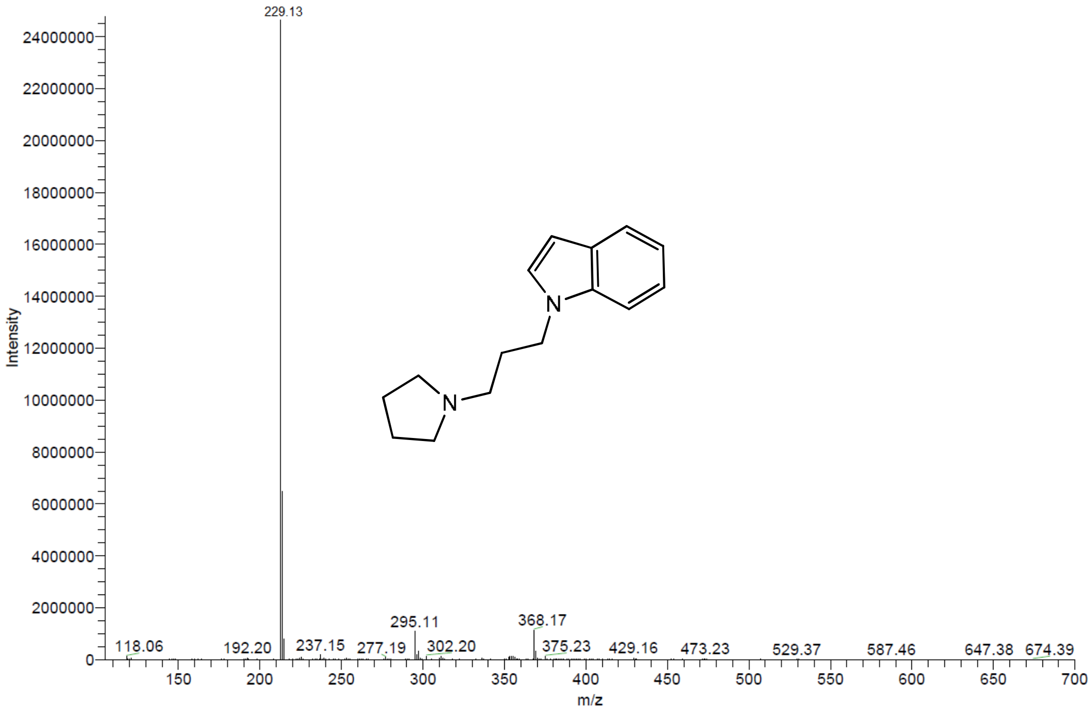

Supplement: Supplementary file 1 — Supplementary [file CMDC-16-1667-s001.pdf]
